# Microbial diversity in Mediterranean sponges as revealed by metataxonomic analysis

**DOI:** 10.1038/s41598-021-00713-9

**Published:** 2021-10-27

**Authors:** Nadia Ruocco, Roberta Esposito, Giacomo Zagami, Marco Bertolino, Sergio De Matteo, Michele Sonnessa, Federico Andreani, Stefania Crispi, Valerio Zupo, Maria Costantini

**Affiliations:** 1 Department of Marine Biotechnology, Stazione Zoologica Anton Dohrn, Villa Comunale, 80121 Naples, Italy; 2 Department of Biology, University of Naples Federico II, Complesso Universitario Di Monte Sant’Angelo, Via Cinthia 21, 80126 Naples, Italy; 3 Dipartimento Di Scienze Biologiche, Chimiche, Farmaceutiche Ed Ambientali, Università Di Messina, 98100 Messina, Italy; 4 DISTAV, Università Degli Studi Di Genova, Corso Europa 26, 16132 Genoa, Italy; 5 Bio-Fab Research Srl, Via Mario Beltrami, 5, 00135 Rome, Italy; 6 Institute of Biosciences and BioResources Naples, National Research Council of Italy, Naples, Italy

**Keywords:** Biotechnology, Ecology, Molecular biology

## Abstract

Although the Mediterranean Sea covers approximately a 0.7% of the world’s ocean area, it represents a major reservoir of marine and coastal biodiversity. Among marine organisms, sponges (Porifera) are a key component of the deep-sea benthos, widely recognized as the dominant taxon in terms of species richness, spatial coverage, and biomass. Sponges are evolutionarily ancient, sessile filter-feeders that harbor a largely diverse microbial community within their internal mesohyl matrix. In the present work, we firstly aimed at exploring the biodiversity of marine sponges from four different areas of the Mediterranean: Faro Lake in Sicily and “Porto Paone”, “Secca delle fumose”, “Punta San Pancrazio” in the Gulf of Naples. Eight sponge species were collected from these sites and identified by morphological analysis and amplification of several conserved molecular markers (18S and 28S RNA ribosomal genes, mitochondrial cytochrome oxidase subunit 1 and internal transcribed spacer). In order to analyze the bacterial diversity of symbiotic communities among these different sampling sites, we also performed a metataxonomic analysis through an Illumina MiSeq platform, identifying more than 1500 bacterial taxa. Amplicon Sequence Variants (ASVs) analysis revealed a great variability of the host-specific microbial communities. Our data highlight the occurrence of dominant and locally enriched microbes in the Mediterranean, together with the biotechnological potential of these sponges and their associated bacteria as sources of bioactive natural compounds.

## Introduction

Several marine organisms, such as macro and microalgae, sponges and fishes have developed various defence mechanisms during their evolution, including the exploitation of a large variety of natural molecules. In addition to their ecological roles, these compounds display several biological activities, such as anticancer, anti-inflammatory, antioxidant, antimicrobial, antihypertensive, making them good candidates for biotechnological applications in the pharmaceutical, nutraceutical and cosmeceutical fields^1^. Marine sponges are multicellular, benthic and generally sessile organisms, spread throughout the seabed, from the tropics to the poles. In the 1950s, the interest in sponges was relighted thanks to the discovery of new bioactive nucleosides (spongotimidine and spongouridine) in the marine sponge *Tectitethya crypta* (i.e., *Tethya cripta*)^2^. These nucleosides were the basis for the synthesis of Ara-C, the first marine antitumor agent and antiviral drug^3^. It is important to consider that marine sponges are known for hosting microbial communities whose composition can be quite complex^4^. These symbiotic bacteria are usually involved in carbon and nitrogen fixation, nitrification, anaerobic metabolism, stabilization of the sponge skeleton, protection against UV. However, they are mainly responsible for the production of bioactive metabolites^5^. For example, it has been demonstrated that a Alphaproteobacteria symbiotic of the sponge *Dysidea avara* produce an inhibitor of angiogenesis 2-methylthio-1,4-naphthoquinone^6^. The structural classes of natural products commonly associated with microbial sources include non-ribosomal peptides (penicillin and vancomycin), polyketides (erythromycin and tetracycline) and hybrid peptide polyketides (cyclosporin A and rapamycin). Some of these molecules are synthesized by non-ribosomal peptide synthases (NRPSs) and polyketide synthases (PKSs), which are encoded by genes clustered in the genome^7,8^. Several studies have highlighted the biotechnological potential of bacterial communities in marine sponges through the identification of PKSs and NRPSs genes, encoding for secondary metabolites^9–13^.

Concerning the phylum Porifera, the Mediterranean is known to host a huge biodiversity, counting about 700 species^14^ and more than half of these live in the coralligenous (a hard bottom of biogenic origin mainly produced by the accumulation of calcareous encrusting algae)^15–17^. Unfortunately, anthropogenic activities together with climate changes are strongly impacting the biodiversity of the Mediterranean^18,19^ and, as a consequence, this facilitates the spreading of alien species^20^. Examples of these environmental events are *Paraleucilla magna*, a sponge firstly described in 2004 off the Brazilian waters and now widespread in many areas of the Mediterranean^21^.

In the present work, we aim at deeper exploring the microbial communities associated with sponges in the Mediterranean Sea. Eight species of sponges were collected from four different areas of the Mediterranean, in Italy: Faro Lake (in Sicily) and “Porto Paone”, “Secca delle fumose”, “Punta San Pancrazio” (in the Gulf of Naples). Species characterization was performed by morphological observation of the skeleton and amplification of several conserved molecular markers (18S and 28S rRNA, ITS and CO1), with the only exception of *Geodia cydonium*, which has been previously characterized^22–24^. In order to analyse the biodiversity of symbiotic communities among different sampling sites, we performed a metataxonomic analysis of sponge samples through an Illumina MiSeq platform. More than 1500 bacterial isolates from eight samples were phylogenetically identified to understand if they were host-specific and/or site-specific. The ASVs analysis was then discussed to evaluate the biotechnological potential of sponges under investigation, in view of literature data.

## Results

### Morphological identification

All eight studied sponges belong to the class Demospongiae (Table 1). Seven species commonly live in the Mediterranean Sea, while *Oceanapia* cf*. perforate* (Sarà, 1960) is a rare species distributed in the western Mediterranean.

**Table 1:** Sample IDs, taxonomy, sampling depth (m) and sites, geographical coordinates and type of substrate.

Sample IDs	Sponge taxonomy	Sampling depth (m)	Sampling site	Coordinates	Substrate
O.per	*Oceanapia* cf. *perforata* (Sarà 1960)	2–3	Faro Lake	38°16'N, 15°38'E	Rocks, coralligenous concretions and caves
S.spi	*Sarcotragus spinosulus* (Schmidt 1862)	2–3	Faro Lake	38°16'N, 15°38'E	Hard substrates, coralligenous concretions and caves
E.dis	*Erylus discophorus* (Schmidt 1862)	2–3	Faro Lake	38°16'N, 15°38'E	Rocks, coralligenous concretions and caves
A.oro	*Agelas oroides* (Schmidt 1864)	7–9	Punta San Pancrazio	40°42ʹN, 13°57ʹE	Rocks, sand and coralligenous concretions
T.aur	*Tethya aurantium* (Pallas 1766)	15–17	Porto Paone	40°47ʹN, 14°9ʹE	Sand, mud, rocks, and coralligenous concretions
A.dam	*Axinella damicornis* (Esper 1794)	15–17	Porto Paone	40°47ʹN, 14°9ʹE	Rocks, mud, coralligenous concretions and caves
A.acu	*Acanthella acuta* (Schmidt 1862)	7–9	Punta San Pancrazio	40°42ʹN, 13°57ʹE	Rocks, sand and coralligenous concretions
G.cyd	*Geodia cydonium* (Jamenson 1811)	20	Secca delle Fumose, Parco Sommerso di Baia	40°49ʹN, 14°5ʹE	Roks, sand, mud, coralligenous concretions, *Posidonia* meadows and caves

### Molecular characterization

BLAST similarity search corresponded with the morphological identification achieved with two (S.spi and E.dis) of the three sponge samples collected in the Faro Lake (Sicily; see Tables S1-S2). In particular, molecular analyses confirmed S.spi as *Sarcotragus spinosulus* and E.dis as *Erylus discophorus*. ITS region displayed the best alignment for the identification of S.spi specimen with 98% of identity. Concerning the sample O.per, collected in the same site, the sequence of the species *Oceanapia* cf. *perforata*, identified by morphological analysis was not available in GenBank. Nevertheless, 18S and 28S rRNA primer pairs allowed a partial identification at the genus level, with high similarity to *Oceanapia isodictyiformis*, plus several hits annotated as *Oceanapia* sp. at low percentage of identity (Tables S1-S2).

In the case of sponges collected from “Porto Paone” in the Gulf of Naples, CO1 and 18S rRNA were the best molecular markers since they allowed the identification of sponges at the species level. Concerning T.aur sample, the species *Tethya aurantium* was identified (95% of identities) by CO1, whereas the sample A.dam, collected in the same site, was well identified as *Axinella damicornis* by 18S rRNA primers, producing a high percentage of sequence similarity (99%) (Tables S1-S2).

Molecular analysis confirmed the samples A.acu and A.oro, as *Acanthella acuta* and *Agelas oroides*, respectively, with 28S and 18S rRNA reporting the highest percentage of sequence similarity (100%) (Tables S1-S2). Alignments are reported in Figures S1-S7.

### Diversity analysis

**Figure 1:**
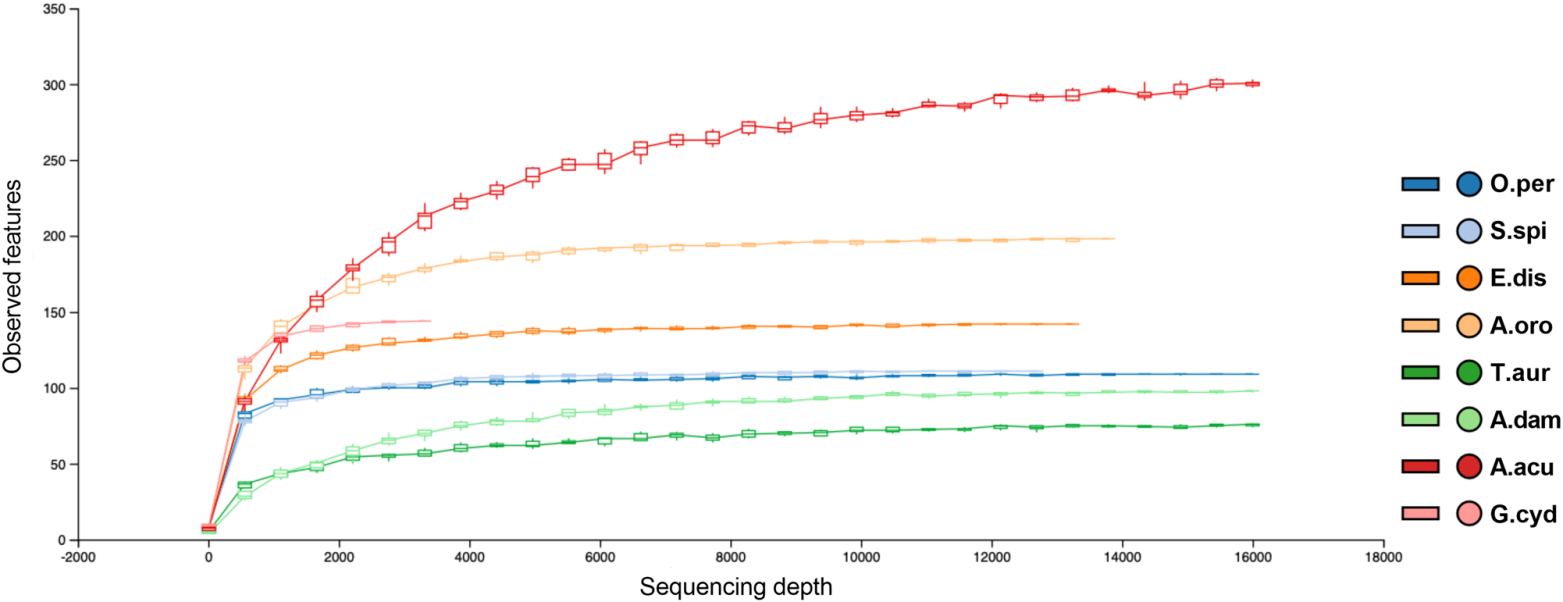
Alpha rarefaction of the observed features for each sponge sample under analysis. Sample IDs: O.per = *Oceanapia* cf. *perforata*, S.spi = *Sarcotragus spinosulus*, E.dis = *Erylus discophorus*, A.oro = *Agelas oroides*, T.aur = *Tethya aurantium*, A.dam = *Axinella damicornis* and A.acu = *Acanthella acuta*.

Alpha rarefaction on the observed features and three diversity indices (Chao1, Shannon and Simpson), were used to determine whether each sample was sequenced up to a sufficient depth. Rarefaction curves indicated that the majority of taxa was captured, since all samples under analysis reached a plateau (Fig. 1; Figure S8). Alpha diversity within each sample, measured through diversity indices at the family, genus and species levels, revealed a considerable bacterial diversity for all sponges under analysis, particularly when the species were considered (Table S3). When the impact of the abiotic features (temperature, pH and salinity) was examined, no statistical differences were observed between the groups. Overall, PERMANOVA analysis revealed that the environmental features did not affect the species composition or abundance in the symbiotic community.

**Figure 2:**
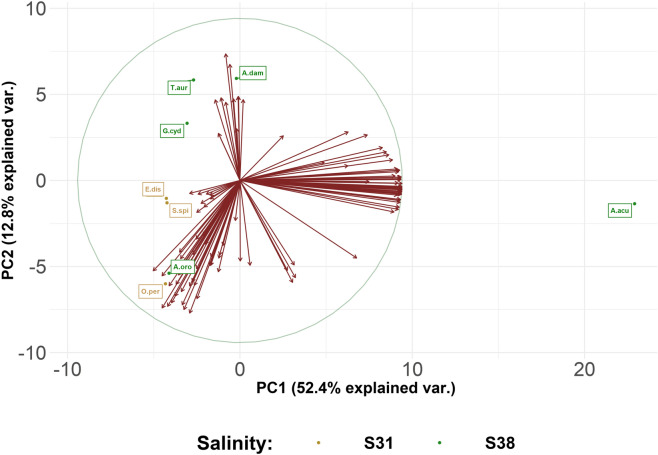
PCA analysis—Biplot of individuals (n = 8) and explanatory variables (n = 167) of two principal components (PC1 and PC2) of metataxonomic data. The majority of species diversity is explained by the first three PCs (76.7%), with PC1 and PC2 having the highest contribution (PC1 = 52.4% and PC2 = 12.8%). Biplot shows the PCA scores of the explanatory variables as vectors (in red) and individuals grouped for salinity class (S38 = Gulf of Naples, S31 = Strait of Messina). The circle represents the equilibrium of variables contribution. The importance of each variable is reflected by the magnitude of the corresponding values in the eigenvectors (higher magnitude-higher importance). Vectors pointing towards similar (small angle) and opposite directions (0 to 180 degrees) indicate positively or negatively correlated variables, and vectors at approximately right angles (90 to 270 degrees) suggest a low correlation. Sample IDs: O.per = *Oceanapia* cf. *perforata*, S.spi = *Sarcotragus spinosulus*, E.dis = *Erylus discophorus*, A.oro = *Agelas oroides*, T.aur = *Tethya aurantium*, A.dam = *Axinella damicornis* and A.acu = *Acanthella acuta*.

Further, a principal component analysis (PCA) was performed to reveal the bacterial species that greater contributed to the clustering of samples (Fig. 2). PCA showed seven components, with eigenvalues of 87.57, 21.42,19.12, 11.49, 10.82, 8.48, and 8.11. The first two components, including around 65.2% of the inertia of data, were used for further analyses, in order to detect the most interesting patterns. Firstly, PCA displayed three major groups, with 167 bacterial species that particularly influenced the clustering (Fig. 2).

Moreover, the sponge *A. acuta* (indicated as A.acu) clearly segregated, with several bacterial species (red vectors) contributing to the clustering (Fig. 2). Several taxa, such as Proteobacteria, Bacteroidia, Actinobacteria, Gracilibacteria, Cyanobacteria, Flavobacteria, Verrucomicrobiae, Campylobacteria, Planctomycetacia, Phycisphaerae, Nitrososphaeria, greatly affected the separation from the other sponges under analysis (Fig. 2). On the other hand, *G. cydonium*, *T. aurantium* and *A. damicornis* (reported as G.cyd, T.aur and A.dam, respectively), clustered into another group, where the bacteria belonging to the classes Anaerolineae, Alphaproteobacteria (Rhodobacteraceae, Hyphomicrobiaceae, Hyphomonadaceae, Kiloniellaceae, Magnetospiraceae families) and Gammaproteobacteria (Colwelliaceae and Vibrionaceae families) particularly contributed to the divergence (Fig. 2). The third cluster, corresponding to *O.* cf. *perforata*, *S. spinosulus*, *E. discophorus* and *A. oroides* (indicated as O.per, S.spi, E.dis and A.oro, respectively), was chacterized by an abundance of bacteria included into the phyla Proteobacteria, Chloroflexi, Tectomicrobia and Acidobacteria (Fig. 2).

### Taxonomic profiling

**Figure 3:**
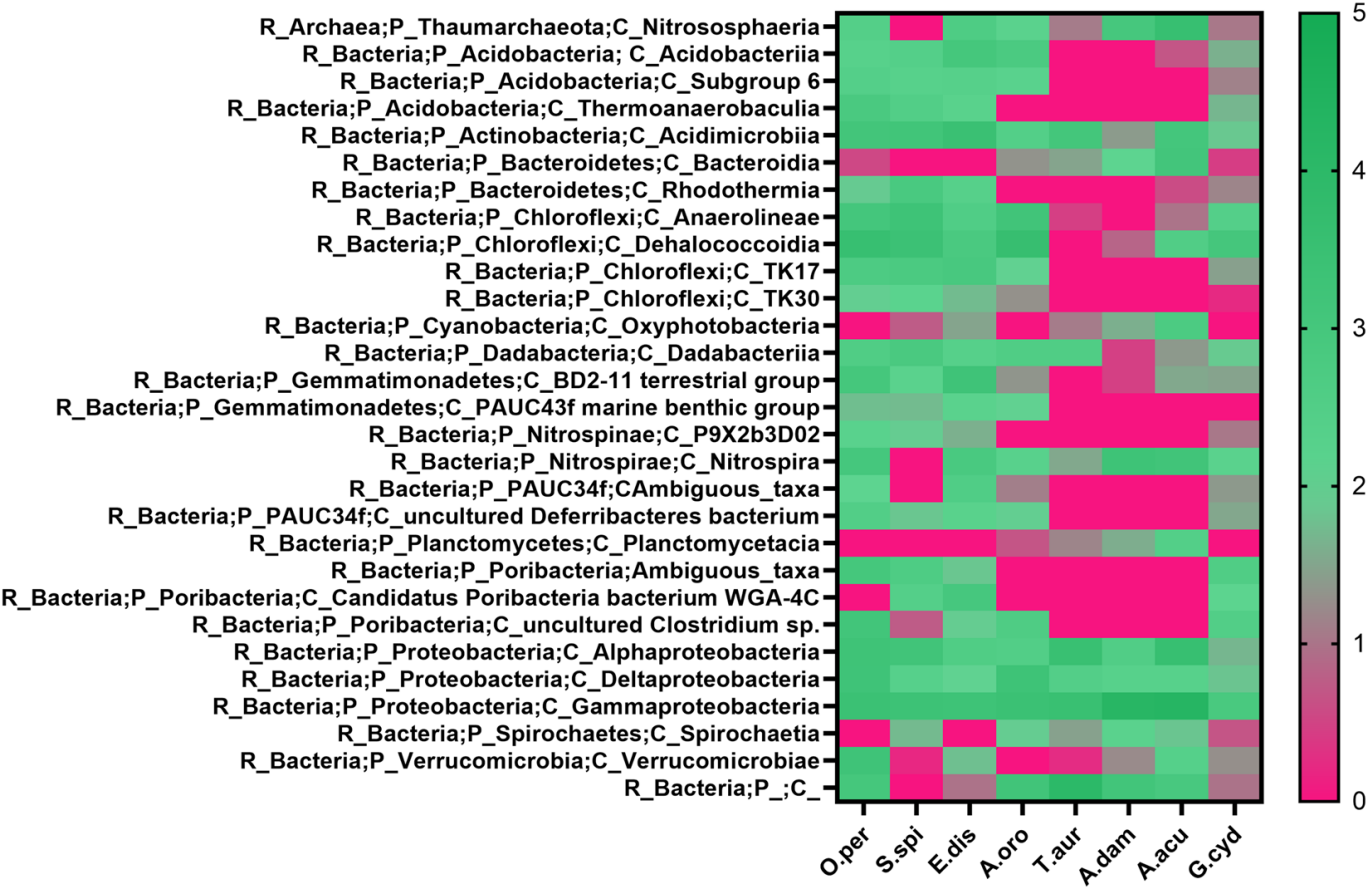
Heat-map comparing the abundance of the most representative bacterial classes identified from *O.* cf. *perforata* (O.per), *S. spinosulus* (S.spi), *E. discophorus* (E.dis), *A. oroides* (A.oro), *T. aurantium* (T.aur), *A. damicornis* (A.dam), *A. acuta* (A.acu) and *G. cydonium* (G.cyd). Color code: green = high number of features, pink = low number of features. Taxonomy code: R = regnum, P = phylum, C = class. Heat-map was performed using GraphPad Prism V. 9 (GraphPad Software, San Diego, CA, USA).

ASVs analysis was performed on those reporting a confidence percentage ≥ of 75%. The full taxonomy of sponge samples was reported in Tables S4-S11. Among the sponges collected in the Faro Lake (Sicily) was found i. the largest number of features (142) in *E. discophorus*, especially Acidimicrobiia, Gemmatimonadetes, Nitrospira, Acidobacteria and Gammaproteobacteria (Fig. 3); ii. 111 ASVs in *S. spinosulus*, with a greater abundance of Dehalococcoidia, Anaerolineae, Acidimicrobiia, Dadabacteriia, Rhodothermia and Gammaproteobacteria (Fig. 3); iii. 109 ASVs in *Oceanapia* cf. *perforata*, where Verrucomicrobiae, Clostridium, Deltaproteobacteria, Nitrospirae, Dehalococcoidia, Anaerolineae, Thermoanaerobaculia, Acidimicrobiia, Gemmatimonadetes and Deltaproteobacteria were highly represented (Fig. 3).

In sponges *T. aurantium* and *A. damicornis*, both collected in the Porto Paone, in the Gulf of Naples, 76 and 98 ASVs were detected, respectively. The most abundant bacterial classes in *T. aurantium* were Alphaproteobacteria, Gammaproteobacteria, Acidimicrobiia and Dadabacteriia, whereas bacterial community of *A. damicornis* was dominated by Gammaproteobacteria, Nitrospira and Deltaproteobacteria (Fig. 3).

**Figure 4:**
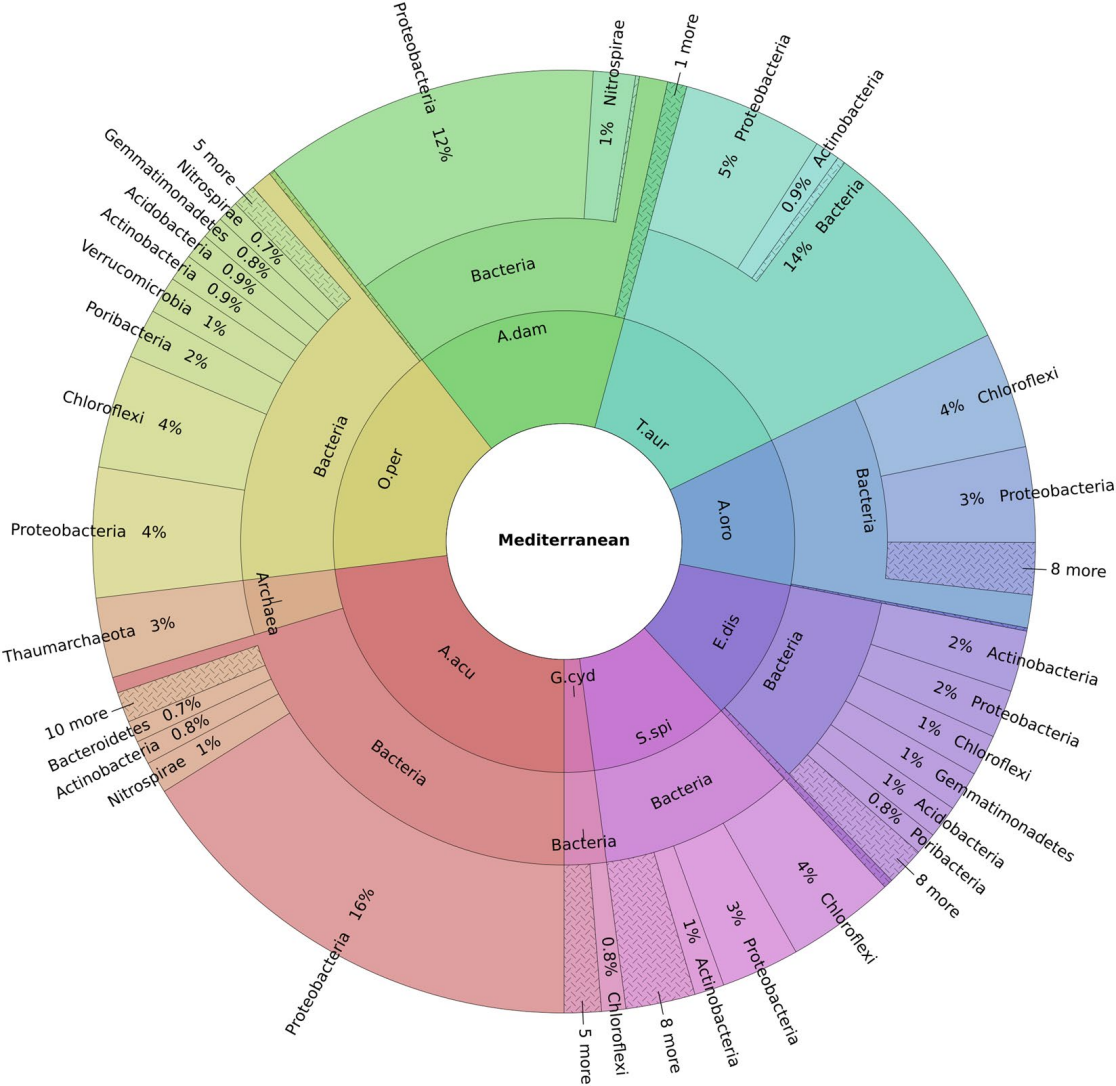
Krona Plot representing the most abundant phyla for each sponge under analysis. Sample code: T.aur = *T. aurantium*; A.dam = *A. damicornis*, A.acu = *A. acuta*, O.per = *O.* cf. *perforata*, S.spi = *S. spinosulus*, E.dis = *E. discophorus*, A.oro = *A. oroides* and G.cyd = *G. cydonium*.

Among the sponges collected in Punta San Pancrazio (Ischia island, Bay of Naples), *A. acuta*, showed 316 features, with a few abundant classes (Gammaproteobacteria, Nitrospira, Alphaproteobacteria and Acidimicrobiia) and a long tail of extremely low ASVs (Fig. 4; Figure S9). In contrast, *A. oroides* mainly revealed five bacterial groups (Dehalococcoidia, Anaerolineae, Gammaproteobacteria, Dadabacteria and Deltaproteobacteria), with a total of 198 ASVs (Fig. 4; Figure S9).

*G. cydonium*, collected in “Parco Sommerso” of Baia (Bay of Naples), showed 144 ASVs with a significant abundance of Gammaproteobacteria, Poribacteria and Nitrospira (Fig. 3).

Specifically, the taxonomic composition revealed an abundance of Proteobacteria and Cloroflexi found in *O.* cf. *perforata* (4%, respectively), *S. spinosulus* (3% and 4%, respectively) and *A. oroides* (3% and 4% respectively) (Fig. 4; Figure S9).

Differently, a high percentage (17–20%) of Actinobacteria and Proteobacteria were detected in *E. discophorus*. In addition, *T. aurantium* revealed 14% of an unknown phylum, and low percentages (5%) of another phylum (Proteobacteria) (Fig. 4; Figure S9). The sponge *A. damicornis* revealed 12% of Gammaproteobacteria class, while a low percentage (1%) of Nitrospirae phylum. The sponge *A. acuta* revealed 16% of Proteobacteria phylum and 1% of Nitrospirae phylum. Interestingly, this sponge was the only species revealing a certain abundance of Archea belonging to the phylum Thaumarchaeota (Fig. 4; Figure S9). Concerning *G. cydonium*, the most abundant class was Dehalococcoidia with 29.1% and Gammaproteobacteria with 19.4% (Fig. 4; Figure S9).

As reported above, the sponges *O.* cf. *perforata*, *S. spinosulus, A. oroides* and *G. cydonium* revealed a similar composition in bacterial species distribution. In fact, a high abundance of Cloroflexi and Proteobacteria was observed in these species (Fig. 4; Figure S9).

The absolute abundance of each bacterial phylum retrieved from the eight sponge samples was reported in Table S12.

## Discussion

In this study we analyzed the biodiversity of marine sponges in the Mediterranean, focusing on four sampling sites: one in the Messina Strait (North–East of Sicily) and three in the Gulf of Naples. This work represents an important step forward in the investigation of the Mediterranean, being considered as a biodiversity hotspot^25^. Long-term variations of biodiversity are significant signs of environmental change. Concerning the Mediterranean sea, data are available to compare possible variations in the species richness and faunal compositions, which are responsible of loss or turn-over of biodiversity^18,19,26^. Moreover, enclosed saline coastal basins, such as the case of the Faro Lake, represent good models of aquatic system to study temporal variation of sponge biodiversity^26^.

Firstly, we identified seven sponges, complementing the traditional identification by morphological features using a molecular approach, based on DNA sequencing of 28S and 18S rDNA, ITS and CO1. Our results demonstrated that none of the molecular markers alone was able to define the sponges under analysis up to the lowest taxonomic level. Indeed, molecular markers were found to be suitable depending on the species of sponge to be classified. However, it must be considered that the barcoding analysis could be negatively affected by the lack of curated sequences collection.

Among these used molecular markers, 28S and 18S rRNA are characterized by sufficiently heterogeneous regions useful to address phylogeny at different levels^27,28^. Because of their rapid evolution, ITS regions are considered markers at high resolution^29^. The COI mitochondrial DNA locus, despite the high variability at the sequence level, it is easy to amplify for its conservation across multicellular animals and abundant in eukaryotic DNA^30,31^. In fact, it resulted to be the most successful molecular marker to discriminate sponges at various taxonomic levels^32,33^. According to these literature data, no single marker exists for all sponge species, having each marker its strength and limitations^34^. This difficulty is also linked to the incomplete sequences annotated in database, so limiting phylogeny-based molecular taxonomic approaches that are commonly used for species identification. For this reason, a multi-locus-based molecular approach is recommended for the reliability in the case of sponge identification^34^. This was in complete agreement with our experimental strategy for the identification of sponges under analysis.

An important finding achieved by this study regarded the fact that, among the three sponges collected at Faro Lake, only *E. discophorus* was recorded in 2013 during a survey on the long-term taxonomic composition and distribution of the shallow-water sponge fauna from this meromictic–anchialine coastal basin^26^. The other two species, *O.* cf. *perforata* and *S. spinosulus*, were not reported so far, suggesting them to be new colonizers of this lake. Recently, the significant number of first reports of species from several biogeographic regions found in the Faro Lake^35–38^ is probably related to the import of bivalves from Atlantic and Mediterranean sites, for aquaculture activities. All three sponges usually live on rocks, coralligenous concretions and marine caves in the Mediterranean. In addition, *O.* cf. *perforata* is a rare species in the Mediterranean. Concerning the other sponges collected in the Gulf of Naples, *T. aurantium*, *A. damicornis*, *A. acuta* and *A. oroides*, represent typical species for the Mediterranean, as well as, *G. cydonium*.

Furthermore, through metataxonomic analysis, we also investigated the bacterial diversity among these Mediterranean sites. Recent advances in molecular ecology techniques, such as the sequencing of bacterial 16S rRNA gene, led to a clear picture of the taxonomic and functional composition of marine microbiota, including associated symbionts^39^.

Our results showed that sponges under analysis host diverse bacterial communities. Surprisingly, sponges collected in the Faro Lake were characterized by a more diversified composition of phyla in comparison to those collected in the Gulf of Naples (Fig. 4; Figure S9). Moreover, *G. cydonium* revealed a little sequencing depth (Fig. 1), probably related to the uniqueness of the collecting site (Table 1), which has strictly influenced the symbiotic community by selecting a few species of well adapted bacteria. In fact, Secca delle Fumose represents a good case study, due to the variations in seawater pH and gas-rich hydrothermal vents^40^. As reported in literature, extreme environments are well-known to inhabit a macro- and micro-biota with high biotechnological value^41–44^.

Moreover, PCA analysis suggested interesting results for the sponges collected from Punta San Pancrazio (Ischia Island). In fact, *A. oroides* revealed considerable similarities to the sponges retrieved in the Strait of Messina, since they clustered in the same group (Fig. 2). On the other hand, *A. acuta* separated from the other sponges under analysis, revealing a completely different symbiotic community that needs to be taken into consideration (Fig. 2).

The phylogeny of sponges must also be considered in our analysis, because it probably influenced the community structure. In fact, *S. spinosolus*, *A. oroides*, *E. discophorus* belonging to Dictyoceratida, Agelasida, Tetractinellida orders, respectively, were recorded as High Microbial Abundance (HMA) species, while *T. aurantium*, *A. damicornis* and *A. acuta* were instead indicated as Low Microbial Abundance (LMA) species^45–47^. HMA sponges hosted a more diversified symbiont community than LMA, which were discovered to be extremely stable over seasonal and inter-annual scales^46^. The correlation of sponge taxonomy to the abundance and diversification of microbial communities was evident in the heat-map, since the HMA species displayed higher values of bacterial features (Fig. 3). Moreover, these considerations could justify the clustering obtained through the PCA analysis, where *T. aurantium* and *A. damicornis* separated from *S. spinosolus*, *A. oroides* and *E. discophorus* (Fig. 2).

Many studies reported about the sponge associated-bacteria as good candidates for the isolation of natural compounds, useful in biotechnological applications. This study represents a first evaluation of the biotechnological potential of the aforementioned sponges. For this reason, we will further discuss the known bioactivities of the most abundant bacterial phyla identified in the considered sponges, according to the available literature.

The symbiotic community of the five sponges from the Gulf of Naples, mainly in *T. aurantium*, *A. damicornis*, *A. acuta*, *A. oroides* and *G. cydonium* was dominated by Proteobacteria, classes Alphaproteobacteria, Deltaproteobacteria and Gammaproteobateria (Figs. 3, 4; Figure S9).

Alphaproteobacteria were commonly found in the Mediterranean, mainly in the sponges *Sarcotragus fasciculatus*, *Ircinia oros* and *Ircinia strobilina*^48,49^. Overall, proteobacteria are known to produce N-acyl homoserine lactone (AHL) signal molecules involved in bacterial quorum sensing^50^. In fact, several species belonging to Alpha- and Gamma-proteobacteria, isolated from the Mediterranean sponges *Halichondria panicea*, *Ircinia fasciculata*, *Axinella polypoides*, and *Acanthella* sp.^51^ and from the Red sea sponge *Suberea mollis*^52^ showed antimicrobial activities, making them suitable tools for pharmacological purposes^53–57^.

The phylum Actinobacteria (class Acidimicrobiia) was the most abundant in *S. spinosulus* and *E. discophorus* (Figs. 3, 4; Figure S9), also found in *T. aurantium* and *A. acuta*. Actinobacteria are Gram positive, mostly aerobic, mycelial and primarily soil organisms, but recent studies revealed that some Actinobacteria taxa were also well-adapted to marine environments. Moreover, these bacteria were attracting interest as key producers of therapeutics, for their great potential in extracellular enzyme production, as well as in the synthesis of a variety of bioactive metabolites with antimicrobial and antifungal activity^11,58,59^. In fact, Actinobacteria together with the already discussed Proteobacteria, showed antagonistic activity against bacterial belonging to the genera *Bacillus*, *Pseudovibrio*, *Ruegeria*, *Staphylococcus aureus*, *Escherichia coli* K12, and fungi *Fusarium* sp. P25, *Trypanosoma brucei* TC 221, *Leishmania major*, *Aspergillus fumigatus*, *Candida glabrata* and *C. albicans*^13,52,60–71^. Furthermore, this group of bacteria, also isolated from *Suberites domuncula* and *Dysidea* sp., showed antimicrobial, antifungal and cytotoxic activity against different cell lines as HeLa cells and pheochromocytoma (PC12) cells^53–56^.

Dehalococcoides and Anaerolineae (a class of the phylum Chloroflexi) seem to be peculiar species of both collection sites, being detected in *S. spinolosus*, *E. discophorous*, and *A. oroides* (Figs. 3, 4; Figure S9). This was an interesting finding, because both bacterial groups were isolated for the first time from Mediterranean sponges. In fact, *Anaerolineae* were found most abundant in *Aaptos suberitoides* and *Xestospongia testudinaria* collected in South East Misool, Raja Ampat, West Papua (Indonesia)^72,73^. No data were reported so far for marine biotechnology applications. In contrast, the anaerobic Dehalococcoides showed surprising capabilities to transform various chlorinated organic compounds via reductive dechlorination. For this reason, Dehalococcoides were extensively used for the restoration of environments contaminated by chlorinated organics, which are normally released through industrial and agricultural activities^74,75^. ASVs analysis showed a peculiar abundance of the phylum Verrucomicrobia (class Verrucomicrobiae) in the sponge *O.* cf. *perforata* (Figs. 3, 4; Figure S9). Little information was reported on the abundance and ecology of aquatic Verrucomicrobia, being prevalent in lakes characterized by nutrient abundance and phosphorus availability^76,77^. These bacteria play an important role in global carbon cycling, processing decaying organic materials and degrading various polysaccharides^78–81^. It was found that the sponge-symbiotic Verrucomicrobiae bacteria (e.g. *Petrosia ficiformis*) exhibited enrichment of the toxin-antitoxin (TA) system suggesting the hypothesis that these bacteria use these systems as a defense mechanism against antimicrobial activity deriving from the abundant microbial community co-inhabiting their host^77^. *Rubritalea squalenifaciens* (strain HOact23T; MBIC08254T) is a rare marine bacterium belonging to the phylum Verrucomicrobia, isolated from *Halichondria okadai* (collected in Japan), from which a novel acyl glyco-carotenoic acids, diapolycopenedioic acid xylosyl esters A, B, and C, were isolated as red pigments with a potent antioxidative activity^82^.

Furthermore, Nitrospirae (class Nitrospira) was the most abundant bacterial phylum in the three sponges from the Gulf of Naples, *A. damicornis*, *G. cydonium* and *A. acuta*, as well as, in *O.* cf. *perforata* and *E. discophous* from the Faro Lake (Figs. 3, 4; Figure S9). The first described *Nitrospira* species was *N. marina*, isolated by Watson et al.^83^ from water collected in the Gulf of Maine. In particular, *Nitrospira* spp. play pivotal roles in nitrification as anaerobic chemolithoautotrophic nitrite-oxidizing bacterium^84^. These bacteria also have been found in several sponge species such as *Theonella swinhoei* and *Geodia barretti*^85,86^. Concerning their biotechnological potential, very little information is available so far. A recent work, using BLASTp search against the Integrated Microbial Genomes (IMG) database, identified a *Pseudoalteromonas luteoviolacea* gene encoding for a L-amino acid oxidase (LAAO) with antimicrobial properties in a strain belonging to the phylum *Nitrospinae*^87^.

Summarizing, our data pointed out the attention on the species biodiversity of the Mediterranean Sea and on 16S rRNA sequence datasets, which allowed to the detection of several signature resident microbial fauna. In addition, data reported on the biotechnological potential of the bacteria identified in the eight sponges under analysis, suggest the need for further validations through bioassay-guided fractionation to identify novel metabolites useful for the pharmaceutical, cosmeceutical and nutraceutical fields.

## Methods

### Sponge collection

**Figure 5:**
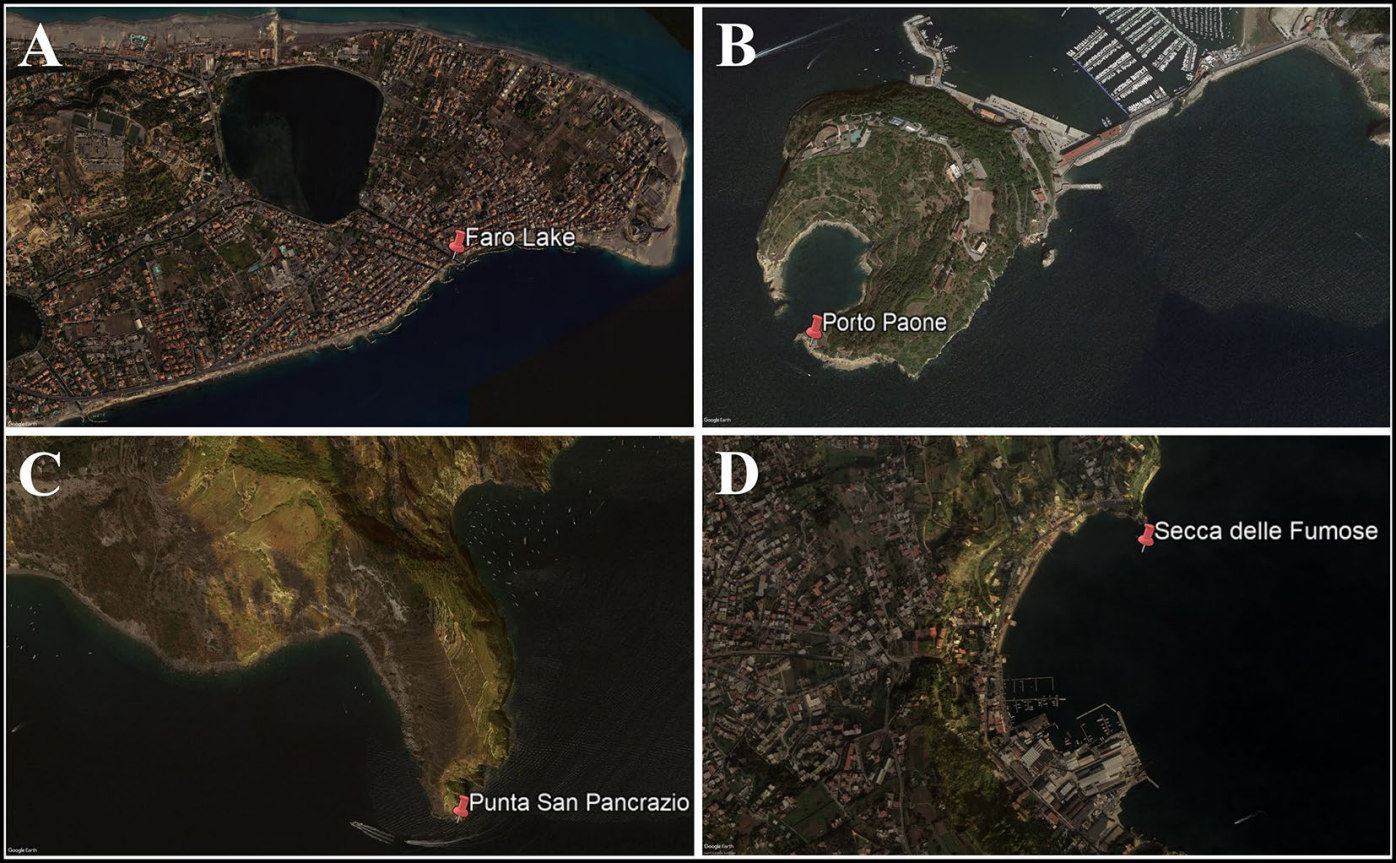
Sampling sites of sponge species collected in Faro Lake (Messina, **A**), Porto Paone (**B**), Punta San Pancrazio (**C**) and Secca delle fumose (**D**). Picture was created by Google Earth Software.

The size of sponge samples ranged from 10 to 20 cm in diameter. Three sponge samples, O.per, S.spi and E.dis were collected at Faro Lake (Messina, Sicily; depth = 2–3 m; 38°16’N, 15°38’E, Fig. 5A; temperature 20 °C, pH 8.25, salinity 31 PSU) in October 2019.

The site Faro Lake (0.263 Km^2^) is the deepest coastal lake in Italy located within the Natural Reserve of “Capo Peloro” (NE Sicily). The Faro Lake is characterized by a funnel-shape profile, with a steep sloping bottom reaching the maximum depth of 29 m in the central area and a wide nearshore shallow waters area. In the deepest part, the Faro Lake shows typical features of a meromictic temperate basin, with an oxygenated mixolimnion (the upper 15 m) and a lower anoxic and sulphidic monimolimnion^88^. Two channels, a northern and a north-eastern, connect the lake to the Tyrrhenian Sea and the Strait of Messina. Salinity ranges are from 26 to 36 PSU, temperature from 10 to 30 °C and pH ranges from 7.0–8.6^26^. Four samples were collected in the Gulf of Naples in September 2019 by scuba diving of Stazione Zoologica Anton Dohrn of Naples (temperature 23.9 °C, pH 8.3, salinity 38 PSU): two samples, reported as T.aur and A.dam, were collected at Porto Paone (depth = 15–17 m; 40°47ʹN, 14°9ʹE, Fig. 5B); A.acu and A.oro were retrieved from Punta San Pancrazio (depth = 7–9 m; 40°42ʹN, 13°57ʹE, Fig. 5C); G.cyd (*Geodia cydonium*) was harvested at Secca delle Fumose, Parco Sommerso di Baia (depth = 20 m; 40°49ʹN, 14°5ʹE, Fig. 5D). All collecting sites were selected on the basis of some data reporting on the great biodiversity and, in some cases, the presence of alien species^26,89,90^.

Collected samples were immediately washed at least three times with filter-sterilized natural seawater. A fragment of each specimen was preserved in 70% ethanol for taxonomic identification; another fragment was then placed into individual sterile tubes and kept in RNA*later*^©^ at − 20 °C used for molecular analysis. Details on sampling were reported in Table 1.

### Morphological analysis of the sponges

For the taxonomic analysis, the spicules of each sponge specimen, spicule complement and skeletal architecture, were examined under light microscopy following published protocols^91,92^. Taxonomic decisions were made according to the classification present in the World Porifera Database (WPD)^14^. The sponge samples were all identified at the species level.

### DNA extraction and PCR amplification

About 10 mg of tissue was used for DNA extraction by *QIAamp® DNA Micro kit* (QIAGEN), according to the manufacturer’s instructions. DNA quantity (ng/μL) was evaluated by a NanoDrop spectrophotometer. PCR reactions were performed on the C1000 Touch Thermal Cycler (BioRad) in a 30 µL reaction mixture final volume including about 50–100 ng of genomic DNA, 6 µL of 5X Buffer GL (GeneSpin Srl, Milan, Italy), 3 µL of dNTPs (2 mM each), 2 µL of each forward and reverse primer (25 pmol/µL), 0.2 µL of Xtra Taq Polymerase (5 U/µL, GeneSpin Srl, Milan, Italy) as follows (for primer sequences, see also Table S13**)**:

i. for 18S and 28S, a denaturation step at 95 °C for 2 min, 35 cycles denaturation step at 95 °C for 1 min, annealing step at 60 °C (A/B^93,94^), 57 °C (C2/D2^95^), 55 °C (18S-AF/18S-BR, NL4F/NL4R^96,97^), 52 °C (18S1/18S2^98^) for 1 min and 72 °C of primer extension for 2 min, a final extension step at 72 °C for 10 min;
ii. ITS primers (RA2/ITS2.2^94,99^), a first denaturation at 95 °C for 2 min, 35 cycles denaturation step at 95 °C for 1 min, annealing step at 67 °C for 1 min and 72 °C of primer extension for 2 min, a final extension step at 72 °C for 10 min;
iii. CO1 primers (dgLCO1490/dgHCO2198^100^), a first denaturation at 94 °C for 3 min, 35 cycles of denaturation at 94 °C for 30 s, annealing at 45 °C for 30 s and primer extension at 72 °C for 1 min.

PCR products were separated on 1.5% agarose gel electrophoresis in TAE buffer (40 mM Tris–acetate, 1 mM EDTA, pH 8.0) using a 100 bp DNA ladder (GeneSpin Srl, Milan, Italy) and purified by *QIAquick Gel Extraction Kit* (Qiagen) according to the manufacturer's instructions. PCR amplicons were then sequenced in both strands through Applied Biosystems (Life Technologies) 3730 Analyzer (48 capillaries). Sequences produced were ~ 650 bases long in average with more than 97.5% accuracy, starting from PCR fragments. Each 18S, 28S and CO1 PCR products were aligned to GenBank using Basic Local Alignment Search Tool (BLAST) and then aligned with highly similar sequence using MultiAlin (http://multalin.toulouse.inra.fr/multalin/, see Figures S1-S7).

### Metagenomic DNA extraction, Illumina MiSeq sequencing and diversity analysis

About 250 mg of tissue were weighted and used for DNA extraction by using *DNeasy® PowerSoil® Pro Kit* (QIAGEN), according to the manufacturer’s instructions. DNA quantity (ng/μL) and quality (A260/280, A260/230) were evaluated by a NanoDrop spectrophotometer. DNA samples were separated by 0.8% agarose gel electrophoresis in TAE buffer (40 mM Tris–acetate, 1 mM EDTA, pH 8.0) to check DNA integrity. 30 ng/μL (final concentration) of sample was used for metataxonomic analysis performed by Bio-Fab Research (Roma, Italy). Illumina adapter overhang nucleotide sequences were added to the gene‐specific primer pairs targeting the V3-V4 region (S-D-Bact-0341-b-S-17/S-D-Bact-0785-a-A-2), with the following sequences:

Forward = 5' TCGTCGGCAGCGTCAGATGTGTATAAGAGACAGCCTACGGGNGGCWGCAG-3',

Reverse = 5'-GTCTCGTGGGCTCGGAGATGTGTATAAGAGACAGGACTACHVGGGTATCTAATCC-3′^101^.

For 16S PCR amplification, 2.5 µL of microbial genomic DNA (5 ng/µL in 10 mM Tris pH 8.5), 5 µL of each Forward and Reverse primer and 12.5 µL of 2 × KAPA HiFi HotStart ReadyMix to a final volume of 25 µL were used. Thermocycler conditions were set as follows: initial denaturation at 95 °C for 3 min, 25 cycles of 95 °C (30 s), 55 °C (30 s), 72 °C (30 s), final extension at 72 °C for 5 min, hold at 4 °C.

After 16S amplification, a PCR clean-up was done to purify the V3-V4 amplicon from free primers and primer dimer species. This step was followed by another limited‐cycle amplification step to add multiplexing indices and Illumina sequencing adapters by using a Nextera XT Index Kit. A second step of clean-up was further performed and then libraries were normalized and pooled by denoising processes (Table S14), and sequenced on Illumina MiSeq Platform with 2 × 300 bp paired-end reads. Taxonomy was assigned using "home made" Naive Bayesian Classifier trained on V3-V4 16S sequences of SILVA 132 database^102^. Frequencies per feature and per sample are shown in Figures S10-S11.

QIIME 2 (Quantitative Insights Into Microbial Ecology) platform^103^ was used for microbiome analysis from raw DNA sequencing data. QIIME 2 analysis workflow was performed by demultiplexing, quality filtering, chimera removal, taxonomic assignment, and diversity analyses (alpha and beta).

Taxonomy BarPlot (Figure S9) was generated through a R version 4.1.1^104^ using Cairo graphics library^105^.

Species diversity was estimated by i. Chao 1 index^106^, which is qualitative species-based method; ii. Shannon^107,108^ and iii. Simpson^109^ indices, which are quantitative species-based measures. All these indices were estimated at three taxa levels (Level 5 = Family, Level 6 = Genus, Level 7 = Species). For alpha and beta diversity, significant differences were assessed by Kruskal–Wallis test and pairwise PERMANOVA analysis, respectively. Moreover, Bray–Curtis and “un-, weighted” UniFrac metrics were used to calculate a distance matrix between each pair of samples, independently from the environmental variables.

## Supplementary Information

Supplementary Information.

## Data Availability

The full dataset of raw data was deposited in the SRA database (Submission ID: SUB8692761; BioProject ID: PRJNA683751).
