# Supplementary material for: Microbial diversity in Mediterranean sponges as revealed by metataxonomic analysis
Source: Sci Rep. 2021 Oct 27;11:21151. doi: 10.1038/s41598-021-00713-9 (PMC8551288; doi:10.1038/s41598-021-00713-9)

Supplementary Material

Table S1. BLAST results from O.per (*Oceanapia* cf. *perforata*), S.spi (*Sarcotragus spinosulus*), E.dis (*Erylus discophorus*), A.oro (*Agelas oroides*) sponges. The primer names, sequence length of amplified fragments, first hits (the species corresponding to those identified by morphological analysis were highlighted in bold), query cover and identity percentages were reported. Correct species were highlighted in bold.

Sample IDs	Region	Primers	Sequence lenght (bp)	BLAST results	Query cover (%)	Identity (%)
O.per	18S	18SAF/18SBR	1500	*Calyx nicaeensis* voucher TAU_ Po.25570	99	99
				Oceanapia isodictyiformis voucher BELUM:Mc6450	97	97
				Oceanapia sp. NIWAKD586	94	88
	18S	A/B	1924	*Calyx nicaeensis* voucher TAU_ Po.25570	99	99
				Oceanapia isodictyiformis voucher BELUM:Mc6450	98	96
				Oceanapia sp. NIWAKD586	92	93
	28S	NL4F/NL4R	1101	*Neopetrosia rosariensis* voucher P10x55	99	98
				Oceanapia sp. NCI101	96	85
	COI	dgLCO1490/dgHCO2198	607	*Calyx nicaeensis* voucher Po.25570	99	95
S.spi	ITS	RA2/ITS2.2	846	***Sarcotragus spinosulus*** voucher Po.25502	99	98
E.dis	18S	18S1/18S2	1696	*Geodia* sp. 0M9H2501-L	99	99
	18S	18SAF/18SBR	1705	*Geodia* sp. 0M9H2501-L	99	99

	28S	NL4F/NL4R	1048	*Penares* cf. *alata* NCI181	99	99
	COI	dgLCO1490/dgHCO2198	462	***Erylus discophorus*** voucher ZMA POR 20420	99	99
A.oro	18S	A/B	1715	***Agelas oroides*** voucher TAU_ Po.25569	99	98
	18S	18SAF/18SBR	1695	***Agelas oroides*** voucher TAU_ Po.25569	100	99
	28S	C2/D2	462	***Agelas oroides*** voucher Po.25569	98	99
	28S	NL4F/NL4R	973	***Agelas oroides*** voucher Po.25569	99	100
	ITS	RA2/ITS2.2	842	*Agelas* sp. QMG317484<br>***Agelas oroides*** voucher ZMA POR 14435	98<br>79	96<br>100

Table S2. BLAST results from T.aur (*Tethya aurantium*), A.dam (*Axinella damicornis*) and A.acu (*Acanthella acuta*) sponges. The primer names, sequence length of amplified fragments, first hits (the species corresponding to those identified by morphological analysis were highlighted in bold), query cover and identity percentages were reported. Correct species were highlighted in bold.

Sample IDs	Region	Primers	Sequence lenght (bp)	BLAST results	Query cover (%)	Identity (%)
T.aur	18S	18S1/18S2	1718	*Tethya* sp.SI06x109<br>***Tethya aurantium***	99<br>91	99<br>99
	18S	A/B	1710	*Tethya* sp. SI06x109<br>***Tethya aurantium***	99<br>90	100<br>99
	28S	C2/D2	449	*Tethya* sp. SI06x109	99	98
	28S	NL4F/NL4R	1034	*Tethya* sp. SI06x109	99	98
	COI	dgLCO1490/dgHCO2198	674	***Tethya aurantium***	95	99
A.dam	18S	A/B	1724	*Hymerhabdia typica*,voucher BELUM:Mc4588<br>***Axinella damicornis*** voucher BELUM:Mc4987	99<br>99	99<br>99
	28S	NL4F/NL4R	997	*Axinella corrugata* voucher HBOIAC	99	99
A.acu	18S	A/B	1681	*Axinella rugosa* voucher BELUM:Mc7456	99	99
	18S	18SAF/18SBR	1710	*Axinella rugosa* voucher BELUM:Mc7456	99	97
	28S	C2/D2	444	***Acanthella acuta*** voucher BELUM:Mc7160	100	96
	COI	dgLCO1490/dgHCO2198	657	***Acanthella acuta*** voucher BELUM<GBR>:Mc7160	98	100

	ITS	RA2/ITS2.2	800	***Acanthella acuta***	100	99

Table S3. Chao1, Shannon and Simpson diversity indices at the family (L5), genus (L6) and species (L7) levels. Sample IDs: O.per= *Oceanapia* cf. *perforata*, S.spi= *Sarcotragus spinosulus*, E.dis= *Erylus discophorus*, A.oro= *Agelas oroides*, T.aur= *Tethya aurantium*, A.dam= *Axinella damicornis*, A.acu= *Acanthella acuta* and G.cyd= *Geodia cydonium*.

	**L5**				**L6**				**L7**			
**Sample ID**	**Observed features**	**Chao1**	**Shannon**	**Simpson**	**Observed features**	**Chao1**	**Shannon**	**Simpson**	**Observed features**	**Chao1**	**Shannon**	**Simpson**
O.per	43	43.17	2.63	0.67	53	53.11	2.7	0.67	59	59.1	2.81	0.67
S.spi	64	64	4.69	0.94	69	69	4.73	0.94	78	78	4.93	0.94
E.dis	61	61	2.55	0.71	76	76	2.58	0.71	83	83	2.59	0.71
A.oro	137	137	3.64	0.79	185	185.18	3.84	0.8	220	220.28	3.92	0.8
T.aur	69	69	5.11	0.96	73	73	5.15	0.96	77	77	5.21	0.96
A.dam	63	63	4.74	0.94	70	70	4.82	0.95	81	81.33	4.98	0.95
A.acu	70	70	4.78	0.93	74	74	4.83	0.93	81	81	5.22	0.96
G.cyd	72	70.5	4.6	0.94	82	82.5	4.69	0.94	102	102.5	4.94	0.95

Table S4. ASVs (198) from *Agelas oroides* with percentage of confidence ≥ 75%.

Feature ID	Number of sequences	Confidence	Taxon
65a27ba6edc1d275d2ecafd787a21198	1459	1.00	D_0__Bacteria
d5d1332c5fb5352de80441aabefe6bcf	891	1.00	D_0__Bacteria;D_1__Chloroflexi;D_2__Dehalococcoidia;D_3__SAR202 clade
f606f45ada87bd37234703bf9c5e15dc	774	0.96	D_0__Bacteria;D_1__Chloroflexi;D_2__Anaerolineae;D_3__Caldilineales;D_4__Caldilineaceae;D_5__uncultured;D_6__uncultured Caldilinea sp.
772106d51e3b344d774582c20fd57531	738	0.95	D_0__Bacteria;D_1__Chloroflexi;D_2__Dehalococcoidia;D_3__SAR202 clade;D_4__uncultured Chloroflexi bacterium;D_5__uncultured Chloroflexi bacterium;D_6__uncultured Chloroflexi bacterium
0424feb01196f37963c60430271e958b	717	0.82	D_0__Bacteria;D_1__Chloroflexi;D_2__Dehalococcoidia;D_3__SAR202 clade;D_4__uncultured Chloroflexi bacterium;D_5__uncultured Chloroflexi bacterium;D_6__uncultured Chloroflexi bacterium
0976440511c93b42f8f54ca0366d05b9	382	1.00	D_0__Bacteria;D_1__Proteobacteria;D_2__Gammaproteobacteria;D_3__KI89A clade;D_4__uncultured bacterium;D_5__uncultured bacterium;D_6__uncultured bacterium
e63bcbf9131484f4976d299237f70005	340	0.94	D_0__Bacteria;D_1__Dadabacteria;D_2__Dadabacteriia;D_3__Dadabacteriales;D_4__uncultured delta proteobacterium;D_5__uncultured delta proteobacterium;D_6__uncultured delta proteobacterium
0071d628da246724b5e7848e9f38ef75	298	1.00	D_0__Bacteria;D_1__Proteobacteria;D_2__Deltaproteobacteria;D_3__Oligoflexales;D_4__Oligoflexaceae;D_5__uncultured;D_6__uncultured delta proteobacterium
4319fc305ab85c1a8c2f4eae07478cc9	297	1.00	D_0__Bacteria;D_1__Proteobacteria;D_2__Gammaproteobacteria;D_3__KI89A clade;D_4__uncultured bacterium;D_5__uncultured bacterium;D_6__uncultured bacterium
cab7e904b5f7cba2f40444d58111f19a	292	1.00	D_0__Bacteria;D_1__Chloroflexi;D_2__Anaerolineae;D_3__SBR1031;D_4__A4b
b8a54e96900c1bb0e64b1e7f9444ab0a	275	0.97	D_0__Bacteria;D_1__Proteobacteria;D_2__Deltaproteobacteria;D_3__Myxococcales;D_4__bacteriap25;D_5__uncultured bacterium;D_6__uncultured bacterium

86e239ee287c04ffb80f7b368ae95134	208	1.00	D_0__Bacteria;D_1__Proteobacteria;D_2__Gammaproteobacteria;D_3__Nitrosococcales;D_4__Nitrosococcaceae;D_5__AqS1
2968a831d1100a78d96b050b315f4717	204	0.99	D_0__Bacteria;D_1__Poribacteria;D_2__uncultured Clostridium sp.;D_3__uncultured Clostridium sp.;D_4__uncultured Clostridium sp.;D_5__uncultured Clostridium sp.;D_6__uncultured Clostridium sp.
de77cf2ed18648bab0a960fd8c03acf6	197	0.99	D_0__Bacteria;D_1__Chloroflexi;D_2__Anaerolineae;D_3__Caldilineales;D_4__Caldilineaceae;D_5__uncultured;D_6__uncultured Chloroflexus sp.
d0a1bd57ffd7d01b68b4708fa0dd5559	189	1.00	D_0__Bacteria;D_1__Poribacteria;D_2__uncultured Clostridium sp.;D_3__uncultured Clostridium sp.;D_4__uncultured Clostridium sp.;D_5__uncultured Clostridium sp.;D_6__uncultured Clostridium sp.
7a0714beeb41da0c19c9efb244a8e1b5	171	1.00	D_0__Bacteria;D_1__Chloroflexi;D_2__Dehalococcoidia;D_3__SAR202 clade
1b4586d811edf9d13cac333810e62adf	159	0.86	D_0__Bacteria;D_1__Proteobacteria;D_2__Deltaproteobacteria;D_3__Oligoflexales;D_4__Oligoflexaceae;D_5__uncultured;D_6__uncultured delta proteobacterium
cac6f7dfa99774379fdfa58642966c85	147	0.90	D_0__Bacteria;D_1__Proteobacteria;D_2__Gammaproteobacteria;D_3__KI89A clade;D_4__uncultured organism;D_5__uncultured organism;D_6__uncultured organism
9e8067b888e0102fa84d94cd401c0e90	140	0.98	D_0__Bacteria;D_1__Proteobacteria;D_2__Gammaproteobacteria;D_3__Nitrosococcales;D_4__Nitrosococcaceae;D_5__AqS1;D_6__uncultured marine bacterium
3c42a7595042d9ee6f9cd4473f09e874	133	1.00	D_0__Bacteria;D_1__Proteobacteria;D_2__Gammaproteobacteria;D_3__KI89A clade;D_4__uncultured bacterium;D_5__uncultured bacterium;D_6__uncultured bacterium
1c2a67b199b9408a08a06f5c3fd9ed36	128	1.00	D_0__Bacteria;D_1__Proteobacteria;D_2__Deltaproteobacteria;D_3__Oligoflexales;D_4__Oligoflexaceae;D_5__uncultured
a7138433b0f9e3bb5937e88a4a4693d4	126	1.00	D_0__Bacteria;D_1__Proteobacteria;D_2__Gammaproteobacteria;D_3__KI89A clade;D_4__uncultured bacterium;D_5__uncultured bacterium;D_6__uncultured bacterium
1fd967a3991584fed6d789871fdc203c	125	0.84	D_0__Archaea;D_1__Thaumarchaeota;D_2__Nitrososphaeria;D_3__Nitrosopumilales;D_4__Nitrosopumilaceae;D_5__Cenarchaeum
4445ebd89e3eac5613fd8e88cb57cc07	111	0.92	D_0__Bacteria;D_1__Proteobacteria;D_2__Deltaproteobacteria;D_3__Myxococcales;D_4__bacteriap25;D_5__uncultured bacterium;D_6__uncultured bacterium

e885326a18addf9f1c2ddbd93abfc8b4	101	0.95	D_0__Bacteria;D_1__Proteobacteria;D_2__Gammaproteobacteria;D_3__Nitrosococcales;D_4__Nitrosococcaceae;D_5__FS142-36B-02
2ceaabe00666c9084e7ae98439e9df21	99	0.98	D_0__Bacteria;D_1__Proteobacteria;D_2__Gammaproteobacteria;D_3__Nitrosococcales;D_4__Nitrosococcaceae;D_5__AqS1
c8f499ad1129269470f4b1ea8dd16df3	99	1.00	D_0__Bacteria;D_1__Proteobacteria;D_2__Gammaproteobacteria;D_3__Nitrosococcales;D_4__Nitrosococcaceae;D_5__AqS1
7f1633e2b7d0c1ae954d4e163d17be06	97	1.00	D_0__Bacteria;D_1__Proteobacteria;D_2__Gammaproteobacteria;D_3__KI89A clade;D_4__uncultured bacterium;D_5__uncultured bacterium;D_6__uncultured bacterium
018906a080b5d33b91201137d6ea419f	94	0.79	D_0__Bacteria;D_1__Nitrospirae;D_2__Nitrospira;D_3__Nitrospirales;D_4__Nitrospiraceae;D_5__Nitrospira;Ambiguous_taxa
f48bb3d53686512badde400ed0307681	93	1.00	D_0__Bacteria;D_1__Acidobacteria;D_2__Subgroup 9
447b20386158433b6ea24baea55cb9d7	91	0.84	D_0__Bacteria;D_1__Chloroflexi;D_2__TK17;D_3__uncultured bacterium;D_4__uncultured bacterium;D_5__uncultured bacterium;D_6__uncultured bacterium
41fe2755d5d6d814972a3336f7e69311	90	1.00	D_0__Bacteria;D_1__Gemmatimonadetes;D_2__PAUC43f marine benthic group
e550faa869b4a4dccac634f9c06ad5de	88	0.89	D_0__Bacteria;D_1__Proteobacteria;D_2__Deltaproteobacteria;D_3__Myxococcales;D_4__bacteriap25;D_5__uncultured bacterium;D_6__uncultured bacterium
e5c8a4745c1145908335ec6bf1f5a6da	85	0.97	D_0__Bacteria;D_1__PAUC34f;D_2__uncultured Deferribacteres bacterium;D_3__uncultured Deferribacteres bacterium;D_4__uncultured Deferribacteres bacterium;D_5__uncultured Deferribacteres bacterium;D_6__uncultured Deferribacteres bacterium
5aa6c7dd80754529e1f404bd72d236c5	84	1.00	D_0__Bacteria;D_1__Actinobacteria;D_2__Acidimicrobiia;D_3__Microtrichales;D_4__Microtrichaceae;D_5__Sva0996 marine group;D_6__uncultured marine bacterium
8e5da6ea87b1d863052acecd59bacb92	82	0.75	D_0__Bacteria;D_1__Proteobacteria;D_2__Deltaproteobacteria;D_3__Myxococcales;D_4__bacteriap25
2798d3bce0f8df68c073905f415c6ddc	77	1.00	D_0__Bacteria;D_1__Acidobacteria;D_2__Subgroup 6
dbefe1f8a0e8405a17b102d0468c8106	77	1.00	D_0__Bacteria;D_1__Chloroflexi;D_2__Dehalococcoidia;D_3__SAR202 clade

09828005445a76fbf003023a27a3079a	76	1.00	D_0__Bacteria;D_1__Proteobacteria;D_2__Gammaproteobacteria;D_3__HOC36;Ambiguous_taxa;Ambiguous_taxa;Ambiguous_taxa
8951d49821c2aafefe5f4d03752f6420	74	0.94	D_0__Bacteria;D_1__Chloroflexi;D_2__Dehalococcoidia;D_3__SAR202 clade;D_4__uncultured Chloroflexus sp.;D_5__uncultured Chloroflexus sp.;D_6__uncultured Chloroflexus sp.
6c091a93bc941d195d4da4bb2d152dc0	74	0.89	D_0__Bacteria;D_1__Chloroflexi;D_2__Dehalococcoidia;D_3__SAR202 clade;D_4__uncultured bacterium;D_5__uncultured bacterium;D_6__uncultured bacterium
c81295bf6a818ebb416f98ef9293b9dd	70	1.00	D_0__Bacteria;D_1__Proteobacteria;D_2__Gammaproteobacteria;D_3__KI89A clade;D_4__uncultured bacterium;D_5__uncultured bacterium;D_6__uncultured bacterium
44a1b849a69bc961187d88b40fe61522	70	1.00	D_0__Bacteria;D_1__Chloroflexi;D_2__Anaerolineae;D_3__SBR1031;D_4__A4b
d001da55fd721b9ea319c83fe1164eee	68	1.00	D_0__Bacteria;D_1__Chloroflexi;D_2__Dehalococcoidia;D_3__SAR202 clade
47fbbb9410f1395149f4ff6528ab7a50	65	1.00	D_0__Bacteria;D_1__Acidobacteria;D_2__Subgroup 6
92416d83123b5426bcec5bbf922e9dfc	63	0.96	D_0__Bacteria;D_1__Proteobacteria;D_2__Alphaproteobacteria;D_3__uncultured
017fa3027ae518d5eec091f58d621496	60	0.99	D_0__Bacteria;D_1__Actinobacteria;D_2__Acidimicrobiia;D_3__Actinomarinales;D_4__uncultured;D_5__uncultured actinobacterium;D_6__uncultured actinobacterium
eea2e0daa674a37498cf59dd9e50d113	55	0.91	D_0__Bacteria;D_1__Chloroflexi;D_2__Anaerolineae;D_3__SBR1031;D_4__A4b;Ambiguous_taxa;Ambiguous_taxa
8a2f2226a9bd42b75b233f02fb4d9142	52	1.00	D_0__Bacteria;D_1__Actinobacteria;D_2__Acidimicrobiia;D_3__Microtrichales;D_4__Microtrichaceae;D_5__Sva0996 marine group
80911654d839b4f762a95f137abaaccd	50	1.00	D_0__Bacteria;D_1__Poribacteria
516232c17e43dc067b05d188e3d326bd	48	1.00	D_0__Bacteria;D_1__Spirochaetes;D_2__Spirochaetia;D_3__Spirochaetales;D_4__Spirochaetaceae;D_5__Spirochaeta 2;D_6__uncultured Spirochaetales bacterium
54553797028b61123b1d587b2c42852a	45	1.00	D_0__Bacteria;D_1__Proteobacteria;D_2__Deltaproteobacteria;D_3__Bdellovibrionales;D_4__Bdellovibrionaceae;D_5__Bdellovibrio

5d39543ba89941af08d6afb097f38617	45	1.00	D_0__Bacteria;D_1__Proteobacteria;D_2__Gammaproteobacteria;D_3__Nitrosococcales;D_4__Nitrosococcaceae;D_5__AqS1
a621d197330cc679888287edac477446	45	0.89	D_0__Bacteria;D_1__Nitrospirae;D_2__Nitrospira;D_3__Nitrospirales;D_4__Nitrospiraceae;D_5__Nitrospira;Ambiguous_taxa
8acded78244b8b5d74172fd0b2d2fd57	45	1.00	D_0__Bacteria;D_1__Chloroflexi;D_2__Dehalococcoidia;D_3__SAR202 clade;D_4__uncultured deep-sea bacterium;D_5__uncultured deep-sea bacterium;D_6__uncultured deep-sea bacterium
441b0f384b3d7ef1741ffa4b862b6624	43	0.83	D_0__Bacteria;D_1__Actinobacteria;D_2__Acidimicrobiia;D_3__Microtrichales;D_4__Microtrichaceae;D_5__Sva0996 marine group;D_6__uncultured actinobacterium
a7a8d5fa58a688312efc5d582748c3b1	40	1.00	D_0__Bacteria;D_1__Chloroflexi;D_2__Dehalococcoidia;D_3__SAR202 clade
6781398f398690e754802767faba0717	40	1.00	D_0__Bacteria;D_1__Chloroflexi;D_2__Dehalococcoidia;D_3__SAR202 clade
d732727d68625267e77ff170131c8e1d	38	1.00	D_0__Bacteria;D_1__Proteobacteria;D_2__Gammaproteobacteria;D_3__KI89A clade
690884e72c29d1be446338f022f6a3b6	38	0.93	D_0__Bacteria;D_1__Proteobacteria;D_2__Deltaproteobacteria;D_3__Myxococcales;D_4__bacteriap25;D_5__uncultured bacterium;D_6__uncultured bacterium
21206b4dedf1c295c3e558db27f34167	37	0.87	D_0__Bacteria;D_1__Proteobacteria;D_2__Gammaproteobacteria;D_3__Nitrosococcales;D_4__Nitrosococcaceae;D_5__AqS1;D_6__uncultured bacterium
09a22a825508cd6b8151db8d14cb4980	37	0.97	D_0__Bacteria;D_1__Proteobacteria;D_2__Gammaproteobacteria;D_3__JTB23;D_4__uncultured bacterium;D_5__uncultured bacterium;D_6__uncultured bacterium
9ef03e986585cc0368af5e9dd7a4be87	37	1.00	D_0__Bacteria;D_1__Proteobacteria;D_2__Deltaproteobacteria;D_3__Bdellovibrionales;D_4__Bdellovibrionaceae;D_5__Bdellovibrio;D_6__uncultured delta proteobacterium
f45468a2fb22d6101d596b223e1ba4ab	36	0.80	D_0__Bacteria;D_1__Proteobacteria;D_2__Alphaproteobacteria;D_3__SAR11 clade;D_4__Clade I;D_5__Clade Ia
c2ef3a4de4437c03e66036dca8d4c1b2	34	0.92	D_0__Bacteria;D_1__Proteobacteria;D_2__Deltaproteobacteria;D_3__Bdellovibrionales;D_4__Bdellovibrionaceae;D_5__Bdellovibrio;D_6__uncultured delta proteobacterium
61263ef30965fbcb5f8f13ee212bbfa3	33	0.95	D_0__Bacteria;D_1__Chloroflexi;D_2__Dehalococcoidia;D_3__SAR202 clade;D_4__uncultured Chloroflexi bacterium;D_5__uncultured Chloroflexi bacterium;D_6__uncultured Chloroflexi bacterium

9e5903c2287ddbf33a1264544766187b	31	0.95	D_0__Bacteria;D_1__Proteobacteria;D_2__Alphaproteobacteria;D_3__Sneathiellales;D_4__Sneathiellaceae;D_5__AT-s3-44;D_6__uncultured bacterium
dbad9eb966a635c309a85f03dbd3da5e	30	0.78	D_0__Bacteria;D_1__Spirochaetes;D_2__Spirochaetia;D_3__Spirochaetales;D_4__Spirochaetaceae;D_5__Spirochaeta 2;Ambiguous_taxa
7bc6c597f823d1dc17f64977afdf2992	30	0.99	D_0__Bacteria;D_1__Poribacteria;D_2__uncultured bacterium;D_3__uncultured bacterium;D_4__uncultured bacterium;D_5__uncultured bacterium;D_6__uncultured bacterium
b053eeecb8f1af123e041c675da72246	30	0.99	D_0__Bacteria;D_1__Proteobacteria;D_2__Deltaproteobacteria;D_3__Bdellovibrionales;D_4__Bdellovibrionaceae;D_5__Bdellovibrio;D_6__uncultured delta proteobacterium
308b59475810f3c5f78196786de9a047	29	1.00	D_0__Bacteria;D_1__Proteobacteria;D_2__Deltaproteobacteria;D_3__Bdellovibrionales;D_4__Bdellovibrionaceae;D_5__Bdellovibrio;D_6__uncultured delta proteobacterium
e2244d739d233651197d4a4da9452c34	29	0.98	D_0__Bacteria;D_1__Proteobacteria;D_2__Alphaproteobacteria;D_3__Puniceispirillales;D_4__EF100-94H03;D_5__uncultured bacterium;D_6__uncultured bacterium
61147b3def6a9e50de57d3fbd97d192c	29	0.78	D_0__Bacteria;D_1__Nitrospirae;D_2__Nitrospira;D_3__Nitrospirales;D_4__Nitrospiraceae;D_5__Nitrospira;Ambiguous_taxa
765b7fe0d7a35671220871fb767cc907	28	1.00	D_0__Bacteria;D_1__Proteobacteria;D_2__Deltaproteobacteria;D_3__Bdellovibrionales;D_4__Bdellovibrionaceae;D_5__Bdellovibrio
3de1f41fdb8623ee1535489c1c5a05cd	28	0.99	D_0__Bacteria;D_1__Proteobacteria;D_2__Gammaproteobacteria;D_3__EPR3968-O8a-Bc78;Ambiguous_taxa;Ambiguous_taxa;Ambiguous_taxa
1fa7f0c83a98c222f595545599a18e96	27	1.00	D_0__Bacteria;D_1__Proteobacteria;D_2__Gammaproteobacteria;D_3__KI89A clade;D_4__uncultured bacterium;D_5__uncultured bacterium;D_6__uncultured bacterium
705313b4aadc737a0b2919caf775e86e	26	1.00	D_0__Bacteria;D_1__Proteobacteria;D_2__Deltaproteobacteria;D_3__Bdellovibrionales;D_4__Bdellovibrionaceae;D_5__Bdellovibrio;D_6__uncultured delta proteobacterium
6ef7c8c05b9fbebdc2e64443eaaf1d3d	26	0.97	D_0__Bacteria;D_1__Proteobacteria;D_2__Gammaproteobacteria;D_3__JTB23;D_4__uncultured bacterium;D_5__uncultured bacterium;D_6__uncultured bacterium
d2e9743c97fc8198bec4edeb5bdd2952	26	1.00	D_0__Bacteria;D_1__Proteobacteria;D_2__Gammaproteobacteria;D_3__KI89A clade

995809a88e3e73fdf2e95b1ecf8504db	26	0.88	D_0__Bacteria;D_1__Proteobacteria;D_2__Deltaproteobacteria;D_3__Myxococcales;D_4__bacteriap25;D_5__uncultured bacterium;D_6__uncultured bacterium
1d510d72b9bea7b36e0da1a9fb78165f	25	0.99	D_0__Bacteria;D_1__Proteobacteria;D_2__Deltaproteobacteria;D_3__Myxococcales;D_4__bacteriap25;D_5__uncultured bacterium;D_6__uncultured bacterium
373ca6992956b0450d757ad1da253229	25	0.97	D_0__Bacteria;D_1__Proteobacteria;D_2__Gammaproteobacteria;D_3__JTB23;D_4__uncultured bacterium;D_5__uncultured bacterium;D_6__uncultured bacterium
43c003c18ff0a8f1ed4f03dafa6316e9	25	1.00	D_0__Bacteria;D_1__Proteobacteria;D_2__Gammaproteobacteria
b12aae2a91cb407bf34ef2b432a66420	25	1.00	D_0__Bacteria;D_1__Chloroflexi;D_2__Dehalococcoidia;D_3__SAR202 clade;D_4__uncultured deep-sea bacterium;D_5__uncultured deep-sea bacterium;D_6__uncultured deep-sea bacterium
c2529e62f189851eed36f54b90188122	24	1.00	D_0__Bacteria;D_1__Dadabacteria;D_2__Dadabacteriia;D_3__Dadabacteriales;Ambiguous_taxa;Ambiguous_taxa;Ambiguous_taxa
704d199540d8c8bb592a678206bceb47	24	1.00	D_0__Bacteria;D_1__Proteobacteria;D_2__Deltaproteobacteria;D_3__Bdellovibrionales;D_4__Bdellovibrionaceae;D_5__Bdellovibrio;D_6__uncultured delta proteobacterium
992e0d6d230de4a4792e5f8e45f2de86	23	1.00	D_0__Bacteria;D_1__Proteobacteria;D_2__Deltaproteobacteria;D_3__Bdellovibrionales;D_4__Bdellovibrionaceae;D_5__Bdellovibrio;D_6__uncultured delta proteobacterium
a3498491f0978f24114414dcb7511fc7	23	0.91	D_0__Bacteria;D_1__Chloroflexi;D_2__Dehalococcoidia;D_3__SAR202 clade;Ambiguous_taxa;Ambiguous_taxa;Ambiguous_taxa
33c3533950026b9fcbbad0c709c4f27e	22	0.78	D_0__Bacteria;D_1__Proteobacteria;D_2__Alphaproteobacteria;D_3__Rhodospirillales;D_4__Magnetospiraceae;D_5__uncultured
2fa0f04a49fb915b4b9797102ff332dd	22	1.00	D_0__Bacteria;D_1__Gemmatimonadetes;D_2__BD2-11 terrestrial group
5841aee16fecffbc39d8f537b225702a	21	1.00	D_0__Bacteria;D_1__Proteobacteria;D_2__Deltaproteobacteria;D_3__Bdellovibrionales;D_4__Bacteriovoracaceae
b34288556bafc50acae2ebbffd145fc7	21	1.00	D_0__Bacteria;D_1__Proteobacteria;D_2__Deltaproteobacteria;D_3__Bdellovibrionales;D_4__Bacteriovoracaceae
0e66aab4a0379f6fb521fa579fd257e1	21	1.00	D_0__Bacteria;D_1__Chloroflexi;D_2__Dehalococcoidia;D_3__SAR202 clade

d4d097e66b39fa6c885b9c3568c779a5	20	0.85	D_0__Archaea;D_1__Thaumarchaeota;D_2__Nitrososphaeria;D_3__Nitrosopumilales;D_4__Nitrosopumilaceae;D_5__Cenarchaeum
ac738887fb4e5853063e2afc07f95d0c	20	1.00	D_0__Bacteria;D_1__Chloroflexi;D_2__TK30;D_3__uncultured bacterium;D_4__uncultured bacterium;D_5__uncultured bacterium;D_6__uncultured bacterium
cbb100c3a96d485a8375d9910c137591	19	0.90	D_0__Bacteria;D_1__Actinobacteria;D_2__Acidimicrobiia;D_3__Microtrichales;D_4__Microtrichaceae;D_5__Sva0996 marine group;D_6__uncultured bacterium
016f879f37caf3f6e271fa90114ca82c	17	1.00	D_0__Bacteria;D_1__Proteobacteria;D_2__Deltaproteobacteria;D_3__Bdellovibrionales;D_4__Bdellovibrionaceae;D_5__Bdellovibrio
159f9ab094e6f0d6cc67f7d4086316a3	17	0.89	D_0__Bacteria;D_1__Proteobacteria;D_2__Gammaproteobacteria;D_3__Nitrosococcales;D_4__Nitrosococcaceae;D_5__AqS1;D_6__uncultured marine bacterium
2ebf1203587733d94789706fb41cc3ff	17	0.81	D_0__Bacteria;D_1__Chloroflexi;D_2__Dehalococcoidia;D_3__SAR202 clade;D_4__uncultured Chloroflexi bacterium;D_5__uncultured Chloroflexi bacterium;D_6__uncultured Chloroflexi bacterium
c36792501371d518441d9d6ec33638bd	17	0.76	D_0__Bacteria;D_1__Proteobacteria;D_2__Alphaproteobacteria;D_3__Sneathiellales;D_4__Sneathiellaceae;D_5__AT-s3-44;D_6__uncultured bacterium
16c909a2179e793ab502e2c15357217b	16	0.90	D_0__Bacteria;D_1__Proteobacteria;D_2__Deltaproteobacteria;D_3__Bdellovibrionales;D_4__Bdellovibrionaceae;D_5__Bdellovibrio;D_6__uncultured bacterium
90f352a0674fc8980132633cf7437b61	16	1.00	D_0__Bacteria;D_1__Proteobacteria;D_2__Deltaproteobacteria;D_3__Bdellovibrionales;D_4__Bdellovibrionaceae;D_5__Bdellovibrio;D_6__uncultured delta proteobacterium
2bc597b06dabd648132bd735e7b4c409	15	0.95	D_0__Bacteria;D_1__Acidobacteria;D_2__Acidobacteriia;D_3__Solibacterales;D_4__Solibacteraceae (Subgroup 3);D_5__PAUC26f;D_6__uncultured bacterium
2fd1057473cc51c4287670399335854e	15	0.76	D_0__Bacteria;D_1__Chloroflexi;D_2__Dehalococcoidia;D_3__SAR202 clade;D_4__uncultured deep-sea bacterium;D_5__uncultured deep-sea bacterium;D_6__uncultured deep-sea bacterium
31ffdc7521fa9e1b686901e464fe462d	14	1.00	D_0__Bacteria;D_1__Proteobacteria;D_2__Deltaproteobacteria;D_3__Bdellovibrionales;D_4__Bdellovibrionaceae;D_5__Bdellovibrio;D_6__uncultured delta proteobacterium
6c595a6bc561471062f85c054f2281e3	14	1.00	D_0__Bacteria;D_1__Proteobacteria;D_2__Deltaproteobacteria;D_3__Bdellovibrionales;D_4__Bdellovibrionaceae;D_5__Bdellovibrio;D_6__uncultured delta proteobacterium

69390477f0b882ec9fa823dc1c5fefbe	14	1.00	D_0__Bacteria;D_1__Proteobacteria;D_2__Gammaproteobacteria;D_3__HOC36
e881f1300ed3d1b6ae05845f85758955	14	0.80	D_0__Bacteria;D_1__Proteobacteria;D_2__Deltaproteobacteria;D_3__Bdellovibrionales;D_4__Bdellovibrionaceae;D_5__Bdellovibrio;D_6__uncultured delta proteobacterium
cf3f11164b4c8f9399d9ae487b1f96a6	13	1.00	D_0__Bacteria;D_1__Proteobacteria;D_2__Deltaproteobacteria;D_3__Bdellovibrionales;D_4__Bdellovibrionaceae;D_5__Bdellovibrio;D_6__uncultured delta proteobacterium
48625dccd44972d02ff1619e9dc8d1fd	13	0.95	D_0__Bacteria;D_1__PAUC34f;Ambiguous_taxa;Ambiguous_taxa;Ambiguous_taxa;Ambiguous_taxa;Ambiguous_taxa
6f6e79b40c2205294a881fedd0bb1da6	12	0.87	D_0__Bacteria;D_1__Proteobacteria;D_2__Gammaproteobacteria;D_3__SAR86 clade;D_4__bacterium WHC4-9;D_5__bacterium WHC4-9;D_6__bacterium WHC4-9
bad1bec5687203184dabefe8a14a8ef3	12	1.00	D_0__Bacteria;D_1__Proteobacteria;D_2__Deltaproteobacteria;D_3__Bdellovibrionales;D_4__Bdellovibrionaceae;D_5__Bdellovibrio;D_6__uncultured delta proteobacterium
1e4acb44fcf8f2a0e2d44b6c4f53afd7	12	1.00	D_0__Bacteria;D_1__Proteobacteria;D_2__Deltaproteobacteria;D_3__Bdellovibrionales;D_4__Bdellovibrionaceae;D_5__Bdellovibrio
f7a861d46ed2068ce6538e8d4852a037	12	1.00	D_0__Bacteria;D_1__Proteobacteria;D_2__Deltaproteobacteria;D_3__Bdellovibrionales;D_4__Bdellovibrionaceae;D_5__Bdellovibrio
e0de5f2541a69a98aec31d241d4170e3	12	1.00	D_0__Bacteria;D_1__Proteobacteria;D_2__Deltaproteobacteria;D_3__Bdellovibrionales;D_4__Bdellovibrionaceae;D_5__Bdellovibrio;D_6__uncultured delta proteobacterium
6d7938bac915f75012fbdc6eb31df000	12	1.00	D_0__Bacteria;D_1__Proteobacteria;D_2__Deltaproteobacteria;D_3__Bdellovibrionales;D_4__Bdellovibrionaceae;D_5__Bdellovibrio
2fc5314f45a3521c2dd051fa9d98c9a1	11	0.96	D_0__Bacteria;D_1__Proteobacteria;D_2__Deltaproteobacteria;D_3__Bdellovibrionales;D_4__Bdellovibrionaceae;D_5__Bdellovibrio;D_6__uncultured delta proteobacterium
80e847ddc486cade32b0852599496017	11	1.00	D_0__Bacteria;D_1__Proteobacteria;D_2__Deltaproteobacteria;D_3__Bdellovibrionales;D_4__Bdellovibrionaceae;D_5__Bdellovibrio;D_6__uncultured delta proteobacterium
ef233d1487aa99ea9273a5d8c18d2a1e	11	1.00	D_0__Archaea;D_1__Nanoarchaeaeota;D_2__Woesearchaeia

15c1aff5154b38759b01871a53cb057d	11	0.96	D_0__Bacteria;D_1__Proteobacteria;D_2__Deltaproteobacteria;D_3__Bdellovibrionales;D_4__Bdellovibrionaceae;D_5__Bdellovibrio;D_6__Vibrio sp. Gp-3-1.3.1-voll
b9bd8d8cd056814fb398f507d26e2b62	11	0.99	D_0__Bacteria;D_1__Spirochaetes;D_2__Leptospirae;D_3__Leptospirales;D_4__Leptospiraceae
22df131d2ba7d92f6c1c402e8861e782	11	1.00	D_0__Bacteria;D_1__Proteobacteria;D_2__Deltaproteobacteria;D_3__Bdellovibrionales;D_4__Bdellovibrionaceae;D_5__Bdellovibrio;D_6__uncultured Bdellovibrio sp.
77e9cd3039eee3d14b9d60b092e6dd78	10	1.00	D_0__Bacteria;D_1__Proteobacteria;D_2__Deltaproteobacteria;D_3__Bdellovibrionales;D_4__Bdellovibrionaceae;D_5__Bdellovibrio;D_6__uncultured delta proteobacterium
a194009c5bc7b560876f3e7e1e7ff60e	10	0.75	D_0__Bacteria;D_1__Patescibacteria;D_2__Parcubacteria;D_3__Candidatus Spechtbacteria;D_4__uncultured bacterium;D_5__uncultured bacterium;D_6__uncultured bacterium
f2513337e3b190014e440f4a9de26641	10	0.79	D_0__Bacteria;D_1__Proteobacteria;D_2__Alphaproteobacteria;D_3__Sneathiellales;D_4__Sneathiellaceae;D_5__AT-s3-44;D_6__uncultured bacterium
2a5aac497eed406206cfefa0169f3cce	10	1.00	D_0__Bacteria;D_1__Chloroflexi;D_2__Dehalococcoidia;D_3__SAR202 clade
762868e81785147c729dc2e819d2e3fc	10	1.00	D_0__Bacteria;D_1__Proteobacteria;D_2__Deltaproteobacteria;D_3__Bdellovibrionales;D_4__Bdellovibrionaceae;D_5__Bdellovibrio;D_6__uncultured delta proteobacterium
12ad9235b3639a019c081d69e7acd0f3	10	1.00	D_0__Bacteria;D_1__Proteobacteria;D_2__Deltaproteobacteria;D_3__Bdellovibrionales;D_4__Bdellovibrionaceae;D_5__Bdellovibrio;D_6__uncultured delta proteobacterium
3f040d8d207f6171ff820ff50d01dd4e	9	0.76	D_0__Bacteria;D_1__Proteobacteria;D_2__Gammaproteobacteria;D_3__KI89A clade;D_4__uncultured bacterium;D_5__uncultured bacterium;D_6__uncultured bacterium
68abfb66e7f355cb8941f9c3cb7564c5	9	0.84	D_0__Bacteria;D_1__Entotheonellaeota;D_2__Entotheonellia;D_3__Entotheonellales;D_4__Entotheonellaceae;D_5__uncultured delta proteobacterium;D_6__uncultured delta proteobacterium
63364a66fc96658f7134282fdc114eff	9	1.00	D_0__Bacteria;D_1__Chloroflexi;D_2__Dehalococcoidia;D_3__SAR202 clade
fe66a81f3bd25d641495156627a37ca1	9	1.00	D_0__Bacteria;D_1__Proteobacteria;D_2__Gammaproteobacteria;D_3__KI89A clade;D_4__uncultured bacterium;D_5__uncultured bacterium;D_6__uncultured bacterium

82e2fcbe556f2d457c561098cb4eeb27	8	0.85	D_0__Bacteria;D_1__Proteobacteria;D_2__Gammaproteobacteria;D_3__Oceanospirillales;D_4__Endozoicomonadaceae;D_5__Endozoicomonas;D_6__Endozoicomonas sp. Ez302
6a22aa2d30a07158dfa8ca0dc9cf9ba3	8	1.00	D_0__Bacteria;D_1__Proteobacteria;D_2__Alphaproteobacteria;D_3__Rhodobacterales;D_4__Rhodobacteraceae
0bf7de0ba288ad6fc1b2104bc3608b5c	8	1.00	D_0__Bacteria;D_1__Lentisphaerae;D_2__Lentisphaeria;D_3__Lentisphaerales;D_4__Lentisphaeraceae;D_5__Lentisphaera
e12d3f20ba531e929b3a60a1f615ea5b	8	0.83	D_0__Bacteria;D_1__Acidobacteria;D_2__Subgroup 6;D_3__uncultured bacterium;D_4__uncultured bacterium;D_5__uncultured bacterium;D_6__uncultured bacterium
40e56b05348058467f6f102250158342	8	1.00	D_0__Bacteria;D_1__Proteobacteria;D_2__Deltaproteobacteria;D_3__Bdellovibrionales;D_4__Bacteriovoracaceae
f33d78c9c35e47d53f4efb931cf10bf4	8	1.00	D_0__Bacteria;D_1__Proteobacteria;D_2__Deltaproteobacteria;D_3__NB1-j
2b4bdffda8e068ad3d6ce3fa641690e7	8	0.93	D_0__Bacteria;D_1__Chloroflexi;D_2__Dehalococcoidia;D_3__SAR202 clade;D_4__uncultured Chloroflexi bacterium;D_5__uncultured Chloroflexi bacterium;D_6__uncultured Chloroflexi bacterium
ede5a829055a14f24b62ce4951de68b1	8	1.00	D_0__Bacteria;D_1__Acidobacteria;D_2__Acidobacteriia;D_3__Solibacterales;D_4__Solibacteraceae (Subgroup 3);D_5__PAUC26f
89bebc6de0eac3b493aa909294c5f738	8	0.91	D_0__Bacteria;D_1__Chloroflexi;D_2__Anaerolineae;D_3__SBR1031;D_4__A4b;Ambiguous_taxa;Ambiguous_taxa
54d0ed65e851cf4d6cb0804f1ebdc7d8	8	0.79	D_0__Bacteria;D_1__Entotheonellaeota;D_2__Entotheonellia;D_3__Entotheonellales;D_4__Entotheonellaceae;D_5__uncultured delta proteobacterium;D_6__uncultured delta proteobacterium
06cee4044600ed6b2fa450d46863950c	8	1.00	D_0__Bacteria;D_1__Proteobacteria;D_2__Deltaproteobacteria;D_3__Bdellovibrionales;D_4__Bdellovibrionaceae;D_5__Bdellovibrio;D_6__uncultured delta proteobacterium
97ae1181d84aac03e7fde5a899585660	8	0.99	D_0__Bacteria;D_1__Proteobacteria;D_2__Gammaproteobacteria;D_3__Nitrosococcales;D_4__Nitrosococcaceae;D_5__AqS1
67ff312feb3ba3201a75f86d95c46ac6	7	0.86	D_0__Bacteria;D_1__Proteobacteria;D_2__Gammaproteobacteria;D_3__Thiomicrospirales;D_4__Thioglobaceae;D_5__SUP05 cluster
91ac3e76986889795b13599e53d0952e	7	1.00	D_0__Bacteria;D_1__Proteobacteria;D_2__Alphaproteobacteria;D_3__Puniceispirillales;D_4__EF100-94H03

dcf1e0f4fc3184efac88c1c93d6e703a	6	0.98	D_0__Bacteria;D_1__Bacteroidetes;D_2__Bacteroidia;D_3__Flavobacteriales;D_4__Flavobacteriaceae;D_5__Aurantivirga;Ambiguous_taxa
5137ef949a0c8bf90cc078dff87db691	6	1.00	D_0__Bacteria;D_1__Bacteroidetes;D_2__Bacteroidia;D_3__Flavobacteriales;D_4__Flavobacteriaceae;D_5__NS2b marine group
68b8bff88f05ebe9a8a21aa60fb98e2f	6	0.99	D_0__Bacteria;D_1__Proteobacteria;D_2__Deltaproteobacteria;D_3__Bdellovibrionales;D_4__Bacteriovoracaceae;D_5__Peredibacter;D_6__uncultured Bacteriovorax sp.
c22e400b0b4d6bbe8ce26be411e71a0e	6	0.98	D_0__Bacteria;D_1__Chloroflexi;D_2__Dehalococcoidia;D_3__SAR202 clade;D_4__uncultured Chloroflexus sp.;D_5__uncultured Chloroflexus sp.;D_6__uncultured Chloroflexus sp.
9ee1da247f6b9d00bb9021204178a006	6	1.00	D_0__Bacteria;D_1__Proteobacteria;D_2__Deltaproteobacteria;D_3__Bdellovibrionales;D_4__Bacteriovoracaceae
8748a4f44f9c973ff8858289e5f129d5	6	1.00	D_0__Bacteria;D_1__Proteobacteria;D_2__Deltaproteobacteria;D_3__Bdellovibrionales;D_4__Bdellovibrionaceae;D_5__Bdellovibrio;D_6__Vibrio sp. Gp-3-1.3.1-voll
f80173b8b8b6ed6af3ea634c805c1b46	6	1.00	D_0__Bacteria;D_1__Patescibacteria;D_2__Parcubacteria;D_3__Candidatus Kaiserbacteria
bb07da13f4962cdf27bf81bfe034f315	6	0.97	D_0__Bacteria;D_1__Proteobacteria;D_2__Deltaproteobacteria;D_3__Bdellovibrionales;D_4__Bdellovibrionaceae;D_5__Bdellovibrio;D_6__uncultured delta proteobacterium
58ae890b1fda6270baf0b22517c44781	6	0.90	D_0__Bacteria;D_1__Proteobacteria;D_2__Gammaproteobacteria;D_3__HOC36;D_4__uncultured bacterium;D_5__uncultured bacterium;D_6__uncultured bacterium
ccf2a3ec00b404364a421bd83d3ff0cf	5	1.00	D_0__Bacteria;D_1__Bacteroidetes;D_2__Bacteroidia;D_3__Flavobacteriales;D_4__NS9 marine group;D_5__uncultured marine bacterium;D_6__uncultured marine bacterium
8e93ff62d0cc769ac5e520a48a8a2ae6	5	1.00	D_0__Bacteria;D_1__Planctomycetes;D_2__Planctomycetacia;D_3__Pirellulales;D_4__Pirellulaceae;D_5__Rhodopirellula;D_6__marine metagenome
67e7850681f5c9fb8a49514daec61f00	5	0.95	D_0__Bacteria;D_1__Proteobacteria;D_2__Alphaproteobacteria;D_3__SAR11 clade;D_4__Clade IV;D_5__uncultured marine bacterium;D_6__uncultured marine bacterium
33843aa51792715720a27c110f66f31e	5	1.00	D_0__Bacteria;D_1__Proteobacteria;D_2__Gammaproteobacteria;D_3__KI89A clade;D_4__uncultured bacterium;D_5__uncultured bacterium;D_6__uncultured bacterium

8b6b3fd03635f7e210ba4aff715c1872	5	0.90	D_0__Bacteria;D_1__Patescibacteria;D_2__Parcubacteria;D_3__Candidatus Spechtbacteria;D_4__uncultured bacterium;D_5__uncultured bacterium;D_6__uncultured bacterium
3ba53e7994c54e763a51f5eb9ca7aa4d	5	0.87	D_0__Bacteria;D_1__Proteobacteria;D_2__Gammaproteobacteria;D_3__Nitrosococcales;D_4__Nitrosococcaceae;D_5__AqS1;D_6__uncultured marine bacterium
8835217731dd20a9c167182a6aff5ee4	5	1.00	D_0__Bacteria;D_1__Proteobacteria;D_2__Alphaproteobacteria;D_3__Rhodospirillales;D_4__AEGEAN-169 marine group;Ambiguous_taxa;Ambiguous_taxa
ad24af429cfa122edcd73e93349c4d95	5	1.00	D_0__Bacteria;D_1__Proteobacteria;D_2__Deltaproteobacteria;D_3__Bdellovibrionales;D_4__Bacteriovoracaceae
5182b6032a99449bcf71040b49247f14	5	1.00	D_0__Bacteria;D_1__Proteobacteria;D_2__Alphaproteobacteria;D_3__Puniceispirillales;D_4__SAR116 clade;D_5__uncultured organism;D_6__uncultured organism
2c9695a7330d97c5996acf5b965247cc	5	1.00	D_0__Bacteria;D_1__Proteobacteria;D_2__Deltaproteobacteria;D_3__Bdellovibrionales;D_4__Bdellovibrionaceae;D_5__Bdellovibrio;D_6__uncultured Bdellovibrio sp.
cc644d614b0dfc45aae06a2dc979fed5	5	0.82	D_0__Bacteria;D_1__Proteobacteria;D_2__Deltaproteobacteria;D_3__Bdellovibrionales;D_4__Bacteriovoracaceae;D_5__Peredibacter;D_6__uncultured Bacteriovoracaceae bacterium
39fad84774ff6d94b7798053096f59e3	5	0.99	D_0__Bacteria;D_1__Proteobacteria;D_2__Deltaproteobacteria;D_3__Bdellovibrionales;D_4__Bdellovibrionaceae;D_5__Bdellovibrio;D_6__uncultured delta proteobacterium
91f860f57ab6e8edd8df264f1af928ae	5	0.93	D_0__Bacteria;D_1__Entotheonellaeota;D_2__Entotheonellia;D_3__Entotheonellales;D_4__Entotheonellaceae;D_5__u ncultured delta proteobacterium;D_6__uncultured delta proteobacterium
6fdb2876b8fe6afdd319e899f73b2a26	4	0.83	D_0__Bacteria;D_1__Proteobacteria;D_2__Gammaproteobacteria;D_3__Arenicellales;D_4__Arenicellaceae;D_5__unc ultured;D_6__uncultured bacterium
d45befa63ed56d7b57c176e41a988f6c	4	1.00	D_0__Bacteria;D_1__Proteobacteria;D_2__Gammaproteobacteria
b179f619cfbd75605d6cb3bf0edb8de9	4	0.80	D_0__Bacteria;D_1__Proteobacteria;D_2__Alphaproteobacteria;D_3__Rhodospirillales;D_4__AEGEAN-169 marine group;D_5__unidentified marine bacterioplankton;D_6__unidentified marine bacterioplankton
6c291eec175c8c1a7f339d6cff83b3af	4	0.94	D_0__Bacteria;D_1__Proteobacteria;D_2__Deltaproteobacteria;D_3__Bdellovibrionales;D_4__Bacteriovoracaceae;D_5__Peredibacter

a2564a9303e130fda4343e15648c8e18	4	1.00	D_0__Bacteria;D_1__Proteobacteria;D_2__Deltaproteobacteria;D_3__Bdellovibrionales;D_4__Bdellovibrionaceae;D_5__Bdellovibrio
3afad7ec5e737d30c250eae48f1af0d0	3	0.98	D_0__Bacteria;D_1__Proteobacteria;D_2__Alphaproteobacteria
d49791e1d7ea4b6fae9350e9effcc53e	3	0.92	D_0__Bacteria;D_1__Actinobacteria;D_2__Acidimicrobiia;D_3__Actinomarinales;D_4__Actinomarinaceae;D_5__Candidatus Actinomarina;D_6__Candidatus Actinomarina minuta
aba67bc2c9c151f7435c2de7251b6f21	3	0.97	D_0__Bacteria;D_1__Proteobacteria;D_2__Alphaproteobacteria;D_3__Rhodobacterales;D_4__Rhodobacteraceae;D_5__Ruegeria;Ambiguous_taxa
91929c431e38588b4d25eb30324b9b63	3	0.92	D_0__Bacteria;D_1__Spirochaetes;D_2__Spirochaetia;D_3__Spirochaetales;D_4__Spirochaetaceae;D_5__Spirochaeta 2;D_6__uncultured bacterium
9f17d52bc7ca9fbdffed33cf0a4a1e37	3	1.00	D_0__Bacteria;D_1__Proteobacteria
3060d80239f23ffce9c04f2f8f2f8a70	2	1.00	D_0__Bacteria;D_1__Chloroflexi;D_2__Dehalococcoidia;D_3__SAR202 clade
e5f9ce8b4d82dbbda8f78e1e74c57bfa	2	1.00	D_0__Bacteria;D_1__Dadabacteria;D_2__Dadabacteriia;D_3__Dadabacteriales
fdb93e66361367611bbbc4ecc6f7b266	1	0.77	D_0__Bacteria;D_1__Proteobacteria;D_2__Gammaproteobacteria;D_3__SAR86 clade;D_4__uncultured bacterium ARCTIC11_B_09;D_5__uncultured bacterium ARCTIC11_B_09;D_6__uncultured bacterium ARCTIC11_B_09
48d4096a2316eca6a255c285efd4e646	1	0.95	D_0__Bacteria;D_1__Proteobacteria;D_2__Deltaproteobacteria;D_3__Bdellovibrionales;D_4__Bdellovibrionaceae;D_5__Bdellovibrio
c92483349bf20d60a77c9978461d07a1	1	1.00	D_0__Bacteria;D_1__Proteobacteria;D_2__Gammaproteobacteria
04c3add4b12d0918d8a2d6e46e08c3e5	1	0.83	D_0__Bacteria;D_1__Proteobacteria;D_2__Gammaproteobacteria;D_3__OM182 clade
7a0f4f78b2d69ba370dd8e319ff8894b	1	0.81	D_0__Bacteria;D_1__Proteobacteria;D_2__Gammaproteobacteria;D_3__Nitrosococcales;D_4__Nitrosococcaceae;D_5__AqS1;D_6__uncultured marine bacterium
9308a35a3d318509def274907e443073	1	1.00	D_0__Bacteria;D_1__Proteobacteria;D_2__Gammaproteobacteria;D_3__KI89A clade;D_4__uncultured bacterium;D_5__uncultured bacterium;D_6__uncultured bacterium

Table S5. ASVs (142) from *Erylus discophorus* with percentage of confidence ≥ 75%.

Feature ID	Number of sequences	Confidence	Taxon
854f4e49244b64e70125efe1ffe68a8f	1048	0.98	D_0__Bacteria;D_1__Actinobacteria;D_2__Acidimicrobiia;D_3__Microtrichales;D_4__Microtrichaceae;D_5__Sva0996 marine group;D_6__uncultured bacterium
7a2a06d3f2f4b501aa65909a818c2897	678	1.00	D_0__Bacteria;D_1__Actinobacteria;D_2__Acidimicrobiia;D_3__Microtrichales;D_4__Microtrichaceae;D_5__Sva0996 marine group
2e06d9a8dd891e62aa404eb0285ef3ad	614	1.00	D_0__Bacteria;D_1__Gemmatimonadetes;D_2__BD2-11 terrestrial group
851f2657ffac970feead9638b27e4c33	611	0.93	D_0__Bacteria;D_1__Nitrospirae;D_2__Nitrospira;D_3__Nitrospirales;D_4__Nitrospiraceae;D_5__Nitrospira;D_6__uncultured Nitrospirae bacterium
e8753c670d63c0001e8a4c2d232451c4	563	1.00	D_0__Bacteria;D_1__Acidobacteria;D_2__Acidobacteriia;D_3__Solibacterales;D_4__Solibacteraceae (Subgroup 3);D_5__PAUC26f;D_6__uncultured bacterium
851e8012e91152230e8ac62f1a6d8884	474	1.00	D_0__Bacteria;D_1__Proteobacteria;D_2__Gammaproteobacteria;D_3__KI89A clade;D_4__uncultured bacterium;D_5__uncultured bacterium;D_6__uncultured bacterium
fd48905167b54399e95c363d9b17bf80	420	0.99	D_0__Archaea;D_1__Thaumarchaeota;D_2__Nitrososphaeria;D_3__Nitrosopumilales;D_4__Nitrosopumilaceae;D_5__Candidatus Nitrosopumilus
9101a4e6eae37bde17cf64fb7be21246	396	1.00	D_0__Bacteria;D_1__Acidobacteria;D_2__Acidobacteriia;D_3__Solibacterales;D_4__Solibacteraceae (Subgroup 3);D_5__PAUC26f;D_6__uncultured bacterium
548e719ca9a94d9abcdfed16db76efba	387	0.98	D_0__Bacteria;D_1__Poribacteria;D_2__Candidatus Poribacteria bacterium WGA-4C;D_3__Candidatus Poribacteria bacterium WGA-4C;D_4__Candidatus Poribacteria bacterium WGA-4C;D_5__Candidatus Poribacteria bacterium WGA-4C;D_6__Candidatus Poribacteria bacterium WGA-4C
be3c73452201b90ef6d4c0ede59f7827	382	0.88	D_0__Bacteria;D_1__Actinobacteria;D_2__Acidimicrobiia;D_3__Microtrichales;D_4__Microtrichaceae;D_5__Sva0996 marine group;D_6__uncultured actinobacterium

4d58056cc0ef38da08ac85896bfcbb22	362	1.00	D_0__Bacteria;D_1__Poribacteria;D_2__Candidatus Poribacteria bacterium WGA-4C;D_3__Candidatus Poribacteria bacterium WGA-4C;D_4__Candidatus Poribacteria bacterium WGA-4C;D_5__Candidatus Poribacteria bacterium WGA-4C;D_6__Candidatus Poribacteria bacterium WGA-4C
440372014bb52e796573e652206cd42b	338	0.95	D_0__Bacteria;D_1__PAUC34f;Ambiguous_taxa;Ambiguous_taxa;Ambiguous_taxa;Ambiguous_taxa;Ambiguous_taxa
1bb1706de6355fa5b0c0e20d631a2edd	285	1.00	D_0__Bacteria;D_1__Chloroflexi;D_2__TK17
8af02bc807f04196cebfefd60b7b1ceb	247	0.80	D_0__Bacteria;D_1__Actinobacteria;D_2__Acidimicrobiia;D_3__Microtrichales;D_4__Microtrichaceae;D_5__Sva0996 marine group;D_6__actinobacterium MSI70
471195b09a0fb63742bebcd7cdbe8873	219	1.00	D_0__Bacteria;D_1__Proteobacteria;D_2__Gammaproteobacteria;D_3__EPR3968-O8a-Bc78;Ambiguous_taxa;Ambiguous_taxa;Ambiguous_taxa
d1ae44a6ce6cb531cffa434a812cc1af	211	0.98	D_0__Bacteria;D_1__Dadabacteria;D_2__Dadabacteriia;D_3__Dadabacteriales;D_4__uncultured delta proteobacterium;D_5__uncultured delta proteobacterium;D_6__uncultured delta proteobacterium
3b2ed357b7db45320c77760b8294db42	206	0.98	D_0__Bacteria;D_1__Acidobacteria;D_2__Subgroup 9;D_3__uncultured bacterium;D_4__uncultured bacterium;D_5__uncultured bacterium;D_6__uncultured bacterium
0b592c4e9a81331b114d9419dc69bc83	198	1.00	D_0__Bacteria;D_1__Gemmatimonadetes;D_2__BD2-11 terrestrial group
1d77e4f7f18d7aa6bdafd1c742fd0c40	193	1.00	D_0__Bacteria;D_1__Bacteroidetes;D_2__Rhodothermia;D_3__Rhodothermales;D_4__Rhodothermaceae;D_5__uncultured
f66c2a7cae98e194102fa235871c78f4	185	1.00	D_0__Bacteria;D_1__Gemmatimonadetes;D_2__BD2-11 terrestrial group
40dd3ce434a77b9cb9cec052318a6dac	166	1.00	D_0__Bacteria;D_1__Chloroflexi;D_2__Dehalococcoidia;D_3__SAR202 clade
408b59118f65314c1c90b2b5f6a55dbb	159	1.00	D_0__Bacteria;D_1__Acidobacteria;D_2__Thermoanaerobaculia;D_3__Thermoanaerobaculales;D_4__Thermoanaerobaculaceae;D_5__Subgroup 10
921a66099036cb00fc5d09439e9fb6d4	158	0.84	D_0__Bacteria;D_1__Chloroflexi;D_2__Dehalococcoidia;D_3__SAR202 clade;D_4__uncultured bacterium;D_5__uncultured bacterium;D_6__uncultured bacterium
84c091f50111453bf7bdd8132d5d5b43	141	1.00	D_0__Bacteria;D_1__Proteobacteria;D_2__Gammaproteobacteria;D_3__Steroidobacterales;D_4__Woeseiaceae;D_5__JTB255 marine benthic group;Ambiguous_taxa

5fe55b01e9730b483cf5163eb019384e	132	0.78	D_0__Bacteria;D_1__Chloroflexi;D_2__JG30-KF-CM66;Ambiguous_taxa;Ambiguous_taxa;Ambiguous_taxa;Ambiguous_taxa
dd0f61ed38a385bfdf7966ff19333b16	127	0.99	D_0__Bacteria;D_1__Actinobacteria;D_2__Acidimicrobiia;D_3__Actinomarinales;D_4__uncultured;D_5__uncultured actinobacterium;D_6__uncultured actinobacterium
cb3903e58b3e61c940045cf599b3812c	121	0.87	D_0__Bacteria;D_1__Gemmatimonadetes;D_2__BD2-11 terrestrial group;D_3__uncultured bacterium;D_4__uncultured bacterium;D_5__uncultured bacterium;D_6__uncultured bacterium
675610c0fdeabcb59b9fc9b2094f4462	120	0.77	D_0__Bacteria;D_1__Gemmatimonadetes;D_2__PAUC43f marine benthic group;D_3__uncultured bacterium;D_4__uncultured bacterium;D_5__uncultured bacterium;D_6__uncultured bacterium
7b44eb00032d861a29316601d8131106	106	0.98	D_0__Bacteria;D_1__Proteobacteria;D_2__Gammaproteobacteria;D_3__JTB23;D_4__uncultured bacterium;D_5__uncultured bacterium;D_6__uncultured bacterium
cbe097b2843bf71d7e66ed46b947a58a	103	0.75	D_0__Bacteria;D_1__Proteobacteria;D_2__Gammaproteobacteria;D_3__HOC36;Ambiguous_taxa;Ambiguous_taxa;Ambiguous_taxa
793045ce6707d8593a7aa016b3d425cb	102	1.00	D_0__Bacteria;D_1__Chloroflexi;D_2__Anaerolineae;D_3__SBR1031;D_4__A4b
e2cb43f92be46162001c56192fbfa46b	98	0.89	D_0__Bacteria;D_1__Proteobacteria;D_2__Gammaproteobacteria;D_3__EPR3968-O8a-Bc78;D_4__uncultured bacterium;D_5__uncultured bacterium;D_6__uncultured bacterium
7fb620f63b0ba7a2dcca8d3b6b3b7182	97	1.00	D_0__Bacteria;D_1__Proteobacteria;D_2__Gammaproteobacteria;D_3__KI89A clade
b40d45e15fd2e2b3beb51b1c84626a41	96	1.00	D_0__Bacteria;D_1__Proteobacteria;D_2__Gammaproteobacteria;D_3__Steroidobacterales;D_4__Woeseiaceae;D_5__JTB255 marine benthic group;Ambiguous_taxa
ab16214a92b9c6584fc61174c967ba27	91	1.00	D_0__Bacteria;D_1__Acidobacteria;D_2__Subgroup 6
02dbb71ca3012d339c35e4ce491ccf4d	89	0.93	D_0__Bacteria;D_1__PAUC34f;D_2__uncultured Deferribacteres bacterium;D_3__uncultured Deferribacteres bacterium;D_4__uncultured Deferribacteres bacterium;D_5__uncultured Deferribacteres bacterium;D_6__uncultured Deferribacteres bacterium
343128e8b51345ce61bcaabb1a70f898	79	1.00	D_0__Bacteria;D_1__Proteobacteria;D_2__Alphaproteobacteria;D_3__Puniceispirillales;Ambiguous_taxa;Ambiguous_taxa;Ambiguous_taxa

780bb610683bccb8da96924032841de5	74	1.00	D_0__Bacteria;D_1__Proteobacteria;D_2__Gammaproteobacteria;D_3__Nitrosococcales;D_4__Nitrosococcaceae;D_5__AqS1
c18fd2bfef8e264fdbf032d29a8cc71c	73	1.00	D_0__Bacteria;D_1__Poribacteria;D_2__Candidatus Poribacteria bacterium WGA-4C;D_3__Candidatus Poribacteria bacterium WGA-4C;D_4__Candidatus Poribacteria bacterium WGA-4C;D_5__Candidatus Poribacteria bacterium WGA-4C;D_6__Candidatus Poribacteria bacterium WGA-4C
4d2f1794d620881577baf24aaa2fffa2	72	0.83	D_0__Bacteria;D_1__Proteobacteria;D_2__Alphaproteobacteria;D_3__Sneathiellales;D_4__Sneathiellaceae;D_5__AT-s3-44;D_6__uncultured bacterium
bc7ef34401f0e8edb858c24bd0a996f9	67	0.86	D_0__Bacteria;D_1__Nitrospirae;D_2__Nitrospira;D_3__Nitrospirales;D_4__Nitrospiraceae;D_5__Nitrospira;D_6__uncultured Nitrospirae bacterium
15f6fa09e73e03a57b15782457b2e070	67	0.89	D_0__Bacteria;D_1__Actinobacteria;D_2__Acidimicrobiia;D_3__Microtrichales;D_4__Microtrichaceae;D_5__Sva0996 marine group;Ambiguous_taxa
a5335857f940b9ecf53c323be01e8f25	64	0.99	D_0__Bacteria;D_1__Proteobacteria;D_2__Gammaproteobacteria;D_3__JTB23;D_4__uncultured bacterium;D_5__uncultured bacterium;D_6__uncultured bacterium
ede01e35b9bda5596afaf5de344be60a	60	1.00	D_0__Bacteria;D_1__Poribacteria;D_2__uncultured Clostridium sp.;D_3__uncultured Clostridium sp.;D_4__uncultured Clostridium sp.;D_5__uncultured Clostridium sp.;D_6__uncultured Clostridium sp.
5482cfb4b230acd5e15f63d13a932d5a	59	1.00	D_0__Bacteria;D_1__Verrucomicrobia;D_2__Verrucomicrobiae;D_3__Opitutales;D_4__Puniceicoccaceae;D_5__Cerasicoccus;Ambiguous_taxa
e3a85f83ff526059c3be54bab904f84f	58	1.00	D_0__Bacteria;D_1__Chloroflexi;D_2__JG30-KF-CM66
dda81ed5756d61b76454b48695d3f504	54	0.99	D_0__Bacteria;D_1__Proteobacteria;D_2__Gammaproteobacteria;D_3__KI89A clade;D_4__uncultured bacterium;D_5__uncultured bacterium;D_6__uncultured bacterium
27dfde9ef4d98288c46ee88bc4f2d30a	54	0.76	D_0__Bacteria;D_1__Chloroflexi;D_2__TK30;Ambiguous_taxa;Ambiguous_taxa;Ambiguous_taxa;Ambiguous_taxa
0f4a02277d3363604c1f95c85fe1e07e	53	1.00	D_0__Bacteria;D_1__Chloroflexi;D_2__Dehalococcoidia;D_3__SAR202 clade
92479a958d13d8729846c440e84434a0	53	1.00	D_0__Bacteria;D_1__Chloroflexi;D_2__Dehalococcoidia;D_3__SAR202 clade

0d319457b4c520ce1e2d4120f0704b17	53	0.95	D_0__Bacteria;D_1__PAUC34f;D_2__uncultured Deferribacteres bacterium;D_3__uncultured Deferribacteres bacterium;D_4__uncultured Deferribacteres bacterium;D_5__uncultured Deferribacteres bacterium;D_6__uncultured Deferribacteres bacterium
7bf4a14498b8eddff75fe495902f6e32	53	0.91	D_0__Bacteria;D_1__Proteobacteria;D_2__Gammaproteobacteria;D_3__EPR3968-O8a-Bc78;D_4__uncultured bacterium;D_5__uncultured bacterium;D_6__uncultured bacterium
e23c156642078fb48f89bc01faadac18	52	0.99	D_0__Bacteria;D_1__Poribacteria;Ambiguous_taxa;Ambiguous_taxa;Ambiguous_taxa;Ambiguous_taxa;Ambiguous_taxa
fa0846f779fbf4f2903618e2dcc6f3c0	48	0.83	D_0__Bacteria;D_1__Proteobacteria;D_2__Deltaproteobacteria;D_3__Myxococcales;D_4__bacteriap25;Ambiguous_taxa;Ambiguous_taxa
dc9e89fcc0e372deb4ad8ebd6b660162	45	0.76	D_0__Bacteria;D_1__Actinobacteria;D_2__Acidimicrobiia;D_3__Microtrichales;D_4__Microtrichaceae;D_5__Sva0996 marine group;D_6__uncultured actinobacterium
4e5a2f5d71ecef14a9892319e6739ab2	44	0.91	D_0__Bacteria;D_1__Proteobacteria;D_2__Gammaproteobacteria;D_3__JTB23;D_4__uncultured bacterium;D_5__uncultured bacterium;D_6__uncultured bacterium
8a209b077e9310c40529c899cfc28412	40	0.90	D_0__Bacteria;D_1__Nitrospinae;D_2__P9X2b3D02;D_3__uncultured bacterium;D_4__uncultured bacterium;D_5__uncultured bacterium;D_6__uncultured bacterium
f73c36170563004ed97fe815be7468f7	40	0.90	D_0__Bacteria;D_1__Gemmatimonadetes;D_2__BD2-11 terrestrial group;D_3__uncultured bacterium;D_4__uncultured bacterium;D_5__uncultured bacterium;D_6__uncultured bacterium
31fff8d12b7f55a0547a4400045e7b5e	39	1.00	D_0__Bacteria;D_1__Chloroflexi;D_2__Dehalococcoidia;D_3__SAR202 clade
59ba4ec313924002f2058b8a8cf25b7b	37	0.98	D_0__Bacteria;D_1__Chloroflexi;D_2__Anaerolineae;D_3__SBR1031;D_4__A4b;D_5__uncultured Chloroflexus sp.;D_6__uncultured Chloroflexus sp.
bfef3b3d36a5fb763ebd0422c74d1733	36	1.00	D_0__Bacteria;D_1__Chloroflexi;D_2__Anaerolineae;D_3__SBR1031;D_4__A4b;D_5__uncultured Chloroflexus sp.;D_6__uncultured Chloroflexus sp.
2194a824269dfed37ff5873706fe5fcc	35	0.97	D_0__Bacteria;D_1__Actinobacteria;D_2__Acidimicrobiia;D_3__Microtrichales;D_4__Microtrichaceae;D_5__Sva0996 marine group;D_6__actinobacterium MSI70
ab0893b7de88347f92003023342fa769	35	0.92	D_0__Bacteria;D_1__Actinobacteria;D_2__Acidimicrobiia;D_3__Microtrichales;D_4__Microtrichaceae;D_5__Sva0996 marine group;D_6__uncultured actinobacterium

2556ff27c8b418b9c038f90ca8eaa7f3	35	0.75	D_0__Bacteria;D_1__Proteobacteria;D_2__Gammaproteobacteria;D_3__OM182 clade;D_4__uncultured gamma proteobacterium;D_5__uncultured gamma proteobacterium;D_6__uncultured gamma proteobacterium
7bd374a6c270a0c0a0477cc7067a61c3	35	1.00	D_0__Bacteria;D_1__Proteobacteria;D_2__Alphaproteobacteria;D_3__Rhodobacterales;D_4__Rhodobacteraceae
f34d1fbf32dc7b137af8bc422a131bb5	33	1.00	D_0__Bacteria;D_1__Chloroflexi;D_2__Dehalococcoidia;D_3__SAR202 clade
74fb75f885374e290262a669196ea182	33	0.78	D_0__Bacteria;D_1__AncK6;D_2__uncultured delta proteobacterium;D_3__uncultured delta proteobacterium;D_4__uncultured delta proteobacterium;D_5__uncultured delta proteobacterium;D_6__uncultured delta proteobacterium
3bb53e6579e782a8fccf15872926a521	32	0.91	D_0__Bacteria;D_1__Proteobacteria;D_2__Alphaproteobacteria;D_3__Sneathiellales;D_4__Sneathiellaceae;D_5__AT-s3-44;D_6__uncultured bacterium
8d21848ccfc247e9e92028e02e2cfd66	29	1.00	D_0__Bacteria;D_1__Acidobacteria;D_2__Subgroup 6
1ce7efd5759facd9083eafde3a31781d	29	1.00	D_0__Bacteria;D_1__Proteobacteria;D_2__Gammaproteobacteria;D_3__KI89A clade;D_4__uncultured bacterium;D_5__uncultured bacterium;D_6__uncultured bacterium
47f7cd8e48355810280cfdd9253dee9e	28	1.00	D_0__Bacteria;D_1__Poribacteria;D_2__Candidatus Poribacteria bacterium WGA-4C;D_3__Candidatus Poribacteria bacterium WGA-4C;D_4__Candidatus Poribacteria bacterium WGA-4C;D_5__Candidatus Poribacteria bacterium WGA-4C;D_6__Candidatus Poribacteria bacterium WGA-4C
a1e583abe9ddf5c937cf9710203ca1a8	28	0.98	D_0__Bacteria;D_1__Proteobacteria;D_2__Gammaproteobacteria;D_3__Nitrosococcales;D_4__Nitrosococcaceae;D_5__AqS1;D_6__uncultured marine bacterium
a8319db4cc193409bb9f79a6c8259839	26	0.97	D_0__Bacteria;D_1__Proteobacteria;D_2__Alphaproteobacteria;D_3__uncultured
ff32f0f497298d7e5f2977e831e447e2	26	1.00	D_0__Bacteria;D_1__Acidobacteria;D_2__Subgroup 6
03adfab8f1649f310edb31130ffe42a4	26	0.99	D_0__Bacteria;D_1__Actinobacteria;D_2__Acidimicrobiia;D_3__Microtrichales;D_4__Microtrichaceae;D_5__Sva0996 marine group
f9c2a75f1329dcc7a3ab77204ec61d5b	25	1.00	D_0__Bacteria;D_1__Chloroflexi;D_2__Dehalococcoidia;D_3__SAR202 clade
10640292c2a101b8596ee78e386b4e39	25	1.00	D_0__Bacteria;D_1__Proteobacteria;D_2__Gammaproteobacteria;D_3__EPR3968-O8a-Bc78;Ambiguous_taxa;Ambiguous_taxa;Ambiguous_taxa

3a17a62414627555360e70cb6ae523cc	24	0.94	D_0__Bacteria;D_1__Proteobacteria;D_2__Alphaproteobacteria;D_3__Rhodovibrionales;D_4__Kiloniellaceae;D_5__uncultured;D_6__uncultured bacterium
30e12c3496fff9a956ed0ff908667b3c	23	0.83	D_0__Bacteria;D_1__Proteobacteria;D_2__Gammaproteobacteria;D_3__KI89A clade;D_4__uncultured bacterium;D_5__uncultured bacterium;D_6__uncultured bacterium
fd867f92e3851ddd3630d30f7f2f7b7a	23	0.89	D_0__Bacteria;D_1__Proteobacteria;D_2__Alphaproteobacteria;D_3__Sneathiellales;D_4__Sneathiellaceae;D_5__AT-s3-44;D_6__uncultured bacterium
9865f63ba4eda05ffd69de6e44c98bdd	22	1.00	D_0__Bacteria;D_1__Proteobacteria;D_2__Alphaproteobacteria;D_3__Puniceispirillales;D_4__EF100-94H03;Ambiguous_taxa;Ambiguous_taxa
26203390307e16f46b16b1469177aaa8	22	0.85	D_0__Bacteria;D_1__Gemmatimonadetes;D_2__PAUC43f marine benthic group;D_3__uncultured bacterium;D_4__uncultured bacterium;D_5__uncultured bacterium;D_6__uncultured bacterium
91405b14b91dc95506affd822ebae203	21	1.00	D_0__Bacteria;D_1__Firmicutes;D_2__Bacilli;D_3__Bacillales;D_4__Family XII;D_5__Exiguobacterium
9cb095b0b9dabdad7d1156f225fd4a0a	21	1.00	D_0__Bacteria;D_1__Proteobacteria;D_2__Deltaproteobacteria
180fedbc153f1a1b30bfe85bdcc0dc4c	21	0.91	D_0__Bacteria;D_1__Actinobacteria;D_2__Acidimicrobiia;D_3__Actinomarinales;D_4__uncultured;Ambiguous_taxa; Ambiguous_taxa
279243e03c1efffef876c2aac925a677	21	0.82	D_0__Bacteria;D_1__Actinobacteria;D_2__Acidimicrobiia;D_3__Microtrichales;D_4__Microtrichaceae;D_5__Sva0996 marine group;D_6__uncultured actinobacterium
94765c8a51ad1b53c72cfa58a4e208e1	20	1.00	D_0__Bacteria;D_1__Poribacteria;D_2__uncultured Clostridium sp.;D_3__uncultured Clostridium sp.;D_4__uncultured Clostridium sp.;D_5__uncultured Clostridium sp.;D_6__uncultured Clostridium sp.
f124bfe2ad946e478a9b2a1450ed9aaf	20	1.00	D_0__Bacteria;D_1__Chloroflexi;D_2__Dehalococcoidia;D_3__SAR202 clade
da12db4ab27cd8061c031ebcca1f0003	19	1.00	D_0__Bacteria;D_1__Proteobacteria;D_2__Gammaproteobacteria;D_3__KI89A clade;D_4__uncultured bacterium;D_5__uncultured bacterium;D_6__uncultured bacterium
c5ce5fedffa09328ae705cef668d6285	19	1.00	D_0__Bacteria;D_1__Proteobacteria;D_2__Gammaproteobacteria;D_3__pItb-vmat-80;D_4__uncultured bacterium;D_5__uncultured bacterium;D_6__uncultured bacterium
316f64c416fcdbd313b194e24fd974c7	19	1.00	D_0__Bacteria;D_1__Proteobacteria;D_2__Gammaproteobacteria;D_3__UBA10353 marine group

780d2d6ba894eac5bd9ae6a0c90b4692	19	0.99	D_0__Bacteria;D_1__Proteobacteria;D_2__Alphaproteobacteria;D_3__uncultured;D_4__uncultured bacterium;D_5__uncultured bacterium;D_6__uncultured bacterium
056a34391a66945f09c782d54be93313	18	0.82	D_0__Bacteria;D_1__Chloroflexi;D_2__Dehalococcoidia;D_3__SAR202 clade;D_4__uncultured Chloroflexi bacterium;D_5__uncultured Chloroflexi bacterium;D_6__uncultured Chloroflexi bacterium
38cefcbb1b5ffdac825b27dbe17271aa	18	1.00	D_0__Bacteria;D_1__Chloroflexi;D_2__Dehalococcoidia;D_3__SAR202 clade
679aee879fd5868fad6e4b808c09c6d5	17	1.00	D_0__Bacteria;D_1__Poribacteria;Ambiguous_taxa;Ambiguous_taxa;Ambiguous_taxa;Ambiguous_taxa;Ambiguous_ta xa
f3f6062f5d2247b18625e15d048f943a	17	1.00	D_0__Bacteria;D_1__Acidobacteria;D_2__Subgroup 6
2d44639ad050a6d3c4ecef9eeb3d7f42	16	1.00	D_0__Bacteria;D_1__Acidobacteria;D_2__Subgroup 6
86b607314dc1d54080da5de2697058ba	15	0.84	D_0__Bacteria;D_1__Acidobacteria;D_2__Subgroup 6;D_3__uncultured bacterium;D_4__uncultured bacterium;D_5__uncultured bacterium;D_6__uncultured bacterium
07b1f2959d9e88eca130090027ca72aa	15	0.98	D_0__Bacteria;D_1__Chloroflexi;D_2__Dehalococcoidia;D_3__SAR202 clade;D_4__uncultured bacterium;D_5__uncultured bacterium;D_6__uncultured bacterium
dfc774b95c692f50a08706f8c793f29a	15	1.00	D_0__Bacteria;D_1__Chloroflexi;D_2__Anaerolineae;D_3__SBR1031;D_4__A4b
f97b500e62ccb9b4930eaada5f6847bf	14	0.98	D_0__Bacteria;D_1__Proteobacteria;D_2__Alphaproteobacteria;D_3__uncultured
1b972ad19a7a3b059c3fb330b4147bc9	14	1.00	D_0__Bacteria;D_1__Cyanobacteria;D_2__Oxyphotobacteria;D_3__Nostocales;D_4__Prochloraceae;D_5__Prochloron P1-Palau
81c21fe55c98672337ef2fefe95ff5ee	12	0.84	D_0__Bacteria;D_1__Proteobacteria;D_2__Alphaproteobacteria;D_3__Rhodospirillales;D_4__Magnetospiraceae;D_5__uncultured;D_6__uncultured bacterium
8dbdbb0927c2b1680a9c74b95009203d	12	0.78	D_0__Bacteria;D_1__PAUC34f;D_2__uncultured Deferribacteres bacterium;D_3__uncultured Deferribacteres bacterium;D_4__uncultured Deferribacteres bacterium;D_5__uncultured Deferribacteres bacterium;D_6__uncultured Deferribacteres bacterium
ba8f3cd31f8d458714462f83beb8fe64	10	0.90	D_0__Bacteria;D_1__Proteobacteria;D_2__Gammaproteobacteria;D_3__Oceanospirillales;D_4__Pseudohongiellaceae;D_5__Pseudohongiella;D_6__uncultured bacterium

51d927e913a1083dd93869b5008bfbfd	10	1.00	D_0__Bacteria;D_1__Gemmatimonadetes;D_2__BD2-11 terrestrial group
907a73ea999935824b0b2453969e99a6	8	0.85	D_0__Bacteria;D_1__Chloroflexi;D_2__Dehalococcoidia;D_3__SAR202 clade;D_4__uncultured Chloroflexi bacterium;D_5__uncultured Chloroflexi bacterium;D_6__uncultured Chloroflexi bacterium
77e5e068af7ed05154eb464218768112	8	1.00	D_0__Bacteria;D_1__Proteobacteria;D_2__Gammaproteobacteria;D_3__pItb-vmat-80;D_4__uncultured bacterium;D_5__uncultured bacterium;D_6__uncultured bacterium
fc4800da3dacf655645ca91a7940fc91	8	1.00	D_0__Bacteria;D_1__Proteobacteria;D_2__Gammaproteobacteria;D_3__KI89A clade;D_4__uncultured gamma proteobacterium;D_5__uncultured gamma proteobacterium;D_6__uncultured gamma proteobacterium
eacb688d550429579977a3a817d0e8d5	8	1.00	D_0__Bacteria;D_1__Acidobacteria;D_2__Subgroup 6
b79586769e3d2d1a8df9a278dd7e5b58	8	1.00	D_0__Bacteria
5a58e98306ed49f6430672b5298e9d92	7	0.99	D_0__Bacteria;D_1__Chloroflexi;D_2__Dehalococcoidia;D_3__SAR202 clade;D_4__uncultured Chloroflexus sp.;D_5__uncultured Chloroflexus sp.;D_6__uncultured Chloroflexus sp.
bcedef9c3b79306c47ae4d0a9e3db64d	7	0.93	D_0__Bacteria;D_1__Proteobacteria;D_2__Deltaproteobacteria;D_3__Bdellovibrionales;D_4__Bacteriovoracaceae;D_5__Peredibacter;D_6__uncultured Bacteriovoracaceae bacterium
17167a25ec8cadc05613156ac8da13dc	7	1.00	D_0__Bacteria;D_1__Chloroflexi;D_2__Dehalococcoidia;D_3__SAR202 clade
211ac56cc9f17100af3480e0895a4250	7	1.00	D_0__Bacteria;D_1__Poribacteria;D_2__Candidatus Poribacteria bacterium WGA-4C;D_3__Candidatus Poribacteria bacterium WGA-4C;D_4__Candidatus Poribacteria bacterium WGA-4C;D_5__Candidatus Poribacteria bacterium WGA-4C;D_6__Candidatus Poribacteria bacterium WGA-4C
44db0a8e5cece22e01376cca56925676	7	0.82	D_0__Bacteria;D_1__Gemmatimonadetes;D_2__BD2-11 terrestrial group;D_3__uncultured bacterium;D_4__uncultured bacterium;D_5__uncultured bacterium;D_6__uncultured bacterium
1c5c4e3317b4de9086e931883f0b9905	7	0.90	D_0__Bacteria;D_1__Proteobacteria;D_2__Deltaproteobacteria;D_3__NB1-j;D_4__uncultured bacterium;D_5__uncultured bacterium;D_6__uncultured bacterium
2e5202c9febc5b65148486edb223eead	6	0.94	D_0__Bacteria;D_1__Bacteroidetes;D_2__Rhodothermia;D_3__Rhodothermales;D_4__Rhodothermaceae;D_5__uncultured;D_6__uncultured bacterium

678c4d816ed36ad976aa8cc560d05248	6	1.00	D_0__Bacteria;D_1__Proteobacteria;D_2__Gammaproteobacteria;D_3__EPR3968-O8a-Bc78;Ambiguous_taxa;Ambiguous_taxa;Ambiguous_taxa
dbeaa8a15dd5da3bce9f9c2e8c3088b4	6	0.90	D_0__Bacteria;D_1__Entotheonellaeota;D_2__Entotheonellia;D_3__Entotheonellales;D_4__Entotheonellaceae;D_5__uncultured delta proteobacterium;D_6__uncultured delta proteobacterium
1d1ed03410df4629cd95235f6e445bad	5	1.00	D_0__Bacteria;D_1__Acidobacteria;D_2__Acidobacteriia;D_3__Solibacterales;D_4__Solibacteraceae (Subgroup 3);D_5__PAUC26f;Ambiguous_taxa
7f1f36bcd007a41f5db7b70fe8a2c1e1	5	0.87	D_0__Bacteria;D_1__Proteobacteria;D_2__Gammaproteobacteria;D_3__Cellvibrionales;D_4__Spongiibacteraceae;D_5__Sinobacterium;D_6__uncultured bacterium
5f30195482dcecf53598a8bb048db9c1	5	0.92	D_0__Bacteria;D_1__Proteobacteria;D_2__Deltaproteobacteria;D_3__Bdellovibrionales;D_4__Bacteriovoracaceae;D_5__Peredibacter;D_6__uncultured Bacteriovoracaceae bacterium
494cbbebe12c5615994b23f587419ac6	4	1.00	D_0__Bacteria;D_1__Proteobacteria;D_2__Gammaproteobacteria;D_3__Enterobacteriales;D_4__Enterobacteriaceae
13c74ffa1c204ac2c3d33816c2e8ac3c	4	0.98	D_0__Bacteria;D_1__PAUC34f;D_2__uncultured Deferribacteres bacterium;D_3__uncultured Deferribacteres bacterium;D_4__uncultured Deferribacteres bacterium;D_5__uncultured Deferribacteres bacterium;D_6__uncultured Deferribacteres bacterium
b4ea6084892aaaa9a51d5f462cfdf591	4	0.93	D_0__Bacteria;D_1__Proteobacteria;D_2__Deltaproteobacteria;D_3__Bdellovibrionales;D_4__Bacteriovoracaceae;D_5__Peredibacter;D_6__uncultured Bacteriovoracaceae bacterium
8bd83882b5707799b6560d2fb1b2bfa5	3	1.00	D_0__Bacteria;D_1__Proteobacteria;D_2__Gammaproteobacteria;D_3__Steroidobacterales;D_4__Woeseiaceae;D_5__JTB255 marine benthic group;Ambiguous_taxa
f28cd0cb187a5be094b950b7882108f2	3	0.97	D_0__Bacteria;D_1__Entotheonellaeota;D_2__Entotheonellia;D_3__Entotheonellales;D_4__Entotheonellaceae;D_5__uncultured delta proteobacterium;D_6__uncultured delta proteobacterium
a7832212353d74bbfca9574c193abae1	3	0.95	D_0__Bacteria;D_1__Proteobacteria;D_2__Alphaproteobacteria;D_3__Caulobacterales;D_4__Hyphomonadaceae;D_5__uncultured;Ambiguous_taxa
640b90ee3ef094e39010abe68904a487	3	0.92	D_0__Bacteria;D_1__Proteobacteria;D_2__Gammaproteobacteria;D_3__Cellvibrionales;D_4__Halieaceae
ee3be8380292d39eb649f533234265a3	2	1.00	D_0__Bacteria;D_1__Proteobacteria;D_2__Gammaproteobacteria;D_3__Nitrosococcales;D_4__Nitrosococcaceae

7105cc2af81a68c6d08b9d8e75c46c9c	2	1.00	D_0__Bacteria;D_1__Proteobacteria;D_2__Gammaproteobacteria
eda1f56cc5091b7c2ff4c2e683b8ff4a	2	0.81	D_0__Bacteria;D_1__Lentisphaerae;D_2__Lentisphaeria;D_3__Lentisphaerales;D_4__Lentisphaeraceae;D_5__Lentisphaera;D_6__uncultured bacterium
e5506a8dd6bbcfc19a9b266868d430b6	2	1.00	D_0__Bacteria
42b39b693fd62f4f47b0f7df2b05c592	1	0.97	D_0__Bacteria;D_1__Cyanobacteria;D_2__Oxyphotobacteria;D_3__Synechococcales;D_4__Synechococcales Incertae Sedis;D_5__Schizothrix LEGE 07164;Ambiguous_taxa
32817791b0193ca80e5335af556f8a30	1	0.98	D_0__Bacteria;D_1__Nitrospinae;D_2__P9X2b3D02;D_3__uncultured bacterium;D_4__uncultured bacterium;D_5__uncultured bacterium;D_6__uncultured bacterium

Table S6. ASVs (111) from *Sarcotragus spinosulus* with percentage of confidence ≥ 75%.

**Feature ID**	**Number of sequences**	**Confidence**	**Taxon**
f9c2a75f1329dcc7a3ab77204ec61d5b	1255	1.00	D_0__Bacteria;D_1__Chloroflexi;D_2__Dehalococcoidia;D_3__SAR202 clade
23825c0d1018211d4b7750b1a12b57ee	1055	1.00	D_0__Bacteria;D_1__Chloroflexi;D_2__Anaerolineae;D_3__Caldilineales;D_4__Caldilineaceae;D_5__uncultured
be3c73452201b90ef6d4c0ede59f7827	895	0.88	D_0__Bacteria;D_1__Actinobacteria;D_2__Acidimicrobiia;D_3__Microtrichales;D_4__Microtrichaceae;D_5__Sva0996 marine group;D_6__uncultured actinobacterium
fa1edc007a7617cd2794fe965f3447f2	646	0.98	D_0__Bacteria;D_1__Dadabacteria;D_2__Dadabacteriia;D_3__Dadabacteriales;D_4__uncultured delta proteobacterium;D_5__uncultured delta proteobacterium;D_6__uncultured delta proteobacterium
907a73ea999935824b0b2453969e99a6	634	0.85	D_0__Bacteria;D_1__Chloroflexi;D_2__Dehalococcoidia;D_3__SAR202 clade;D_4__uncultured Chloroflexi bacterium;D_5__uncultured Chloroflexi bacterium;D_6__uncultured Chloroflexi bacterium
2e5202c9febc5b65148486edb223eead	621	0.94	D_0__Bacteria;D_1__Bacteroidetes;D_2__Rhodothermia;D_3__Rhodothermales;D_4__Rhodothermaceae;D_5__uncultured;D_6__uncultured bacterium
4c5fcc2c48c10396e71aac6ceedef841	509	0.85	D_0__Bacteria;D_1__Chloroflexi;D_2__Anaerolineae;D_3__SBR1031;D_4__A4b;D_5__uncultured Chloroflexus sp.;D_6__uncultured Chloroflexus sp.
343128e8b51345ce61bcaabb1a70f898	453	1.00	D_0__Bacteria;D_1__Proteobacteria;D_2__Alphaproteobacteria;D_3__Puniceispirillales;Ambiguous_taxa;Ambiguous_taxa;Ambiguous_taxa
679aee879fd5868fad6e4b808c09c6d5	392	1.00	D_0__Bacteria;D_1__Poribacteria;Ambiguous_taxa;Ambiguous_taxa;Ambiguous_taxa;Ambiguous_taxa;Ambiguous_taxa
da12db4ab27cd8061c031ebcca1f0003	315	1.00	D_0__Bacteria;D_1__Proteobacteria;D_2__Gammaproteobacteria;D_3__KI89A clade;D_4__uncultured bacterium;D_5__uncultured bacterium;D_6__uncultured bacterium

025b4605187229af9fe5cfc3d92720c6	314	1.00	D_0__Bacteria;D_1__Proteobacteria;D_2__Gammaproteobacteria;D_3__KI89A clade;D_4__uncultured bacterium;D_5__uncultured bacterium;D_6__uncultured bacterium
408b59118f65314c1c90b2b5f6a55dbb	291	1.00	D_0__Bacteria;D_1__Acidobacteria;D_2__Thermoanaerobaculia;D_3__Thermoanaerobaculales;D_4__Thermoanaerobaculaceae;D_5__Subgroup 10
73bd7535b17807cd48e6f4293c75afd7	247	0.97	D_0__Bacteria;D_1__Proteobacteria;D_2__Alphaproteobacteria;D_3__uncultured;D_4__uncultured bacterium;D_5__uncultured bacterium;D_6__uncultured bacterium
4d58056cc0ef38da08ac85896bfcbb22	241	1.00	D_0__Bacteria;D_1__Poribacteria;D_2__Candidatus Poribacteria bacterium WGA-4C;D_3__Candidatus Poribacteria bacterium WGA-4C;D_4__Candidatus Poribacteria bacterium WGA-4C;D_5__Candidatus Poribacteria bacterium WGA-4C;D_6__Candidatus Poribacteria bacterium WGA-4C
592a928e5dcc906cce2d487a01d84c9f	176	1.00	D_0__Bacteria;D_1__Proteobacteria;D_2__Alphaproteobacteria;D_3__Rhodobacterales;D_4__Rhodobacteraceae;D_5__Albidovulum;D_6__uncultured bacterium
f97b500e62ccb9b4930eaada5f6847bf	166	0.98	D_0__Bacteria;D_1__Proteobacteria;D_2__Alphaproteobacteria;D_3__uncultured
970871b60c7de2f701cbe085d28a7091	162	1.00	D_0__Bacteria;D_1__Proteobacteria;D_2__Gammaproteobacteria;D_3__KI89A clade;D_4__uncultured bacterium;D_5__uncultured bacterium;D_6__uncultured bacterium
cbe097b2843bf71d7e66ed46b947a58a	158	0.75	D_0__Bacteria;D_1__Proteobacteria;D_2__Gammaproteobacteria;D_3__HOC36;Ambiguous_taxa;Ambiguous_taxa;Ambiguous_taxa
e8753c670d63c0001e8a4c2d232451c4	150	1.00	D_0__Bacteria;D_1__Acidobacteria;D_2__Acidobacteriia;D_3__Solibacterales;D_4__Solibacteraceae (Subgroup 3);D_5__PAUC26f;D_6__uncultured bacterium
f41351edafdb78975b82fe1c05e0870f	150	0.92	D_0__Bacteria;D_1__Proteobacteria;D_2__Gammaproteobacteria;D_3__Nitrosococcales;D_4__Nitrosococcaceae;D_5__AqS1;Ambiguous_taxa
2194a824269dfed37ff5873706fe5fcc	147	0.97	D_0__Bacteria;D_1__Actinobacteria;D_2__Acidimicrobiia;D_3__Microtrichales;D_4__Microtrichaceae;D_5__Sva0996 marine group;D_6__actinobacterium MSI70
5ea65b40ea4782c6452ea8b37d81e4c8	147	1.00	D_0__Bacteria;D_1__Proteobacteria;D_2__Alphaproteobacteria;D_3__Puniceispirillales;Ambiguous_taxa;Ambiguous_taxa;Ambiguous_taxa
0f4a02277d3363604c1f95c85fe1e07e	143	1.00	D_0__Bacteria;D_1__Chloroflexi;D_2__Dehalococcoidia;D_3__SAR202 clade

fb2a4a62f6e91244f0d2ba059167b492	134	0.95	D_0__Bacteria;D_1__Actinobacteria;D_2__Acidimicrobiia;D_3__Actinomarinales;D_4__uncultured;Ambiguous_taxa;Ambiguous_taxa
f8c6265d9164c3ae9b8fed342747cfb5	133	1.00	D_0__Bacteria;D_1__Chloroflexi;D_2__TK30
5218f08d0699090d480112f7a2e42b04	126	0.97	D_0__Bacteria;D_1__Chloroflexi;D_2__Anaerolineae;D_3__Caldilineales;D_4__Caldilineaceae;D_5__uncultured;D_6__uncultured Chloroflexi bacterium
21b7b83ac78240bcc7b31478f75b30b1	125	1.00	D_0__Bacteria;D_1__Chloroflexi;D_2__TK17
dbb6f0662a546fe19be713d2bd88eefd	122	1.00	D_0__Bacteria;D_1__Proteobacteria;D_2__Gammaproteobacteria;D_3__Oceanospirillales;D_4__Endozoicomonadaceae;D_5__Endozoicomonas
8af02bc807f04196cebfefd60b7b1ceb	119	0.80	D_0__Bacteria;D_1__Actinobacteria;D_2__Acidimicrobiia;D_3__Microtrichales;D_4__Microtrichaceae;D_5__Sva0996 marine group;D_6__actinobacterium MSI70
c1e4e5423b9dc8dc5e1dbe2699b14275	112	1.00	D_0__Bacteria;D_1__Chloroflexi;D_2__Anaerolineae;D_3__SBR1031;D_4__A4b
d110df1d18f2eb83ddd4d36f1119a0ea	107	1.00	D_0__Bacteria;D_1__Proteobacteria;D_2__Deltaproteobacteria;D_3__Myxococcales;D_4__bacteriap25
6c74b815c61f0f1c4d36ca2b589303b2	103	1.00	D_0__Bacteria;D_1__Proteobacteria;D_2__Gammaproteobacteria;D_3__UBA10353 marine group
cb3903e58b3e61c940045cf599b3812c	98	0.87	D_0__Bacteria;D_1__Gemmatimonadetes;D_2__BD2-11 terrestrial group;D_3__uncultured bacterium;D_4__uncultured bacterium;D_5__uncultured bacterium;D_6__uncultured bacterium
2d44639ad050a6d3c4ecef9eeb3d7f42	96	1.00	D_0__Bacteria;D_1__Acidobacteria;D_2__Subgroup 6
b16b6d1e6551c97518f6def91b1600b3	96	0.87	D_0__Bacteria;D_1__Proteobacteria;D_2__Gammaproteobacteria;D_3__Nitrosococcales;D_4__Nitrosococcaceae;D_5__AqS1;Ambiguous_taxa
c12d9a30bb0798e4a4e346fd83a12e5a	93	1.00	D_0__Bacteria;D_1__Chloroflexi;D_2__Dehalococcoidia;D_3__SAR202 clade
9101a4e6eae37bde17cf64fb7be21246	91	1.00	D_0__Bacteria;D_1__Acidobacteria;D_2__Acidobacteriia;D_3__Solibacterales;D_4__Solibacteraceae (Subgroup 3);D_5__PAUC26f;D_6__uncultured bacterium

63c4e46b237b7825de4785a4e4ffe55a	87	0.97	D_0__Bacteria;D_1__Proteobacteria;D_2__Gammaproteobacteria;D_3__KI89A clade;D_4__uncultured bacterium;D_5__uncultured bacterium;D_6__uncultured bacterium
c5ce5fedffa09328ae705cef668d6285	80	1.00	D_0__Bacteria;D_1__Proteobacteria;D_2__Gammaproteobacteria;D_3__pItb-vmat-80;D_4__uncultured bacterium;D_5__uncultured bacterium;D_6__uncultured bacterium
fbd6446f99ff4514d87375fd7bfed37a	80	0.92	D_0__Bacteria;D_1__Nitrospinae;D_2__P9X2b3D02;D_3__uncultured bacterium;D_4__uncultured bacterium;D_5__uncultured bacterium;D_6__uncultured bacterium
4d2f1794d620881577baf24aaa2fffa2	70	0.83	D_0__Bacteria;D_1__Proteobacteria;D_2__Alphaproteobacteria;D_3__Sneathiellales;D_4__Sneathiellaceae;D_5__AT-s3-44;D_6__uncultured bacterium
9809a78dddfcc4f8fc2edd09e1237651	70	0.97	D_0__Bacteria;D_1__PAUC34f;D_2__uncultured Deferribacteres bacterium;D_3__uncultured Deferribacteres bacterium;D_4__uncultured Deferribacteres bacterium;D_5__uncultured Deferribacteres bacterium;D_6__uncultured Deferribacteres bacterium
3a17a62414627555360e70cb6ae523cc	68	0.94	D_0__Bacteria;D_1__Proteobacteria;D_2__Alphaproteobacteria;D_3__Rhodovibrionales;D_4__Kiloniellaceae;D_5__uncultured;D_6__uncultured bacterium
c0cffc1917fd8a24119321ed9ffd7c80	67	1.00	D_0__Bacteria;D_1__Proteobacteria;D_2__Deltaproteobacteria
7fb620f63b0ba7a2dcca8d3b6b3b7182	66	1.00	D_0__Bacteria;D_1__Proteobacteria;D_2__Gammaproteobacteria;D_3__KI89A clade
92479a958d13d8729846c440e84434a0	66	1.00	D_0__Bacteria;D_1__Chloroflexi;D_2__Dehalococcoidia;D_3__SAR202 clade
f275249eb28723c7dac962d20055208b	58	0.95	D_0__Bacteria;D_1__Acidobacteria;D_2__Subgroup 9;D_3__uncultured bacterium;D_4__uncultured bacterium;D_5__uncultured bacterium;D_6__uncultured bacterium
d2856aadd83fad3297613e2da25a3073	58	0.95	D_0__Bacteria;D_1__Chloroflexi;D_2__Anaerolineae;D_3__SBR1031;D_4__A4b;D_5__uncultured Chloroflexus sp.;D_6__uncultured Chloroflexus sp.
471195b09a0fb63742bebcd7cdbe8873	55	1.00	D_0__Bacteria;D_1__Proteobacteria;D_2__Gammaproteobacteria;D_3__EPR3968-O8a-Bc78;Ambiguous_taxa;Ambiguous_taxa;Ambiguous_taxa
734f25088d2f464de431ff355629ba84	55	1.00	D_0__Bacteria;D_1__Bacteroidetes;D_2__Rhodothermia;D_3__Rhodothermales;D_4__Rhodothermaceae;D_5__uncultured

131f4f18fb16759c2bc2a3b05beaeb30	53	1.00	D_0__Bacteria;D_1__Gemmatimonadetes;D_2__PAUC43f marine benthic group
77e5e068af7ed05154eb464218768112	51	1.00	D_0__Bacteria;D_1__Proteobacteria;D_2__Gammaproteobacteria;D_3__pItb-vmat-80;D_4__uncultured bacterium;D_5__uncultured bacterium;D_6__uncultured bacterium
8d21848ccfc247e9e92028e02e2cfd66	48	1.00	D_0__Bacteria;D_1__Acidobacteria;D_2__Subgroup 6
a0ee66d38228277e4df60a94a3e798ca	41	0.84	D_0__Bacteria;D_1__Proteobacteria;D_2__Alphaproteobacteria;D_3__Rhodobacterales;D_4__Rhodobacteraceae;D_5__Ruegeria;Ambiguous_taxa
c35f759bb339817fdeaf73d965be811e	38	1.00	D_0__Bacteria;D_1__Chloroflexi;D_2__Dehalococcoidia;D_3__SAR202 clade
f0e652807ef1c513c319699ca832a27d	38	0.91	D_0__Bacteria;D_1__Proteobacteria;D_2__Gammaproteobacteria;D_3__JTB23;D_4__uncultured bacterium;D_5__uncultured bacterium;D_6__uncultured bacterium
ab0893b7de88347f92003023342fa769	36	0.92	D_0__Bacteria;D_1__Actinobacteria;D_2__Acidimicrobiia;D_3__Microtrichales;D_4__Microtrichaceae;D_5__Sva0996 marine group;D_6__uncultured actinobacterium
7b2b216db6109b7a7f33f0aceb3a8198	35	0.77	D_0__Bacteria;D_1__Spirochaetes;D_2__Spirochaetia;D_3__Spirochaetales;D_4__Spirochaetaceae;D_5__Spirochaeta 2;Ambiguous_taxa
b226f51c765c28de2675e26f8fa2683a	33	1.00	D_0__Bacteria;D_1__Proteobacteria;D_2__Alphaproteobacteria;D_3__Rhodospirillales;D_4__Magnetospiraceae;D_5__uncultured
3b777c591c656625fd4b3290b5a54e72	31	1.00	D_0__Bacteria;D_1__Proteobacteria;D_2__Gammaproteobacteria;D_3__Oceanospirillales;D_4__Endozoicomonadaceae;D_5__Endozoicomonas
c861054d32d66fb24c1a8fd7a3a96296	29	1.00	D_0__Bacteria;D_1__Gemmatimonadetes;D_2__BD2-11 terrestrial group
9865f63ba4eda05ffd69de6e44c98bdd	28	1.00	D_0__Bacteria;D_1__Proteobacteria;D_2__Alphaproteobacteria;D_3__Puniceispirillales;D_4__EF100-94H03;Ambiguous_taxa;Ambiguous_taxa
e6b60e4d45f0a1e5d3d7e8627ae4707d	28	0.86	D_0__Bacteria;D_1__Chloroflexi;D_2__Dehalococcoidia;D_3__SAR202 clade;D_4__uncultured Chloroflexus sp.;D_5__uncultured Chloroflexus sp.;D_6__uncultured Chloroflexus sp.
ab16214a92b9c6584fc61174c967ba27	26	1.00	D_0__Bacteria;D_1__Acidobacteria;D_2__Subgroup 6

48991897dd1ac07f733f610b4a598314	25	0.84	D_0__Bacteria;D_1__Poribacteria;Ambiguous_taxa;Ambiguous_taxa;Ambiguous_taxa;Ambiguous_taxa;Ambiguous_taxa
151248c7f6fe13adb0253a3281022027	25	0.99	D_0__Bacteria;D_1__Chloroflexi;D_2__Anaerolineae;D_3__SBR1031;D_4__A4b;Ambiguous_taxa;Ambiguous_taxa
074a4c0124e2c764b48955b1c73766a8	25	0.99	D_0__Bacteria;D_1__Proteobacteria;D_2__Gammaproteobacteria;D_3__KI89A clade;D_4__uncultured bacterium;D_5__uncultured bacterium;D_6__uncultured bacterium
1471635f9956b4e9c795e016fe21bb59	24	0.89	D_0__Bacteria;D_1__Proteobacteria;D_2__Gammaproteobacteria;D_3__Oceanospirillales;D_4__Pseudohongiellaceae;D_5__Pseudohongiella;D_6__uncultured bacterium
7b44eb00032d861a29316601d8131106	22	0.98	D_0__Bacteria;D_1__Proteobacteria;D_2__Gammaproteobacteria;D_3__JTB23;D_4__uncultured bacterium;D_5__uncultured bacterium;D_6__uncultured bacterium
8fa000503813b3213d947bad474c85fd	19	0.86	D_0__Bacteria;D_1__Proteobacteria;D_2__Alphaproteobacteria;D_3__Sneathiellales;D_4__Sneathiellaceae;D_5__AT-s3-44;D_6__uncultured bacterium
9eabf5cf53b386e304e8f92ddc45fdfa	19	0.97	D_0__Bacteria;D_1__Actinobacteria;D_2__Acidimicrobiia;D_3__Actinomarinales;D_4__uncultured;D_5__uncultured actinobacterium;D_6__uncultured actinobacterium
d936171702c1fbc2c09c88b89f82b811	18	0.76	D_0__Bacteria;D_1__Actinobacteria;D_2__Acidimicrobiia;D_3__Microtrichales;D_4__Microtrichaceae;D_5__Sva0996 marine group;Ambiguous_taxa
b3f6b713da93880b1dc8760e4a96ec84	18	0.96	D_0__Bacteria;D_1__Proteobacteria;D_2__Gammaproteobacteria;D_3__Alteromonadales;D_4__Shewanellaceae;D_5__Shewanella;Ambiguous_taxa
03241d598a0425ae03563b29eb4cf618	17	0.89	D_0__Bacteria;D_1__Proteobacteria;D_2__Alphaproteobacteria;D_3__Rhodobacterales;D_4__Rhodobacteraceae;D_5__uncultured;D_6__uncultured Rhodobacter sp.
b0f6f40c85c4c5b4e220196f5dc9a4d4	17	0.90	D_0__Bacteria;D_1__Spirochaetes;D_2__Spirochaetia;D_3__Spirochaetales;D_4__Spirochaetaceae;D_5__Spirochaeta 2;D_6__uncultured Spirochaetales bacterium
a93a23c7ffa1eca8f7127e83599dc30e	17	0.90	D_0__Bacteria;D_1__Proteobacteria;D_2__Deltaproteobacteria;D_3__Myxococcales;D_4__bacteriap25;Ambiguous_taxa;Ambiguous_taxa
5fe55b01e9730b483cf5163eb019384e	14	0.78	D_0__Bacteria;D_1__Chloroflexi;D_2__JG30-KF-CM66;Ambiguous_taxa;Ambiguous_taxa;Ambiguous_taxa;Ambiguous_taxa

3bb53e6579e782a8fccf15872926a521	13	0.91	D_0__Bacteria;D_1__Proteobacteria;D_2__Alphaproteobacteria;D_3__Sneathiellales;D_4__Sneathiellaceae;D_5__AT-s3-44;D_6__uncultured bacterium
fb8e95afe2f66823cc872e25bdc98947	13	0.88	D_0__Bacteria;D_1__Proteobacteria;D_2__Gammaproteobacteria;D_3__Nitrosococcales;D_4__Nitrosococcaceae;D_5__AqS1;D_6__uncultured gamma proteobacterium
e1428e39c1d30e45826bc6cadc71b8c9	12	1.00	D_0__Bacteria;D_1__Chloroflexi;D_2__Dehalococcoidia;D_3__SAR202 clade
86b607314dc1d54080da5de2697058ba	12	0.84	D_0__Bacteria;D_1__Acidobacteria;D_2__Subgroup 6;D_3__uncultured bacterium;D_4__uncultured bacterium;D_5__uncultured bacterium;D_6__uncultured bacterium
d98ea7329c16ba02e782d47c4083cc11	11	0.99	D_0__Bacteria;D_1__Acidobacteria;D_2__Subgroup 11;Ambiguous_taxa;Ambiguous_taxa;Ambiguous_taxa;Ambiguous_taxa
1decc14b2512caa809b388b729c1087f	11	0.97	D_0__Bacteria;D_1__Entotheonellaeota;D_2__Entotheonellia;D_3__Entotheonellales;D_4__Entotheonellaceae;D_5__uncultured delta proteobacterium;D_6__uncultured delta proteobacterium
4152a70d56bcb15ea2f7fd2477c387c8	11	1.00	D_0__Bacteria;D_1__Proteobacteria;D_2__Gammaproteobacteria;D_3__Oceanospirillales;D_4__Endozoicomonadaceae;D_5__Endozoicomonas;D_6__uncultured gamma proteobacterium
d644029ea9c1776aef75cb247dbd33e6	10	1.00	D_0__Bacteria;D_1__Proteobacteria;D_2__Gammaproteobacteria;D_3__Steroidobacterales;D_4__Woeseiaceae;D_5__JTB255 marine benthic group;Ambiguous_taxa
d1afa73745b70ca14dd9335e7a8499a6	10	0.86	D_0__Bacteria;D_1__Proteobacteria;D_2__Gammaproteobacteria;D_3__Nitrosococcales;D_4__Nitrosococcaceae;D_5__AqS1;D_6__uncultured gamma proteobacterium
ec2a683cc530e945270aeafa76ccc777	10	0.99	D_0__Bacteria;D_1__Entotheonellaeota;D_2__Entotheonellia;D_3__Entotheonellales;D_4__Entotheonellaceae;D_5__uncultured delta proteobacterium;D_6__uncultured delta proteobacterium
2e1f87bd1c8c5a458ee068f1f3b424c5	9	1.00	D_0__Bacteria;D_1__Proteobacteria;D_2__Alphaproteobacteria
40ab3484850b34bb4889e28325284b1a	9	0.95	D_0__Bacteria;D_1__Proteobacteria;D_2__Gammaproteobacteria;D_3__Nitrosococcales;D_4__Nitrosococcaceae;D_5__AqS1;Ambiguous_taxa
1b292f6ddc75e6694b00ab437a68137f	7	1.00	D_0__Bacteria;D_1__Chloroflexi;D_2__Dehalococcoidia;D_3__SAR202 clade

9335449e382d8ed87b56f0ca64eb79fc	7	0.83	D_0__Bacteria;D_1__Chloroflexi;D_2__Dehalococcoidia;D_3__SAR202 clade;D_4__uncultured Chloroflexi bacterium;D_5__uncultured Chloroflexi bacterium;D_6__uncultured Chloroflexi bacterium
fe1a9e0e68cbd9d447ebb18e4a736a41	6	0.98	D_0__Bacteria;D_1__Cyanobacteria;D_2__Oxyphotobacteria;D_3__Synechococcales;D_4__Cyanobiaceae;D_5__Cyanobium PCC-6307;Ambiguous_taxa
74fb75f885374e290262a669196ea182	6	0.78	D_0__Bacteria;D_1__AncK6;D_2__uncultured delta proteobacterium;D_3__uncultured delta proteobacterium;D_4__uncultured delta proteobacterium;D_5__uncultured delta proteobacterium;D_6__uncultured delta proteobacterium
f0e8c772641da0f536d15d18db41bf7e	6	0.92	D_0__Bacteria;D_1__Poribacteria;D_2__uncultured Clostridium sp.;D_3__uncultured Clostridium sp.;D_4__uncultured Clostridium sp.;D_5__uncultured Clostridium sp.;D_6__uncultured Clostridium sp.
e069ea44f2497c46fbd26a5a62407cee	6	0.92	D_0__Bacteria;D_1__Proteobacteria;D_2__Gammaproteobacteria;D_3__Nitrosococcales;D_4__Nitrosococcaceae;D_5__FS142-36B-02;Ambiguous_taxa
2556ff27c8b418b9c038f90ca8eaa7f3	4	0.75	D_0__Bacteria;D_1__Proteobacteria;D_2__Gammaproteobacteria;D_3__OM182 clade;D_4__uncultured gamma proteobacterium;D_5__uncultured gamma proteobacterium;D_6__uncultured gamma proteobacterium
6b4cfcc8dcf18b48a27f6bb034491b08	4	1.00	D_0__Bacteria;D_1__Proteobacteria;D_2__Deltaproteobacteria;D_3__Bdellovibrionales;D_4__Bacteriovoracaceae
4431f1a5d086ff8bc441a897db606772	4	1.00	D_0__Bacteria;D_1__Proteobacteria;D_2__Gammaproteobacteria;D_3__Oceanospirillales;D_4__Endozoicomonadaceae;D_5__Endozoicomonas;D_6__Endozoicomonas sp. Ez302
436f5b7fd4f7b51ea25fadf2ece211f0	4	1.00	D_0__Bacteria;D_1__Proteobacteria;D_2__Alphaproteobacteria;D_3__Rhodobacterales;D_4__Rhodobacteraceae;D_5__Albidovulum
52128ba808ec7031c608ab02a9b9b27a	4	1.00	D_0__Bacteria;D_1__Proteobacteria;D_2__Gammaproteobacteria
18ddb8d7d5b6253e9d0dc234dc679e8a	3	0.94	D_0__Bacteria;D_1__Proteobacteria;D_2__Alphaproteobacteria;D_3__Rhodobacterales;D_4__Rhodobacteraceae;D_5__uncultured
031a9141a848332f51ec34297ba293f9	3	0.96	D_0__Bacteria;D_1__Proteobacteria;D_2__Gammaproteobacteria;D_3__Vibrionales;D_4__Vibrionaceae;D_5__Vibrio
738ca63a3916b750ba4e60642673974c	2	0.99	D_0__Bacteria;D_1__Proteobacteria;D_2__Deltaproteobacteria;D_3__Bdellovibrionales;D_4__Bdellovibrionaceae;D_5__Bdellovibrio;D_6__uncultured delta proteobacterium

26064c129cc1e1099ae127443533c90b	2	1.00	D_0__Bacteria;D_1__Acidobacteria;D_2__Subgroup 6
7e15b663975047e0a56ce1e3d4acfcd6	2	0.99	D_0__Bacteria;D_1__Verrucomicrobia;D_2__Verrucomicrobiae;D_3__Verrucomicrobiales;D_4__Rubritaleaceae;D_5__Roseibacillus;D_6__uncultured marine bacterium
a7f17a599ee8c38202eeea499deabc39	1	1.00	D_0__Bacteria;D_1__Proteobacteria;D_2__Gammaproteobacteria;D_3__Nitrosococcales;D_4__Nitrosococcaceae;D_5__AqS1
f2d036ddf6a664f2126db27875360b00	1	1.00	D_0__Bacteria;D_1__Chloroflexi;D_2__TK17;D_3__uncultured bacterium;D_4__uncultured bacterium;D_5__uncultured bacterium;D_6__uncultured bacterium

Table S7. ASVs (109) from *Oceanapia* cf. *perforata* (Sarà, 1960) with percentage of confidence ≥ 75%.

Feature ID	Number of sequences	Confidence	Taxon
34073cbf11ad1feb5bc31ff5dcff3cff	1621	0.96	D_0__Bacteria;D_1__Verrucomicrobia;D_2__Verrucomicrobiae;D_3__Pedosphaerales;D_4__Pedosphaeraceae;D_5__ uncultured bacterium;D_6__uncultured bacterium
031b0a1b024a8ca1fd6c7279476c6386	1255	1.00	D_0__Bacteria;D_1__Poribacteria;D_2__uncultured Clostridium sp.;D_3__uncultured Clostridium sp.;D_4__uncultured Clostridium sp.;D_5__uncultured Clostridium sp.;D_6__uncultured Clostridium sp.
209bc93b993e041863cd6c878cb0e9ee	1092	1.00	D_0__Bacteria;D_1__Proteobacteria;D_2__Deltaproteobacteria;D_3__Oligoflexales;D_4__Oligoflexaceae;D_5__uncul tured;D_6__uncultured delta proteobacterium
e23c156642078fb48f89bc01faadac18	966	0.99	D_0__Bacteria;D_1__Poribacteria;Ambiguous_taxa;Ambiguous_taxa;Ambiguous_taxa;Ambiguous_taxa;Ambiguous_ta xa
130f11b7a1d6a93130f2bdab144bd474	958	0.78	D_0__Bacteria;D_1__Nitrospirae;D_2__Nitrospira;D_3__Nitrospirales;D_4__Nitrospiraceae;D_5__Nitrospira;D_6__u ncultured Nitrospirae bacterium
832aba4255059f1034a1d584d111257d	945	1.00	D_0__Bacteria
33308451bcbd23d19c9305d793345001	850	1.00	D_0__Bacteria;D_1__Chloroflexi;D_2__Dehalococcoidia;D_3__SAR202 clade
4a246419b16b57fd95824fda89b86361	695	0.97	D_0__Bacteria;D_1__Chloroflexi;D_2__Anaerolineae;D_3__Caldilineales;D_4__Caldilineaceae;D_5__uncultured;D_6 __uncultured Chloroflexi bacterium
8e9712816ef9e61accc05bb2fd74a2d6	638	1.00	D_0__Bacteria;D_1__Acidobacteria;D_2__Thermoanaerobaculia;D_3__Thermoanaerobaculales;D_4__Thermoanaerob aculaceae;D_5__Subgroup 10
3a6c8091f03a0848963dc67302b87f9c	557	0.78	D_0__Bacteria;D_1__Chloroflexi;D_2__Dehalococcoidia;D_3__SAR202 clade;D_4__uncultured Chloroflexi bacterium;D_5__uncultured Chloroflexi bacterium;D_6__uncultured Chloroflexi bacterium
f34d1fbf32dc7b137af8bc422a131bb5	478	1.00	D_0__Bacteria;D_1__Chloroflexi;D_2__Dehalococcoidia;D_3__SAR202 clade
be3c73452201b90ef6d4c0ede59f7827	442	0.88	D_0__Bacteria;D_1__Actinobacteria;D_2__Acidimicrobiia;D_3__Microtrichales;D_4__Microtrichaceae;D_5__Sva099 6 marine group;D_6__uncultured actinobacterium

219b06bc78989f181bccf5fc43a503dc	409	0.86	D_0__Bacteria;D_1__Gemmatimonadetes;D_2__BD2-11 terrestrial group;D_3__uncultured bacterium;D_4__uncultured bacterium;D_5__uncultured bacterium;D_6__uncultured bacterium
44ee7c17ead31b2c7686dd9c3517e8b6	391	1.00	D_0__Bacteria;D_1__Proteobacteria;D_2__Deltaproteobacteria;D_3__Myxococcales;D_4__bacteriap25
97510843a4efb9f9accd20d98d915d8e	360	0.97	D_0__Bacteria;D_1__Dadabacteria;D_2__Dadabacteriia;D_3__Dadabacteriales;D_4__uncultured delta proteobacterium;D_5__uncultured delta proteobacterium;D_6__uncultured delta proteobacterium
debecd21c44c833dbbe9f24e14e36f3c	357	0.77	D_0__Bacteria;D_1__Actinobacteria;D_2__Acidimicrobiia;D_3__Microtrichales;D_4__Microtrichaceae;D_5__Sva0996 marine group;D_6__uncultured actinobacterium
49fdfc324b7540e940c20f43b8ffe776	353	0.86	D_0__Bacteria;D_1__Gemmatimonadetes;D_2__BD2-11 terrestrial group;D_3__uncultured bacterium;D_4__uncultured bacterium;D_5__uncultured bacterium;D_6__uncultured bacterium
02c39114d42bb476275b760a30b42e06	352	1.00	D_0__Bacteria;D_1__Chloroflexi;D_2__Dehalococcoidia;D_3__SAR202 clade
dde8c21e8f628150294efd6c43091af7	351	0.86	D_0__Bacteria;D_1__Proteobacteria;D_2__Alphaproteobacteria;D_3__Rhodobacterales;D_4__Rhodobacteraceae;D_5__Albidovulum;D_6__uncultured alpha proteobacterium
36bd2e3e51937d0ddd197a01b9a584a8	348	0.99	D_0__Bacteria;D_1__Proteobacteria;D_2__Alphaproteobacteria;D_3__uncultured;D_4__uncultured bacterium;D_5__uncultured bacterium;D_6__uncultured bacterium
e2202f2d3f81df39ba1d35e95ef97d63	348	0.93	D_0__Bacteria;D_1__Proteobacteria;D_2__Gammaproteobacteria;D_3__KI89A clade
c56b28bebd34ff3f8af4a06d18a05688	315	0.97	D_0__Bacteria;D_1__Chloroflexi;D_2__Anaerolineae;D_3__SBR1031;D_4__A4b;D_5__uncultured Chloroflexus sp.;D_6__uncultured Chloroflexus sp.
62b37697acf744151ceea8d1b7cba4d4	301	1.00	D_0__Bacteria;D_1__Chloroflexi;D_2__TK17
0b8e939eeda34fdab37888c1b96dd0d6	266	0.85	D_0__Bacteria;D_1__Proteobacteria;D_2__Gammaproteobacteria;D_3__UBA10353 marine group;Ambiguous_taxa;Ambiguous_taxa;Ambiguous_taxa
d1a483a03af61e579431dad693505ebe	251	0.83	D_0__Archaea;D_1__Thaumarchaeota;D_2__Nitrososphaeria;D_3__Nitrosopumilales;D_4__Nitrosopumilaceae;D_5__Candidatus Nitrosopumilus;Ambiguous_taxa

b3f43baa746efedf73e3f13961c6d66e	248	0.95	D_0__Bacteria;D_1__PAUC34f;D_2__uncultured Deferribacteres bacterium;D_3__uncultured Deferribacteres bacterium;D_4__uncultured Deferribacteres bacterium;D_5__uncultured Deferribacteres bacterium;D_6__uncultured Deferribacteres bacterium
fef8fb776b2bf0ff175283e2fc6c38f0	232	1.00	D_0__Bacteria;D_1__Proteobacteria;D_2__Gammaproteobacteria;D_3__Nitrosococcales;D_4__Nitrosococcaceae;D_5__AqS1
343128e8b51345ce61bcaabb1a70f898	229	1.00	D_0__Bacteria;D_1__Proteobacteria;D_2__Alphaproteobacteria;D_3__Puniceispirillales;Ambiguous_taxa;Ambiguous_taxa;Ambiguous_taxa
ead66d8f28b550e125ca45457e1358cf	220	1.00	D_0__Bacteria;D_1__Proteobacteria;D_2__Gammaproteobacteria;D_3__Steroidobacterales;D_4__Woeseiaceae;D_5__JTB255 marine benthic group;Ambiguous_taxa
49afe14763e9edc14cbfd5ff68a8687d	209	1.00	D_0__Bacteria;D_1__Proteobacteria;D_2__Alphaproteobacteria;D_3__Puniceispirillales;D_4__EF100-94H03;Ambiguous_taxa;Ambiguous_taxa
ce15d545839045d8e744da9ee5e044ea	198	1.00	D_0__Bacteria;D_1__Chloroflexi;D_2__Dehalococcoidia;D_3__SAR202 clade
36503692645bcbacd6f0bf85ac7aabf1	196	0.89	D_0__Bacteria;D_1__Proteobacteria;D_2__Gammaproteobacteria;D_3__Nitrosococcales;D_4__Nitrosococcaceae;D_5__AqS1;Ambiguous_taxa
356a97017d5a7ceb8ee93fa1f50dee9b	189	1.00	D_0__Bacteria;D_1__Acidobacteria;D_2__Acidobacteriia;D_3__Solibacterales;D_4__Solibacteraceae (Subgroup 3);D_5__PAUC26f;D_6__uncultured bacterium
3c7fe7b02cffe834b2230f4b3ad637ed	170	1.00	D_0__Bacteria;D_1__Chloroflexi;D_2__Dehalococcoidia;D_3__SAR202 clade
d3ed017adb739c6464776110c93b40ab	163	0.94	D_0__Bacteria;D_1__Proteobacteria;D_2__Deltaproteobacteria;D_3__Myxococcales;D_4__bacteriap25;Ambiguous_taxa;Ambiguous_taxa
89f5f7693af5a37d6e4751bb89e31eb5	153	0.90	D_0__Bacteria;D_1__Nitrospinae;D_2__P9X2b3D02;D_3__uncultured bacterium;D_4__uncultured bacterium;D_5__uncultured bacterium;D_6__uncultured bacterium
834de45f208d6672da4f42b6cb122d33	132	0.89	D_0__Bacteria;D_1__Chloroflexi;D_2__Dehalococcoidia;D_3__S085;Ambiguous_taxa;Ambiguous_taxa;Ambiguous_taxa
4d2f1794d620881577baf24aaa2fffa2	126	0.83	D_0__Bacteria;D_1__Proteobacteria;D_2__Alphaproteobacteria;D_3__Sneathiellales;D_4__Sneathiellaceae;D_5__AT-s3-44;D_6__uncultured bacterium

6fd8315d77d6e5bfa37bd9dc27e4e644	122	1.00	D_0__Bacteria;D_1__Proteobacteria;D_2__Gammaproteobacteria;D_3__Betaproteobacteriales;D_4__EC94;D_5__uncultured bacterium;D_6__uncultured bacterium
8d5d7a29ea6f67ba175662165f080470	116	1.00	D_0__Bacteria;D_1__Gemmatimonadetes;D_2__BD2-11 terrestrial group
588632a54b15a0be73a6de0f1d4c77e4	107	0.97	D_0__Bacteria;D_1__Proteobacteria;D_2__Alphaproteobacteria;D_3__uncultured
2ad1f6cc443b3f725867749cf8fa12a5	103	1.00	D_0__Bacteria;D_1__Proteobacteria;D_2__Gammaproteobacteria;D_3__EPR3968-O8a-Bc78
440372014bb52e796573e652206cd42b	102	0.95	D_0__Bacteria;D_1__PAUC34f;Ambiguous_taxa;Ambiguous_taxa;Ambiguous_taxa;Ambiguous_taxa;Ambiguous_taxa
583304ca9d9badfebf11ec24dda8a067	100	1.00	D_0__Bacteria;D_1__Proteobacteria;D_2__Gammaproteobacteria;D_3__KI89A clade;D_4__uncultured bacterium;D_5__uncultured bacterium;D_6__uncultured bacterium
9e8608b599258654b6007912cad071b9	97	1.00	D_0__Bacteria;D_1__Proteobacteria;D_2__Gammaproteobacteria;D_3__EPR3968-O8a-Bc78;Ambiguous_taxa;Ambiguous_taxa;Ambiguous_taxa
25c62d31b3dfa461940902ef52071555	90	0.95	D_0__Bacteria;D_1__Acidobacteria;D_2__Subgroup 9;D_3__uncultured bacterium;D_4__uncultured bacterium;D_5__uncultured bacterium;D_6__uncultured bacterium
e8099654bf3162e9495aa54e6e154639	89	1.00	D_0__Bacteria;D_1__Chloroflexi;D_2__Dehalococcoidia;D_3__SAR202 clade
2d44639ad050a6d3c4ecef9eeb3d7f42	87	1.00	D_0__Bacteria;D_1__Acidobacteria;D_2__Subgroup 6
affcc0a65fbd734c32b86850d4bb89f6	85	0.94	D_0__Bacteria;D_1__Chloroflexi;D_2__TK30;D_3__uncultured bacterium;D_4__uncultured bacterium;D_5__uncultured bacterium;D_6__uncultured bacterium
68a906fb2f401e137027dc39117fe4df	83	0.99	D_0__Bacteria;D_1__Actinobacteria;D_2__Acidimicrobiia;D_3__Actinomarinales;D_4__uncultured;D_5__uncultured actinobacterium;D_6__uncultured actinobacterium
f240478b19293bfcedf50f24a864818a	81	0.76	D_0__Bacteria;D_1__Acidobacteria;D_2__Subgroup 6;D_3__uncultured bacterium;D_4__uncultured bacterium;D_5__uncultured bacterium;D_6__uncultured bacterium
cb7f6cd2038a14db8508dde6d2d50353	80	0.96	D_0__Bacteria;D_1__Proteobacteria;D_2__Gammaproteobacteria;D_3__HOC36;Ambiguous_taxa;Ambiguous_taxa;Ambiguous_taxa

58d1cff95c7f207607ef510a0da2c003	77	0.98	D_0__Bacteria;D_1__Bacteroidetes;D_2__Rhodothermia;D_3__Rhodothermales;D_4__Rhodothermaceae;D_5__uncultured;Ambiguous_taxa
68c5af62c1820bd64c00454bece5444b	74	0.93	D_0__Bacteria;D_1__Proteobacteria;D_2__Gammaproteobacteria;D_3__KI89A clade;D_4__uncultured bacterium;D_5__uncultured bacterium;D_6__uncultured bacterium
7fb620f63b0ba7a2dcca8d3b6b3b7182	73	1.00	D_0__Bacteria;D_1__Proteobacteria;D_2__Gammaproteobacteria;D_3__KI89A clade
6e0ed25ea0ab7a0d49bbbbcca0b38cd8	72	1.00	D_0__Bacteria;D_1__Actinobacteria;D_2__Acidimicrobiia;D_3__Microtrichales;D_4__Microtrichaceae;D_5__Sva0996 marine group
d0499bd052e7c7b4fc3d6dde2bf08e70	69	0.99	D_0__Bacteria;D_1__Chloroflexi;D_2__Dehalococcoidia;D_3__SAR202 clade
567f0766f4ca51b2e5e35adc2fe97ef3	65	0.90	D_0__Bacteria;D_1__Proteobacteria;D_2__Alphaproteobacteria;D_3__Rhodobacterales;D_4__Rhodobacteraceae;D_5__uncultured;D_6__uncultured delta proteobacterium
973ba1ba56cb30f60cf662252caccda4	65	0.99	D_0__Bacteria;D_1__AncK6;D_2__uncultured delta proteobacterium;D_3__uncultured delta proteobacterium;D_4__uncultured delta proteobacterium;D_5__uncultured delta proteobacterium;D_6__uncultured delta proteobacterium
0af77a8259690b11d1b645f177114c0d	65	0.99	D_0__Bacteria;D_1__Proteobacteria;D_2__Gammaproteobacteria;D_3__KI89A clade;D_4__uncultured bacterium;D_5__uncultured bacterium;D_6__uncultured bacterium
3690ff2d0fa8d59c19b6b02248133a39	61	0.88	D_0__Bacteria;D_1__Proteobacteria;D_2__Gammaproteobacteria;D_3__JTB23;D_4__uncultured bacterium;D_5__uncultured bacterium;D_6__uncultured bacterium
4f614979c5b2c62fc4e079fd79a77c79	60	0.85	D_0__Bacteria;D_1__Proteobacteria;D_2__Alphaproteobacteria;D_3__Rhodovibrionales;D_4__Kiloniellaceae;D_5__uncultured;D_6__uncultured bacterium
b82c25630bdd7fe7c2c0e6328f424a9a	60	1.00	D_0__Bacteria;D_1__Acidobacteria;D_2__Subgroup 6
2359140ca70cc94837689af7ac8e3344	58	1.00	D_0__Bacteria;D_1__Gemmatimonadetes;D_2__PAUC43f marine benthic group
ebb114f5b3df953d904d3bd0fdc646c6	56	0.95	D_0__Bacteria;D_1__Proteobacteria;D_2__Alphaproteobacteria;D_3__Thalassobaculales;D_4__Nisaeaceae;D_5__OM75 clade;D_6__uncultured alpha proteobacterium

e8f0564b9284abd069a3c9f8f8df94ba	54	1.00	D_0__Bacteria;D_1__Proteobacteria;D_2__Gammaproteobacteria;D_3__Nitrosococcales;D_4__Nitrosococcaceae;D_5__AqS1
28bc00fdcdf29f7af9127fd94a261d28	52	0.95	D_0__Bacteria;D_1__Proteobacteria;D_2__Alphaproteobacteria;D_3__Thalassobaculales;D_4__Nisaeaceae;D_5__OM75 clade;D_6__uncultured alpha proteobacterium
0d319457b4c520ce1e2d4120f0704b17	50	0.95	D_0__Bacteria;D_1__PAUC34f;D_2__uncultured Deferribacteres bacterium;D_3__uncultured Deferribacteres bacterium;D_4__uncultured Deferribacteres bacterium;D_5__uncultured Deferribacteres bacterium;D_6__uncultured Deferribacteres bacterium
6770536470e352f2ac35d2df8381a95f	47	0.99	D_0__Bacteria;D_1__Actinobacteria;D_2__Acidimicrobiia;D_3__Microtrichales;D_4__Microtrichaceae;D_5__Sva0996 marine group
5482cfb4b230acd5e15f63d13a932d5a	46	1.00	D_0__Bacteria;D_1__Verrucomicrobia;D_2__Verrucomicrobiae;D_3__Opitutales;D_4__Puniceicoccaceae;D_5__Cerasicoccus;Ambiguous_taxa
ac1f5354b6a7d47a9e17022a1b7db86e	44	0.92	D_0__Bacteria;D_1__Proteobacteria;D_2__Gammaproteobacteria;D_3__Oceanospirillales;D_4__Pseudohongiellaceae;D_5__Pseudohongiella;D_6__uncultured bacterium
ae9bc21b2b4e58798c9cd2ebfa423ad9	43	0.98	D_0__Bacteria;D_1__Proteobacteria;D_2__Gammaproteobacteria;D_3__JTB23;D_4__uncultured bacterium;D_5__uncultured bacterium;D_6__uncultured bacterium
4d1dd210738c03559e82971f7a94797a	41	0.95	D_0__Bacteria;D_1__Proteobacteria;D_2__Gammaproteobacteria;D_3__JTB23;D_4__uncultured bacterium;D_5__uncultured bacterium;D_6__uncultured bacterium
c83f64b45e5d1f860a87c4754b35a98b	34	1.00	D_0__Bacteria;D_1__Proteobacteria;D_2__Gammaproteobacteria;D_3__KI89A clade;D_4__uncultured bacterium;D_5__uncultured bacterium;D_6__uncultured bacterium
f0924ff24a531fd218df9f334858459b	33	1.00	D_0__Bacteria;D_1__Proteobacteria;D_2__Gammaproteobacteria;D_3__pItb-vmat-80;D_4__uncultured bacterium;D_5__uncultured bacterium;D_6__uncultured bacterium
f77c143f7843bb7a6ddd70e4d9aa1201	33	0.96	D_0__Bacteria;D_1__Proteobacteria;D_2__Alphaproteobacteria;D_3__Rhodobacterales;D_4__Rhodobacteraceae;D_5__Albidovulum;D_6__uncultured bacterium
460ed5507919c714cd9239d35b4092b9	32	1.00	D_0__Bacteria;D_1__Proteobacteria;D_2__Alphaproteobacteria
3c3a5c3d1d0ed8269587fe5e4f8bcffd	28	1.00	D_0__Bacteria;D_1__Proteobacteria;D_2__Gammaproteobacteria;D_3__KI89A clade;D_4__uncultured bacterium;D_5__uncultured bacterium;D_6__uncultured bacterium

880049b3855c97790ef7a70af803ae09	25	1.00	D_0__Bacteria;D_1__Chloroflexi;D_2__Anaerolineae;D_3__SBR1031;D_4__A4b
d21f8c497e52082b92dda1ea6115bff9	25	1.00	D_0__Bacteria;D_1__Proteobacteria;D_2__Alphaproteobacteria;D_3__Rhodobacterales;D_4__Rhodobacteraceae
d1848f208d68fe51ba6d848eef29a78d	24	0.79	D_0__Bacteria;D_1__Proteobacteria;D_2__Gammaproteobacteria;D_3__UBA10353 marine group;Ambiguous_taxa;Ambiguous_taxa;Ambiguous_taxa
494cbbebe12c5615994b23f587419ac6	22	1.00	D_0__Bacteria;D_1__Proteobacteria;D_2__Gammaproteobacteria;D_3__Enterobacteriales;D_4__Enterobacteriaceae
9cd0f666fd184c7ffb5a5815b2fb3e77	21	0.86	D_0__Bacteria;D_1__Proteobacteria;D_2__Gammaproteobacteria;D_3__Arenicellales;D_4__Arenicellaceae;D_5__uncultured;Ambiguous_taxa
678c070c733bf5b8d4c81c106d56e04b	18	0.99	D_0__Bacteria
db279d732a197d24aea6d8a956f18f07	18	0.93	D_0__Bacteria;D_1__Proteobacteria;D_2__Gammaproteobacteria;D_3__Oceanospirillales;D_4__Pseudohongiellaceae;D_5__Pseudohongiella;D_6__uncultured bacterium
91405b14b91dc95506affd822ebae203	12	1.00	D_0__Bacteria;D_1__Firmicutes;D_2__Bacilli;D_3__Bacillales;D_4__Family XII;D_5__Exiguobacterium
7b3c474ef944f9f111d6ade2878e235e	10	1.00	D_0__Bacteria;D_1__Proteobacteria;D_2__Deltaproteobacteria;D_3__Bdellovibrionales;D_4__Bacteriovoracaceae
27c11efdddd1118d5df8c5fc40c77275	9	0.81	D_0__Bacteria;D_1__Gemmatimonadetes;D_2__BD2-11 terrestrial group;D_3__uncultured bacterium;D_4__uncultured bacterium;D_5__uncultured bacterium;D_6__uncultured bacterium
33b51e527d28a0eb619bc28b16c0ce3f	7	0.85	D_0__Bacteria;D_1__Proteobacteria;D_2__Gammaproteobacteria;D_3__Nitrosococcales;D_4__Nitrosococcaceae;D_5__AqS1;D_6__uncultured marine bacterium
e075ba67351a8e898e4a4776557eda58	7	0.98	D_0__Bacteria
5979210de5db54bf822e7613aa2f3816	4	0.92	D_0__Bacteria;D_1__Proteobacteria;D_2__Alphaproteobacteria;D_3__Rhodospirillales;D_4__AEGEAN-169 marine group;D_5__marine metagenome;D_6__marine metagenome
643490d8ae62893a86315255d7d67fa9	4	0.99	D_0__Bacteria;D_1__Entotheonellaeota;D_2__Entotheonellia;D_3__Entotheonellales;D_4__Entotheonellaceae;D_5__uncultured delta proteobacterium;D_6__uncultured delta proteobacterium

a8559b1297c9c522a239b51d569b3b06	4	0.97	D_0__Bacteria;D_1__Bacteroidetes;D_2__Bacteroidia;D_3__Cytophagales
917fd7b0e1db31e2abedb3d0a32b2447	4	1.00	D_0__Bacteria
0c608db1a2bd886227b119134d2b358b	3	1.00	D_0__Bacteria;D_1__Proteobacteria;D_2__Alphaproteobacteria;D_3__Rhodobacterales;D_4__Rhodobacteraceae
56a2cbbb61838f339fbbb9014241e61e	3	1.00	D_0__Bacteria;D_1__Margulisbacteria;D_2__uncultured bacterium;D_3__uncultured bacterium;D_4__uncultured bacterium;D_5__uncultured bacterium;D_6__uncultured bacterium
7241a2f2b631b04aefd0f7df4fea468c	3	1.00	D_0__Bacteria;D_1__Proteobacteria;D_2__Deltaproteobacteria;D_3__Bdellovibrionales;D_4__Bdellovibrionaceae;D_5__Bdellovibrio;D_6__uncultured marine bacterium
f7fd1c239d3771d67cd07cc73df7c0f7	3	1.00	D_0__Bacteria;D_1__Proteobacteria;D_2__Gammaproteobacteria;D_3__Oceanospirillales;D_4__Endozoicomonadaceae;D_5__Endozoicomonas
74f07f90fc19be81e8166f34e2c3eb14	2	0.76	D_0__Bacteria;D_1__Proteobacteria;D_2__Gammaproteobacteria;D_3__OM182 clade;D_4__uncultured gamma proteobacterium;D_5__uncultured gamma proteobacterium;D_6__uncultured gamma proteobacterium

Table S8. ASVs (76) from *Tethya aurantium* with percentage of confidence ≥ 75%.

Feature ID	Number of sequences	Confidence	Taxon
4c02cc1d84ecd795178db19c156b56a6	9734	0.99	D_0__Bacteria
caf7aed20d004064b23e8af02b94865b	1396	1.00	D_0__Bacteria;D_1__Proteobacteria;D_2__Alphaproteobacteria;D_3__Rhodobacterales;D_4__Rhodobacteraceae;D_5__Silicimonas;D_6__uncultured bacterium
4b4e17152f168965e25f265b0389e0c5	1202	0.93	D_0__Bacteria;D_1__Proteobacteria;D_2__Gammaproteobacteria;D_3__OM182 clade;D_4__uncultured gamma proteobacterium;D_5__uncultured gamma proteobacterium;D_6__uncultured gamma proteobacterium
f14e1f5c14c7bf39efbe2572c9fe14f0	661	0.90	D_0__Bacteria;D_1__Proteobacteria;D_2__Gammaproteobacteria;D_3__JTB23;D_4__uncultured bacterium;D_5__uncultured bacterium;D_6__uncultured bacterium
7237fb139821c965bef2d8c2415a0762	470	0.92	D_0__Bacteria;D_1__Proteobacteria;D_2__Gammaproteobacteria;D_3__UBA10353 marine group;D_4__uncultured bacterium;D_5__uncultured bacterium;D_6__uncultured bacterium
82c43af7ffb20f7e70c81e12b3b8f67e	421	0.92	D_0__Bacteria;D_1__Proteobacteria;D_2__Alphaproteobacteria;D_3__uncultured;D_4__uncultured bacterium;D_5__uncultured bacterium;D_6__uncultured bacterium
433059d1ddea3965d69c9dfcea86a38a	416	0.93	D_0__Bacteria;D_1__Actinobacteria;D_2__Acidimicrobiia;D_3__Microtrichales;D_4__Microtrichaceae;D_5__Sva0996 marine group;D_6__uncultured bacterium
61fede81a7e515c0bca2d61febad79b6	349	0.83	D_0__Bacteria;D_1__Dadabacteria;D_2__Dadabacteriia;D_3__Dadabacteriales;D_4__uncultured bacterium;D_5__uncultured bacterium;D_6__uncultured bacterium
334b59b78880bbbb2ed0fe3605ab1dbb	332	1.00	D_0__Bacteria;D_1__Actinobacteria;D_2__Acidimicrobiia;D_3__Microtrichales;D_4__Microtrichaceae;D_5__Sva0996 marine group;D_6__uncultured marine bacterium
10c7d40768de55bc1902fda820167703	237	0.97	D_0__Bacteria;D_1__Proteobacteria;D_2__Alphaproteobacteria;D_3__Puniceispirillales;Ambiguous_taxa;Ambiguous_taxa;Ambiguous_taxa
9fc747e35c014e188432f5ef0c3ffc22	228	0.99	D_0__Bacteria

bd6dc559cd7d4692a53f8ceaaa524574	215	1.00	D_0__Bacteria;D_1__Proteobacteria;D_2__Alphaproteobacteria;D_3__Rhodospirillales;D_4__Magnetospiraceae;D_5__uncultured
577347ac8e6378dcc3e9226a6b41d29d	185	0.89	D_0__Bacteria;D_1__Proteobacteria;D_2__Gammaproteobacteria;D_3__OM182 clade;D_4__uncultured gamma proteobacterium;D_5__uncultured gamma proteobacterium;D_6__uncultured gamma proteobacterium
c41c43bef790592ccf2621a1a5a937f1	182	1.00	D_0__Bacteria;D_1__Proteobacteria;D_2__Deltaproteobacteria;D_3__Bdellovibrionales;D_4__Bdellovibrionaceae;D_5__Bdellovibrio
db69eaa01c979379a6148ad849798dd1	170	1.00	D_0__Bacteria;D_1__Proteobacteria;D_2__Alphaproteobacteria;D_3__Puniceispirillales;D_4__uncultured
77851be65cfa35d152cde8d74ab46d79	165	1.00	D_0__Bacteria;D_1__Proteobacteria;D_2__Gammaproteobacteria
7b5c13d11e2aa7ee1b7487f9cbe647d4	159	0.93	D_0__Bacteria;D_1__Proteobacteria;D_2__Gammaproteobacteria;D_3__HOC36;Ambiguous_taxa;Ambiguous_taxa;Ambiguous_taxa
8854dec42a33112d2ee42e1bd3fdc624	134	1.00	D_0__Bacteria;D_1__Proteobacteria;D_2__Gammaproteobacteria
89c2682b495c78250403a42d864e93ce	118	0.90	D_0__Bacteria;D_1__Proteobacteria;D_2__Alphaproteobacteria;D_3__Rhodobacterales;D_4__Rhodobacteraceae;D_5__uncultured
1c58de505252af4f9b0d67a14d21e35f	111	0.81	D_0__Bacteria;D_1__Proteobacteria;D_2__Alphaproteobacteria;D_3__Rhizobiales;D_4__Rhizobiaceae;D_5__Hoeflea;Ambiguous_taxa
131a4b6919c14d28aa7ea0692294f995	81	0.85	D_0__Bacteria;D_1__Proteobacteria;D_2__Alphaproteobacteria;D_3__uncultured
0bcf43ee7fd5dd6e970ce572b923462b	62	1.00	D_0__Bacteria;D_1__Proteobacteria;D_2__Deltaproteobacteria;D_3__Bdellovibrionales;D_4__Bdellovibrionaceae;D_5__Bdellovibrio
15a9db80494bff62096798114df955e7	53	1.00	D_0__Bacteria;D_1__Proteobacteria;D_2__Alphaproteobacteria;D_3__Rhodobacterales;D_4__Rhodobacteraceae;D_5__Silicimonas;D_6__uncultured bacterium
b5dbb679f4c8bb89e28170f5363506ea	48	0.86	D_0__Bacteria;D_1__Proteobacteria;D_2__Alphaproteobacteria;D_3__Rhodospirillales;D_4__Magnetospiraceae
a1debc1b0ad33d0c355797fc258c5fb8	31	0.81	D_0__Bacteria;D_1__Proteobacteria;D_2__Alphaproteobacteria;D_3__Rhizobiales;D_4__Rhizobiaceae;D_5__Hoeflea;Ambiguous_taxa

74cd885371c9472bc6d7fcdfe887ca9c	22	0.78	D_0__Bacteria;D_1__Proteobacteria;D_2__Alphaproteobacteria;D_3__Rhizobiales;D_4__Rhizobiaceae;D_5__Hoeflea; Ambiguous_taxa
917a4c3728db7460af3ad1ba5101bd54	20	0.86	D_0__Bacteria;D_1__Spirochaetes;D_2__Spirochaetia;D_3__Spirochaetales;D_4__Spirochaetaceae;D_5__Salinispira; D_6__uncultured marine bacterium
49fecf1b8717a59c794ac905f91e6ea9	20	1.00	D_0__Bacteria;D_1__Proteobacteria;D_2__Gammaproteobacteria
948c8f33f9c2a4bb02c137993f3210b0	17	0.96	D_0__Bacteria;D_1__Bacteroidetes;D_2__Bacteroidia;D_3__Cytophagales;D_4__Cyclobacteriaceae;D_5__uncultured; D_6__uncultured bacterium
6fe71a308e905e33340c4c880954098b	17	0.94	D_0__Bacteria;D_1__Proteobacteria;D_2__Gammaproteobacteria;D_3__pItb-vmat-80;Ambiguous_taxa;Ambiguous_taxa;Ambiguous_taxa
cdbc38c2625d0d3651377762d1730a42	17	0.82	D_0__Bacteria;D_1__Nitrospirae;D_2__Nitrospira;D_3__Nitrospirales;D_4__Nitrospiraceae;D_5__Nitrospira;Ambiguous_taxa
ff19e692223fd96bd98dc25e10e09c97	17	0.95	D_0__Bacteria;D_1__Nitrospirae;D_2__Nitrospira;D_3__Nitrospirales;D_4__Nitrospiraceae;D_5__Nitrospira;Ambiguous_taxa
d01dd12718a40625034b398db0c7fbee	16	0.99	D_0__Bacteria;D_1__Proteobacteria;D_2__Alphaproteobacteria;D_3__Caulobacterales;D_4__Hyphomonadaceae;D_5__uncultured
a539547ada37a4b1fe25d6446cf5c63d	14	0.96	D_0__Bacteria;D_1__Proteobacteria;D_2__Deltaproteobacteria;D_3__Bdellovibrionales;D_4__Bdellovibrionaceae;D_5__Bdellovibrio;D_6__uncultured bacterium
0c414b9be811bf3974b4f0aa6ae4653d	14	1.00	D_0__Bacteria;D_1__Proteobacteria;D_2__Gammaproteobacteria
fa3590e0429d37c0005d39200cb1ca51	12	1.00	D_0__Archaea;D_1__Thaumarchaeota;D_2__Nitrososphaeria;D_3__Nitrosopumilales;D_4__Nitrosopumilaceae
be9afc7b0890edbcc4b2862b36ea9198	11	0.97	D_0__Bacteria;D_1__Spirochaetes;D_2__Leptospirae;D_3__Leptospirales;D_4__Leptospiraceae;D_5__Turneriella;D_6__uncultured bacterium
790740c1a5d20296264acde32d2549a1	10	1.00	D_0__Bacteria;D_1__Planctomycetes;D_2__Planctomycetacia;D_3__Pirellulales;D_4__Pirellulaceae;D_5__Blastopirellula

b89a9654c927675934d821ebff6d62a3	9	1.00	D_0__Bacteria;D_1__Proteobacteria;D_2__Alphaproteobacteria;D_3__Rhizobiales;D_4__Hyphomicrobiaceae;D_5__Filomicrobium
5b29af7e3476e9f979d9e01e37fcf94f	8	0.99	D_0__Bacteria;D_1__Proteobacteria;D_2__Deltaproteobacteria;D_3__Bdellovibrionales;D_4__Bdellovibrionaceae;D_5__Bdellovibrio;D_6__uncultured delta proteobacterium
b547ab6796e124237d3edd3aa3db2173	7	0.96	D_0__Bacteria;D_1__Spirochaetes;D_2__Spirochaetia;D_3__Spirochaetales;D_4__Spirochaetaceae;D_5__uncultured;D_6__uncultured organism
c398cc110757bce847798a265c16f6d2	6	0.97	D_0__Bacteria;D_1__Proteobacteria;D_2__Gammaproteobacteria;D_3__Oceanospirillales;D_4__Endozoicomonadaceae;D_5__Endozoicomonas;D_6__uncultured bacterium
b6442b3865eb8747ac148921fd7036e1	6	1.00	D_0__Bacteria;D_1__Proteobacteria;D_2__Gammaproteobacteria;D_3__Oceanospirillales;D_4__Endozoicomonadaceae;D_5__Endozoicomonas;Ambiguous_taxa
2bc1fa05e8dc3161efcd1dbfd7832edd	5	0.97	D_0__Bacteria;D_1__Proteobacteria;D_2__Alphaproteobacteria;D_3__uncultured;D_4__uncultured alpha proteobacterium;D_5__uncultured alpha proteobacterium;D_6__uncultured alpha proteobacterium
2b4a0fe55fbc777744c12f982a4688b6	4	1.00	D_0__Bacteria;D_1__Proteobacteria;D_2__Gammaproteobacteria;D_3__SAR86 clade
2f4481679ee50feb2b6fe7da40de70d1	4	0.95	D_0__Bacteria;D_1__Proteobacteria;D_2__Deltaproteobacteria;D_3__Myxococcales;D_4__Nannocystaceae;D_5__uncultured;Ambiguous_taxa
ad6ba948fdd3dee3c0a26ca8b625879e	4	1.00	D_0__Bacteria;D_1__Proteobacteria;D_2__Gammaproteobacteria
fa5d7e11432c8e7ab07590c5bd25d55e	3	1.00	D_0__Bacteria;D_1__Proteobacteria;D_2__Alphaproteobacteria;D_3__Rhodobacterales;D_4__Rhodobacteraceae
b08249dcfa5b42b7817b352a7c13a63a	3	1.00	D_0__Bacteria;D_1__Planctomycetes;D_2__Planctomycetacia;D_3__Pirellulales;D_4__Pirellulaceae;D_5__Rubripirellula
0f8311b8f17bf7fb656324ce196d1bc9	3	0.94	D_0__Bacteria;D_1__Proteobacteria;D_2__Gammaproteobacteria;D_3__Oceanospirillales;D_4__Endozoicomonadaceae;D_5__Endozoicomonas;Ambiguous_taxa
5ba019aa47388ac3cd9ff978824246fb	3	0.78	D_0__Bacteria;D_1__Proteobacteria;D_2__Alphaproteobacteria;D_3__uncultured
ddbd631cf4472a43ae0c6439fd930058	3	0.99	D_0__Bacteria;D_1__Bacteroidetes;D_2__Bacteroidia;D_3__Flavobacteriales;D_4__Flavobacteriaceae;D_5__NS2b marine group

bc29fb8338ce52cf8b48125d4c9e575c	3	1.00	D_0__Bacteria;D_1__Proteobacteria;D_2__Alphaproteobacteria;D_3__Rhodospirillales;D_4__AEGEAN-169 marine group
0610bbb5d172a09606dcb7f640b4d3bc	3	0.95	D_0__Bacteria;D_1__Chloroflexi;D_2__Anaerolineae
a7d8a2ff2804e30cab6002b1a7e5d739	3	0.99	D_0__Bacteria;D_1__Proteobacteria;D_2__Gammaproteobacteria;D_3__Alteromonadales;D_4__Colwelliaceae;D_5__ Thalassotalea
65a27ba6edc1d275d2ecafd787a21198	2	1.00	D_0__Bacteria
5cb0831e8f2297156c8e8ec13affcb47	2	0.99	D_0__Bacteria;D_1__Planctomycetes;D_2__Planctomycetacia;D_3__Pirellulales;D_4__Pirellulaceae;D_5__uncultured; D_6__marine metagenome
a3999a07a4a8b56b135ad8f837cfef30	2	0.99	D_0__Bacteria;D_1__Bacteroidetes;D_2__Bacteroidia;D_3__Flavobacteriales;D_4__NS9 marine group;D_5__uncultured marine bacterium;D_6__uncultured marine bacterium
dcf1e0f4fc3184efac88c1c93d6e703a	2	0.98	D_0__Bacteria;D_1__Bacteroidetes;D_2__Bacteroidia;D_3__Flavobacteriales;D_4__Flavobacteriaceae;D_5__Aurantiv irga;Ambiguous_taxa
72575a728cd13e723ee3ec708a4a6dae	2	0.93	D_0__Bacteria;D_1__Proteobacteria;D_2__Alphaproteobacteria;D_3__Rhodospirillales;D_4__AEGEAN-169 marine group;D_5__uncultured marine bacterium;D_6__uncultured marine bacterium
2853265953caf52402fb6bce42e1e349	2	0.99	D_0__Bacteria;D_1__Spirochaetes;D_2__Spirochaetia;D_3__Spirochaetales;D_4__Spirochaetaceae;D_5__uncultured
4686c4b285a7435b5d620997593e74a3	2	0.96	D_0__Bacteria;D_1__Proteobacteria;D_2__Gammaproteobacteria;D_3__Betaproteobacteriales;D_4__Nitrosomonadace ae;D_5__Nitrosospira;Ambiguous_taxa
d5f7207464732b71ec06cb0c489fc5cd	2	0.99	D_0__Bacteria;D_1__Planctomycetes;D_2__Phycisphaerae;D_3__Phycisphaerales;D_4__Phycisphaeraceae;D_5__Ura nia-1B-19 marine sediment group;D_6__uncultured bacterium
cddef885ae4bc361d6c4ecf979c8feed	2	0.94	D_0__Bacteria;D_1__Verrucomicrobia;D_2__Verrucomicrobiae;D_3__Pedosphaerales;D_4__Pedosphaeraceae;D_5__ SCGC AAA164-E04;Ambiguous_taxa
514bda9350438af8fd75a91683c9f851	1	0.81	D_0__Bacteria;D_1__Proteobacteria;D_2__Alphaproteobacteria;D_3__Rhizobiales;D_4__Hyphomicrobiaceae;D_5__Fi lomicrobium

2ed64430c4aeb03267953f03f3fe8d29	1	0.82	D_0__Bacteria;D_1__Proteobacteria;D_2__Gammaproteobacteria;D_3__Thiomicrospirales;D_4__Thioglobaceae;D_5__SUP05 cluster
22021986e6d84df8883a9f7e23ddbe91	1	0.80	D_0__Bacteria;D_1__Proteobacteria;D_2__Gammaproteobacteria;D_3__Arenicellales;D_4__Arenicellaceae;D_5__uncultured;D_6__uncultured bacterium
ed86fecf77d6178618757a5c2cd049ca	1	1.00	D_0__Bacteria;D_1__Proteobacteria;D_2__Gammaproteobacteria

Table S9. ASVs (98) from *Axinella damicormis* with percentage of confidence ≥ 75%.

Feature ID	Number of sequences	Confidence	Taxon
1c772a071db1578e1b24e49da65ef169	9818	1.00	D_0__Bacteria;D_1__Proteobacteria;D_2__Gammaproteobacteria;D_3__UBA10353 marine group
19137f8420401d33a200aec5efd79904	3165	1.00	D_0__Bacteria;D_1__Proteobacteria;D_2__Gammaproteobacteria;D_3__OM182 clade;D_4__uncultured gamma proteobacterium;D_5__uncultured gamma proteobacterium;D_6__uncultured gamma proteobacterium
ab416220d7ad0c7e500f37fe3951b27d	1880	0.98	D_0__Bacteria;D_1__Nitrospirae;D_2__Nitrospira;D_3__Nitrospirales;D_4__Nitrospiraceae;D_5__Nitrospira;D_6__uncultured Nitrospirae bacterium
6619a97d6147ea46cedfeb27b2ceb4af	1077	0.96	D_0__Bacteria
f97bd75415f062629da2641ebec60c8d	1020	0.99	D_0__Bacteria;D_1__Proteobacteria;D_2__Gammaproteobacteria;D_3__Cellvibrionales;D_4__BD2-7
2a254ae88841d11e080dbf011f047fe8	870	1.00	D_0__Archaea;D_1__Thaumarchaeota;D_2__Nitrososphaeria;D_3__Nitrosopumilales;D_4__Nitrosopumilaceae;D_5__Cenarchaeum;Ambiguous_taxa
848ad90cd05b479dfda8c5caab01d6f3	694	1.00	D_0__Bacteria;D_1__Proteobacteria;D_2__Gammaproteobacteria;D_3__HOC36
196ca9f8f16dce20290694e4566d0cd1	170	0.96	D_0__Bacteria
44f05bb926fdbfd4f20dc8c2ac028eb1	146	1.00	D_0__Bacteria;D_1__Proteobacteria;D_2__Deltaproteobacteria;D_3__Bdellovibrionales;D_4__Bdellovibrionaceae;D_5__Bdellovibrio;D_6__uncultured Bdellovibrio sp.
31c9c153674277aff7e28a05ec2dc976	72	0.96	D_0__Bacteria;D_1__Spirochaetes;D_2__Spirochaetia;D_3__Spirochaetales;D_4__Spirochaetaceae;D_5__Spirochaeta 2;D_6__uncultured bacterium
fe1162016c00379fc27e8b6758ed900b	48	0.77	D_0__Bacteria;D_1__Proteobacteria;D_2__Alphaproteobacteria;D_3__SAR11 clade;D_4__Clade II
5812f609d4ac6d4cc4a458899ac10819	48	0.83	D_0__Bacteria;D_1__Spirochaetes;D_2__Spirochaetia;D_3__Spirochaetales;D_4__Spirochaetaceae;D_5__Salinispira; Ambiguous_taxa

ee0f5caff94365896ef095e26d1fed6d	37	0.75	D_0__Bacteria;D_1__Proteobacteria;D_2__Alphaproteobacteria;D_3__SAR11 clade;D_4__Clade I;D_5__Clade Ia
383d0646c340d55509275f4ed7a5d71b	33	0.98	D_0__Bacteria;D_1__Proteobacteria;D_2__Alphaproteobacteria;D_3__SAR11 clade;D_4__Clade I;D_5__Clade Ia
f45468a2fb22d6101d596b223e1ba4ab	31	0.80	D_0__Bacteria;D_1__Proteobacteria;D_2__Alphaproteobacteria;D_3__SAR11 clade;D_4__Clade I;D_5__Clade Ia
bef08b4f2d4b0d20344998cbd8374092	31	0.93	D_0__Bacteria;D_1__Proteobacteria;D_2__Alphaproteobacteria;D_3__uncultured;D_4__uncultured alpha proteobacterium;D_5__uncultured alpha proteobacterium;D_6__uncultured alpha proteobacterium
b03d52ef0e9995fcc323addecdf2c17a	25	0.83	D_0__Bacteria;D_1__Spirochaetes;D_2__Spirochaetia;D_3__Spirochaetales;D_4__Spirochaetaceae;D_5__Salinispira;Ambiguous_taxa
0f2612f663d381a482bf3e58387fe78c	23	1.00	D_0__Bacteria;D_1__Planctomycetes;D_2__Planctomycetacia;D_3__Pirellulales;D_4__Pirellulaceae;D_5__Rhodopirellula;D_6__marine metagenome
dfaf617afcd26dc12f9cf96355971555	23	1.00	D_0__Bacteria;D_1__Proteobacteria;D_2__Deltaproteobacteria;D_3__NB1-j;D_4__uncultured delta proteobacterium;D_5__uncultured delta proteobacterium;D_6__uncultured delta proteobacterium
8f3aa47b47d7ef67537b715cebda862f	22	0.97	D_0__Bacteria
78e1cef68072d74b4b290c959265f818	18	1.00	D_0__Bacteria;D_1__Bacteroidetes;D_2__Bacteroidia;D_3__Flavobacteriales;D_4__Flavobacteriaceae;D_5__NS4 marine group
26db72af554f00518f4d11d60965dd7a	18	1.00	D_0__Bacteria;D_1__Proteobacteria;D_2__Gammaproteobacteria;D_3__Vibrionales;D_4__Vibrionaceae;D_5__Vibrio;D_6__uncultured bacterium
72575a728cd13e723ee3ec708a4a6dae	17	0.93	D_0__Bacteria;D_1__Proteobacteria;D_2__Alphaproteobacteria;D_3__Rhodospirillales;D_4__AEGEAN-169 marine group;D_5__uncultured marine bacterium;D_6__uncultured marine bacterium
65e607c7385c091c2881209e95c45a54	17	0.80	D_0__Bacteria;D_1__Proteobacteria;D_2__Alphaproteobacteria;D_3__Rickettsiales;D_4__S25-593;Ambiguous_taxa;Ambiguous_taxa
6f6e79b40c2205294a881fedd0bb1da6	16	0.87	D_0__Bacteria;D_1__Proteobacteria;D_2__Gammaproteobacteria;D_3__SAR86 clade;D_4__bacterium WHC4-9;D_5__bacterium WHC4-9;D_6__bacterium WHC4-9
6598bf8b035cf4c390b8bec3d94cfdf2	16	0.82	D_0__Bacteria;D_1__Firmicutes;D_2__Clostridia;D_3__Clostridiales;D_4__Peptostreptococcaceae;D_5__Paeniclostridium;D_6__uncultured bacterium

2fafaaef9248efb80056ce99aec93764	15	0.98	D_0__Bacteria;D_1__Cyanobacteria;D_2__Oxyphotobacteria;D_3__Synechococcales;D_4__Cyanobiaceae;D_5__Synechococcus CC9902
f879dba477998c0a9f48ad6fa0cc8e0d	12	0.98	D_0__Bacteria;D_1__Proteobacteria;D_2__Alphaproteobacteria;D_3__Rhodobacterales;D_4__Rhodobacteraceae;D_5__Ascidiaceihabitans
a3999a07a4a8b56b135ad8f837cfef30	12	0.99	D_0__Bacteria;D_1__Bacteroidetes;D_2__Bacteroidia;D_3__Flavobacteriales;D_4__NS9 marine group;D_5__uncultured marine bacterium;D_6__uncultured marine bacterium
70581dea5ded43371fd6fefd46f3a5a7	12	0.98	D_0__Bacteria;D_1__Proteobacteria;D_2__Alphaproteobacteria;D_3__Rhizobiales;D_4__Hyphomicrobiaceae;D_5__Filomicrobium;Ambiguous_taxa
dc88762d931633f035ba4ed4695f6a3c	11	1.00	D_0__Bacteria;D_1__Proteobacteria;D_2__Alphaproteobacteria;D_3__Parvibaculales;D_4__OCS116 clade
4245b126888cd95fe8e1662a756cd189	11	0.95	D_0__Bacteria;D_1__Actinobacteria;D_2__Acidimicrobiia;D_3__Actinomarinales;D_4__Actinomarinaceae;D_5__Candidatus Actinomarina;D_6__uncultured bacterium
add3536443f7fb635867074699cc4a39	10	1.00	D_0__Bacteria;D_1__Proteobacteria;D_2__Alphaproteobacteria;D_3__Puniceispirillales;D_4__SAR116 clade;D_5__uncultured organism;D_6__uncultured organism
68736e5da6063e7498a762bbccd02518	10	0.85	D_0__Bacteria;D_1__Actinobacteria;D_2__Acidimicrobiia;D_3__Microtrichales;D_4__Microtrichaceae;D_5__Sva0996 marine group;Ambiguous_taxa
73d226840a5d395195f0df9ee6c5d1e8	10	0.97	D_0__Bacteria;D_1__Proteobacteria;D_2__Alphaproteobacteria;D_3__SAR11 clade;D_4__Clade IV;Ambiguous_taxa;Ambiguous_taxa
258ae07e67fbdce3520dd402df7ab986	9	1.00	D_0__Bacteria;D_1__Verrucomicrobia;D_2__Verrucomicrobiae;D_3__Verrucomicrobiales;D_4__DEV007;Ambiguous_taxa;Ambiguous_taxa
6d82646963474ca7ea0a9e236eaf3918	9	1.00	D_0__Bacteria;D_1__Bacteroidetes;D_2__Bacteroidia;D_3__Flavobacteriales;D_4__Flavobacteriaceae;D_5__NS5 marine group
ee5f5921190381d04d53b26080f01429	9	0.96	D_0__Bacteria
82e2fcbe556f2d457c561098cb4eeb27	8	0.85	D_0__Bacteria;D_1__Proteobacteria;D_2__Gammaproteobacteria;D_3__Oceanospirillales;D_4__Endozoicomonadaceae;D_5__Endozoicomonas;D_6__Endozoicomonas sp. Ez302

8e0580f0918c4dcac2c07f3162fd7104	8	0.83	D_0__Bacteria;D_1__Bacteroidetes;D_2__Bacteroidia;D_3__Flavobacteriales;D_4__Flavobacteriaceae;D_5__NS2b marine group;D_6__uncultured bacterium
91ea660b9d1f840704c6dca1ff9f241b	8	1.00	D_0__Bacteria;D_1__Bacteroidetes;D_2__Bacteroidia;D_3__Flavobacteriales;D_4__Flavobacteriaceae;D_5__NS4 marine group
378cfeba13f7e8e24f23e836f5a633a7	8	0.82	D_0__Bacteria;D_1__Bacteroidetes;D_2__Bacteroidia;D_3__Cytophagales;D_4__Cyclobacteriaceae;D_5__Marinoscill um;Ambiguous_taxa
5fa80111fb467728b8fd040cd3cc1411	8	0.78	D_0__Bacteria;D_1__Proteobacteria;D_2__Alphaproteobacteria;D_3__Thalassobaculales;D_4__Nisaeaceae;D_5__OM 75 clade;Ambiguous_taxa
f36778f732a655750b4550b3f670f1b8	8	1.00	D_0__Bacteria;D_1__Proteobacteria;D_2__Alphaproteobacteria;D_3__Rhodospirillales;D_4__AEGEAN-169 marine group
fe1a9e0e68cbd9d447ebb18e4a736a41	7	0.98	D_0__Bacteria;D_1__Cyanobacteria;D_2__Oxyphotobacteria;D_3__Synechococcales;D_4__Cyanobiaceae;D_5__Cyan obium PCC-6307;Ambiguous_taxa
5cb0831e8f2297156c8e8ec13affcb47	7	0.99	D_0__Bacteria;D_1__Planctomycetes;D_2__Planctomycetacia;D_3__Pirellulales;D_4__Pirellulaceae;D_5__uncultured; D_6__marine metagenome
a44824b095e1a7b84be67fa7c65bba66	7	0.97	D_0__Bacteria;D_1__Bacteroidetes;D_2__Bacteroidia;D_3__Flavobacteriales;D_4__Crocinitomicaceae;D_5__Fluviico la;D_6__uncultured marine bacterium
80650a4e365cd2b09631f1d913d06d76	7	0.99	D_0__Bacteria;D_1__Proteobacteria;D_2__Alphaproteobacteria;D_3__Rhodospirillales;D_4__AEGEAN-169 marine group;Ambiguous_taxa;Ambiguous_taxa
66ce2907819edc5dd2d49163cbe15956	7	0.77	D_0__Bacteria;D_1__Firmicutes;D_2__Clostridia;D_3__Clostridiales;D_4__Clostridiaceae 1;D_5__Clostridium sensu stricto 1;D_6__uncultured bacterium
a424f2f52964bd31ab5fa062fa54b78b	7	0.76	D_0__Bacteria;D_1__Bacteroidetes;D_2__Bacteroidia;D_3__Flavobacteriales;D_4__Flavobacteriaceae;D_5__Tenaciba culum;D_6__Tenacibaculum soleae
04f49f9ceb77405195de3ec0844dfbeb	6	1.00	D_0__Bacteria;D_1__Actinobacteria;D_2__Actinobacteria;D_3__PeM15
0e028214895602d4967e7f6f2d04d0a9	6	1.00	D_0__Bacteria;D_1__Proteobacteria;D_2__Alphaproteobacteria;D_3__Rhizobiales;D_4__Stappiaceae;D_5__Pseudovi brio;Ambiguous_taxa

7aa1817b824952245b72f7627ff71a0d	6	0.95	D_0__Bacteria;D_1__Bacteroidetes;D_2__Bacteroidia;D_3__Bacteroidales;D_4__Bacteroidaceae;D_5__Bacteroides;D_6__uncultured isopod gut bacterium
e47d8acb20261648f8ae16d465fabfb8	6	1.00	D_0__Archaea;D_1__Thaumarchaeota;D_2__Nitrososphaeria;D_3__Nitrosopumilales;D_4__Nitrosopumilaceae
c11143942f6caf7c788882440d06fa3f	6	0.99	D_0__Bacteria;D_1__Proteobacteria;D_2__Gammaproteobacteria;D_3__UBA10353 marine group
a1f9c1805b721060a47df24e62de23e5	6	0.95	D_0__Bacteria;D_1__Proteobacteria;D_2__Alphaproteobacteria;D_3__Rhodobacterales;D_4__Rhodobacteraceae;D_5__uncultured
67510051a3ca1e02ee77d042eb05abe7	6	0.97	D_0__Bacteria
0e263a618c515f9556c7c2af279ad2a7	6	0.97	D_0__Bacteria;D_1__Proteobacteria;D_2__Gammaproteobacteria;D_3__Oceanospirillales;D_4__Endozoicomonadaceae;D_5__Endozoicomonas;D_6__Endozoicomonas sp. KJ13
66edcabc3197f5d3b3bfd40d13205011	5	1.00	D_0__Bacteria;D_1__Proteobacteria;D_2__Alphaproteobacteria;D_3__Rhodospirillales;D_4__AEGEAN-169 marine group;Ambiguous_taxa;Ambiguous_taxa
d85456cdcc158131c1d6624e6cc3e0cb	5	1.00	D_0__Bacteria;D_1__Proteobacteria;D_2__Gammaproteobacteria;D_3__SAR86 clade;Ambiguous_taxa;Ambiguous_taxa;Ambiguous_taxa
bbb3078cac73ba22fb709fdbd8bb551c	5	1.00	D_0__Bacteria;D_1__Verrucomicrobia;D_2__Verrucomicrobiae;D_3__Verrucomicrobiales;D_4__Rubritaleaceae;D_5__Rubritalea;D_6__uncultured bacterium
f441852fb48304760140fff7cf3354fc	5	0.77	D_0__Archaea;D_1__Euryarchaeota;D_2__Thermoplasmata;D_3__Marine Group II;D_4__uncultured marine archaeon;D_5__uncultured marine archaeon;D_6__uncultured marine archaeon
6944ba506d930cf0d8417ab1bcb8a024	5	0.97	D_0__Bacteria;D_1__Bacteroidetes;D_2__Bacteroidia;D_3__Flavobacteriales;D_4__Flavobacteriaceae;D_5__NS5 marine group;D_6__uncultured Flavobacteriia bacterium
f20d04f063659ed6c30afc5307b38856	5	1.00	D_0__Bacteria;D_1__Planctomycetes;D_2__Planctomycetacia;D_3__Pirellulales;D_4__Pirellulaceae;D_5__Blastopirellula
46a27e550bc4fafd3d37d0eb9811d695	5	1.00	D_0__Bacteria;D_1__Proteobacteria;D_2__Deltaproteobacteria;D_3__Bdellovibrionales;D_4__Bdellovibrionaceae;D_5__Bdellovibrio;D_6__uncultured delta proteobacterium
49b23bc0ededaf0d21bf19b80e2076a6	5	1.00	D_0__Bacteria;D_1__Proteobacteria;D_2__Alphaproteobacteria;D_3__Parvibaculales;D_4__OCS116 clade

dcf1e0f4fc3184efac88c1c93d6e703a	4	0.98	D_0__Bacteria;D_1__Bacteroidetes;D_2__Bacteroidia;D_3__Flavobacteriales;D_4__Flavobacteriaceae;D_5__Aurantivirga;Ambiguous_taxa
b50985bf9adcb1c8b7bcfec5356836a9	4	0.99	D_0__Bacteria;D_1__Bacteroidetes;D_2__Bacteroidia;D_3__Flavobacteriales;D_4__Cryomorphaceae;D_5__uncultured;D_6__uncultured Flavobacteriia bacterium
9c0910f0dbf90db8e850654123ba902d	4	1.00	D_0__Bacteria;D_1__Actinobacteria;D_2__Acidimicrobiia;D_3__Microtrichales;D_4__Microtrichaceae;D_5__Sva0996 marine group
5f184d3a1868df0460e84fdde153a361	4	0.95	D_0__Bacteria;D_1__Chloroflexi;D_2__Dehalococcoidia;D_3__SAR202 clade;Ambiguous_taxa;Ambiguous_taxa;Ambiguous_taxa
a58f27e56e78f4c937af4b950555599c	4	1.00	D_0__Bacteria;D_1__Proteobacteria;D_2__Alphaproteobacteria;D_3__Puniceispirillales;D_4__SAR116 clade
d2e7a135364178afa711645409549994	4	0.92	D_0__Bacteria;D_1__Proteobacteria;D_2__Gammaproteobacteria;D_3__SAR86 clade;D_4__uncultured marine bacterium;D_5__uncultured marine bacterium;D_6__uncultured marine bacterium
65a27ba6edc1d275d2ecafd787a21198	3	1.00	D_0__Bacteria
461a220851589b7510ae4edeba74f5fb	3	1.00	D_0__Bacteria;D_1__Proteobacteria;D_2__Gammaproteobacteria;D_3__Oceanospirillales;D_4__Pseudohongiellaceae;D_5__Pseudohongiella
b08249dcfa5b42b7817b352a7c13a63a	3	1.00	D_0__Bacteria;D_1__Planctomycetes;D_2__Planctomycetacia;D_3__Pirellulales;D_4__Pirellulaceae;D_5__Rubripirellula
55b500bb0664b37a772d2ecc7d9540bf	3	1.00	D_0__Bacteria;D_1__Gemmatimonadetes;D_2__BD2-11 terrestrial group;Ambiguous_taxa;Ambiguous_taxa;Ambiguous_taxa;Ambiguous_taxa
7a988c1beea869cd7f8c054eee481405	3	0.98	D_0__Bacteria;D_1__Proteobacteria;D_2__Deltaproteobacteria;D_3__SAR324 clade(Marine group B);D_4__uncultured SAR324 cluster bacterium;D_5__uncultured SAR324 cluster bacterium;D_6__uncultured SAR324 cluster bacterium
840577c29f59301756b6321bceefa3af	3	1.00	D_0__Bacteria;D_1__Verrucomicrobia;D_2__Verrucomicrobiae;D_3__Pedosphaerales;D_4__Pedosphaeraceae;D_5__SCGC AAA164-E04;Ambiguous_taxa
9285fb928534b86aad25a8862cd9dc0c	3	1.00	D_0__Bacteria;D_1__Proteobacteria;D_2__Alphaproteobacteria;D_3__Parvibaculales;D_4__Parvibaculaceae;D_5__uncultured;Ambiguous_taxa

7117f1efa85e803f054b2ca25d401720	3	1.00	D_0__Bacteria;D_1__Chloroflexi;D_2__Dehalococcoidia;D_3__SAR202 clade;Ambiguous_taxa;Ambiguous_taxa;Ambiguous_taxa
8db0caf5ee40271902d2be9e6661d66b	3	0.97	D_0__Bacteria;D_1__Proteobacteria;D_2__Gammaproteobacteria;D_3__Thiotrichales;D_4__Thiotrichaceae;D_5__uncultured;D_6__uncultured marine bacterium
6cad95b371c104106918bf8d6f168082	3	1.00	D_0__Bacteria;D_1__Dadabacteria;D_2__Dadabacteriia;D_3__Dadabacteriales;D_4__metagenome;D_5__metagenome;D_6__metagenome
6706a0021d0e5765b6c42a6a4dc73965	3	1.00	D_0__Bacteria;D_1__Proteobacteria;D_2__Gammaproteobacteria;D_3__SAR86 clade
6b2e1e7cc0f49a5c2558ed74a173cbb9	3	0.99	D_0__Bacteria;D_1__Spirochaetes;D_2__Leptospirae;D_3__Leptospirales;D_4__Leptospiraceae;D_5__Turneriella
d49e5e5bf13e0f758250bb4e9aeab9ae	3	1.00	D_0__Bacteria;D_1__Proteobacteria;D_2__Gammaproteobacteria;D_3__HOC36;D_4__uncultured gamma proteobacterium;D_5__uncultured gamma proteobacterium;D_6__uncultured gamma proteobacterium
447ca590133816987ba59a99d29b0147	3	0.99	D_0__Bacteria
f9a10e789b35835a58d32a92eea69eed	3	0.79	D_0__Bacteria;D_1__Proteobacteria;D_2__Deltaproteobacteria;D_3__NB1-j;Ambiguous_taxa;Ambiguous_taxa;Ambiguous_taxa
89690644f5e33fe65b76682e71432b26	3	0.97	D_0__Bacteria;D_1__Proteobacteria;D_2__Deltaproteobacteria;D_3__PB19;D_4__uncultured marine bacterium;D_5__uncultured marine bacterium;D_6__uncultured marine bacterium
eb1f5ea9269dd4c0cba4969234eca5ac	2	1.00	D_0__Bacteria;D_1__Bacteroidetes;D_2__Bacteroidia;D_3__Flavobacteriales;D_4__Cryomorphaceae;D_5__uncultured
bee4ae653d9d4d98ce09c5472800abec	2	1.00	D_0__Bacteria;D_1__Marinimicrobia (SAR406 clade);D_2__uncultured marine bacterium;D_3__uncultured marine bacterium;D_4__uncultured marine bacterium;D_5__uncultured marine bacterium;D_6__uncultured marine bacterium
28ad0b067c0eaff714a3843b343726bd	2	0.98	D_0__Bacteria
dec45a4dc81da2006cdb4b9a1f67b1ff	2	0.81	D_0__Bacteria;D_1__Proteobacteria;D_2__Gammaproteobacteria;D_3__Thiomicrospirales;D_4__Thioglobaceae;D_5__SUP05 cluster
f0e40f6447eb6df965fbc0cacc7d0df4	1	1.00	D_0__Bacteria;D_1__Proteobacteria;D_2__Gammaproteobacteria;D_3__Ectothiorhodospirales;D_4__Ectothiorhodospiraceae;D_5__uncultured;Ambiguous_taxa

Table S10. ASVs (316) from *Acanthella acuta* with percentage of confidence ≥ 75%.

Feature ID	Number of sequences	Confidence	Taxon
3efd2b8382008071a18f146700bc2455	10206	0.86	D_0__Bacteria;D_1__Proteobacteria;D_2__Gammaproteobacteria;D_3__Betaproteobacteriales
2f6d1009e09db01730433c8a018cfbb5	3284	1.00	D_0__Archaea;D_1__Thaumarchaeota;D_2__Nitrososphaeria;D_3__Nitrosopumilales;D_4__Nitrosopumilaceae;D_5__Cenarchaeum
97a8f3b071f47965b8b338a799765c52	2913	0.89	D_0__Bacteria;D_1__Proteobacteria;D_2__Gammaproteobacteria;D_3__Betaproteobacteriales
2ce87a02b3de06c9b5eb3d4b5d19b6fd	1403	0.99	D_0__Bacteria;D_1__Nitrospirae;D_2__Nitrospira;D_3__Nitrospirales;D_4__Nitrospiraceae;D_5__Nitrospira;D_6__uncultured Nitrospirae bacterium
0240de2b5d6dd5950495c2fa5fadab22	1400	0.86	D_0__Bacteria;D_1__Proteobacteria;D_2__Gammaproteobacteria;D_3__Betaproteobacteriales;D_4__EC94;D_5__uncultured bacterium;D_6__uncultured bacterium
1476e534629533874a027ce52c0afd43	966	0.98	D_0__Bacteria;D_1__Proteobacteria;D_2__Alphaproteobacteria
0ddc2c2e9de76c9e0088d433b47ae70d	913	1.00	D_0__Bacteria;D_1__Proteobacteria;D_2__Gammaproteobacteria
8269d77688c1acd9fc6b0b62c52873b5	873	1.00	D_0__Bacteria;D_1__Actinobacteria;D_2__Acidimicrobiia;D_3__Microtrichales;D_4__Microtrichaceae;D_5__Sva0996 marine group;D_6__uncultured actinobacterium
2a59faa6a0e6fd61e33bea29c7a5d3c1	708	1.00	D_0__Bacteria
fdd07e1a08599e94e5f0e75ba687b72c	542	1.00	D_0__Bacteria;D_1__Proteobacteria;D_2__Gammaproteobacteria
155d50ede18165b1c412c31fc7ec2ca5	446	1.00	D_0__Bacteria;D_1__Proteobacteria;D_2__Gammaproteobacteria
44e5f5e92b382975964856838b238ffe	359	0.89	D_0__Bacteria;D_1__Bacteroidetes;D_2__Bacteroidia;D_3__Cytophagales;D_4__Cyclobacteriaceae;D_5__Ekhidna;Ambiguous_taxa
ed56f66f9218dc5bcd8edf0169be109d	296	0.85	D_0__Bacteria;D_1__Proteobacteria;D_2__Gammaproteobacteria;D_3__Betaproteobacteriales

82e2fcbe556f2d457c561098cb4eeb27	294	0.85	D_0__Bacteria;D_1__Proteobacteria;D_2__Gammaproteobacteria;D_3__Oceanospirillales;D_4__Endozoicomonadaceae;D_5__Endozoicomonas;D_6__Endozoicomonas sp. Ez302
7a5be8747b31b74f6374ff8854ff4455	292	0.79	D_0__Archaea;D_1__Thaumarchaeota;D_2__Nitrososphaeria;D_3__Nitrosopumilales;D_4__Nitrosopumilaceae;D_5__Candidatus Nitrosopumilus
2fd0473e3a2a58b77406c6cf7645dada	266	0.76	D_0__Bacteria;D_1__Chloroflexi;D_2__Dehalococcoidia;D_3__SAR202 clade;Ambiguous_taxa;Ambiguous_taxa;Ambiguous_taxa
ca6d1097d9d1a149c1d46f054e86b2cf	233	0.77	D_0__Bacteria;D_1__Proteobacteria;D_2__Alphaproteobacteria;D_3__Rickettsiales;D_4__Rickettsiaceae;D_5__Candidatus Cryptoprodotis;D_6__uncultured bacterium
c150ef610e86fe5d6e8a323c12402ec2	202	1.00	D_0__Bacteria;D_1__Proteobacteria;D_2__Gammaproteobacteria
f45468a2fb22d6101d596b223e1ba4ab	166	0.80	D_0__Bacteria;D_1__Proteobacteria;D_2__Alphaproteobacteria;D_3__SAR11 clade;D_4__Clade I;D_5__Clade Ia
ee0f5caff94365896ef095e26d1fed6d	160	0.75	D_0__Bacteria;D_1__Proteobacteria;D_2__Alphaproteobacteria;D_3__SAR11 clade;D_4__Clade I;D_5__Clade Ia
e154b8df737746aa4f3f86280b13499e	157	0.94	D_0__Bacteria;D_1__Proteobacteria;D_2__Alphaproteobacteria
383d0646c340d55509275f4ed7a5d71b	136	0.98	D_0__Bacteria;D_1__Proteobacteria;D_2__Alphaproteobacteria;D_3__SAR11 clade;D_4__Clade I;D_5__Clade Ia
c30f754cd181d29b4d295eac82d0640d	136	1.00	D_0__Bacteria;D_1__Proteobacteria;D_2__Alphaproteobacteria;D_3__Parvibaculales;D_4__PS1 clade
070c529a2966db6d6686878411930a1e	134	0.99	D_0__Bacteria;D_1__Proteobacteria;D_2__Alphaproteobacteria;D_3__uncultured;D_4__uncultured alpha proteobacterium;D_5__uncultured alpha proteobacterium;D_6__uncultured alpha proteobacterium
eda199cb440ae4195071ea393d9aacd3	134	0.76	D_0__Bacteria;D_1__Proteobacteria;D_2__Alphaproteobacteria;D_3__uncultured;D_4__uncultured bacterium;D_5__uncultured bacterium;D_6__uncultured bacterium
fe1a9e0e68cbd9d447ebb18e4a736a41	125	0.98	D_0__Bacteria;D_1__Cyanobacteria;D_2__Oxyphotobacteria;D_3__Synechococcales;D_4__Cyanobiaceae;D_5__Cyanobium PCC-6307;Ambiguous_taxa
0f2612f663d381a482bf3e58387fe78c	95	1.00	D_0__Bacteria;D_1__Planctomycetes;D_2__Planctomycetacia;D_3__Pirellulales;D_4__Pirellulaceae;D_5__Rhodopirellula;D_6__marine metagenome

78e1cef68072d74b4b290c959265f818	89	1.00	D_0__Bacteria;D_1__Bacteroidetes;D_2__Bacteroidia;D_3__Flavobacteriales;D_4__Flavobacteriaceae;D_5__NS4 marine group
add3536443f7fb635867074699cc4a39	79	1.00	D_0__Bacteria;D_1__Proteobacteria;D_2__Alphaproteobacteria;D_3__Puniceispirillales;D_4__SAR116 clade;D_5__uncultured organism;D_6__uncultured organism
8e0580f0918c4dcac2c07f3162fd7104	75	0.83	D_0__Bacteria;D_1__Bacteroidetes;D_2__Bacteroidia;D_3__Flavobacteriales;D_4__Flavobacteriaceae;D_5__NS2b marine group;D_6__uncultured bacterium
dc88762d931633f035ba4ed4695f6a3c	67	1.00	D_0__Bacteria;D_1__Proteobacteria;D_2__Alphaproteobacteria;D_3__Parvibaculales;D_4__OCS116 clade
85e5b31ba16cdfc3099584af526e4634	61	0.76	D_0__Bacteria;D_1__Proteobacteria;D_2__Alphaproteobacteria;D_3__Rhodobacterales;D_4__Rhodobacteraceae;D_5__HIMB11
f879dba477998c0a9f48ad6fa0cc8e0d	60	0.98	D_0__Bacteria;D_1__Proteobacteria;D_2__Alphaproteobacteria;D_3__Rhodobacterales;D_4__Rhodobacteraceae;D_5__Ascidiaceihabitans
68736e5da6063e7498a762bbccd02518	58	0.85	D_0__Bacteria;D_1__Actinobacteria;D_2__Acidimicrobiia;D_3__Microtrichales;D_4__Microtrichaceae;D_5__Sva0996 marine group;Ambiguous_taxa
86c0c55bc1ebedb5b9ffc55614666d48	56	0.76	D_0__Bacteria;D_1__Proteobacteria;D_2__Gammaproteobacteria;D_3__Nitrosococcales;D_4__Nitrosococcaceae;D_5__AqS1
079c2f03c2399757630a913a3955cbdd	54	0.79	D_0__Bacteria;D_1__Proteobacteria;D_2__Deltaproteobacteria;D_3__Bdellovibrionales;D_4__Bacteriovoracaceae;D_5__Peredibacter;D_6__uncultured bacterium
461a220851589b7510ae4edeba74f5fb	53	1.00	D_0__Bacteria;D_1__Proteobacteria;D_2__Gammaproteobacteria;D_3__Oceanospirillales;D_4__Pseudohongiellaceae;D_5__Pseudohongiella
5cb0831e8f2297156c8e8ec13affcb47	52	0.99	D_0__Bacteria;D_1__Planctomycetes;D_2__Planctomycetacia;D_3__Pirellulales;D_4__Pirellulaceae;D_5__uncultured;D_6__marine metagenome
258ae07e67fbdce3520dd402df7ab986	52	1.00	D_0__Bacteria;D_1__Verrucomicrobia;D_2__Verrucomicrobiae;D_3__Verrucomicrobiales;D_4__DEV007;Ambiguous_taxa;Ambiguous_taxa
2b4a0fe55fbc777744c12f982a4688b6	52	1.00	D_0__Bacteria;D_1__Proteobacteria;D_2__Gammaproteobacteria;D_3__SAR86 clade

a4a24e0ad24fefbc3bc8dc89470eec50	52	1.00	D_0__Bacteria;D_1__Cyanobacteria;D_2__Oxyphotobacteria;D_3__Synechococcales;D_4__Cyanobiaceae;D_5__Synechococcus CC9902
4bfd4c4367061dc1fc3a9171219c5751	51	0.88	D_0__Bacteria;D_1__Proteobacteria;D_2__Alphaproteobacteria;D_3__SAR11 clade;D_4__Clade I;D_5__Clade Ib
b321c598aab38165dc7b5aa21c117d7f	50	0.77	D_0__Bacteria;D_1__Proteobacteria;D_2__Alphaproteobacteria;D_3__SAR11 clade;D_4__Clade II
e36a8fd840109f87616a43efd738fcc9	50	1.00	D_0__Bacteria;D_1__Proteobacteria;D_2__Alphaproteobacteria;D_3__uncultured
45c69e6028639f47b1b042b78766f1d9	50	0.98	D_0__Bacteria;D_1__Proteobacteria;D_2__Alphaproteobacteria;D_3__uncultured;D_4__uncultured bacterium;D_5__uncultured bacterium;D_6__uncultured bacterium
fe1162016c00379fc27e8b6758ed900b	49	0.77	D_0__Bacteria;D_1__Proteobacteria;D_2__Alphaproteobacteria;D_3__SAR11 clade;D_4__Clade II
359d1dccc09235c4a42fb40357628f2c	48	1.00	D_0__Bacteria;D_1__Spirochaetes;D_2__Spirochaetia;D_3__Spirochaetales;D_4__Spirochaetaceae;D_5__Spirochaeta 2;D_6__uncultured marine bacterium
15e8a722f9d3380b5844fef13ec0cb79	48	0.98	D_0__Bacteria;D_1__Patescibacteria;D_2__Parcubacteria;D_3__Candidatus Kaiserbacteria
a3999a07a4a8b56b135ad8f837cfef30	45	0.99	D_0__Bacteria;D_1__Bacteroidetes;D_2__Bacteroidia;D_3__Flavobacteriales;D_4__NS9 marine group;D_5__uncultured marine bacterium;D_6__uncultured marine bacterium
dcf1e0f4fc3184efac88c1c93d6e703a	45	0.98	D_0__Bacteria;D_1__Bacteroidetes;D_2__Bacteroidia;D_3__Flavobacteriales;D_4__Flavobacteriaceae;D_5__Aurantivirga;Ambiguous_taxa
ccf2a3ec00b404364a421bd83d3ff0cf	42	1.00	D_0__Bacteria;D_1__Bacteroidetes;D_2__Bacteroidia;D_3__Flavobacteriales;D_4__NS9 marine group;D_5__uncultured marine bacterium;D_6__uncultured marine bacterium
edc849bcd6cd9ab2f0b5bf009b177b80	41	1.00	D_0__Bacteria;D_1__Verrucomicrobia;D_2__Verrucomicrobiae;D_3__Verrucomicrobiales;D_4__Rubritaleaceae;D_5__Roseibacillus;Ambiguous_taxa
9984834142ef1e09a61d6f508ade051c	40	0.79	D_0__Bacteria;D_1__Proteobacteria;D_2__Alphaproteobacteria;D_3__SAR11 clade;D_4__Clade I;D_5__Clade Ib
c961b3435a521d1245a12e04ff6c6e5f	39	1.00	D_0__Bacteria;D_1__Proteobacteria;D_2__Gammaproteobacteria;D_3__SAR86 clade

b6074bfef5fc28dd3cbd693fac8bfa9c	39	0.96	D_0__Bacteria;D_1__Bacteroidetes;D_2__Bacteroidia;D_3__Flavobacteriales;D_4__NS9 marine group;Ambiguous_taxa;Ambiguous_taxa
04f49f9ceb77405195de3ec0844dfbeb	38	1.00	D_0__Bacteria;D_1__Actinobacteria;D_2__Actinobacteria;D_3__PeM15
fa5d7e11432c8e7ab07590c5bd25d55e	38	1.00	D_0__Bacteria;D_1__Proteobacteria;D_2__Alphaproteobacteria;D_3__Rhodobacterales;D_4__Rhodobacteraceae
91ea660b9d1f840704c6dca1ff9f241b	37	1.00	D_0__Bacteria;D_1__Bacteroidetes;D_2__Bacteroidia;D_3__Flavobacteriales;D_4__Flavobacteriaceae;D_5__NS4 marine group
5137ef949a0c8bf90cc078dff87db691	37	1.00	D_0__Bacteria;D_1__Bacteroidetes;D_2__Bacteroidia;D_3__Flavobacteriales;D_4__Flavobacteriaceae;D_5__NS2b marine group
8e93ff62d0cc769ac5e520a48a8a2ae6	35	1.00	D_0__Bacteria;D_1__Planctomycetes;D_2__Planctomycetacia;D_3__Pirellulales;D_4__Pirellulaceae;D_5__Rhodopirel lula;D_6__marine metagenome
dd287e83b4ea94d0e969815ed3ef40cf	35	1.00	D_0__Bacteria;D_1__Proteobacteria;D_2__Alphaproteobacteria;D_3__Rickettsiales;D_4__S25-593
028fe57e5929c6780adeee7fb24dc88e	35	0.93	D_0__Bacteria;D_1__Actinobacteria;D_2__Acidimicrobiia;D_3__Microtrichales;D_4__Microtrichaceae;D_5__uncultu red;Ambiguous_taxa
55b500bb0664b37a772d2ecc7d9540bf	34	1.00	D_0__Bacteria;D_1__Gemmatimonadetes;D_2__BD2-11 terrestrial group;Ambiguous_taxa;Ambiguous_taxa;Ambiguous_taxa;Ambiguous_taxa
e4037ec08ff309d735bc73cf7a528309	34	0.99	D_0__Bacteria;D_1__Proteobacteria;D_2__Alphaproteobacteria;D_3__Rhodospirillales;D_4__AEGEAN-169 marine group;Ambiguous_taxa;Ambiguous_taxa
6a22aa2d30a07158dfa8ca0dc9cf9ba3	33	1.00	D_0__Bacteria;D_1__Proteobacteria;D_2__Alphaproteobacteria;D_3__Rhodobacterales;D_4__Rhodobacteraceae
b08249dcfa5b42b7817b352a7c13a63a	33	1.00	D_0__Bacteria;D_1__Planctomycetes;D_2__Planctomycetacia;D_3__Pirellulales;D_4__Pirellulaceae;D_5__Rubripirell ula
00cedd5fb1b50f08a03461b62c2b71c7	33	1.00	D_0__Bacteria;D_1__Spirochaetes;D_2__Leptospirae;D_3__Leptospirales;D_4__Leptospiraceae;D_5__uncultured
fe2ac3b697c636c804a4f4244468c04e	33	1.00	D_0__Bacteria;D_1__Bacteroidetes;D_2__Bacteroidia;D_3__Flavobacteriales;D_4__Flavobacteriaceae;D_5__NS4 marine group

378cfeba13f7e8e24f23e836f5a633a7	32	0.82	D_0__Bacteria;D_1__Bacteroidetes;D_2__Bacteroidia;D_3__Cytophagales;D_4__Cyclobacteriaceae;D_5__Marinoscillum;Ambiguous_taxa
78b2c7b3c09c494cdc9bf02475fe563b	32	1.00	D_0__Bacteria;D_1__Proteobacteria;D_2__Alphaproteobacteria;D_3__Rhodospirillales;D_4__AEGEAN-169 marine group
2fafaaef9248efb80056ce99aec93764	31	0.98	D_0__Bacteria;D_1__Cyanobacteria;D_2__Oxyphotobacteria;D_3__Synechococcales;D_4__Cyanobiaceae;D_5__Synechococcus CC9902
5fa80111fb467728b8fd040cd3cc1411	31	0.78	D_0__Bacteria;D_1__Proteobacteria;D_2__Alphaproteobacteria;D_3__Thalassobaculales;D_4__Nisaeaceae;D_5__OM75 clade;Ambiguous_taxa
fe49f3ecc5f6a97898f91e1bcf3b2116	31	0.87	D_0__Bacteria;D_1__Proteobacteria;D_2__Alphaproteobacteria;D_3__Rhodobacterales;D_4__Rhodobacteraceae;D_5__uncultured
a44824b095e1a7b84be67fa7c65bba66	30	0.97	D_0__Bacteria;D_1__Bacteroidetes;D_2__Bacteroidia;D_3__Flavobacteriales;D_4__Crocinitomicaceae;D_5__Fluviicola;D_6__uncultured marine bacterium
67e7850681f5c9fb8a49514daec61f00	30	0.95	D_0__Bacteria;D_1__Proteobacteria;D_2__Alphaproteobacteria;D_3__SAR11 clade;D_4__Clade IV;D_5__uncultured marine bacterium;D_6__uncultured marine bacterium
0cad1cd6996c774bf58d5ed530b42d40	30	0.99	D_0__Bacteria;D_1__Cyanobacteria;D_2__Oxyphotobacteria;D_3__Synechococcales;D_4__Cyanobiaceae;D_5__Prochlorococcus MIT9313
5ad7e9e20b02c8957fafe7c4ca13c0c6	29	0.85	D_0__Bacteria;D_1__Proteobacteria;D_2__Gammaproteobacteria;D_3__Oceanospirillales;D_4__Endozoicomonadaceae;D_5__Endozoicomonas;D_6__Endozoicomonas sp. Ez302
900dffac1d8df922f0720247b1591d86	29	0.78	D_0__Bacteria;D_1__Proteobacteria;D_2__Gammaproteobacteria;D_3__Betaproteobacteriales;D_4__uncultured;D_5__beta proteobacterium WY25;D_6__beta proteobacterium WY25
72575a728cd13e723ee3ec708a4a6dae	28	0.93	D_0__Bacteria;D_1__Proteobacteria;D_2__Alphaproteobacteria;D_3__Rhodospirillales;D_4__AEGEAN-169 marine group;D_5__uncultured marine bacterium;D_6__uncultured marine bacterium
86f95e992ee48fb85a69a6943aa38fc4	28	1.00	D_0__Bacteria;D_1__Proteobacteria;D_2__Alphaproteobacteria;D_3__Rhodospirillales;D_4__AEGEAN-169 marine group
f85f6e576f82600d24fc407733ce7c87	28	0.81	D_0__Bacteria;D_1__Proteobacteria;D_2__Alphaproteobacteria;D_3__Thalassobaculales;D_4__Nisaeaceae;D_5__OM75 clade;Ambiguous_taxa

a8e3bbccfe99da3f2ada9257b34c60ab	27	0.78	D_0__Bacteria;D_1__Cyanobacteria;D_2__Oxyphotobacteria;D_3__Synechococcales;D_4__Cyanobiaceae;D_5__Synechococcus CC9902;D_6__uncultured bacterium
9c0910f0dbf90db8e850654123ba902d	25	1.00	D_0__Bacteria;D_1__Actinobacteria;D_2__Acidimicrobiia;D_3__Microtrichales;D_4__Microtrichaceae;D_5__Sva0996 marine group
9d5fa81c5b5ad25d88ffde8a5fadb263	25	0.97	D_0__Bacteria;D_1__Nitrospirae;D_2__Nitrospira;D_3__Nitrospirales;D_4__Nitrospiraceae;D_5__Nitrospira;Ambiguous_taxa
0d79d854d90474c26737607a8d8a751d	25	1.00	D_0__Bacteria;D_1__Verrucomicrobia;D_2__Verrucomicrobiae;D_3__Arctic97B-4 marine group;D_4__uncultured marine bacterium;D_5__uncultured marine bacterium;D_6__uncultured marine bacterium
6f6e79b40c2205294a881fedd0bb1da6	24	0.87	D_0__Bacteria;D_1__Proteobacteria;D_2__Gammaproteobacteria;D_3__SAR86 clade;D_4__bacterium WHC4-9;D_5__bacterium WHC4-9;D_6__bacterium WHC4-9
67ff312feb3ba3201a75f86d95c46ac6	24	0.86	D_0__Bacteria;D_1__Proteobacteria;D_2__Gammaproteobacteria;D_3__Thiomicrospirales;D_4__Thioglobaceae;D_5__SUP05 cluster
43576d4c680c31800709107f1c988df6	24	0.99	D_0__Bacteria;D_1__Proteobacteria;D_2__Alphaproteobacteria;D_3__Rhodobacterales;D_4__Rhodobacteraceae;D_5__Ascidiaceihabitans;Ambiguous_taxa
80650a4e365cd2b09631f1d913d06d76	23	0.99	D_0__Bacteria;D_1__Proteobacteria;D_2__Alphaproteobacteria;D_3__Rhodospirillales;D_4__AEGEAN-169 marine group;Ambiguous_taxa;Ambiguous_taxa
45660e43a3dbdd77690c2450f88ffbca	23	0.90	D_0__Bacteria;D_1__Bacteroidetes;D_2__Bacteroidia;D_3__Flavobacteriales;D_4__Crocinitomicaceae;D_5__Fluviicola;D_6__uncultured marine bacterium
7a988c1beea869cd7f8c054eee481405	22	0.98	D_0__Bacteria;D_1__Proteobacteria;D_2__Deltaproteobacteria;D_3__SAR324 clade(Marine group B);D_4__uncultured SAR324 cluster bacterium;D_5__uncultured SAR324 cluster bacterium;D_6__uncultured SAR324 cluster bacterium
820b81b1fdf11329f853c17074b6a42f	22	0.86	D_0__Bacteria;D_1__Planctomycetes;D_2__Planctomycetacia;D_3__Pirellulales;D_4__Pirellulaceae;D_5__uncultured
07726f567ad4eb17db08e64aa30b4c1a	22	1.00	D_0__Bacteria;D_1__Epsilonbacteraeota;D_2__Campylobacteria;D_3__Campylobacterales;D_4__Arcobacteraceae;D_5__Arcobacter
d42016520c0144860ef1397895441cd4	22	1.00	D_0__Bacteria;D_1__Proteobacteria;D_2__Gammaproteobacteria;D_3__Oceanospirillales;D_4__Pseudohongiellaceae;D_5__Pseudohongiella

00f74702cc537d1ee52d948a49ef8326	22	0.87	D_0__Bacteria;D_1__Proteobacteria;D_2__Alphaproteobacteria;D_3__uncultured;D_4__uncultured marine bacterium;D_5__uncultured marine bacterium;D_6__uncultured marine bacterium
0da4421e8fcce112b686b932937d993b	21	0.89	D_0__Bacteria;D_1__Bacteroidetes;D_2__Bacteroidia;D_3__Flavobacteriales;D_4__Flavobacteriaceae;D_5__NS2b marine group;D_6__uncultured marine bacterium
e60df00c393282165d96827cb1ebfafa	21	0.95	D_0__Bacteria;D_1__Proteobacteria;D_2__Gammaproteobacteria;D_3__Oceanospirillales;D_4__Endozoicomonadaceae;D_5__Endozoicomonas;Ambiguous_taxa
125bbf185feaf46c614a9c1463b39603	21	1.00	D_0__Bacteria;D_1__Marinimicrobia (SAR406 clade)
577f1fb5da36c749b56a95f43f2a6d11	20	1.00	D_0__Bacteria;D_1__Proteobacteria;D_2__Deltaproteobacteria;D_3__SAR324 clade(Marine group B)
73d226840a5d395195f0df9ee6c5d1e8	20	0.97	D_0__Bacteria;D_1__Proteobacteria;D_2__Alphaproteobacteria;D_3__SAR11 clade;D_4__Clade IV;Ambiguous_taxa;Ambiguous_taxa
66ce2907819edc5dd2d49163cbe15956	20	0.77	D_0__Bacteria;D_1__Firmicutes;D_2__Clostridia;D_3__Clostridiales;D_4__Clostridiaceae 1;D_5__Clostridium sensu stricto 1;D_6__uncultured bacterium
5f184d3a1868df0460e84fdde153a361	20	0.95	D_0__Bacteria;D_1__Chloroflexi;D_2__Dehalococcoidia;D_3__SAR202 clade;Ambiguous_taxa;Ambiguous_taxa;Ambiguous_taxa
5ce2b495deab6bda77c74f21ce06f1e7	20	1.00	D_0__Bacteria;D_1__Proteobacteria;D_2__Gammaproteobacteria
c5c77a7d3212a2c4590720e384e231bd	20	0.94	D_0__Bacteria;D_1__Proteobacteria;D_2__Gammaproteobacteria;D_3__OM182 clade;Ambiguous_taxa;Ambiguous_taxa;Ambiguous_taxa
dd07e4d07ab834a9acd80d3466f54297	19	0.95	D_0__Bacteria;D_1__Verrucomicrobia;D_2__Verrucomicrobiae;D_3__Verrucomicrobiales;D_4__DEV007;Ambiguous_taxa;Ambiguous_taxa
352cfca400a704b73f9604757f6b08aa	19	0.97	D_0__Bacteria;D_1__Actinobacteria;D_2__Acidimicrobiia;D_3__Microtrichales;D_4__Microtrichaceae;D_5__Sva0996 marine group;D_6__uncultured bacterium
94c664cdd97233016977cf3b402984bb	18	0.79	D_0__Bacteria;D_1__Bacteroidetes;D_2__Bacteroidia;D_3__Flavobacteriales;D_4__Flavobacteriaceae;D_5__NS5 marine group;Ambiguous_taxa

8d362edb12fb614c931bbf40d43ed790	18	0.96	D_0__Bacteria;D_1__Proteobacteria;D_2__Alphaproteobacteria;D_3__Rhodobacterales;D_4__Rhodobacteraceae;D_5__uncultured
9a1cdf737aa1530f85ff7247364ff319	18	0.99	D_0__Bacteria;D_1__Proteobacteria;D_2__Gammaproteobacteria;D_3__Cellvibrionales;D_4__Halieaceae;D_5__OM60(NOR5) clade
98ea40ad3c6cfe19fbadc401fed8fc9e	18	0.92	D_0__Bacteria;D_1__Proteobacteria;D_2__Alphaproteobacteria;D_3__SAR11 clade;D_4__Clade IV;D_5__uncultured marine bacterium;D_6__uncultured marine bacterium
de0715391da35c9acd7ca460a57709a8	18	0.97	D_0__Bacteria;D_1__Cyanobacteria;D_2__Oxyphotobacteria;D_3__Synechococcales;D_4__Cyanobiaceae;D_5__Cyanobium PCC-6307;Ambiguous_taxa
456a148265a71b0a6473e484b8317f14	18	1.00	D_0__Bacteria;D_1__Spirochaetes;D_2__Spirochaetia;D_3__Spirochaetales;D_4__Spirochaetaceae;D_5__uncultured;D_6__uncultured organism
5979210de5db54bf822e7613aa2f3816	17	0.92	D_0__Bacteria;D_1__Proteobacteria;D_2__Alphaproteobacteria;D_3__Rhodospirillales;D_4__AEGEAN-169 marine group;D_5__marine metagenome;D_6__marine metagenome
1924cc7353f356c38eaf0d3050f1c921	17	1.00	D_0__Bacteria;D_1__Dadabacteria;D_2__Dadabacteriia;D_3__Dadabacteriales;D_4__metagenome;D_5__metagenome;D_6__metagenome
c96901821294d55d69882aaf4b3bc0cf	17	0.83	D_0__Bacteria;D_1__Proteobacteria;D_2__Gammaproteobacteria;D_3__UBA10353 marine group;Ambiguous_taxa;Ambiguous_taxa;Ambiguous_taxa
d40157f7485eb59eb44fc893d4a750bd	16	1.00	D_0__Bacteria;D_1__Proteobacteria;D_2__Deltaproteobacteria;D_3__SAR324 clade(Marine group B)
f0e40f6447eb6df965fbc0cacc7d0df4	15	1.00	D_0__Bacteria;D_1__Proteobacteria;D_2__Gammaproteobacteria;D_3__Ectothiorhodospirales;D_4__Ectothiorhodospiraceae;D_5__uncultured;Ambiguous_taxa
7f597b7fcab2816523cf501954d923f7	15	1.00	D_0__Bacteria;D_1__Verrucomicrobia;D_2__Verrucomicrobiae;D_3__Arctic97B-4 marine group;Ambiguous_taxa;Ambiguous_taxa;Ambiguous_taxa
b50985bf9adcb1c8b7bcfec5356836a9	14	0.99	D_0__Bacteria;D_1__Bacteroidetes;D_2__Bacteroidia;D_3__Flavobacteriales;D_4__Cryomorphaceae;D_5__uncultured;D_6__uncultured Flavobacteriia bacterium
d7ffd5e2497c350d9bbbc252bd02abb0	14	0.87	D_0__Bacteria;D_1__Proteobacteria;D_2__Alphaproteobacteria;D_3__SAR11 clade;D_4__Clade III;Ambiguous_taxa;Ambiguous_taxa

182748248caeebe548c74f3f983287a9	14	1.00	D_0__Bacteria;D_1__Proteobacteria;D_2__Alphaproteobacteria;D_3__Rhodobacterales;D_4__Rhodobacteraceae
197dde00f50b4dd406d29015e19ab32f	14	0.88	D_0__Bacteria;D_1__Proteobacteria;D_2__Alphaproteobacteria;D_3__uncultured;D_4__uncultured organism;D_5__uncultured organism;D_6__uncultured organism
59806ef23b797cc8231682e991e4fdd9	14	0.85	D_0__Bacteria;D_1__Proteobacteria;D_2__Gammaproteobacteria;D_3__KI89A clade;D_4__uncultured bacterium;D_5__uncultured bacterium;D_6__uncultured bacterium
7ef19ba913ee9b7507e46a47b18be808	14	0.99	D_0__Bacteria;D_1__Actinobacteria;D_2__Acidimicrobiia;D_3__Microtrichales;D_4__Microtrichaceae;D_5__Sva099 6 marine group;Ambiguous_taxa
d1da42df41a34be6728944ea57a4d3ad	13	1.00	D_0__Bacteria;D_1__Proteobacteria;D_2__Alphaproteobacteria;D_3__SAR11 clade;D_4__Clade I
d2fab9a12f3e457f82bd71938de0aa55	13	0.93	D_0__Bacteria;D_1__Proteobacteria;D_2__Deltaproteobacteria;D_3__SAR324 clade(Marine group B);D_4__uncultured bacterium;D_5__uncultured bacterium;D_6__uncultured bacterium
938103f52294f55873a6fab0eb577862	13	0.99	D_0__Bacteria;D_1__Proteobacteria;D_2__Gammaproteobacteria;D_3__Cellvibrionales;D_4__Halieaceae;D_5__OM6 0(NOR5) clade
80735d5437c384ca28b980037b8667fd	13	0.89	D_0__Bacteria;D_1__Marinimicrobia (SAR406 clade);D_2__uncultured marine bacterium;D_3__uncultured marine bacterium;D_4__uncultured marine bacterium;D_5__uncultured marine bacterium;D_6__uncultured marine bacterium
e404eef661d3b59265eff891509f027b	13	1.00	D_0__Bacteria;D_1__Proteobacteria;D_2__Alphaproteobacteria;D_3__Rickettsiales;D_4__S25-593
dafa30457fd5c45dd84d0943005593b5	13	1.00	D_0__Bacteria;D_1__Proteobacteria;D_2__Gammaproteobacteria;D_3__Ga0077536;Ambiguous_taxa;Ambiguous_taxa ;Ambiguous_taxa
dd291ffd9b6ed2ae8fb6471423d4017b	13	0.75	D_0__Bacteria;D_1__Cyanobacteria;D_2__Oxyphotobacteria;D_3__Synechococcales;D_4__Cyanobiaceae;D_5__Proc hlorococcus MIT9313;D_6__uncultured marine cyanobacterium
66edcabc3197f5d3b3bfd40d13205011	12	1.00	D_0__Bacteria;D_1__Proteobacteria;D_2__Alphaproteobacteria;D_3__Rhodospirillales;D_4__AEGEAN-169 marine group;Ambiguous_taxa;Ambiguous_taxa
6fdb2876b8fe6afdd319e899f73b2a26	12	0.83	D_0__Bacteria;D_1__Proteobacteria;D_2__Gammaproteobacteria;D_3__Arenicellales;D_4__Arenicellaceae;D_5__unc ultured;D_6__uncultured bacterium

9285fb928534b86aad25a8862cd9dc0c	12	1.00	D_0__Bacteria;D_1__Proteobacteria;D_2__Alphaproteobacteria;D_3__Parvibaculales;D_4__Parvibaculaceae;D_5__uncultured;Ambiguous_taxa
3c054bdcd17f565efb12af7c23ce6841	12	0.97	D_0__Bacteria;D_1__Proteobacteria;D_2__Gammaproteobacteria;D_3__Oceanospirillales;D_4__Endozoicomonadaceae;D_5__Endozoicomonas;Ambiguous_taxa
687b5222eb243abcf25353185e82c95d	12	0.84	D_0__Bacteria;D_1__Proteobacteria;D_2__Alphaproteobacteria;D_3__Parvibaculales;D_4__PS1 clade;Ambiguous_taxa;Ambiguous_taxa
37125a6ab96e7774c0fcfcef253e3d61	12	1.00	D_0__Bacteria;D_1__Verrucomicrobia;D_2__Verrucomicrobiae;D_3__Opitutales;D_4__Puniceicoccaceae
b279ffd1945c774864ba214a0cd2c58b	12	0.99	D_0__Bacteria;D_1__Proteobacteria;D_2__Alphaproteobacteria;D_3__Puniceispirillales;D_4__SAR116 clade;Ambiguous_taxa;Ambiguous_taxa
086ff877afdcfb48e7451b8839729953	11	1.00	D_0__Bacteria;D_1__Bacteroidetes;D_2__Bacteroidia;D_3__Flavobacteriales;D_4__Flavobacteriaceae;D_5__NS5 marine group
840577c29f59301756b6321bceefa3af	11	1.00	D_0__Bacteria;D_1__Verrucomicrobia;D_2__Verrucomicrobiae;D_3__Pedosphaerales;D_4__Pedosphaeraceae;D_5__SCGC AAA164-E04;Ambiguous_taxa
cec03da2d8ccd118a1269bd00eb876be	11	1.00	D_0__Bacteria;D_1__Bacteroidetes;D_2__Bacteroidia;D_3__Flavobacteriales;D_4__Flavobacteriaceae;D_5__NS4 marine group
a1847e583dfd99b4464b0c36f4f75152	11	1.00	D_0__Bacteria;D_1__Proteobacteria;D_2__Alphaproteobacteria;D_3__uncultured;D_4__unidentified marine bacterioplankton;D_5__unidentified marine bacterioplankton;D_6__unidentified marine bacterioplankton
ea3073d39b46c87a002110d3d80ed631	11	0.78	D_0__Bacteria;D_1__Proteobacteria;D_2__Gammaproteobacteria;D_3__Alteromonadales;D_4__Alteromonadaceae;D_5__Glaciecola;Ambiguous_taxa
3a65fb6af65a4c3d160edc25f67d381d	11	0.98	D_0__Bacteria;D_1__Proteobacteria;D_2__Deltaproteobacteria;D_3__Desulfovibrionales;D_4__Desulfovibrionaceae;D_5__Halodesulfovibrio;Ambiguous_taxa
e91ea8cc49fbe2403e5fcbd484f6a875	11	1.00	D_0__Bacteria;D_1__Proteobacteria;D_2__Alphaproteobacteria;D_3__uncultured
0f5177bf3f4f3fd5dbc3d1d49171e34d	11	0.97	D_0__Bacteria;D_1__Proteobacteria;D_2__Gammaproteobacteria;D_3__Oceanospirillales;D_4__Endozoicomonadaceae;D_5__Endozoicomonas;Ambiguous_taxa

65a27ba6edc1d275d2ecafd787a21198	10	1.00	D_0__Bacteria
0e028214895602d4967e7f6f2d04d0a9	10	1.00	D_0__Bacteria;D_1__Proteobacteria;D_2__Alphaproteobacteria;D_3__Rhizobiales;D_4__Stappiaceae;D_5__Pseudovibrio;Ambiguous_taxa
d85456cdcc158131c1d6624e6cc3e0cb	10	1.00	D_0__Bacteria;D_1__Proteobacteria;D_2__Gammaproteobacteria;D_3__SAR86 clade;Ambiguous_taxa;Ambiguous_taxa;Ambiguous_taxa
bbb3078cac73ba22fb709fdbd8bb551c	10	1.00	D_0__Bacteria;D_1__Verrucomicrobia;D_2__Verrucomicrobiae;D_3__Verrucomicrobiales;D_4__Rubritaleaceae;D_5__Rubritalea;D_6__uncultured bacterium
7117f1efa85e803f054b2ca25d401720	10	1.00	D_0__Bacteria;D_1__Chloroflexi;D_2__Dehalococcoidia;D_3__SAR202 clade;Ambiguous_taxa;Ambiguous_taxa;Ambiguous_taxa
ebb6ab1507d93698f804c246b1020a3c	10	0.93	D_0__Bacteria;D_1__Proteobacteria;D_2__Alphaproteobacteria;D_3__Rhodospirillales;D_4__AEGEAN-169 marine group;D_5__uncultured marine bacterium;D_6__uncultured marine bacterium
4eed5c4b88ade1b1b22e423ffd870e61	10	1.00	D_0__Bacteria;D_1__Verrucomicrobia;D_2__Verrucomicrobiae;D_3__Verrucomicrobiales;D_4__DEV007;Ambiguous_taxa;Ambiguous_taxa
bac6aa885e6c08501a46e043cfced81b	10	0.99	D_0__Archaea;D_1__Euryarchaeota;D_2__Thermoplasmata;D_3__Marine Group II;Ambiguous_taxa;Ambiguous_taxa;Ambiguous_taxa
9ad993d4118381301289b0b4b89d56c2	10	0.81	D_0__Bacteria;D_1__Proteobacteria;D_2__Alphaproteobacteria;D_3__Rhodobacterales;D_4__Rhodobacteraceae;D_5__uncultured;D_6__Rhodobacteraceae bacterium REDSEA-S03_B4
8e8383cc0ca975bed2e4d5ba2840da59	10	0.78	D_0__Bacteria;D_1__Proteobacteria;D_2__Alphaproteobacteria;D_3__Rhodobacterales;D_4__Rhodobacteraceae;D_5__Ruegeria;D_6__uncultured bacterium
888dc141f4a7b821a4f2168efcf8208c	10	1.00	D_0__Bacteria;D_1__Proteobacteria;D_2__Gammaproteobacteria;D_3__SAR86 clade
224b523f51d30e1c8aebe636fdd76654	10	1.00	D_0__Bacteria;D_1__Verrucomicrobia;D_2__Verrucomicrobiae;D_3__Verrucomicrobiales;D_4__Rubritaleaceae;D_5__Rubritalea
7aa1817b824952245b72f7627ff71a0d	9	0.95	D_0__Bacteria;D_1__Bacteroidetes;D_2__Bacteroidia;D_3__Bacteroidales;D_4__Bacteroidaceae;D_5__Bacteroides;D_6__uncultured isopod gut bacterium

f441852fb48304760140fff7cf3354fc	9	0.77	D_0__Archaea;D_1__Euryarchaeota;D_2__Thermoplasmata;D_3__Marine Group II;D_4__uncultured marine archaeon;D_5__uncultured marine archaeon;D_6__uncultured marine archaeon
8db0caf5ee40271902d2be9e6661d66b	9	0.97	D_0__Bacteria;D_1__Proteobacteria;D_2__Gammaproteobacteria;D_3__Thiotrichales;D_4__Thiotrichaceae;D_5__uncultured;D_6__uncultured marine bacterium
eb1f5ea9269dd4c0cba4969234eca5ac	9	1.00	D_0__Bacteria;D_1__Bacteroidetes;D_2__Bacteroidia;D_3__Flavobacteriales;D_4__Cryomorphaceae;D_5__uncultured
3db69d1784046c5733ebbcbf0227f3ec	9	0.93	D_0__Bacteria;D_1__Proteobacteria;D_2__Alphaproteobacteria;D_3__Rhodospirillales;D_4__AEGEAN-169 marine group;D_5__uncultured marine bacterium;D_6__uncultured marine bacterium
e52e87ffd72f37f4aa13f3f56ee8653f	9	0.77	D_0__Bacteria;D_1__Proteobacteria;D_2__Alphaproteobacteria;D_3__Rhodospirillales;D_4__Terasakiellaceae;D_5__uncultured;D_6__uncultured alpha proteobacterium
82935983fcf33e48819f10799c7f4166	9	1.00	D_0__Bacteria;D_1__Planctomycetes;D_2__Pla3 lineage;Ambiguous_taxa;Ambiguous_taxa;Ambiguous_taxa;Ambiguous_taxa
68eca3f2dff9f80124509a680eb0b97e	9	0.92	D_0__Bacteria;D_1__Bacteroidetes;D_2__Bacteroidia;D_3__Flavobacteriales;D_4__Flavobacteriaceae;D_5__NS5 marine group;Ambiguous_taxa
839b77f49e66e33cba1b7dcb4b867095	9	0.84	D_0__Bacteria;D_1__Bacteroidetes;D_2__Bacteroidia;D_3__Flavobacteriales;D_4__Flavobacteriaceae;D_5__NS5 marine group;D_6__uncultured Flavobacteriaceae bacterium
0db13ee94d8215e68ce5b2d2d41a8fa2	9	0.99	D_0__Bacteria;D_1__Proteobacteria;D_2__Gammaproteobacteria;D_3__Cellvibrionales;D_4__Halieaceae;D_5__OM60(NOR5) clade;Ambiguous_taxa
12c00043e5fc6e36eb6f9b9ff5570844	9	0.93	D_0__Bacteria;D_1__Actinobacteria;D_2__Acidimicrobiia;D_3__Actinomarinales;D_4__Actinomarinaceae;D_5__Candidatus Actinomarina;D_6__uncultured marine bacterium
136add0dacfdabe3814008cf60e17cdb	9	0.86	D_0__Bacteria;D_1__Planctomycetes;D_2__Planctomycetacia;D_3__Planctomycetales;D_4__Gimesiaceae;D_5__uncultured;D_6__uncultured Planctomyces sp.
6598bf8b035cf4c390b8bec3d94cfdf2	8	0.82	D_0__Bacteria;D_1__Firmicutes;D_2__Clostridia;D_3__Clostridiales;D_4__Peptostreptococcaceae;D_5__Paeniclostridium;D_6__uncultured bacterium
56897a07a28b91d2f5d521554cab3af1	8	0.76	D_0__Bacteria;D_1__Bacteroidetes;D_2__Bacteroidia;D_3__Flavobacteriales;D_4__Flavobacteriaceae;D_5__NS5 marine group;D_6__uncultured marine bacterium

b42d6f1f6528e4ecbd238930dbbf8b0a	8	0.99	D_0__Bacteria;D_1__Proteobacteria;D_2__Alphaproteobacteria;D_3__Puniceispirillales;D_4__SAR116 clade;D_5__marine metagenome;D_6__marine metagenome
432112989bc7deb540adfca2b8ba1fe8	8	0.89	D_0__Bacteria;D_1__Marinimicrobia (SAR406 clade);D_2__uncultured marine bacterium;D_3__uncultured marine bacterium;D_4__uncultured marine bacterium;D_5__uncultured marine bacterium;D_6__uncultured marine bacterium
27a7395fbdf178788723b1cf90ca9ef1	8	1.00	D_0__Bacteria;D_1__Proteobacteria;D_2__Gammaproteobacteria;D_3__Betaproteobacteriales;D_4__EC94
f54b98b1d131a1568071a797f89ca94a	8	0.99	D_0__Bacteria;D_1__Proteobacteria;D_2__Alphaproteobacteria
825c9dee653fdfd80658fc84bb9d654e	8	1.00	D_0__Bacteria;D_1__Bacteroidetes;D_2__Bacteroidia;D_3__Flavobacteriales;D_4__Flavobacteriaceae;D_5__NS5 marine group
624f6b19c1a893d5b6f64571a931fe60	8	0.75	D_0__Bacteria;D_1__Proteobacteria;D_2__Deltaproteobacteria;D_3__Bdellovibrionales;D_4__Bdellovibrionaceae;D_5__OM27 clade;D_6__uncultured bacterium
3ac4a00c18fc225d20f21690431a8acb	8	1.00	D_0__Bacteria;D_1__Marinimicrobia (SAR406 clade);D_2__uncultured marine bacterium;D_3__uncultured marine bacterium;D_4__uncultured marine bacterium;D_5__uncultured marine bacterium;D_6__uncultured marine bacterium
4df7e9e016e573b5393280f7025756dc	8	0.97	D_0__Bacteria;D_1__Proteobacteria;D_2__Deltaproteobacteria;D_3__PB19;Ambiguous_taxa;Ambiguous_taxa;Ambiguous_taxa
746e7eb53d73ef58d026df56c2f2bf44	8	0.96	D_0__Bacteria;D_1__Proteobacteria;D_2__Gammaproteobacteria;D_3__Cellvibrionales;D_4__Halieaceae;D_5__OM60(NOR5) clade;Ambiguous_taxa
b52363e24166237ed0022e0c8757b444	8	1.00	D_0__Bacteria;D_1__Bacteroidetes;D_2__Bacteroidia;D_3__Flavobacteriales;D_4__Flavobacteriaceae;D_5__Spongiiferula;D_6__Spongiiferula fulva
774f7293364d9c07219111b380457d73	8	0.86	D_0__Bacteria;D_1__Bacteroidetes;D_2__Bacteroidia;D_3__Flavobacteriales;D_4__NS7 marine group;D_5__uncultured organism;D_6__uncultured organism
f90c3a689268b9aa6c5ec593d1678b74	8	1.00	D_0__Bacteria;D_1__Proteobacteria;D_2__Gammaproteobacteria
f1509ea9bc1703d313e377ade95f134f	7	1.00	D_0__Bacteria;D_1__Actinobacteria;D_2__Acidimicrobiia;D_3__Microtrichales;D_4__Ilumatobacteraceae
6944ba506d930cf0d8417ab1bcb8a024	7	0.97	D_0__Bacteria;D_1__Bacteroidetes;D_2__Bacteroidia;D_3__Flavobacteriales;D_4__Flavobacteriaceae;D_5__NS5 marine group;D_6__uncultured Flavobacteriia bacterium

6cad95b371c104106918bf8d6f168082	7	1.00	D_0__Bacteria;D_1__Dadabacteria;D_2__Dadabacteriia;D_3__Dadabacteriales;D_4__metagenome;D_5__metagenome;D_6__metagenome
ab8d766cdbff59ddfaf02d91abf83f1c	7	0.88	D_0__Bacteria;D_1__Planctomycetes;D_2__Planctomycetacia;D_3__Pirellulales;D_4__Pirellulaceae;D_5__uncultured
5599728dd55f74e0c82aeec0fe3050b3	7	1.00	D_0__Bacteria;D_1__Chlamydiae;D_2__Chlamydiae;D_3__Chlamydiales;D_4__Waddliaceae;D_5__Waddlia
171df6b908553651947ec8519a95221c	7	0.83	D_0__Bacteria;D_1__Proteobacteria;D_2__Alphaproteobacteria;D_3__Rickettsiales;D_4__S25-593;Ambiguous_taxa;Ambiguous_taxa
10aac04c1f389e8e08f371b55cfad890	7	1.00	D_0__Bacteria;D_1__Proteobacteria;D_2__Gammaproteobacteria;D_3__Betaproteobacteriales;D_4__Nitrosomonadaceae;D_5__IS-44;D_6__uncultured marine bacterium
037e17b163e042a3d5e8185bf096b274	7	0.86	D_0__Bacteria;D_1__Proteobacteria;D_2__Alphaproteobacteria;D_3__Rhodobacterales;D_4__Rhodobacteraceae;D_5__Ascidiaceihabitans;Ambiguous_taxa
164c9bee1a5cfddabb139c168a12af7a	7	0.94	D_0__Bacteria;D_1__Proteobacteria;D_2__Gammaproteobacteria;D_3__Oceanospirillales;D_4__Endozoicomonadaceae;D_5__Endozoicomonas;Ambiguous_taxa
09887b95245287b93eeae64fd436e809	7	0.84	D_0__Bacteria;D_1__Proteobacteria;D_2__Alphaproteobacteria;D_3__Rhodospirillales;D_4__Magnetospiraceae;D_5__uncultured
46bc30ed87b36c540e2d15ade4995aeb	7	1.00	D_0__Bacteria;D_1__Chloroflexi;D_2__Dehalococcoidia;D_3__SAR202 clade;Ambiguous_taxa;Ambiguous_taxa;Ambiguous_taxa
6706a0021d0e5765b6c42a6a4dc73965	6	1.00	D_0__Bacteria;D_1__Proteobacteria;D_2__Gammaproteobacteria;D_3__SAR86 clade
e926e94396f51a387cb442c3b69721b2	6	1.00	D_0__Bacteria;D_1__Bacteroidetes;D_2__Bacteroidia;D_3__Flavobacteriales;D_4__Flavobacteriaceae;D_5__NS4 marine group;D_6__uncultured marine bacterium
3e60e3be6aff719e028c80bc92e45a45	6	1.00	D_0__Bacteria;D_1__Proteobacteria;D_2__Alphaproteobacteria;D_3__Puniceispirillales;D_4__uncultured
ff7cf18f937806f6eb27bb065a2ae716	6	0.99	D_0__Bacteria;D_1__Spirochaetes;D_2__Leptospirae;D_3__Leptospirales;D_4__Leptospiraceae;D_5__Turneriella
8f47f79d697ded71a080ddd078f6497b	6	0.90	D_0__Bacteria;D_1__Proteobacteria;D_2__Gammaproteobacteria;D_3__SAR86 clade;Ambiguous_taxa;Ambiguous_taxa;Ambiguous_taxa

7b2a3d1f99a616e3b7c14a70ee49a64d	6	0.96	D_0__Bacteria;D_1__Planctomycetes;D_2__OM190;Ambiguous_taxa;Ambiguous_taxa;Ambiguous_taxa;Ambiguous_taxa
6da6b7933913af0123a84fbba0781031	6	1.00	D_0__Bacteria;D_1__Bacteroidetes;D_2__Bacteroidia;D_3__Flavobacteriales;D_4__Cryomorphaceae;D_5__uncultured;D_6__uncultured Owenweeksia sp.
a3c99905dd8b0fd8d0a3bcfe9aed6ade	6	1.00	D_0__Bacteria;D_1__Chloroflexi;D_2__Anaerolineae;D_3__SBR1031;D_4__A4b
3d7c227af88a5fbe40eea964958e91f1	6	1.00	D_0__Bacteria;D_1__Bacteroidetes;D_2__Bacteroidia;D_3__Flavobacteriales;D_4__Crocinitomicaceae;D_5__Crocinitomix;D_6__uncultured bacterium
db728f19676f1a00ca637cc7d62e192d	6	0.77	D_0__Bacteria;D_1__Bacteroidetes;D_2__Bacteroidia;D_3__Flavobacteriales;D_4__Cryomorphaceae;D_5__NS10 marine group;D_6__uncultured marine bacterium
33c8d62dfb001123078685165efd5604	6	1.00	D_0__Bacteria;D_1__Verrucomicrobia;D_2__Verrucomicrobiae;D_3__Verrucomicrobiales;D_4__Rubritaleaceae;D_5__Roseibacillus
28137f1dd5f924174ad5a633288380f9	6	0.86	D_0__Bacteria;D_1__Proteobacteria;D_2__Deltaproteobacteria;D_3__NB1-j;Ambiguous_taxa;Ambiguous_taxa;Ambiguous_taxa
7c84d830a6c259b700fe2c6b59bc629b	6	1.00	D_0__Bacteria;D_1__Planctomycetes;D_2__Phycisphaerae;D_3__Phycisphaerales;D_4__Phycisphaeraceae;D_5__CL500-3;Ambiguous_taxa
700eda817b20c82932b59ffa0c312991	6	0.86	D_0__Bacteria;D_1__Bacteroidetes;D_2__Bacteroidia;D_3__Flavobacteriales;D_4__Flavobacteriaceae;D_5__NS2b marine group
00bedf3112e1c835b9c2e48f9c1d3b05	6	0.98	D_0__Bacteria;D_1__Firmicutes;D_2__Erysipelotrichia;D_3__Erysipelotrichales;D_4__Erysipelotrichaceae;D_5__Turicibacter;D_6__uncultured bacterium
dc28edbddc3b74802735beba8990dbb7	5	1.00	D_0__Bacteria;D_1__Bacteroidetes;D_2__Bacteroidia;D_3__Flavobacteriales;D_4__Flavobacteriaceae;D_5__uncultured;D_6__uncultured Flavobacteriia bacterium
d7cd41e6f5765d6c01a994195503ebc5	5	0.94	D_0__Bacteria
ce6da91c6e5c0c23bad4cd7d45e96af5	5	1.00	D_0__Bacteria;D_1__Proteobacteria;D_2__Gammaproteobacteria;D_3__Aeromonadales;D_4__Aeromonadaceae;D_5__Tolumonas

c0f66e9f69d1611262d18297ddf7cb9b	5	0.98	D_0__Bacteria;D_1__Firmicutes;D_2__Clostridia;D_3__Clostridiales;D_4__Lachnospiraceae;D_5__Blautia
6c8879c36583e16ec4cea6a8803f170f	5	1.00	D_0__Bacteria;D_1__Proteobacteria;D_2__Gammaproteobacteria;D_3__Diplorickettsiales;D_4__Diplorickettsiaceae;D_5__Rickettsiella;Ambiguous_taxa
fd81101a9c42619cdaec84f55b6f7a54	5	1.00	D_0__Bacteria;D_1__Patescibacteria;D_2__Gracilibacteria
85b74756e32be994bef1f689fee42e83	5	1.00	D_0__Bacteria;D_1__Proteobacteria;D_2__Deltaproteobacteria;D_3__Myxococcales;D_4__Blfdi19;Ambiguous_taxa;Ambiguous_taxa
aa4196dd541291014fd3bced33426c20	5	0.75	D_0__Bacteria;D_1__Proteobacteria;D_2__Alphaproteobacteria;D_3__Rickettsiales;D_4__S25-593;Ambiguous_taxa;Ambiguous_taxa
3a58196eed5a570fe7565d97c2633593	5	0.88	D_0__Bacteria;D_1__Firmicutes;D_2__Bacilli;D_3__Lactobacillales;D_4__Carnobacteriaceae;D_5__Trichococcus
a77527210533790ea959a9c938293889	5	0.95	D_0__Bacteria;D_1__Bacteroidetes;D_2__Bacteroidia;D_3__Flavobacteriales;D_4__NS9 marine group;Ambiguous_taxa;Ambiguous_taxa
53fcd0b8ff2970bfb6dfdc9b81f8a494	5	1.00	D_0__Bacteria;D_1__Actinobacteria;D_2__Actinobacteria;D_3__Bifidobacteriales;D_4__Bifidobacteriaceae;D_5__Bifidobacterium
3638eeebaf4b2ea458c274607042b258	5	0.78	D_0__Bacteria;D_1__Proteobacteria;D_2__Alphaproteobacteria;D_3__uncultured;D_4__uncultured bacterium;D_5__uncultured bacterium;D_6__uncultured bacterium
76aadd750ce17c262636e7b8c415f113	5	0.97	D_0__Bacteria;D_1__Proteobacteria;D_2__Gammaproteobacteria;D_3__OM182 clade;Ambiguous_taxa;Ambiguous_taxa;Ambiguous_taxa
e518942223b955e3aad55d1e9f4f881b	5	1.00	D_0__Bacteria;D_1__Fusobacteria;D_2__Fusobacteriia;D_3__Fusobacteriales;D_4__Leptotrichiaceae;D_5__Hypnocyclicus;D_6__Hypnocyclicus thermotrophus
b0489551c28fbb52342a7a70bf8924a8	5	1.00	D_0__Bacteria;D_1__Bacteroidetes;D_2__Bacteroidia;D_3__Flavobacteriales;D_4__NS9 marine group;D_5__hydrothermal vent metagenome;D_6__hydrothermal vent metagenome
529f28cd0f2d6bdb5d24e75819e4652f	5	0.75	D_0__Bacteria;D_1__Planctomycetes;D_2__Planctomycetacia;D_3__Planctomycetales;D_4__Rubinisphaeraceae;D_5__uncultured;Ambiguous_taxa
d001da55fd721b9ea319c83fe1164eee	4	1.00	D_0__Bacteria;D_1__Chloroflexi;D_2__Dehalococcoidia;D_3__SAR202 clade

e2cdc815cffd5deb557641052675f1f7	4	1.00	D_0__Bacteria;D_1__Bacteroidetes;D_2__Rhodothermia;D_3__Balneolales;D_4__Balneolaceae;D_5__Balneola
a9759aed115583af48981c771e9090b2	4	0.96	D_0__Bacteria;D_1__Proteobacteria;D_2__Gammaproteobacteria;D_3__KI89A clade;D_4__uncultured bacterium;D_5__uncultured bacterium;D_6__uncultured bacterium
6fcf48c1eb201de677bbb96f4732c80e	4	1.00	D_0__Bacteria;D_1__Proteobacteria;D_2__Alphaproteobacteria;D_3__Rhodospirillales;D_4__Magnetospiraceae
6c8deb257a2cf51bca6a4e798c242d33	4	1.00	D_0__Archaea;D_1__Euryarchaeota;D_2__Thermoplasmata;D_3__Marine Group II
cf05c93b9617d4018855b629c9676330	4	0.97	D_0__Bacteria
714a80cd29386538156c342df2243859	4	0.77	D_0__Bacteria;D_1__Bacteroidetes;D_2__Bacteroidia;D_3__Flavobacteriales;D_4__Flavobacteriaceae;D_5__NS5 marine group;Ambiguous_taxa
5e5c53c341c4c13a95f057bb74d5308a	4	0.97	D_0__Bacteria;D_1__Bacteroidetes;D_2__Bacteroidia;D_3__Flavobacteriales;D_4__NS9 marine group;D_5__uncultured marine bacterium;D_6__uncultured marine bacterium
56fee19e83a98199e7f556e0bea810ee	4	1.00	D_0__Bacteria;D_1__Actinobacteria;D_2__Actinobacteria;D_3__PeM15
0edd7ef25768409332e68f9fa7e49d5c	4	0.81	D_0__Bacteria;D_1__Proteobacteria;D_2__Deltaproteobacteria;D_3__NB1-j;Ambiguous_taxa;Ambiguous_taxa;Ambiguous_taxa
c9f58d334849b39336760d15a9e151c4	4	0.76	D_0__Bacteria;D_1__Lentisphaerae;D_2__Lentisphaeria;D_3__Lentisphaerales;D_4__Lentisphaeraceae;D_5__Lentisphaera;D_6__uncultured organism
7adebb0e196ec6f800e7ea931e737f72	4	0.92	D_0__Archaea;D_1__Euryarchaeota;D_2__Thermoplasmata;D_3__Marine Group III;Ambiguous_taxa;Ambiguous_taxa;Ambiguous_taxa
2a16f0a6af742321c959858f489f31ee	4	1.00	D_0__Bacteria;D_1__Chloroflexi;D_2__Dehalococcoidia;D_3__SAR202 clade;Ambiguous_taxa;Ambiguous_taxa;Ambiguous_taxa
89975fd7054c79c070e44e117914a7be	4	0.99	D_0__Bacteria;D_1__Proteobacteria;D_2__Gammaproteobacteria;D_3__EPR3968-O8a-Bc78;D_4__uncultured gamma proteobacterium;D_5__uncultured gamma proteobacterium;D_6__uncultured gamma proteobacterium
c0b0d4653d56783a6aab1f2cc47f68d1	4	1.00	D_0__Bacteria;D_1__Planctomycetes;D_2__Phycisphaerae;D_3__Phycisphaerales;D_4__Phycisphaeraceae;D_5__FS140-16B-02 marine group;Ambiguous_taxa

e52209f8597c953aa3d9187e03c073aa	4	1.00	D_0__Bacteria;D_1__Bacteroidetes;D_2__Bacteroidia;D_3__Flavobacteriales;D_4__NS7 marine group
1b6cd33b1edc0f704af1d0483addbbae	4	1.00	D_0__Bacteria;D_1__Proteobacteria;D_2__Alphaproteobacteria;D_3__Rickettsiales;D_4__S25-593
dcd1f91101452df464c3c5309faaca9f	4	1.00	D_0__Bacteria;D_1__Bacteroidetes;D_2__Bacteroidia;D_3__Flavobacteriales;D_4__Flavobacteriaceae
92f0e58df59682cce6cba5468237174b	4	1.00	D_0__Bacteria;D_1__Proteobacteria;D_2__Gammaproteobacteria;D_3__Cellvibrionales;D_4__Porticoccaceae;D_5__S AR92 clade;D_6__uncultured marine bacterium
0424feb01196f37963c60430271e958b	3	0.82	D_0__Bacteria;D_1__Chloroflexi;D_2__Dehalococcoidia;D_3__SAR202 clade;D_4__uncultured Chloroflexi bacterium;D_5__uncultured Chloroflexi bacterium;D_6__uncultured Chloroflexi bacterium
9f794e999f1505e8686c67d2126a6706	3	1.00	D_0__Bacteria;D_1__Verrucomicrobia;D_2__Verrucomicrobiae;D_3__Opitutales;D_4__Puniceicoccaceae;D_5__Pelag icoccus
61b0e57b514d46f3098fd68476626a39	3	1.00	D_0__Bacteria;D_1__Proteobacteria;D_2__Deltaproteobacteria;D_3__Bdellovibrionales;D_4__Bdellovibrionaceae;D_5 __Bdellovibrio;D_6__uncultured delta proteobacterium
01c41dc4dc1756e408ec6f40be04acba	3	0.98	D_0__Bacteria;D_1__Proteobacteria;D_2__Deltaproteobacteria;D_3__PB19;D_4__uncultured marine bacterium;D_5__uncultured marine bacterium;D_6__uncultured marine bacterium
7c040b9adb9d37992100c7b7c885b662	3	0.98	D_0__Bacteria;D_1__Planctomycetes;D_2__Planctomycetacia;D_3__Pirellulales;D_4__Pirellulaceae;D_5__Blastopirel lula;D_6__uncultured marine bacterium
0b970a82afc8ad88bdafd649c0b36447	3	0.87	D_0__Bacteria;D_1__Proteobacteria;D_2__Gammaproteobacteria;D_3__Steroidobacterales;D_4__Woeseiaceae;D_5__ Woeseia;D_6__uncultured marine bacterium
775413fe8f6d8f9c0cd429d02ce184cc	3	1.00	D_0__Bacteria;D_1__Planctomycetes;D_2__Planctomycetacia;D_3__Pirellulales;D_4__Pirellulaceae;D_5__Rhodopirel lula;D_6__uncultured marine microorganism
e8d03e74305a5c384aeff861bebfabbc	3	1.00	D_0__Bacteria;D_1__Chloroflexi;D_2__Dehalococcoidia;D_3__SAR202 clade
b0ca840a930beaf6970a659c2b537c5f	3	1.00	D_0__Bacteria;D_1__Actinobacteria;D_2__Actinobacteria;D_3__Corynebacteriales;D_4__Mycobacteriaceae;D_5__M ycobacterium
95b55d9ec3425bc1c0c9d71072359252	3	0.91	D_0__Bacteria;D_1__Actinobacteria;D_2__Thermoleophilia;D_3__Solirubrobacterales;D_4__67-14;D_5__uncultured bacterium;D_6__uncultured bacterium

1496b348ef9912e566b567f2a7adaaef	3	0.95	D_0__Bacteria;D_1__Proteobacteria;D_2__Gammaproteobacteria;D_3__KI89A clade;D_4__uncultured bacterium;D_5__uncultured bacterium;D_6__uncultured bacterium
61d528498c4e8b9fde46b60fc8195af3	3	0.99	D_0__Bacteria;D_1__Bacteroidetes;D_2__Bacteroidia;D_3__Flavobacteriales;D_4__NS9 marine group;D_5__uncultured marine bacterium;D_6__uncultured marine bacterium
d5080af3bbc399a6385e04e39b866cc8	3	1.00	D_0__Bacteria;D_1__Chloroflexi;D_2__Dehalococcoidia;D_3__SAR202 clade;Ambiguous_taxa;Ambiguous_taxa;Ambiguous_taxa
f606f45ada87bd37234703bf9c5e15dc	2	0.96	D_0__Bacteria;D_1__Chloroflexi;D_2__Anaerolineae;D_3__Caldilineales;D_4__Caldilineaceae;D_5__uncultured;D_6__uncultured Caldilinea sp.
516232c17e43dc067b05d188e3d326bd	2	1.00	D_0__Bacteria;D_1__Spirochaetes;D_2__Spirochaetia;D_3__Spirochaetales;D_4__Spirochaetaceae;D_5__Spirochaeta 2;D_6__uncultured Spirochaetales bacterium
731ce5ad07ceb06ee24295e9fa112cc8	2	1.00	D_0__Archaea;D_1__Nanoarchaeaeota;D_2__Woesearchaeia
2be8e9546c675d391cd44d43ab7c3601	2	1.00	D_0__Bacteria;D_1__Proteobacteria;D_2__Deltaproteobacteria;D_3__Bdellovibrionales;D_4__Bdellovibrionaceae;D_5__OM27 clade
8fb1bf98c139fcaf28fafd463c8cbe33	2	0.95	D_0__Bacteria;D_1__Bacteroidetes;D_2__Bacteroidia;D_3__Bacteroidales;D_4__Prolixibacteraceae;D_5__Draconibacterium;D_6__uncultured bacterium
9584f7533a55b36f44e80486dc4519e1	2	1.00	D_0__Bacteria;D_1__Chlamydiae;D_2__Chlamydiae;D_3__Chlamydiales;D_4__cvE6
77dba9029170160a86f4e6080d831936	2	0.78	D_0__Bacteria;D_1__Firmicutes;D_2__Negativicutes;D_3__Selenomonadales;D_4__Veillonellaceae;D_5__Anaerosinus;D_6__uncultured bacterium
fc676af5f6d938de718e02bc11bf8f4a	2	0.97	D_0__Bacteria;D_1__Bacteroidetes;D_2__Bacteroidia;D_3__Chitinophagales;D_4__Saprospiraceae;D_5__uncultured; Ambiguous_taxa
860d91d2a158d184befda51e351e60aa	2	0.78	D_0__Bacteria;D_1__Bacteroidetes;D_2__Bacteroidia;D_3__Flavobacteriales;D_4__Weeksellaceae;D_5__Cloacibacterium;Ambiguous_taxa
423062c992e4453cf6a4bb33f465b0fb	2	0.84	D_0__Bacteria;D_1__Proteobacteria;D_2__Gammaproteobacteria;D_3__Oceanospirillales;D_4__Endozoicomonadaceae;D_5__Endozoicomonas;D_6__Endozoicomonas sp. Ez302

76facc81948c05417f538228c88144fa	2	0.98	D_0__Bacteria;D_1__Bacteroidetes;D_2__Bacteroidia;D_3__Bacteroidales;D_4__Marinilabiliaceae;D_5__uncultured;D_6__uncultured bacterium
4c0de77a89aa6dfe41a0894e69b94a64	2	1.00	D_0__Bacteria;D_1__Proteobacteria;D_2__Deltaproteobacteria;D_3__Bdellovibrionales;D_4__Bdellovibrionaceae;D_5__OM27 clade;Ambiguous_taxa
45719fa1483552c59e3eb48965674807	2	0.99	D_0__Bacteria;D_1__Planctomycetes;D_2__Planctomycetacia;D_3__Planctomycetales;D_4__Gimesiaceae;D_5__uncultured;Ambiguous_taxa
d88791e8c74769f38d6ffd30e1828cd8	2	0.94	D_0__Bacteria;D_1__Bacteroidetes;D_2__Bacteroidia;D_3__Bacteroidales;D_4__Marinifilaceae;D_5__uncultured;Ambiguous_taxa
4e86f444adb8c3d7aefe4298591898ca	2	1.00	D_0__Bacteria;D_1__Epsilonbacteraeota;D_2__Campylobacteria;D_3__Campylobacterales;D_4__Sulfurovaceae;D_5__Sulfurovum
48f0444bd797e7536ea0a79fd7bcb740	2	0.94	D_0__Bacteria;D_1__Chloroflexi;D_2__Anaerolineae;D_3__SBR1031;D_4__A4b;Ambiguous_taxa;Ambiguous_taxa
61a40bcded89032a27fbbbb431ee1d13	2	0.82	D_0__Bacteria;D_1__Bacteroidetes;D_2__Bacteroidia;D_3__Flavobacteriales;D_4__Flavobacteriaceae;D_5__uncultured;D_6__uncultured Bacteroidetes bacterium
06f793fbfe91d37ed9e8eb067a58f09c	2	0.98	D_0__Bacteria;D_1__Proteobacteria;D_2__Gammaproteobacteria;D_3__Alteromonadales;D_4__Pseudoalteromonadaceae;D_5__Pseudoalteromonas
5ed1e74bfd3855f132935655ae747be0	2	1.00	D_0__Bacteria;D_1__Proteobacteria;D_2__Alphaproteobacteria;D_3__Rhodospirillales;D_4__Magnetospiraceae;D_5__uncultured
a0f9ae2ff8f5d47511160fbc34ba624b	2	0.81	D_0__Bacteria;D_1__Bacteroidetes;D_2__Bacteroidia;D_3__Cytophagales
08d74e0fb374bcc4389c83fb8a3f087b	2	1.00	D_0__Bacteria;D_1__Proteobacteria;D_2__Gammaproteobacteria;D_3__Betaproteobacteriales;D_4__Burkholderiaceae
2b4edd8408ad973f4e17856f6449492a	2	0.80	D_0__Bacteria;D_1__Epsilonbacteraeota;D_2__Campylobacteria;D_3__Campylobacterales;D_4__Sulfurospirillaceae;D_5__Sulfurospirillum;Ambiguous_taxa
878ba67048b32eece2957f703569c4bd	1	0.94	D_0__Bacteria;D_1__Bacteroidetes;D_2__Bacteroidia;D_3__Flavobacteriales;D_4__NS7 marine group;D_5__seawater metagenome;D_6__seawater metagenome

4e1dc3bbc4e9605466c24730d6f073f9	1	0.93	D_0__Bacteria;D_1__Bacteroidetes;D_2__Bacteroidia;D_3__Flavobacteriales;D_4__NS7 marine group;D_5__seawater metagenome;D_6__seawater metagenome
d96ffb7c81abad77671ff382ecfa4cb4	1	0.87	D_0__Bacteria;D_1__Proteobacteria;D_2__Gammaproteobacteria;D_3__UBA10353 marine group;D_4__uncultured bacterium;D_5__uncultured bacterium;D_6__uncultured bacterium
1f48a92ba41a03177881bf49b5fc70e1	1	1.00	D_0__Bacteria;D_1__Proteobacteria;D_2__Gammaproteobacteria;D_3__Enterobacteriales;D_4__Enterobacteriaceae;D_5__Escherichia-Shigella
b673ed1b1ee53704ee2c82a593985eb6	1	0.99	D_0__Bacteria;D_1__Proteobacteria;D_2__Alphaproteobacteria;D_3__Puniceispirillales;D_4__SAR116 clade
65b78508474060868174e28f4487e64a	1	0.96	D_0__Bacteria;D_1__Proteobacteria;D_2__Gammaproteobacteria;D_3__Enterobacteriales;D_4__Enterobacteriaceae;D_5__Escherichia-Shigella
c47c499f0562f0120797fbbb580f26de	1	0.97	D_0__Bacteria;D_1__Bacteroidetes;D_2__Bacteroidia;D_3__Flavobacteriales;D_4__NS7 marine group;D_5__seawater metagenome;D_6__seawater metagenome
b51da3554ea7999a5d9cc35e3f151beb	1	0.92	D_0__Bacteria;D_1__Proteobacteria;D_2__Alphaproteobacteria;D_3__Rhodobacterales;D_4__Rhodobacteraceae
2ceaac4a8b39a8c2d5a8e611590ff137	1	1.00	D_0__Bacteria;D_1__Proteobacteria;D_2__Gammaproteobacteria;D_3__SAR86 clade
efcaeb6a3fbbf116a025ad1f9ffe7119	1	0.83	D_0__Bacteria;D_1__Proteobacteria;D_2__Gammaproteobacteria;D_3__EPR3968-O8a-Bc78;D_4__uncultured gamma proteobacterium;D_5__uncultured gamma proteobacterium;D_6__uncultured gamma proteobacterium
15bb0912ce0c9df80171d29c49293135	1	0.99	D_0__Bacteria;D_1__Proteobacteria;D_2__Gammaproteobacteria;D_3__Ga0077536;Ambiguous_taxa;Ambiguous_taxa;Ambiguous_taxa
b986a21c8e5352a260a5251a3801469e	1	0.99	D_0__Bacteria;D_1__Proteobacteria;D_2__Deltaproteobacteria;D_3__Bdellovibrionales;D_4__Bdellovibrionaceae;D_5__OM27 clade;Ambiguous_taxa

Table S11. ASVs (144) from *Geodia cydonium* with percentage of confidence ≥ 75%.

Feature ID	Number of sequences	Confidence	Taxon
916ed1e47f3d7f2371304b1a9420e533	187	1.00	D_0__Bacteria;D_1__Proteobacteria;D_2__Gammaproteobacteria;D_3__Oceanospirillales;D_4__Endozoicomonadaceae;D_5__Endozoicomonas
679aee879fd5868fad6e4b808c09c6d5	174	1.00	D_0__Bacteria;D_1__Poribacteria;Ambiguous_taxa;Ambiguous_taxa;Ambiguous_taxa;Ambiguous_taxa;Ambiguous_taxa
bc7ef34401f0e8edb858c24bd0a996f9	130	0.86	D_0__Bacteria;D_1__Nitrospirae;D_2__Nitrospira;D_3__Nitrospirales;D_4__Nitrospiraceae;D_5__Nitrospira;D_6__uncultured Nitrospirae bacterium
a1c32f7df86e1905de04f9b19aa20c04	119	0.97	D_0__Bacteria;D_1__Chloroflexi;D_2__Anaerolineae;D_3__Caldilineales;D_4__Caldilineaceae;D_5__uncultured;D_6__uncultured Chloroflexi bacterium
3f2736283599119e0c996167858814cb	119	1.00	D_0__Bacteria;D_1__Poribacteria;D_2__uncultured Clostridium sp.;D_3__uncultured Clostridium sp.;D_4__uncultured Clostridium sp.;D_5__uncultured Clostridium sp.;D_6__uncultured Clostridium sp.
056a34391a66945f09c782d54be93313	107	0.82	D_0__Bacteria;D_1__Chloroflexi;D_2__Dehalococcoidia;D_3__SAR202 clade;D_4__uncultured Chloroflexi bacterium;D_5__uncultured Chloroflexi bacterium;D_6__uncultured Chloroflexi bacterium
3b10ecbc96341c60b23081417b187109	101	0.85	D_0__Bacteria;D_1__Proteobacteria;D_2__Gammaproteobacteria;D_3__Oceanospirillales;D_4__Endozoicomonadaceae;D_5__Endozoicomonas;D_6__uncultured Spongiobacter sp.
40dd3ce434a77b9cb9cec052318a6dac	86	1.00	D_0__Bacteria;D_1__Chloroflexi;D_2__Dehalococcoidia;D_3__SAR202 clade
f34d1fbf32dc7b137af8bc422a131bb5	83	1.00	D_0__Bacteria;D_1__Chloroflexi;D_2__Dehalococcoidia;D_3__SAR202 clade
d1ae44a6ce6cb531cffa434a812cc1af	76	0.98	D_0__Bacteria;D_1__Dadabacteria;D_2__Dadabacteriia;D_3__Dadabacteriales;D_4__uncultured delta proteobacterium;D_5__uncultured delta proteobacterium;D_6__uncultured delta proteobacterium
23825c0d1018211d4b7750b1a12b57ee	73	1.00	D_0__Bacteria;D_1__Chloroflexi;D_2__Anaerolineae;D_3__Caldilineales;D_4__Caldilineaceae;D_5__uncultured

94765c8a51ad1b53c72cfa58a4e208e1	69	1.00	D_0__Bacteria;D_1__Poribacteria;D_2__uncultured Clostridium sp.;D_3__uncultured Clostridium sp.;D_4__uncultured Clostridium sp.;D_5__uncultured Clostridium sp.;D_6__uncultured Clostridium sp.
907a73ea999935824b0b2453969e99a6	68	0.85	D_0__Bacteria;D_1__Chloroflexi;D_2__Dehalococcoidia;D_3__SAR202 clade;D_4__uncultured Chloroflexi bacterium;D_5__uncultured Chloroflexi bacterium;D_6__uncultured Chloroflexi bacterium
82dd12ee65c6be6f27d3e3388e686e8c	60	0.94	D_0__Bacteria;D_1__Poribacteria;Ambiguous_taxa;Ambiguous_taxa;Ambiguous_taxa;Ambiguous_taxa;Ambiguous_ta xa
f9c2a75f1329dcc7a3ab77204ec61d5b	58	1.00	D_0__Bacteria;D_1__Chloroflexi;D_2__Dehalococcoidia;D_3__SAR202 clade
4d58056cc0ef38da08ac85896bfcbb22	54	1.00	D_0__Bacteria;D_1__Poribacteria;D_2__Candidatus Poribacteria bacterium WGA-4C;D_3__Candidatus Poribacteria bacterium WGA-4C;D_4__Candidatus Poribacteria bacterium WGA-4C;D_5__Candidatus Poribacteria bacterium WGA-4C;D_6__Candidatus Poribacteria bacterium WGA-4C
f6958d697a8701ca9a59190be974b17f	50	0.97	D_0__Bacteria;D_1__Chloroflexi;D_2__Dehalococcoidia;D_3__SAR202 clade;D_4__uncultured Chloroflexus sp.;D_5__uncultured Chloroflexus sp.;D_6__uncultured Chloroflexus sp.
281d6fc0312e2a305c9fed9d46af6a67	50	1.00	D_0__Bacteria;D_1__Chloroflexi;D_2__Dehalococcoidia;D_3__SAR202 clade
408b59118f65314c1c90b2b5f6a55dbb	46	1.00	D_0__Bacteria;D_1__Acidobacteria;D_2__Thermoanaerobaculia;D_3__Thermoanaerobaculales;D_4__Thermoanaerob aculaceae;D_5__Subgroup 10
cbe097b2843bf71d7e66ed46b947a58a	43	0.75	D_0__Bacteria;D_1__Proteobacteria;D_2__Gammaproteobacteria;D_3__HOC36;Ambiguous_taxa;Ambiguous_taxa;A mbiguous_taxa
31fff8d12b7f55a0547a4400045e7b5e	43	1.00	D_0__Bacteria;D_1__Chloroflexi;D_2__Dehalococcoidia;D_3__SAR202 clade
7b44eb00032d861a29316601d8131106	41	0.98	D_0__Bacteria;D_1__Proteobacteria;D_2__Gammaproteobacteria;D_3__JTB23;D_4__uncultured bacterium;D_5__uncultured bacterium;D_6__uncultured bacterium
6998e394d4d5675737a6f726d5be7fa4	39	1.00	D_0__Bacteria;D_1__Proteobacteria;D_2__Gammaproteobacteria;D_3__Oceanospirillales;D_4__Endozoicomonadacea e;D_5__Endozoicomonas
9eb1113ba4ee019ded2da2df8304a48e	37	1.00	D_0__Bacteria;D_1__Poribacteria;D_2__uncultured Clostridium sp.;D_3__uncultured Clostridium sp.;D_4__uncultured Clostridium sp.;D_5__uncultured Clostridium sp.;D_6__uncultured Clostridium sp.

63267e0e6c34bd62a338896a35f92d67	36	1.00	D_0__Bacteria;D_1__Poribacteria;Ambiguous_taxa;Ambiguous_taxa;Ambiguous_taxa;Ambiguous_taxa;Ambiguous_taxa
c3d64039bb8de7cd5356bcd64009997d	35	0.99	D_0__Bacteria;D_1__Chloroflexi;D_2__Dehalococcoidia;D_3__SAR202 clade;D_4__uncultured Chloroflexus sp.;D_5__uncultured Chloroflexus sp.;D_6__uncultured Chloroflexus sp.
c23c65705c648f0128ad57e637cc77bf	34	0.84	D_0__Bacteria;D_1__Chloroflexi;D_2__Dehalococcoidia;D_3__SAR202 clade;D_4__uncultured Chloroflexi bacterium;D_5__uncultured Chloroflexi bacterium;D_6__uncultured Chloroflexi bacterium
921a66099036cb00fc5d09439e9fb6d4	34	0.84	D_0__Bacteria;D_1__Chloroflexi;D_2__Dehalococcoidia;D_3__SAR202 clade;D_4__uncultured bacterium;D_5__uncultured bacterium;D_6__uncultured bacterium
5a58e98306ed49f6430672b5298e9d92	33	0.99	D_0__Bacteria;D_1__Chloroflexi;D_2__Dehalococcoidia;D_3__SAR202 clade;D_4__uncultured Chloroflexus sp.;D_5__uncultured Chloroflexus sp.;D_6__uncultured Chloroflexus sp.
38a61e329dff3bb748d774f116fcec8b	33	1.00	D_0__Bacteria;D_1__Chloroflexi;D_2__Dehalococcoidia;D_3__SAR202 clade
27de7f44fc541243575004a7c44852bd	32	1.00	D_0__Bacteria;D_1__Chloroflexi;D_2__Dehalococcoidia;D_3__SAR202 clade
fa0846f779fbf4f2903618e2dcc6f3c0	31	0.83	D_0__Bacteria;D_1__Proteobacteria;D_2__Deltaproteobacteria;D_3__Myxococcales;D_4__bacteriap25;Ambiguous_taxa;Ambiguous_taxa
e23c156642078fb48f89bc01faadac18	31	0.99	D_0__Bacteria;D_1__Poribacteria;Ambiguous_taxa;Ambiguous_taxa;Ambiguous_taxa;Ambiguous_taxa;Ambiguous_taxa
9101a4e6eae37bde17cf64fb7be21246	30	1.00	D_0__Bacteria;D_1__Acidobacteria;D_2__Acidobacteriia;D_3__Solibacterales;D_4__Solibacteraceae (Subgroup 3);D_5__PAUC26f;D_6__uncultured bacterium
b11fc4b8a2de0f534fe4a6599fa686e7	29	1.00	D_0__Bacteria;D_1__Proteobacteria;D_2__Gammaproteobacteria;D_3__JTB23;D_4__uncultured bacterium;D_5__uncultured bacterium;D_6__uncultured bacterium
f4f84ca1d604e3e067ac4c653b1bf2c5	28	0.86	D_0__Bacteria;D_1__Chloroflexi;D_2__Dehalococcoidia;D_3__SAR202 clade;D_4__uncultured Chloroflexi bacterium;D_5__uncultured Chloroflexi bacterium;D_6__uncultured Chloroflexi bacterium
d98ea7329c16ba02e782d47c4083cc11	28	0.99	D_0__Bacteria;D_1__Acidobacteria;D_2__Subgroup 11;Ambiguous_taxa;Ambiguous_taxa;Ambiguous_taxa;Ambiguous_taxa

48991897dd1ac07f733f610b4a598314	28	0.84	D_0__Bacteria;D_1__Poribacteria;Ambiguous_taxa;Ambiguous_taxa;Ambiguous_taxa;Ambiguous_taxa;Ambiguous_taxa
ede01e35b9bda5596afaf5de344be60a	27	1.00	D_0__Bacteria;D_1__Poribacteria;D_2__uncultured Clostridium sp.;D_3__uncultured Clostridium sp.;D_4__uncultured Clostridium sp.;D_5__uncultured Clostridium sp.;D_6__uncultured Clostridium sp.
aca60ca3e5eaa438d2727812181a5d49	26	0.75	D_0__Bacteria;D_1__Chloroflexi;D_2__Dehalococcoidia;D_3__SAR202 clade;D_4__uncultured Chloroflexi bacterium;D_5__uncultured Chloroflexi bacterium;D_6__uncultured Chloroflexi bacterium
780bb610683bccb8da96924032841de5	25	1.00	D_0__Bacteria;D_1__Proteobacteria;D_2__Gammaproteobacteria;D_3__Nitrosococcales;D_4__Nitrosococcaceae;D_5__AqS1
5218f08d0699090d480112f7a2e42b04	25	0.97	D_0__Bacteria;D_1__Chloroflexi;D_2__Anaerolineae;D_3__Caldilineales;D_4__Caldilineaceae;D_5__uncultured;D_6__uncultured Chloroflexi bacterium
e1428e39c1d30e45826bc6cadc71b8c9	23	1.00	D_0__Bacteria;D_1__Chloroflexi;D_2__Dehalococcoidia;D_3__SAR202 clade
7fb620f63b0ba7a2dcca8d3b6b3b7182	23	1.00	D_0__Bacteria;D_1__Proteobacteria;D_2__Gammaproteobacteria;D_3__KI89A clade
796a67287e1571ef2f76f82d8f2c0877	23	0.92	D_0__Bacteria;D_1__Proteobacteria;D_2__Deltaproteobacteria;D_3__Myxococcales;D_4__bacteriap25;D_5__uncultured bacterium;D_6__uncultured bacterium
eda17da4d4a7ceaa7b068a4bfb8cdf72	22	1.00	D_0__Bacteria;D_1__Poribacteria;Ambiguous_taxa;Ambiguous_taxa;Ambiguous_taxa;Ambiguous_taxa;Ambiguous_taxa
c823fd6495ad85438d95d71c8feccb52	22	0.97	D_0__Bacteria;D_1__Chloroflexi;D_2__Dehalococcoidia;D_3__SAR202 clade;Ambiguous_taxa;Ambiguous_taxa;Ambiguous_taxa
6709981fee5684c0347efc6b38687e24	21	1.00	D_0__Bacteria;D_1__Poribacteria
f4cdcca83dfec05d95f04363d3ffe209	20	0.99	D_0__Bacteria;D_1__Proteobacteria;D_2__Gammaproteobacteria;D_3__KI89A clade;D_4__uncultured bacterium;D_5__uncultured bacterium;D_6__uncultured bacterium
851e8012e91152230e8ac62f1a6d8884	20	1.00	D_0__Bacteria;D_1__Proteobacteria;D_2__Gammaproteobacteria;D_3__KI89A clade;D_4__uncultured bacterium;D_5__uncultured bacterium;D_6__uncultured bacterium

625cb936544fe26b79e2cbd375e19715	20	1.00	D_0__Bacteria;D_1__Poribacteria;D_2__Candidatus Poribacteria bacterium WGA-4C;D_3__Candidatus Poribacteria bacterium WGA-4C;D_4__Candidatus Poribacteria bacterium WGA-4C;D_5__Candidatus Poribacteria bacterium WGA-4C;D_6__Candidatus Poribacteria bacterium WGA-4C
471195b09a0fb63742bebcd7cdbe8873	20	1.00	D_0__Bacteria;D_1__Proteobacteria;D_2__Gammaproteobacteria;D_3__EPR3968-O8a-Bc78;Ambiguous_taxa;Ambiguous_taxa;Ambiguous_taxa
1bb1706de6355fa5b0c0e20d631a2edd	20	1.00	D_0__Bacteria;D_1__Chloroflexi;D_2__TK17
2e900bad2ee68745e7be07113398942a	19	1.00	D_0__Bacteria;D_1__Poribacteria;D_2__uncultured Clostridium sp.;D_3__uncultured Clostridium sp.;D_4__uncultured Clostridium sp.;D_5__uncultured Clostridium sp.;D_6__uncultured Clostridium sp.
0f4a02277d3363604c1f95c85fe1e07e	19	1.00	D_0__Bacteria;D_1__Chloroflexi;D_2__Dehalococcoidia;D_3__SAR202 clade
02dbb71ca3012d339c35e4ce491ccf4d	19	0.93	D_0__Bacteria;D_1__PAUC34f;D_2__uncultured Deferribacteres bacterium;D_3__uncultured Deferribacteres bacterium;D_4__uncultured Deferribacteres bacterium;D_5__uncultured Deferribacteres bacterium;D_6__uncultured Deferribacteres bacterium
c4e8bc19759046573b8b3987c4504841	18	0.82	D_0__Bacteria;D_1__Chloroflexi;D_2__Dehalococcoidia;D_3__SAR202 clade;D_4__uncultured deep-sea bacterium;D_5__uncultured deep-sea bacterium;D_6__uncultured deep-sea bacterium
7ff05623976bcb5dcb6084fb5e1684ac	18	0.99	D_0__Bacteria;D_1__Poribacteria;D_2__uncultured Planctomycetales bacterium;D_3__uncultured Planctomycetales bacterium;D_4__uncultured Planctomycetales bacterium;D_5__uncultured Planctomycetales bacterium;D_6__uncultured Planctomycetales bacterium
c18fd2bfef8e264fdbf032d29a8cc71c	17	1.00	D_0__Bacteria;D_1__Poribacteria;D_2__Candidatus Poribacteria bacterium WGA-4C;D_3__Candidatus Poribacteria bacterium WGA-4C;D_4__Candidatus Poribacteria bacterium WGA-4C;D_5__Candidatus Poribacteria bacterium WGA-4C;D_6__Candidatus Poribacteria bacterium WGA-4C
5482cfb4b230acd5e15f63d13a932d5a	17	1.00	D_0__Bacteria;D_1__Verrucomicrobia;D_2__Verrucomicrobiae;D_3__Opitutales;D_4__Puniceicoccaceae;D_5__Ceras icoccus;Ambiguous_taxa
1763acbf44cb9ce6a54b536de97fd1a6	17	1.00	D_0__Bacteria;D_1__Chloroflexi;D_2__Dehalococcoidia;D_3__SAR202 clade
1130ad4ef5c575f2ddb1fa47bf2ca89d	17	0.97	D_0__Bacteria;D_1__Actinobacteria;D_2__Acidimicrobiia;D_3__Actinomarinales;D_4__uncultured;D_5__uncultured actinobacterium;D_6__uncultured actinobacterium

ca3605d498491f3ba7acc309479682f6	16	0.82	D_0__Bacteria;D_1__Chloroflexi;D_2__Dehalococcoidia;D_3__SAR202 clade;Ambiguous_taxa;Ambiguous_taxa;Ambiguous_taxa
440372014bb52e796573e652206cd42b	16	0.95	D_0__Bacteria;D_1__PAUC34f;Ambiguous_taxa;Ambiguous_taxa;Ambiguous_taxa;Ambiguous_taxa;Ambiguous_taxa
d453f2afbbaac156b3df0f2ef722ec74	15	0.86	D_0__Bacteria;D_1__Poribacteria;D_2__uncultured Clostridium sp.;D_3__uncultured Clostridium sp.;D_4__uncultured Clostridium sp.;D_5__uncultured Clostridium sp.;D_6__uncultured Clostridium sp.
8af02bc807f04196cebfefd60b7b1ceb	15	0.80	D_0__Bacteria;D_1__Actinobacteria;D_2__Acidimicrobiia;D_3__Microtrichales;D_4__Microtrichaceae;D_5__Sva0996 marine group;D_6__actinobacterium MSI70
851f2657ffac970feead9638b27e4c33	15	0.93	D_0__Bacteria;D_1__Nitrospirae;D_2__Nitrospira;D_3__Nitrospirales;D_4__Nitrospiraceae;D_5__Nitrospira;D_6__uncultured Nitrospirae bacterium
ab0893b7de88347f92003023342fa769	14	0.92	D_0__Bacteria;D_1__Actinobacteria;D_2__Acidimicrobiia;D_3__Microtrichales;D_4__Microtrichaceae;D_5__Sva0996 marine group;D_6__uncultured actinobacterium
4dad126290476f6f575ac82cbab927a0	14	1.00	D_0__Bacteria;D_1__Chloroflexi;D_2__Dehalococcoidia;D_3__SAR202 clade;D_4__uncultured bacterium;D_5__uncultured bacterium;D_6__uncultured bacterium
0d319457b4c520ce1e2d4120f0704b17	14	0.95	D_0__Bacteria;D_1__PAUC34f;D_2__uncultured Deferribacteres bacterium;D_3__uncultured Deferribacteres bacterium;D_4__uncultured Deferribacteres bacterium;D_5__uncultured Deferribacteres bacterium;D_6__uncultured Deferribacteres bacterium
ef88bb07bf83ee8b62cc3bc917e57903	13	0.92	D_0__Bacteria;D_1__Chloroflexi;D_2__Dehalococcoidia;D_3__SAR202 clade;D_4__uncultured Chloroflexi bacterium;D_5__uncultured Chloroflexi bacterium;D_6__uncultured Chloroflexi bacterium
5859bb874fc4aa31541f65bbcb03e8a9	13	0.84	D_0__Bacteria;D_1__Poribacteria;D_2__Candidatus Poribacteria bacterium WGA-4C;D_3__Candidatus Poribacteria bacterium WGA-4C;D_4__Candidatus Poribacteria bacterium WGA-4C;D_5__Candidatus Poribacteria bacterium WGA-4C;D_6__Candidatus Poribacteria bacterium WGA-4C
3e190f2424edb0f7403f80bca6371717	13	1.00	D_0__Bacteria;D_1__Poribacteria;D_2__Candidatus Poribacteria bacterium WGA-4C;D_3__Candidatus Poribacteria bacterium WGA-4C;D_4__Candidatus Poribacteria bacterium WGA-4C;D_5__Candidatus Poribacteria bacterium WGA-4C;D_6__Candidatus Poribacteria bacterium WGA-4C
bb86bcdc3ea0d1dfdc60c4fe529f3475	12	0.76	D_0__Bacteria;D_1__Chloroflexi;D_2__Dehalococcoidia;D_3__SAR202 clade;D_4__uncultured bacterium;D_5__uncultured bacterium;D_6__uncultured bacterium

3e395f6548d2cac8ad2900dcb3f98f57	12	0.99	D_0__Bacteria;D_1__Proteobacteria;D_2__Gammaproteobacteria;D_3__KI89A clade;D_4__uncultured bacterium;D_5__uncultured bacterium;D_6__uncultured bacterium
fd48905167b54399e95c363d9b17bf80	11	0.99	D_0__Archaea;D_1__Thaumarchaeota;D_2__Nitrososphaeria;D_3__Nitrosopumilales;D_4__Nitrosopumilaceae;D_5__Candidatus Nitrosopumilus
fba58b3be6d1e44e5f4da2ba24c0d9bc	11	0.99	D_0__Bacteria;D_1__Poribacteria;Ambiguous_taxa;Ambiguous_taxa;Ambiguous_taxa;Ambiguous_taxa;Ambiguous_taxa
d644029ea9c1776aef75cb247dbd33e6	11	1.00	D_0__Bacteria;D_1__Proteobacteria;D_2__Gammaproteobacteria;D_3__Steroidobacterales;D_4__Woeseiaceae;D_5__JTB255 marine benthic group;Ambiguous_taxa
793045ce6707d8593a7aa016b3d425cb	11	1.00	D_0__Bacteria;D_1__Chloroflexi;D_2__Anaerolineae;D_3__SBR1031;D_4__A4b
791953a576079b24b6a5e096f41fc531	11	0.79	D_0__Bacteria;D_1__Nitrospinae;D_2__P9X2b3D02;D_3__uncultured bacterium;D_4__uncultured bacterium;D_5__uncultured bacterium;D_6__uncultured bacterium
78d65f6867453768609355 79de2bfad9	11	1.00	D_0__Bacteria;D_1__Proteobacteria;D_2__Gammaproteobacteria;D_3__KI89A clade;D_4__uncultured bacterium;D_5__uncultured bacterium;D_6__uncultured bacterium
1d1ed03410df4629cd95235f6e445bad	11	1.00	D_0__Bacteria;D_1__Acidobacteria;D_2__Acidobacteriia;D_3__Solibacterales;D_4__Solibacteraceae (Subgroup 3);D_5__PAUC26f;Ambiguous_taxa
1b292f6ddc75e6694b00ab437a68137f	11	1.00	D_0__Bacteria;D_1__Chloroflexi;D_2__Dehalococcoidia;D_3__SAR202 clade
f66c2a7cae98e194102fa235871c78f4	10	1.00	D_0__Bacteria;D_1__Gemmatimonadetes;D_2__BD2-11 terrestrial group
be3c73452201b90ef6d4c0ede59f7827	10	0.88	D_0__Bacteria;D_1__Actinobacteria;D_2__Acidimicrobiia;D_3__Microtrichales;D_4__Microtrichaceae;D_5__Sva0996 marine group;D_6__uncultured actinobacterium
9865f63ba4eda05ffd69de6e44c98bdd	10	1.00	D_0__Bacteria;D_1__Proteobacteria;D_2__Alphaproteobacteria;D_3__Puniceispirillales;D_4__EF100-94H03;Ambiguous_taxa;Ambiguous_taxa
4232fab6250e5143b45c838e81435049	10	1.00	D_0__Bacteria;D_1__Proteobacteria;D_2__Gammaproteobacteria;D_3__Nitrosococcales;D_4__Nitrosococcaceae;D_5__FS142-36B-02

3c1fa1eb0fc81573490a4c0d7516ca59	10	0.98	D_0__Bacteria;D_1__Chloroflexi;D_2__Dehalococcoidia;D_3__SAR202 clade;D_4__uncultured gamma proteobacterium;D_5__uncultured gamma proteobacterium;D_6__uncultured gamma proteobacterium
3b2ed357b7db45320c77760b8294db42	10	0.98	D_0__Bacteria;D_1__Acidobacteria;D_2__Subgroup 9;D_3__uncultured bacterium;D_4__uncultured bacterium;D_5__uncultured bacterium;D_6__uncultured bacterium
2e5202c9febc5b65148486edb223eead	10	0.94	D_0__Bacteria;D_1__Bacteroidetes;D_2__Rhodothermia;D_3__Rhodothermales;D_4__Rhodothermaceae;D_5__uncultured;D_6__uncultured bacterium
8c6e4ae5ad4ac83a74bf3335e4fd5610	9	0.90	D_0__Bacteria;D_1__Gemmatimonadetes;D_2__BD2-11 terrestrial group;D_3__uncultured bacterium;D_4__uncultured bacterium;D_5__uncultured bacterium;D_6__uncultured bacterium
551d8b58dee63e48c7a38f7402073acd	9	0.90	D_0__Bacteria;D_1__Proteobacteria;D_2__Gammaproteobacteria;D_3__UBA10353 marine group;Ambiguous_taxa;Ambiguous_taxa;Ambiguous_taxa
2091bc42610d75305f029cd30a949233	9	0.97	D_0__Bacteria;D_1__Chloroflexi;D_2__Anaerolineae;D_3__Caldilineales;D_4__Caldilineaceae;D_5__uncultured;D_6__uncultured Chloroflexus sp.
1a8b286372fa27b78b54f7580206ee34	9	0.75	D_0__Bacteria;D_1__Proteobacteria;D_2__Gammaproteobacteria;D_3__OM182 clade;D_4__uncultured gamma proteobacterium;D_5__uncultured gamma proteobacterium;D_6__uncultured gamma proteobacterium
c5ce5fedffa09328ae705cef668d6285	8	1.00	D_0__Bacteria;D_1__Proteobacteria;D_2__Gammaproteobacteria;D_3__pItb-vmat-80;D_4__uncultured bacterium;D_5__uncultured bacterium;D_6__uncultured bacterium
b83b6daa1c18fa9c524183e8eb81b897	8	1.00	D_0__Bacteria;D_1__Deinococcus-Thermus;D_2__Deinococci;D_3__Deinococcales;D_4__Trueperaceae;D_5__Truepera;D_6__uncultured Truepera sp.
a4e1ec8c5517cf11b1c058a07deaeba4	8	1.00	D_0__Bacteria;D_1__Proteobacteria;D_2__Gammaproteobacteria;D_3__UBA10353 marine group
854f4e49244b64e70125efe1ffe68a8f	8	0.98	D_0__Bacteria;D_1__Actinobacteria;D_2__Acidimicrobiia;D_3__Microtrichales;D_4__Microtrichaceae;D_5__Sva0996 marine group;D_6__uncultured bacterium
759ca3ffad82623378459ab36d3a3b23	8	1.00	D_0__Bacteria;D_1__Actinobacteria;D_2__Acidimicrobiia;D_3__Actinomarinales;D_4__uncultured;Ambiguous_taxa;Ambiguous_taxa
3fb6df12e25efe2b59fbaced65832f4d	8	0.97	D_0__Bacteria;D_1__Acidobacteria;D_2__Subgroup 21;Ambiguous_taxa;Ambiguous_taxa;Ambiguous_taxa;Ambiguous_taxa

ec0f9d9336a4ae07316c33ba8bc92476	7	1.00	D_0__Bacteria;D_1__Poribacteria;D_2__uncultured Clostridium sp.;D_3__uncultured Clostridium sp.;D_4__uncultured Clostridium sp.;D_5__uncultured Clostridium sp.;D_6__uncultured Clostridium sp.
dfa4284dc3a67e0de2a1974bdb3e749c	7	0.95	D_0__Bacteria;D_1__Proteobacteria;D_2__Alphaproteobacteria;D_3__Rhodobacterales;D_4__Rhodobacteraceae;D_5__uncultured
a8319db4cc193409bb9f79a6c8259839	7	0.97	D_0__Bacteria;D_1__Proteobacteria;D_2__Alphaproteobacteria;D_3__uncultured
81c21fe55c98672337ef2fefe95ff5ee	7	0.84	D_0__Bacteria;D_1__Proteobacteria;D_2__Alphaproteobacteria;D_3__Rhodospirillales;D_4__Magnetospiraceae;D_5__uncultured;D_6__uncultured bacterium
4085a9870507c42aacda48ffbc09c1d2	7	0.91	D_0__Bacteria;D_1__Chloroflexi;D_2__Dehalococcoidia;D_3__SAR202 clade;D_4__uncultured bacterium;D_5__uncultured bacterium;D_6__uncultured bacterium
155d04463122b0cbe3d8ccff635d6b84	7	0.90	D_0__Bacteria;D_1__Proteobacteria;D_2__Deltaproteobacteria;D_3__Myxococcales;D_4__bacteriap25
09b6a5248ed0b4f66a344f633ff3f98b	7	0.90	D_0__Bacteria;D_1__Chloroflexi;D_2__Dehalococcoidia;D_3__SAR202 clade;D_4__uncultured bacterium;D_5__uncultured bacterium;D_6__uncultured bacterium
03241d598a0425ae03563b29eb4cf618	7	0.89	D_0__Bacteria;D_1__Proteobacteria;D_2__Alphaproteobacteria;D_3__Rhodobacterales;D_4__Rhodobacteraceae;D_5__uncultured;D_6__uncultured Rhodobacter sp.
f2dd98d872a988571bf773521f13dcf9	6	1.00	D_0__Bacteria;D_1__Proteobacteria;D_2__Alphaproteobacteria;D_3__Rhodobacterales;D_4__Rhodobacteraceae;D_5__Albidovulum
da12db4ab27cd8061c031ebcca1f0003	6	1.00	D_0__Bacteria;D_1__Proteobacteria;D_2__Gammaproteobacteria;D_3__KI89A clade;D_4__uncultured bacterium;D_5__uncultured bacterium;D_6__uncultured bacterium
a1b218e47c04189a5584e821f0679646	6	1.00	D_0__Bacteria;D_1__Chloroflexi;D_2__Anaerolineae;D_3__SBR1031;D_4__A4b
a000f9f96113681ec1e889f18243ada8	6	0.91	D_0__Bacteria;D_1__Proteobacteria;D_2__Gammaproteobacteria;D_3__KI89A clade;D_4__uncultured bacterium;D_5__uncultured bacterium;D_6__uncultured bacterium
53c2a43138e5ea2380208e37c3b27937	6	0.94	D_0__Bacteria;D_1__Chloroflexi;D_2__Dehalococcoidia;D_3__SAR202 clade;Ambiguous_taxa;Ambiguous_taxa;Ambiguous_taxa
239c8716592fa42d0271ee7c52004cf9	6	0.99	D_0__Bacteria

1451aed214e4abb063e1632186a85017	6	0.78	D_0__Bacteria;D_1__PAUC34f;Ambiguous_taxa;Ambiguous_taxa;Ambiguous_taxa;Ambiguous_taxa;Ambiguous_taxa
0a7637a2daafa5cf5d1d38d4b7ccbd48	6	0.96	D_0__Bacteria;D_1__Poribacteria;D_2__uncultured Clostridium sp.;D_3__uncultured Clostridium sp.;D_4__uncultured Clostridium sp.;D_5__uncultured Clostridium sp.;D_6__uncultured Clostridium sp.
d6936c4d69f6cd1eb8ade88b309e7a4e	5	0.80	D_0__Bacteria;D_1__Chloroflexi;D_2__Dehalococcoidia;D_3__SAR202 clade;D_4__uncultured deep-sea bacterium;D_5__uncultured deep-sea bacterium;D_6__uncultured deep-sea bacterium
cb3903e58b3e61c940045cf599b3812c	5	0.87	D_0__Bacteria;D_1__Gemmatimonadetes;D_2__BD2-11 terrestrial group;D_3__uncultured bacterium;D_4__uncultured bacterium;D_5__uncultured bacterium;D_6__uncultured bacterium
734f25088d2f464de431ff355629ba84	5	1.00	D_0__Bacteria;D_1__Bacteroidetes;D_2__Rhodothermia;D_3__Rhodothermales;D_4__Rhodothermaceae;D_5__uncultured
4d41dbba450e3c89cd3267c359bbe65b	5	0.98	D_0__Bacteria;D_1__Proteobacteria;D_2__Deltaproteobacteria;D_3__Bdellovibrionales;D_4__Bdellovibrionaceae;D_5__Bdellovibrio;D_6__uncultured delta proteobacterium
bc84ec2b2c9b0f949d199dfee490db20	4	1.00	D_0__Bacteria;D_1__Proteobacteria;D_2__Gammaproteobacteria;D_3__KI89A clade
b318a10a8189b581afba620d3dafa2a1	4	0.99	D_0__Bacteria;D_1__Chloroflexi;D_2__Dehalococcoidia;D_3__SAR202 clade;D_4__uncultured Chloroflexi bacterium;D_5__uncultured Chloroflexi bacterium;D_6__uncultured Chloroflexi bacterium
6e3f70f8124a98fa0558ea9b4deaa9c3	4	0.98	D_0__Bacteria
6b2d5b73cd329f963c2600f86fc94c0b	4	1.00	D_0__Bacteria;D_1__Chloroflexi;D_2__TK17;D_3__uncultured bacterium;D_4__uncultured bacterium;D_5__uncultured bacterium;D_6__uncultured bacterium
6b270ec1a451d8edb4dc1a10f4fe32c4	4	1.00	D_0__Bacteria;D_1__Acidobacteria;D_2__Subgroup 6
343128e8b51345ce61bcaabb1a70f898	4	1.00	D_0__Bacteria;D_1__Proteobacteria;D_2__Alphaproteobacteria;D_3__Puniceispirillales;Ambiguous_taxa;Ambiguous_taxa;Ambiguous_taxa
1e95cf44d26ab6edc190a928a2419c85	4	0.84	D_0__Bacteria;D_1__Chloroflexi;D_2__TK17;Ambiguous_taxa;Ambiguous_taxa;Ambiguous_taxa;Ambiguous_taxa
ff4ed4aa2c20df79e589bdf3716dd69c	3	0.89	D_0__Bacteria;D_1__Bacteroidetes;D_2__Bacteroidia;D_3__Flavobacteriales;D_4__Flavobacteriaceae;D_5__NS5 marine group;Ambiguous_taxa

f124bfe2ad946e478a9b2a1450ed9aaf	3	1.00	D_0__Bacteria;D_1__Chloroflexi;D_2__Dehalococcoidia;D_3__SAR202 clade
cc0c237914f8fce030dbf9652e09dab0	3	1.00	D_0__Bacteria;D_1__Gemmatimonadetes;D_2__BD2-11 terrestrial group
f445f846d3229e65ea63ae071543cdec	2	0.94	D_0__Bacteria;D_1__Patescibacteria;D_2__Parcubacteria;D_3__Candidatus Kaiserbacteria;D_4__uncultured Parcubacteria group bacterium;D_5__uncultured Parcubacteria group bacterium;D_6__uncultured Parcubacteria group bacterium
d4500fc535478f4c4893fe42717b41a1	2	1.00	D_0__Bacteria;D_1__PAUC34f;Ambiguous_taxa;Ambiguous_taxa;Ambiguous_taxa;Ambiguous_taxa;Ambiguous_taxa
c9c2e3ffff8a341c0f74a81eb3515478	2	1.00	D_0__Bacteria;D_1__Gemmatimonadetes;D_2__BD2-11 terrestrial group
a765864a968178ae3bac8e359a2c568d	2	1.00	D_0__Bacteria;D_1__Acidobacteria;D_2__Subgroup 6
68cfc217ba7ca1e1c735d8aadaa3c123	2	1.00	D_0__Bacteria;D_1__Chloroflexi;D_2__TK30
48e094f8fcbed116d7e14e3543b09896	2	1.00	D_0__Bacteria;D_1__Verrucomicrobia;D_2__Verrucomicrobiae;D_3__Verrucomicrobiales;D_4__DEV007
27e4fedc76589e582be3a460aa71fa31	2	0.99	D_0__Bacteria;D_1__Patescibacteria;D_2__Saccharimonadia;D_3__Saccharimonadales;D_4__uncultured Candidatus Saccharibacteria bacterium;D_5__uncultured Candidatus Saccharibacteria bacterium;D_6__uncultured Candidatus Saccharibacteria bacterium
1d1377ab647c7cedf07b27e38f9849d7	2	0.83	D_0__Bacteria;D_1__Acidobacteria;D_2__Thermoanaerobaculia;D_3__Thermoanaerobaculales;D_4__Thermoanaerob aculaceae;D_5__Subgroup 10;D_6__uncultured Acidobacteria bacterium
27d44d64f162d8c2fa48cd9ff11022c1	1	0.90	D_0__Bacteria;D_1__Gemmatimonadetes;D_2__BD2-11 terrestrial group;D_3__uncultured bacterium;D_4__uncultured bacterium;D_5__uncultured bacterium;D_6__uncultured bacterium
02c1a4b0ce2eff9808ebe10fdfb5f218	1	0.97	D_0__Bacteria;D_1__Chloroflexi;D_2__Dehalococcoidia;D_3__SAR202 clade

Table S12. Absolute abundance of each phylum from the eight sponges under analysis. Sample IDs: O.per= *Oceanapia* cf. *perforata*, S.spi= *Sarcotragus spinosulus*, E.dis= *Erylus discophorus*, A.oro= *Agelas oroides*, T.aur= *Tethya aurantium*, A.dam= *Axinella damicornis*, A.acu= *Acanthella acuta* and G.cyd= *Geodia cydonium*. The *** indicates the phylum belonging to Archea.

**Phylum**	**O.per**	**S.spi**	**E.dis**	**A.oro**	**T.aur**	**A.dam**	**A.acu**	**G.cyd**
Proteobacteria	5835	3691	2324	4768	6427	15286	21739	800
Chloroflexi	5079	4915	1934	5303	3	7	330	1300
Poribacteria	2221	664	1006	473	0	0	0	817
Verrucomicrobia	1667	2	59	0	2	17	214	19
Actinobacteria	1217	1368	2732	261	1115	31	1122	72
Acidobacteria	1152	785	1535	824	0	0	5	149
Gemmatimonadetes	1026	196	1805	112	0	3	34	30
Nitrospirae	958	0	678	168	34	1880	1428	145
PAUC34f	400	70	496	98	0	0	0	57
Dadabacteria	360	646	211	366	349	3	24	76
Thaumarchaeota***	251	0	420	145	12	876	3576	11
Nitrospinae	153	80	41	0	0	0	0	11
Bacteroidetes	81	676	199	21	31	107	1187	18
AncK6	65	6	33	0	0	0	0	0
Firmicutes	12	0	21	0	0	36	76	0
Entotheonellaeota	4	21	9	22	0	0	0	0
Margulisbacteria	3	0	0	0	0	0	0	0

Spirochaetes	0	52	0	92	40	148	133	5
Planctomycetes	0	0	0	5	17	38	291	0
Patescibacteria	0	0	0	21	0	0	53	4
Marinimicrobia (SAR406 clade)	0	0	0	0	0	6	65	0
Lentisphaerae	0	0	2	15	0	0	4	0
Fusobacteria	0	0	0	0	0	0	5	0
Epsilonbacteraeota	0	0	0	0	0	0	26	0
Deinococcus-Thermus	0	0	0	0	0	0	0	8
Cyanobacteria	0	6	31	0	12	40	495	0
Chlamydiae	0	0	0	0	0	0	9	0

Table S13. Targeted region, forward and reverse primers, sequences (5’→ 3’) and reference of primers pairs used for molecular characterization.

Targeted region	Forward	Sequence (5'→3')	Reverse	Sequence (5'→3')	Reference
**18S**	A	AACCTGGTTGATC CTGCCAGT	B	TGATCCTTCTGCAGGTT CACCTAC	Medlin, 1988; Schmitt et al., 2005
**18S**	18S-AF	CTGGTTGATCCTGCCAG	18S-BR	CTGCAGGTTCACCTAC	Dohrmann et al., 2008; Collins, 2002
**18S**	18S1	AACCTGGTTGATCCTGCCA	18S2	TGCAGGTTCACCTACAGAA	Manuel et al., 2003
**ITS**	RA2	GTCCCTGCCCTTTGT ACACA	ITS2.2	CCTGGTTAGTTTCTTTTCCTCC GC	Wörheide et al., 2002a, b; Schmitt et al., 2005
**28S**	NL4F	GACCCGAAAGATGGTGAACTA	NL4R	ACCTTGGAGACCTGATGCG	Dohrmann et al., 2008; Collins, 2002
**28S**	C2	GAAAAGAACTTTGRARAGAGAGT	D2	TCCGTGTTTCAAGACGGG	Chombard et al., 1998
**COI**	dgLCO1490	GGTCAACAAATCATAAAGAYATYGG	dgHCO2198	TAAACTTCAGGGTGACCAAARAAYCA	Meyer et al., 2005

Table S14. Denoising process.

Sample IDs	Input	Filtered	Percentage of input passed filter	Denoised	Merged	Percentage of input merged	Non-chimeric	Percentage of input non-chimeric
**O.per**	38173	37663	98,66	34648	28729	75,26	21490	56,3
**S.spi**	29372	29323	99,83	26738	21427	72,95	13178	44,87
**E.dis**	27946	27815	99,53	25003	19562	70	13546	48,47
**A.oro**	28251	28191	99,79	25471	19901	70,44	14180	50,19
**T.aur**	25652	25612	99,84	24517	22673	88,39	18023	70,26
**A.dam**	32461	31317	96,48	29971	28653	88,27	19939	61,42
**A.acu**	53265	50325	94,48	47163	42008	78,87	32164	60,38
**G.cyd**	6927	6865	99,1	5826	4431	63,97	3535	51,03

Figure S1. Sample O.per. The sequence from 18SAF/18SBR primer pairs aligned to *O. isodictyiformis* (A) and *Oceanapia* sp. (B), the sequence from A/B primer pairs aligned to *O. isodictyiformis* (C) and *Oceanapia* sp. (D), the sequence of NL4F/NL4R primer pairs aligned to *Oceanapia* sp. (E).

A

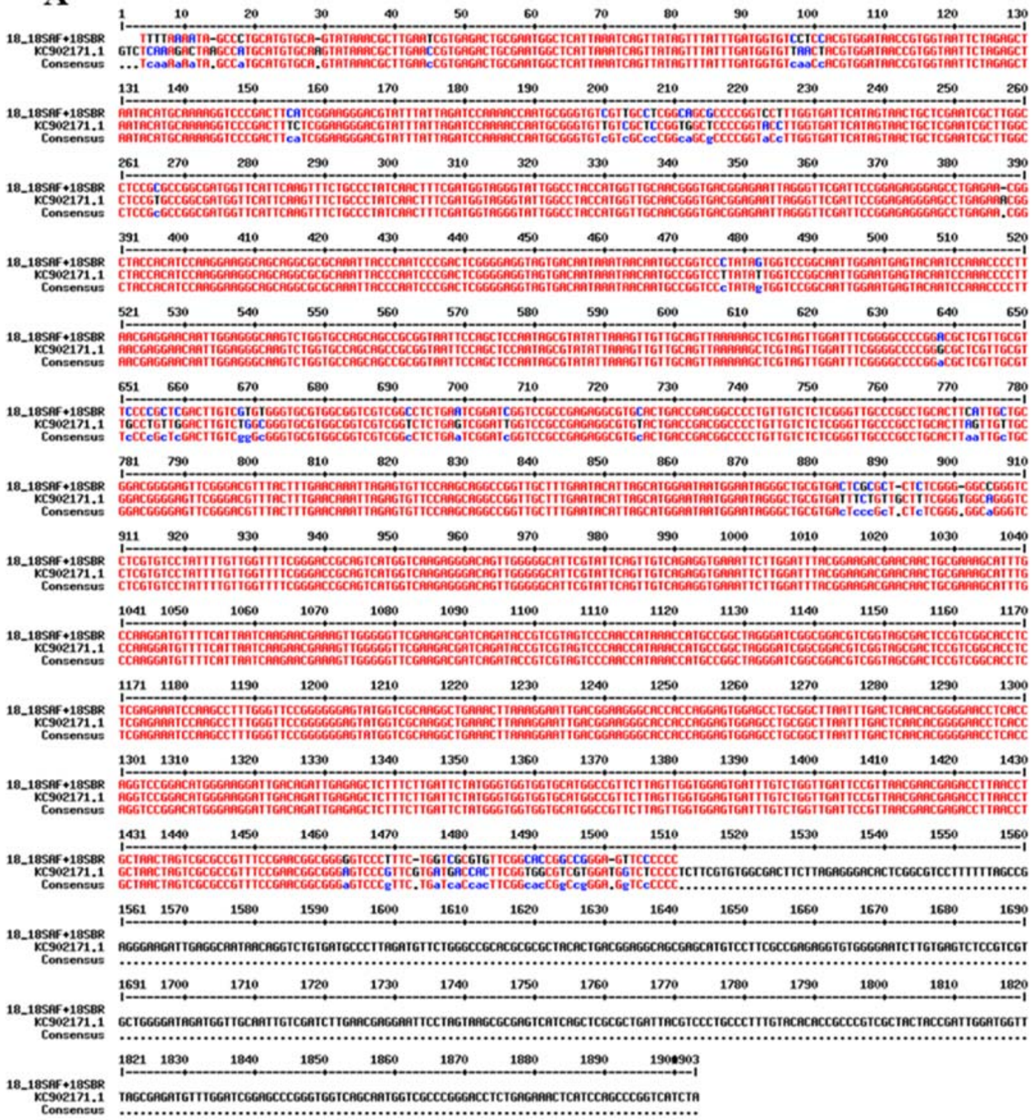

B

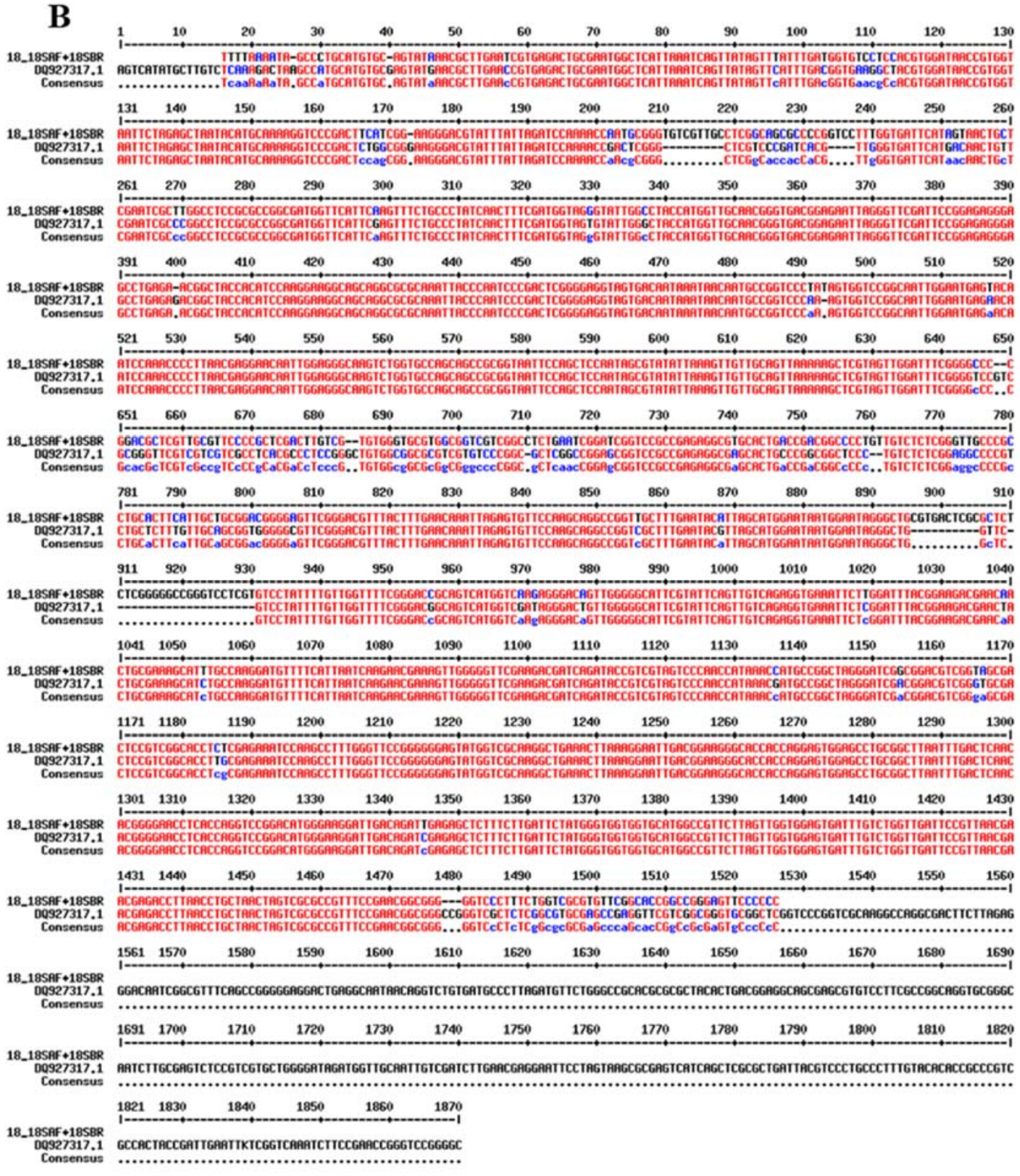

C

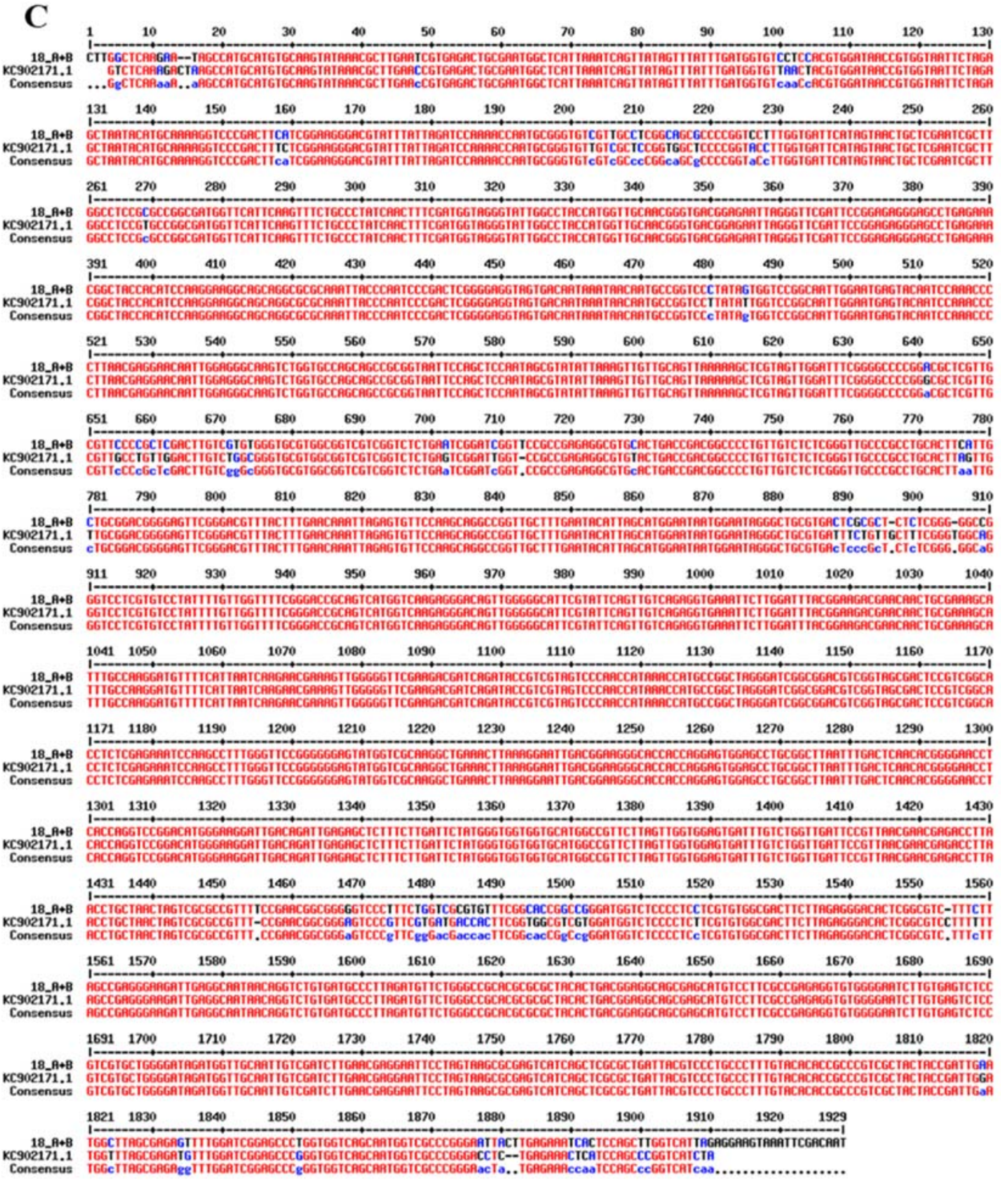

D

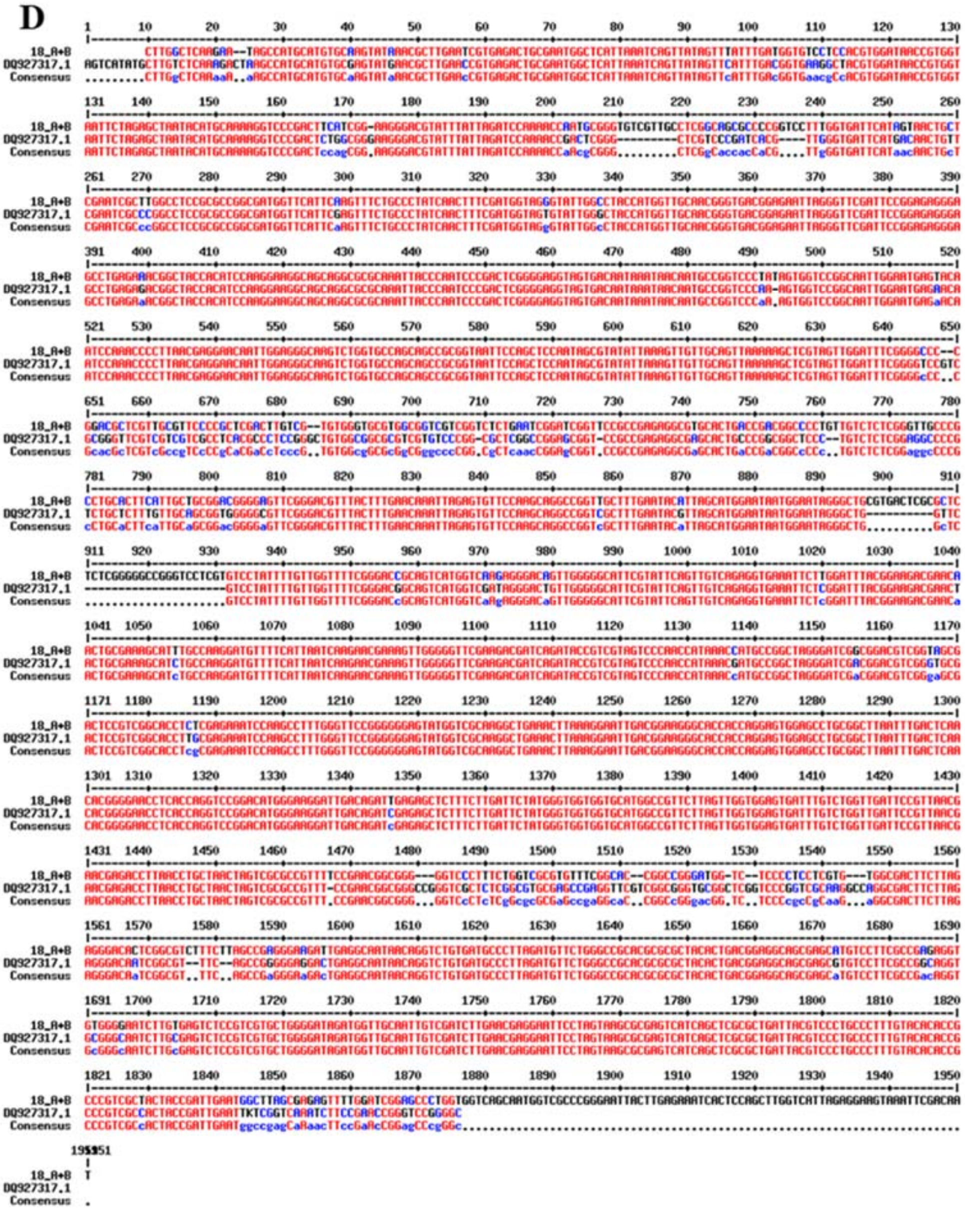

E

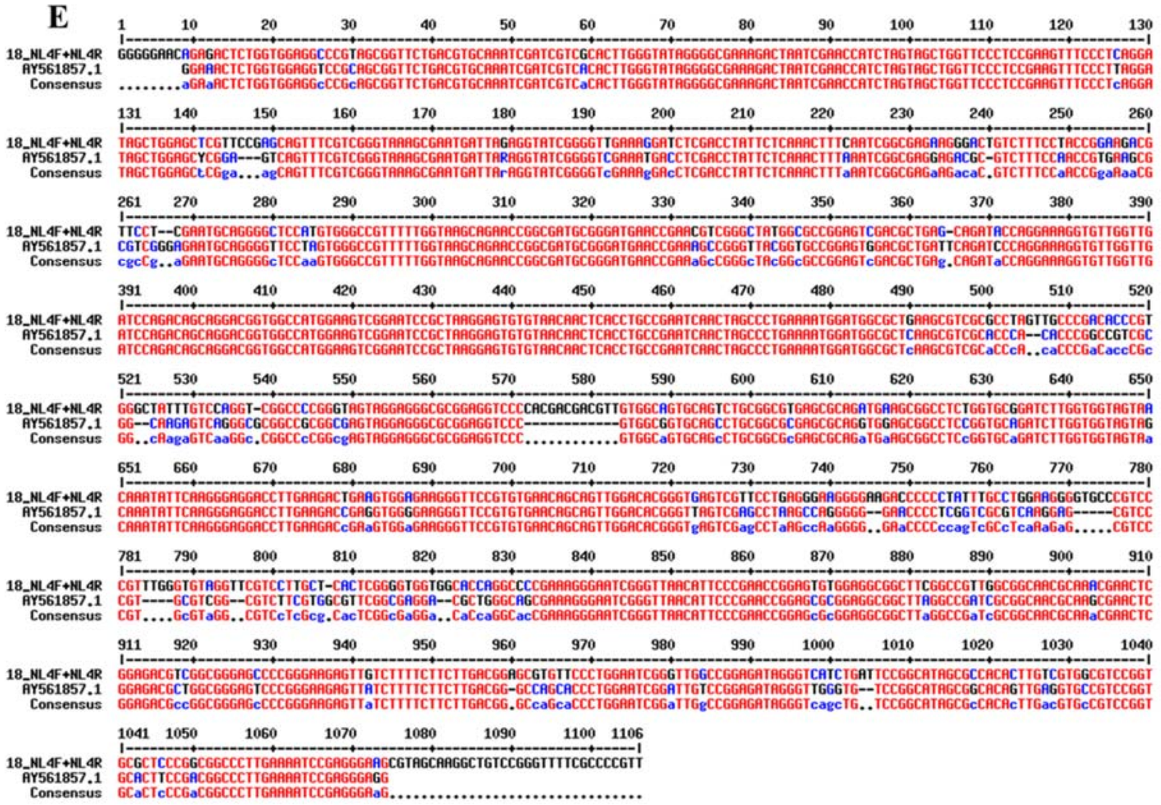

Figure S2. Sample S.spi. Alignment of fragments (355 bp on the total fragment of 846 bp) obtained with RA2/ITS2.2 primer pairs aligned to the first hit (*S. spinosulus*) corresponding to the species identified from morphological analysis.

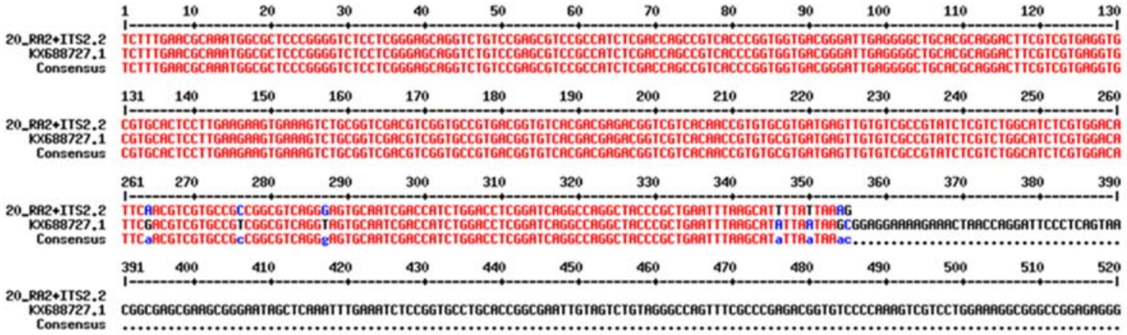

Figure S3. Sample E.dis. Alignments of PCR sequences obtained with dgLCO1490/dgHCO2198 primer pairs aligned to the first hit (*E. discophorus*) corresponding to the species as identified from morphological analysis.

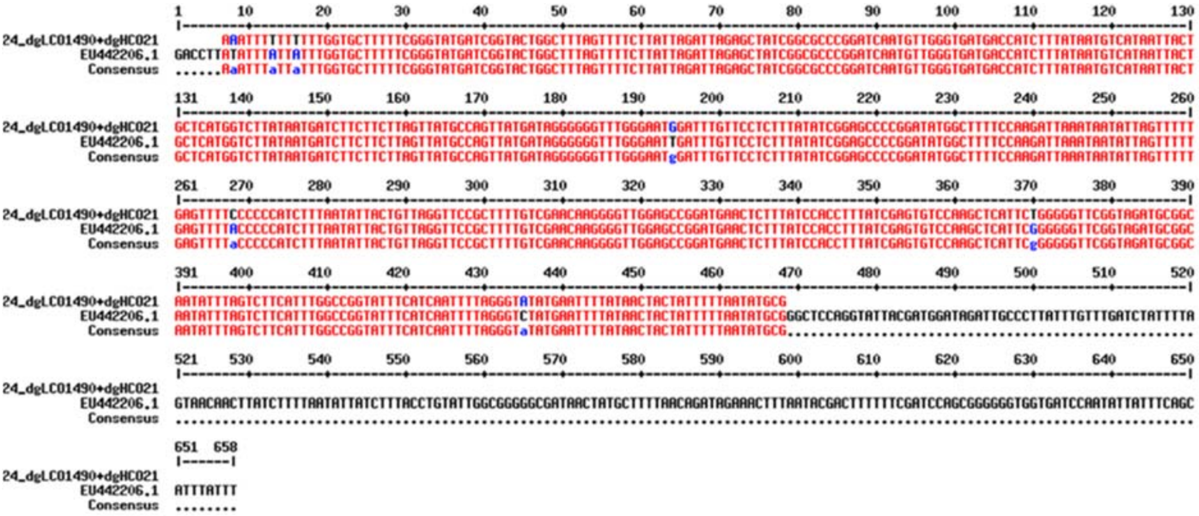

Figure S4. Sample A.oro. Alignments of PCR sequences obtained with RA2/ITS2.2 primer pairs aligned to (A) the first hit (*Agelas* sp.) and (B) *A. oroides* (corresponding to the species as identified from morphological analysis; with C2/D2 primer pairs aligned to (C) the first hit (*A. oroides*); with A/B primer pairs aligned to (D) the first hit; with 18SAF/18SBR primer pairs aligned to (E) the first hit (*A. oroides*); with NL4F/NL4R primer pairs aligned to (F) the first hit (*A. oroides*). Forward and reverse primers were highligted by the green and blue boxes, respectively.

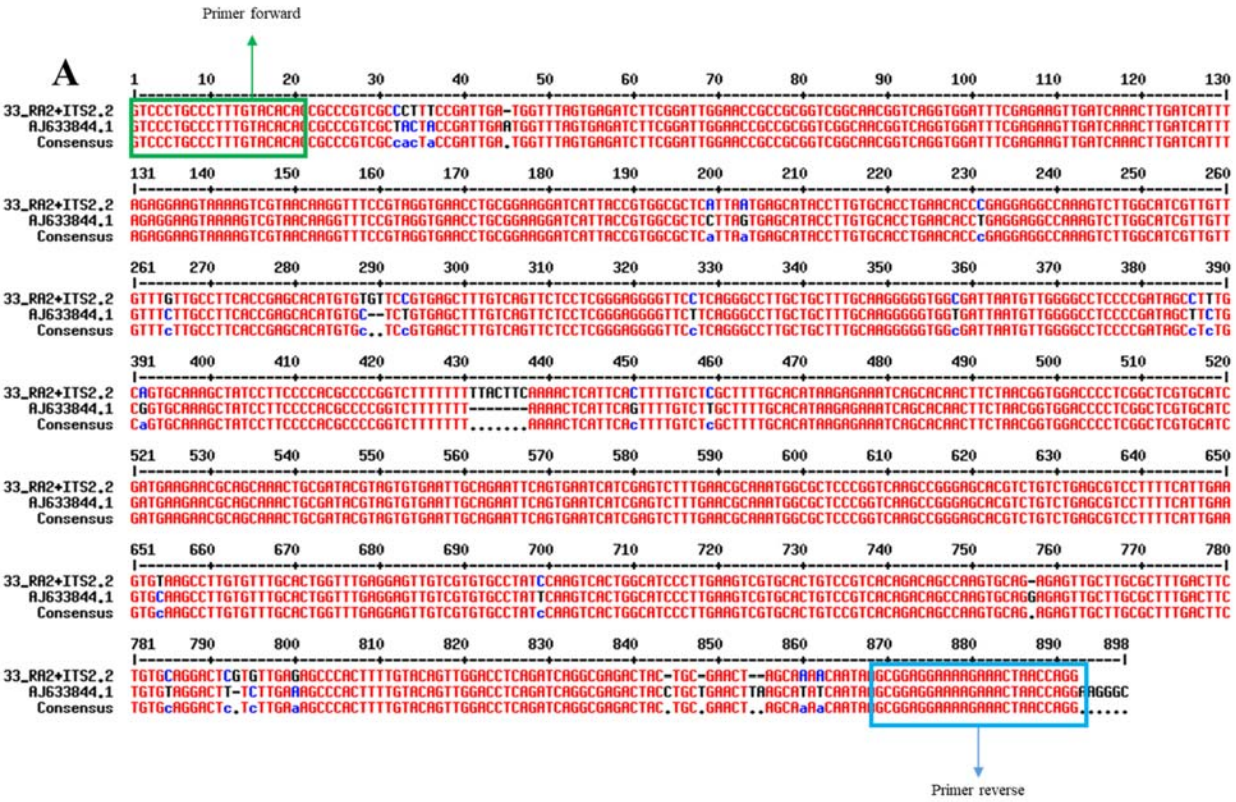

B

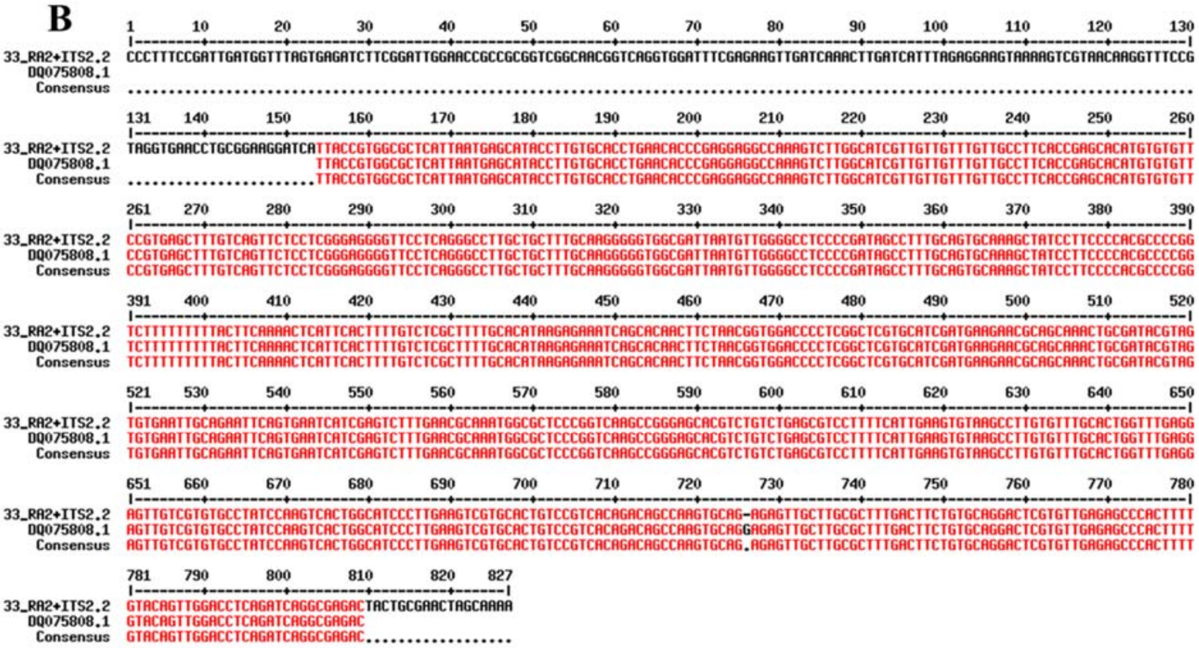

33_RA2+ITS2.2
DQ075808.1
Consensus

C

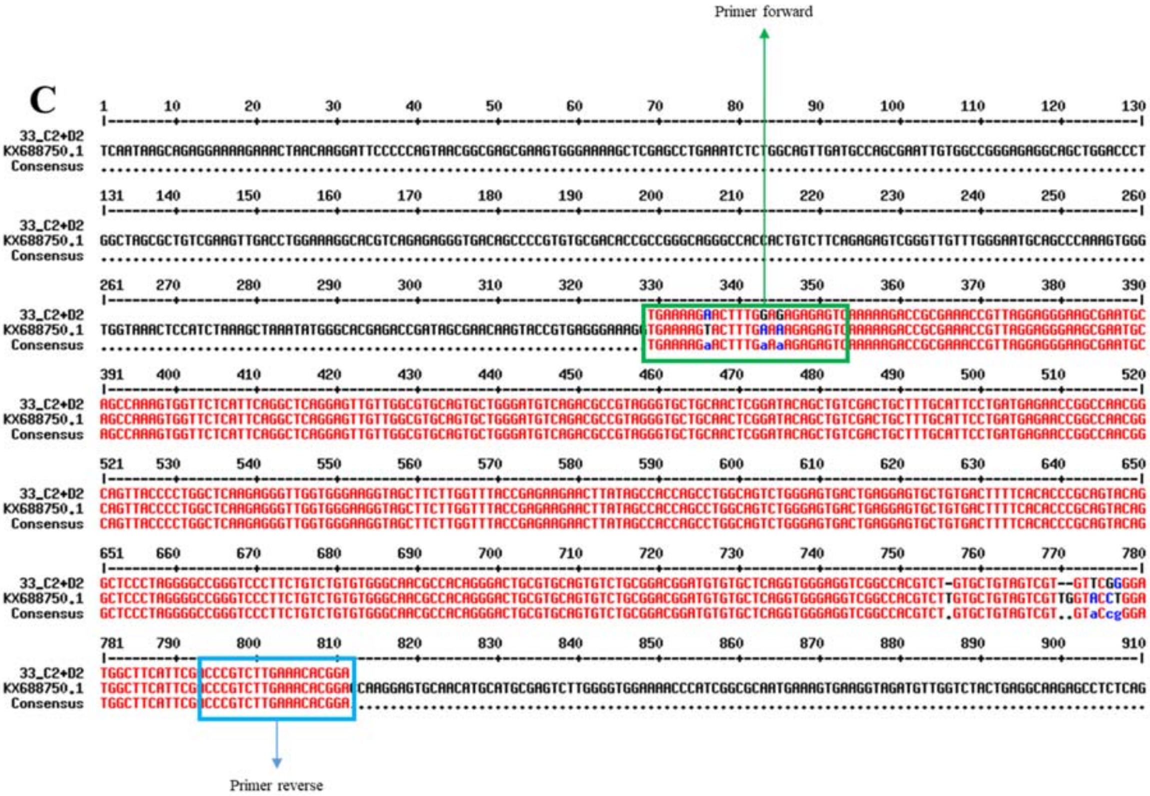

Primer forward
33_C2+D2
KX688750.1
Consensus
Primer reverse

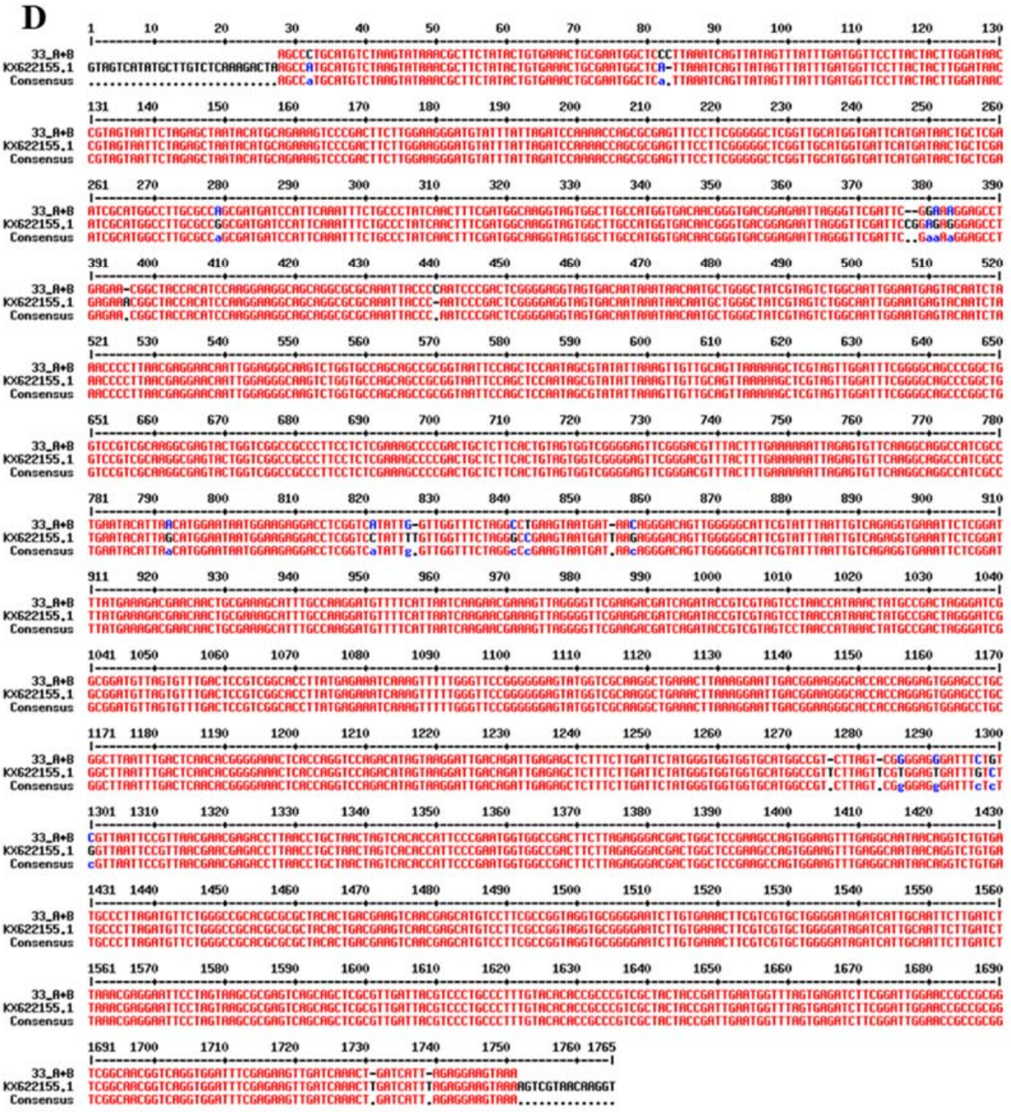
D
33_A+B
KX622155.1
Consensus

E

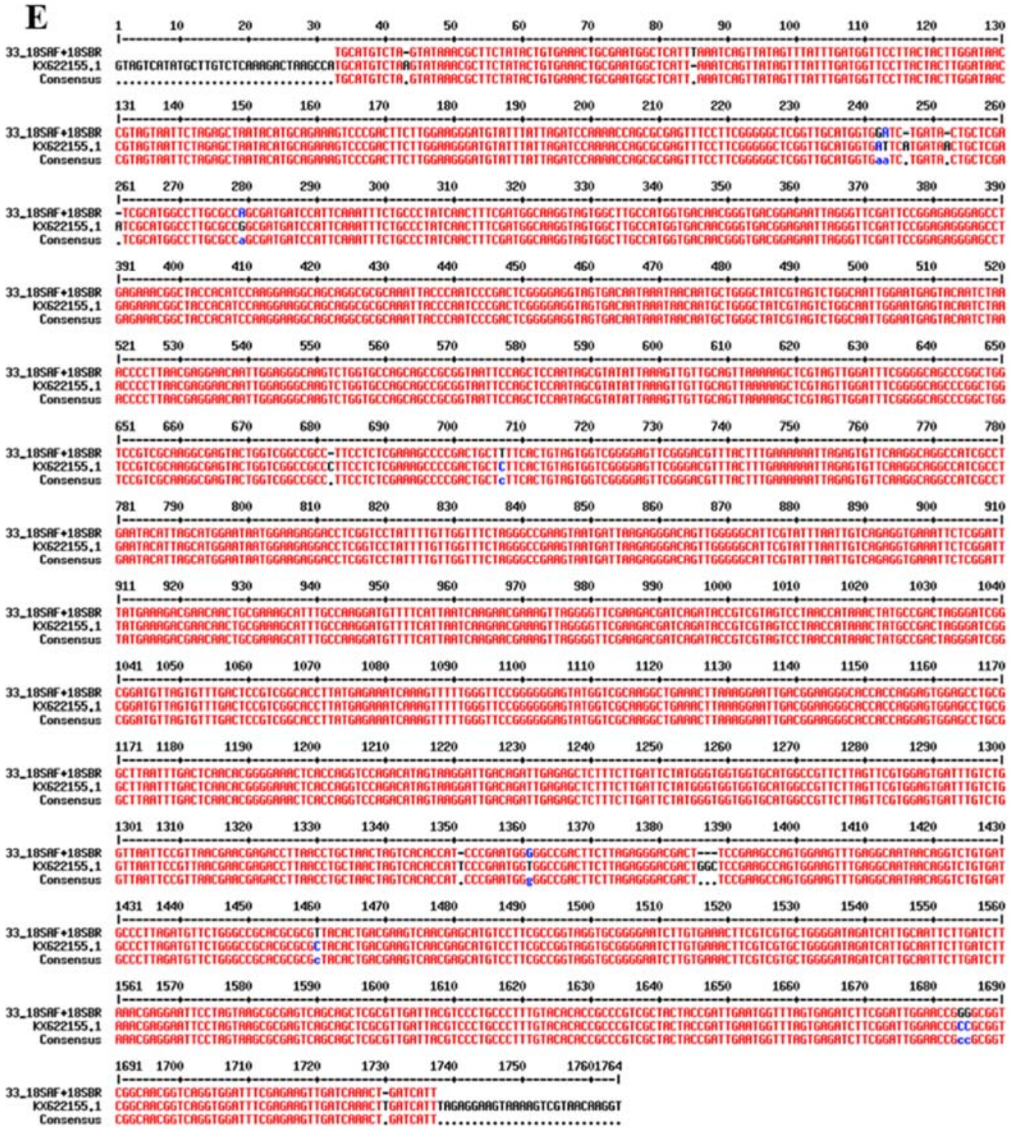

F

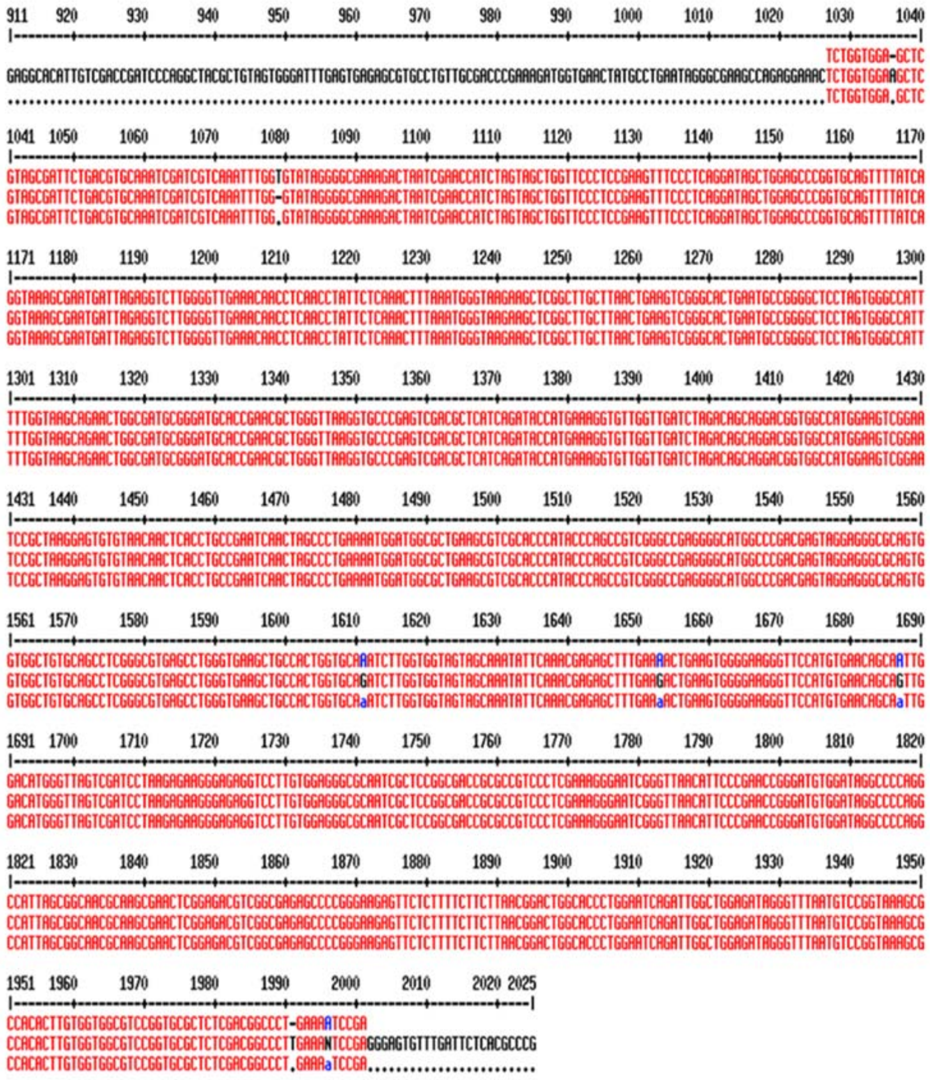

Figure S5. Sample T.aur. Alignments of PCR fragments obtained with 18S1/18S2 primer pairs aligned to (A) the first hit (*Tethya sp.*) and (B) *T. aurantium* (corresponding to the species as identified from morphological analysis); with A/B primer pairs aligned to (C) the first hit *(Tethya sp.*) and (D) *T. aurantium*; (E) with dgLCO1490/dgHCO2198 primer pairs aligned to the first hit (*T. aurantium*).

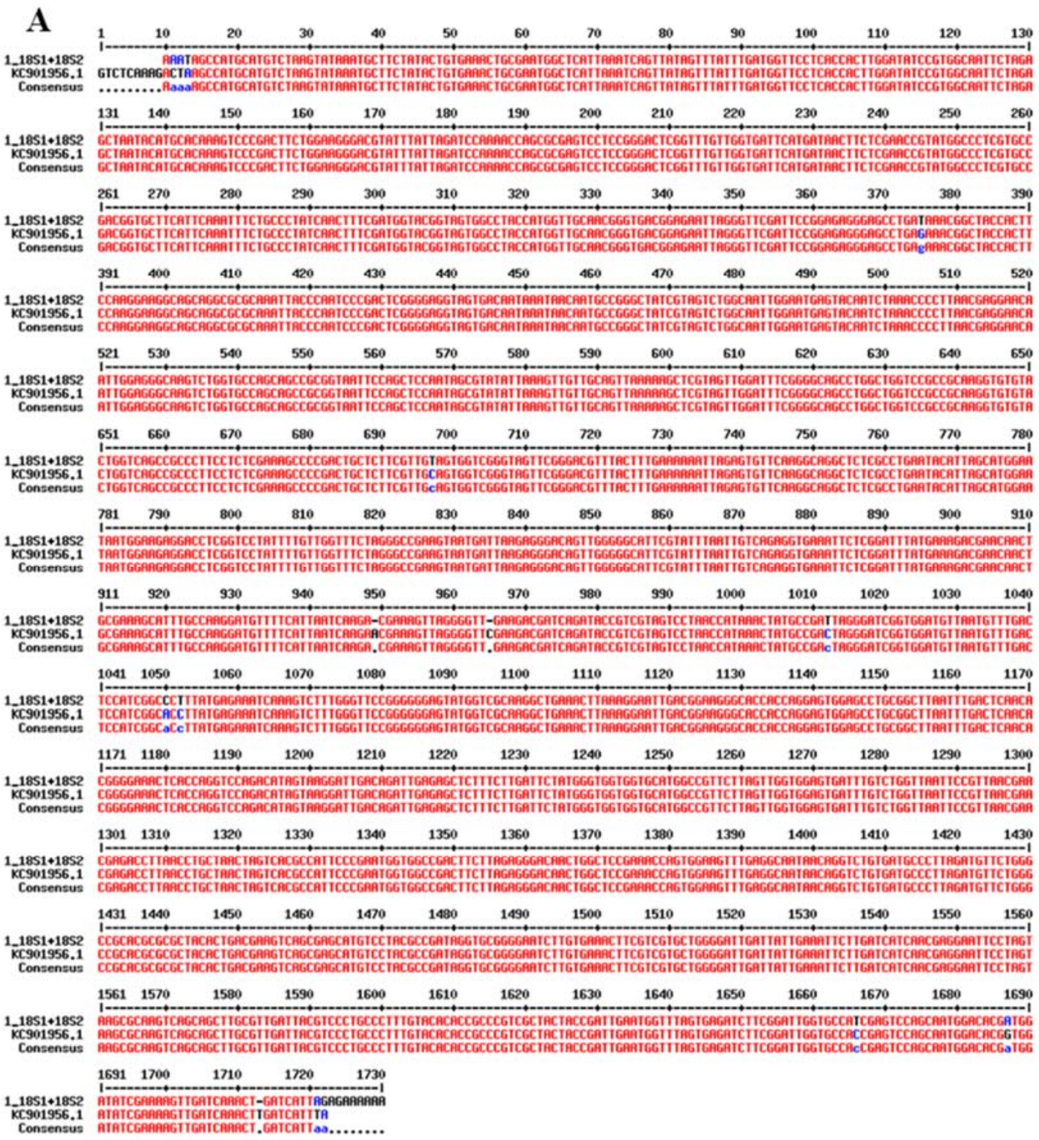

B

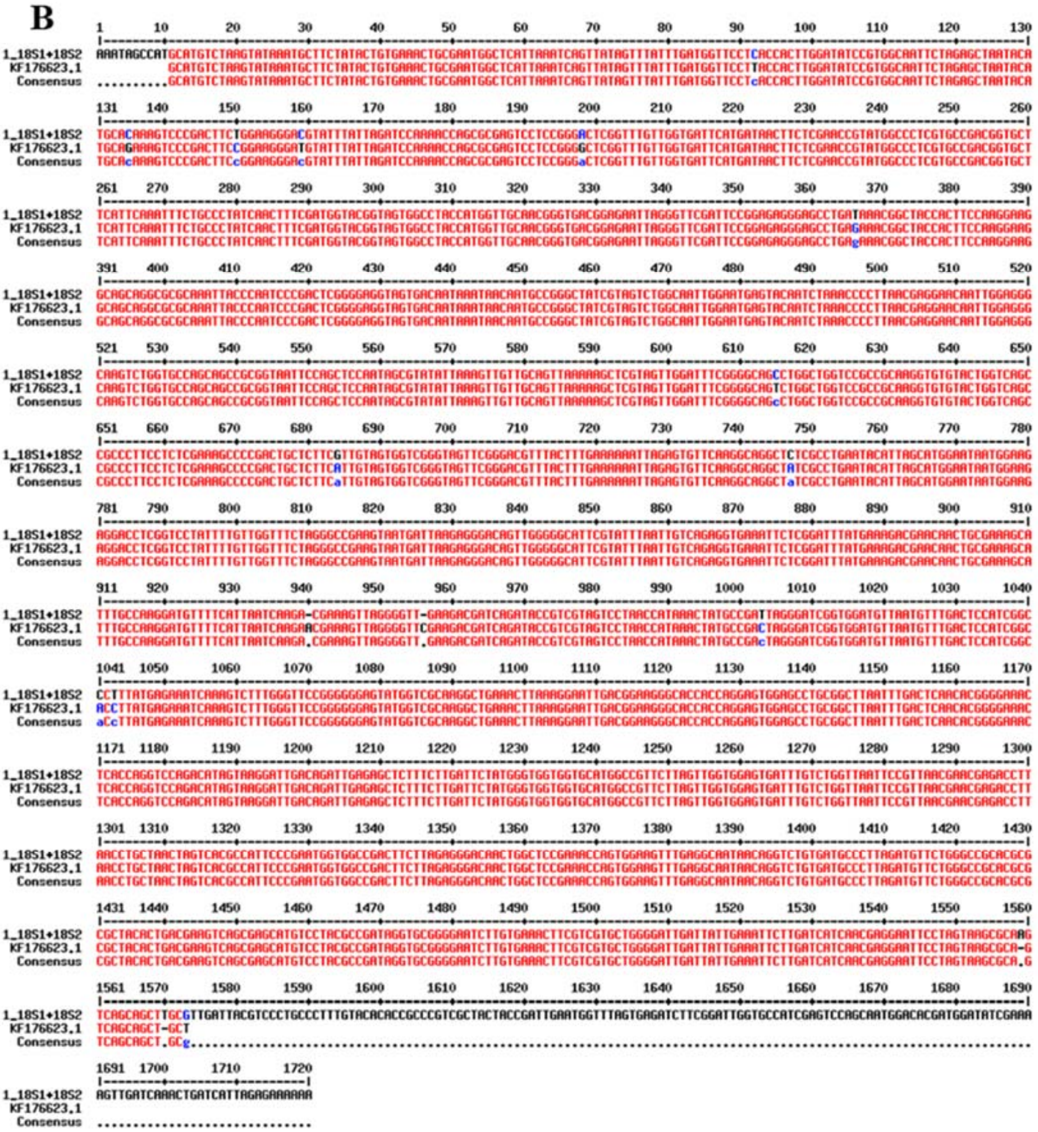

C

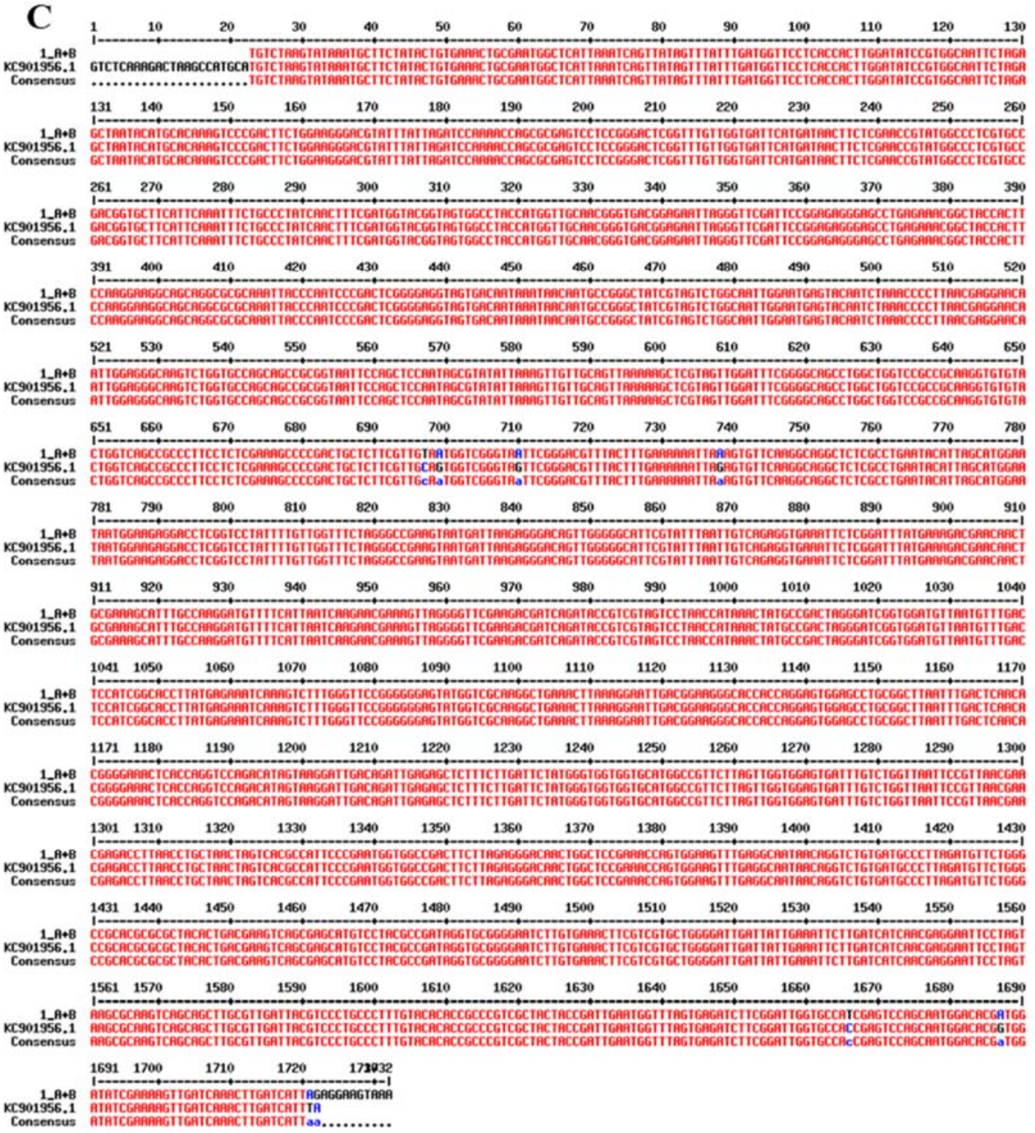

D

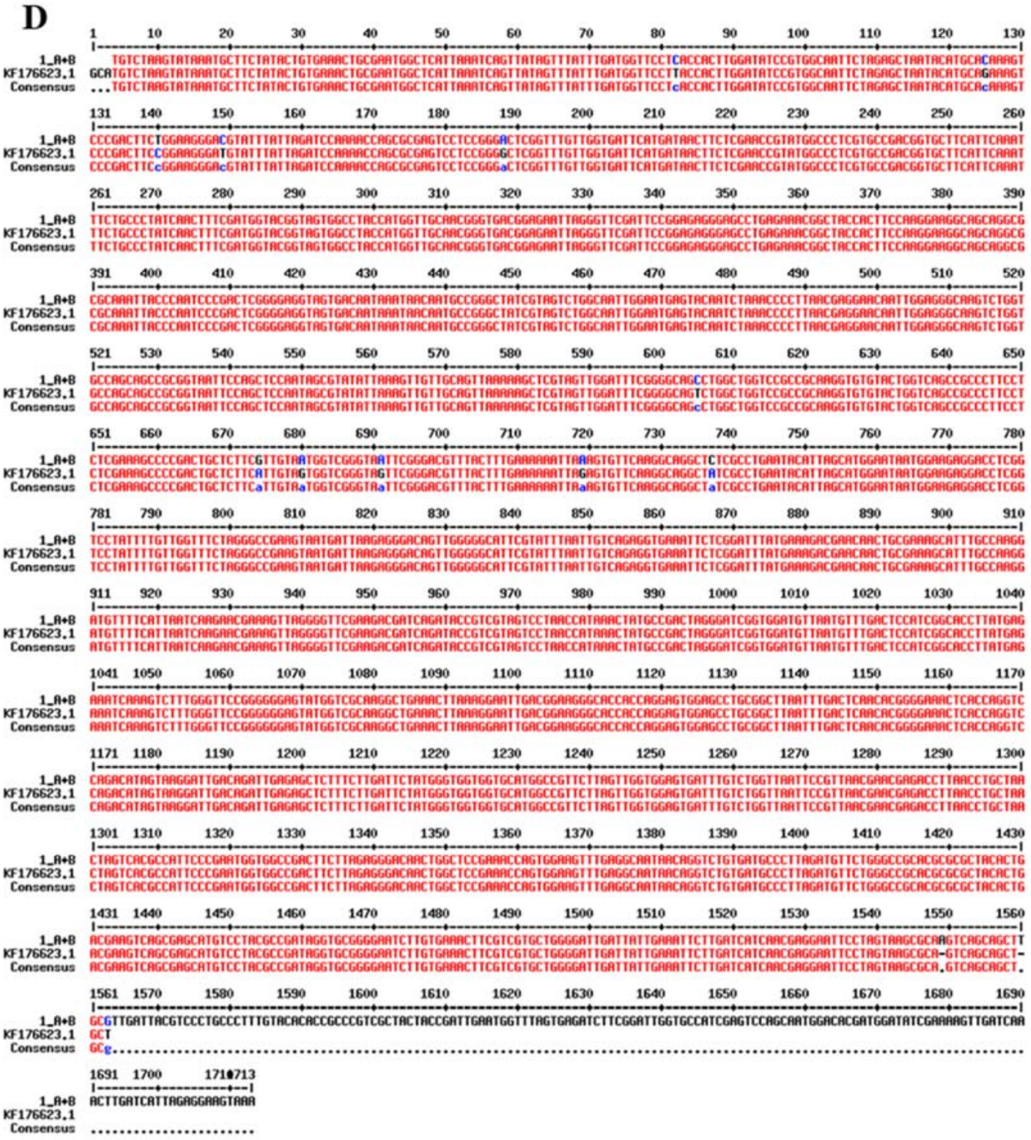

E

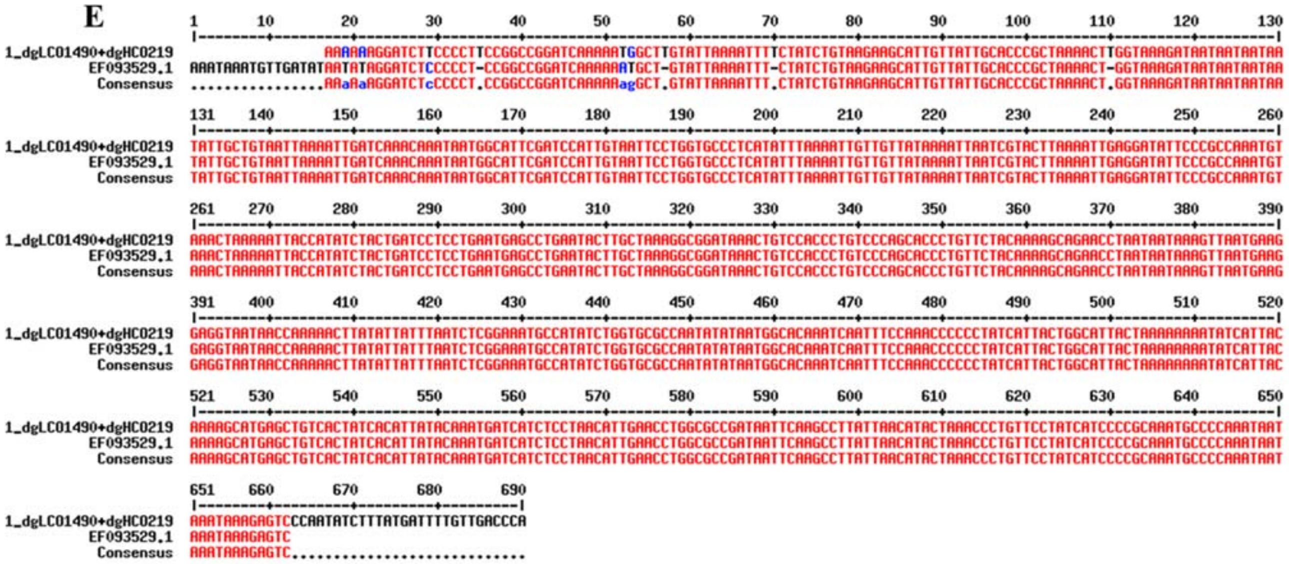

Figure S6. Sample A.dam. Alignments of PCR fragments obtained with A/B primer pairs aligned to (A) the first hit (*H. tipica*) and (B) (*A. damicornis*) corresponding to the species as identified from morphological analysis.

A

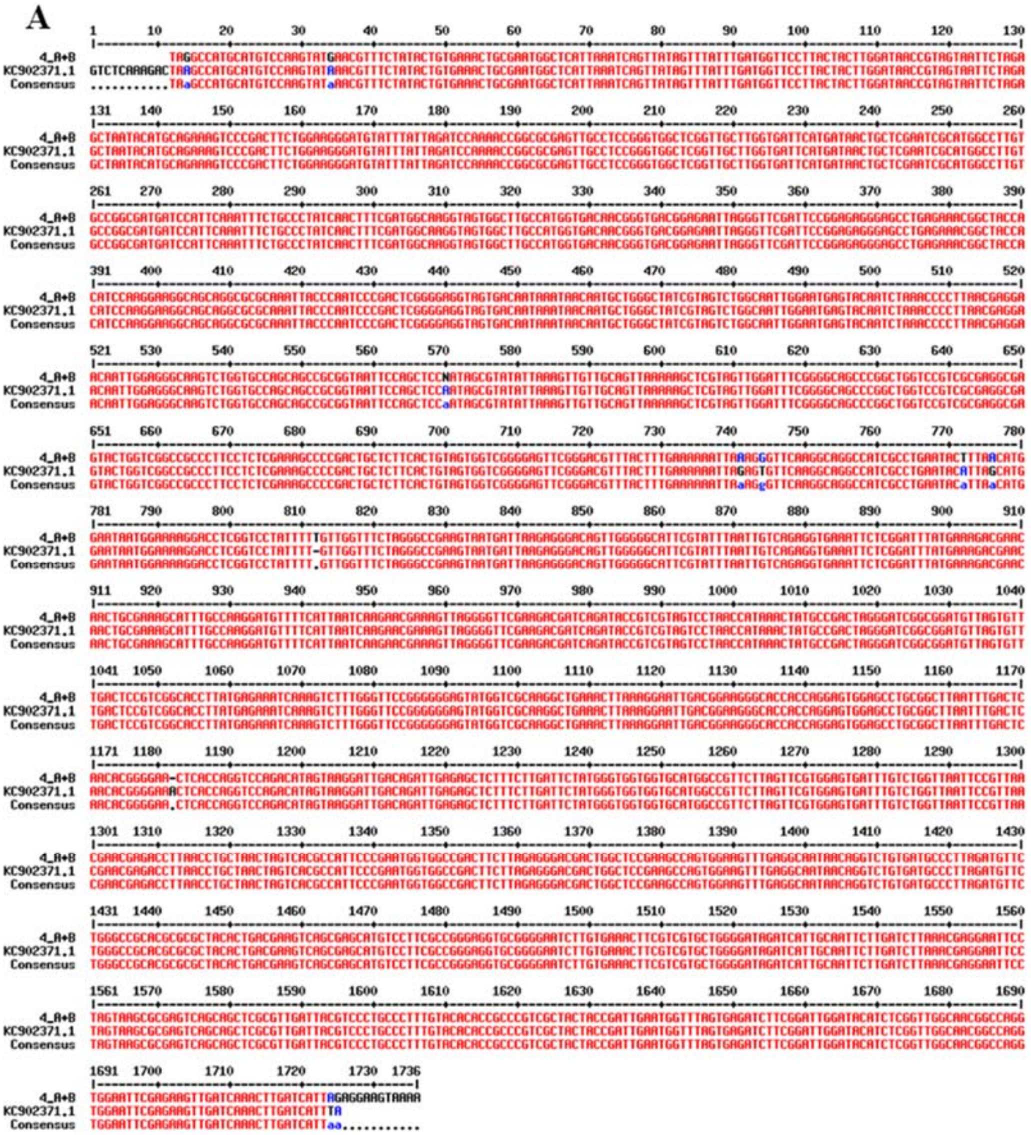

B

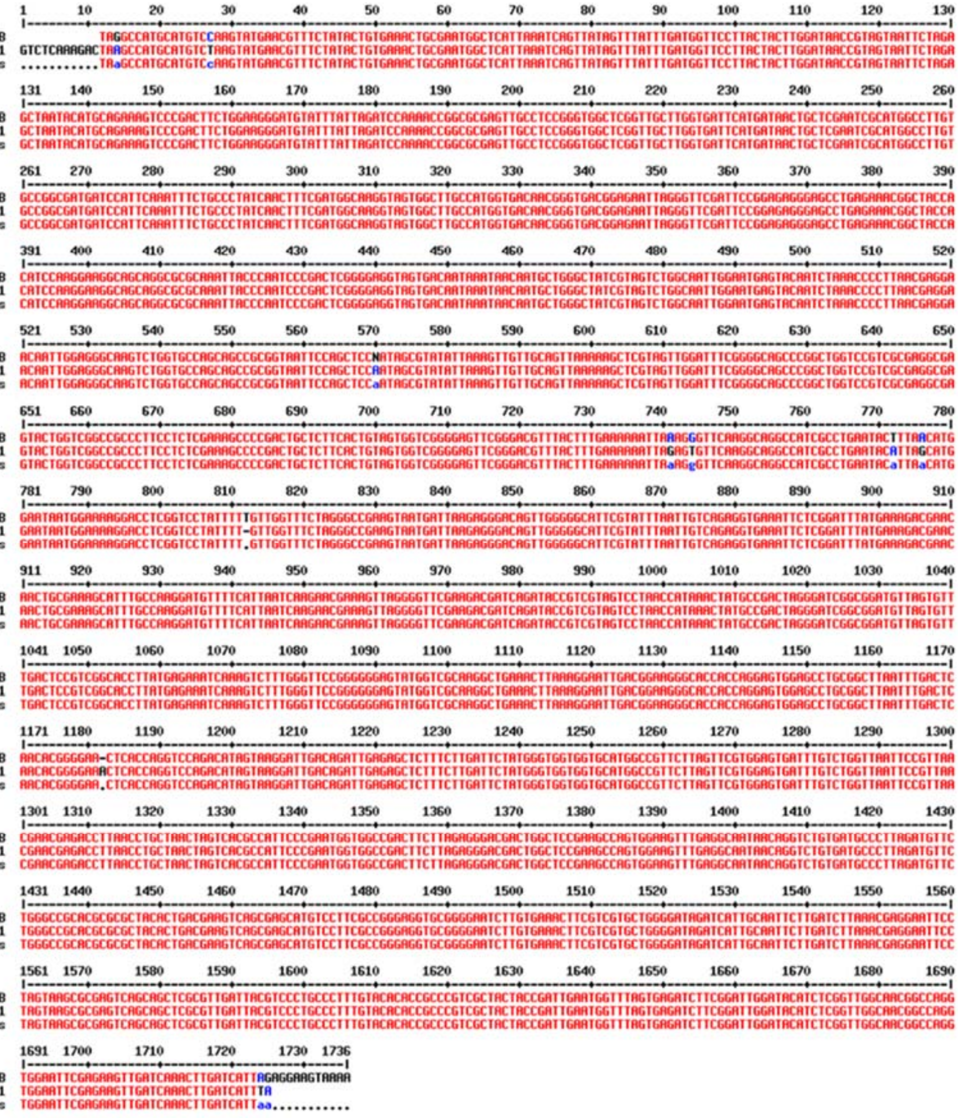

Figure S7. Sample A.acu. Alignments of PCR fragments obtained with dgLCO1490/dgHCO2198 primer pairs aligned to (A) the first hit (*A. acuta*) corresponding to the species as identified from morphological analysis; with C2/D2 primer pairs aligned to (B) the first hit (*A. acuta*). Only 106 bp of total fragment (800 bp) of RA2/ITS2.2 primer pairs aligned to (C) the first hit (*A. acuta*).

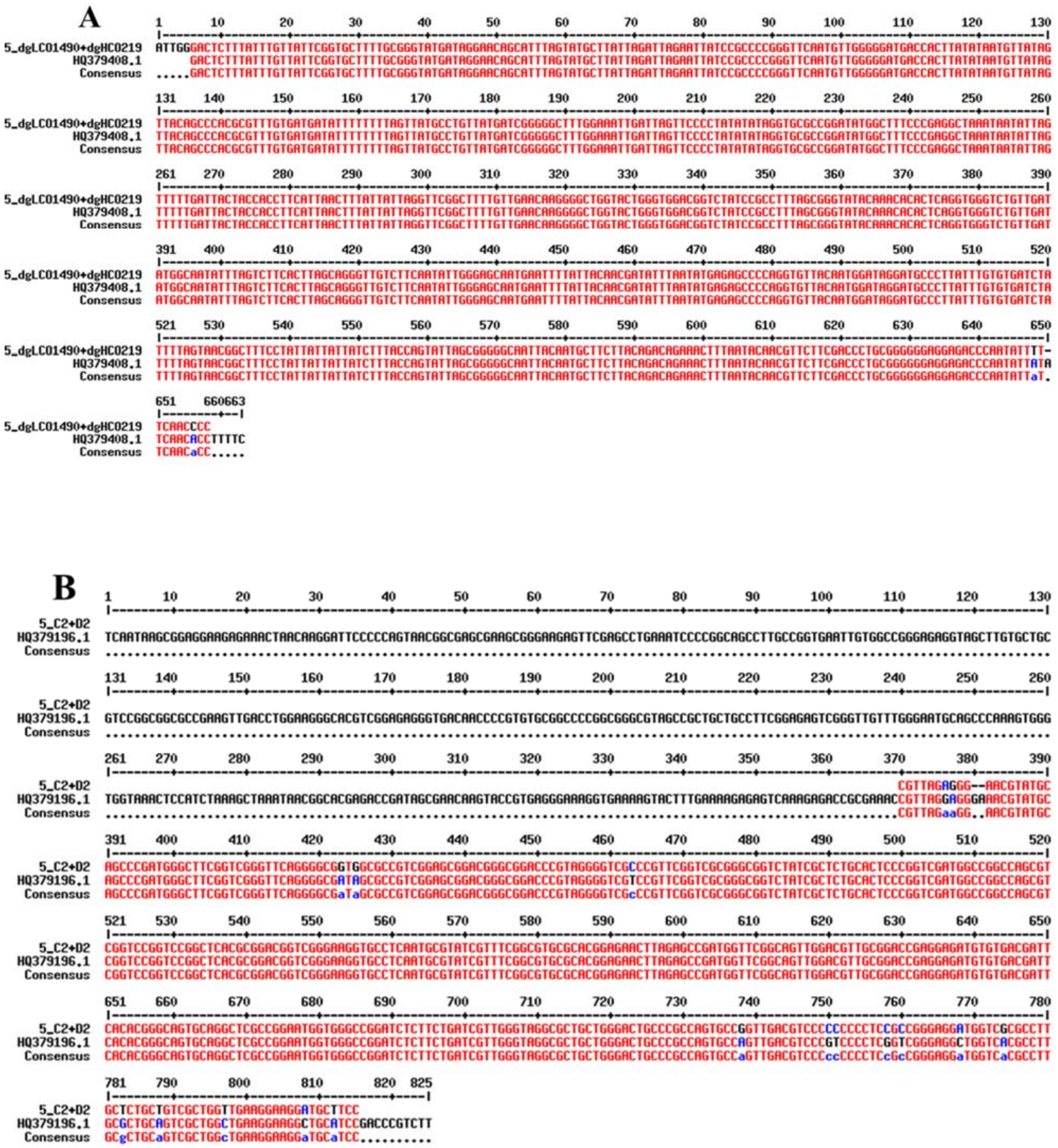

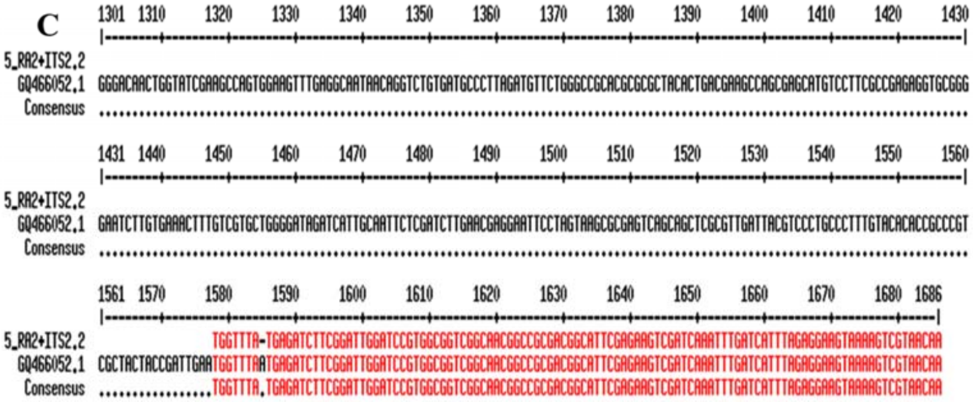
C
5_RA2+ITS2.2
GQ466052.1
Consensus

Figure S8. Alpha rarefaction of Chao1, Shannon and Simpson diversity indices. Sample IDs: O.per= *Oceanapia* cf. *perforata*, S.spi= *Sarcotragus spinosulus*, E.dis= *Erylus discophorus*, A.oro= *Agelas oroides*, T.aur= *Tethya aurantium*, A.dam= *Axinella damicornis*, A.acu= *Acanthella acuta* and G.cyd= *Geodia cydonium*.

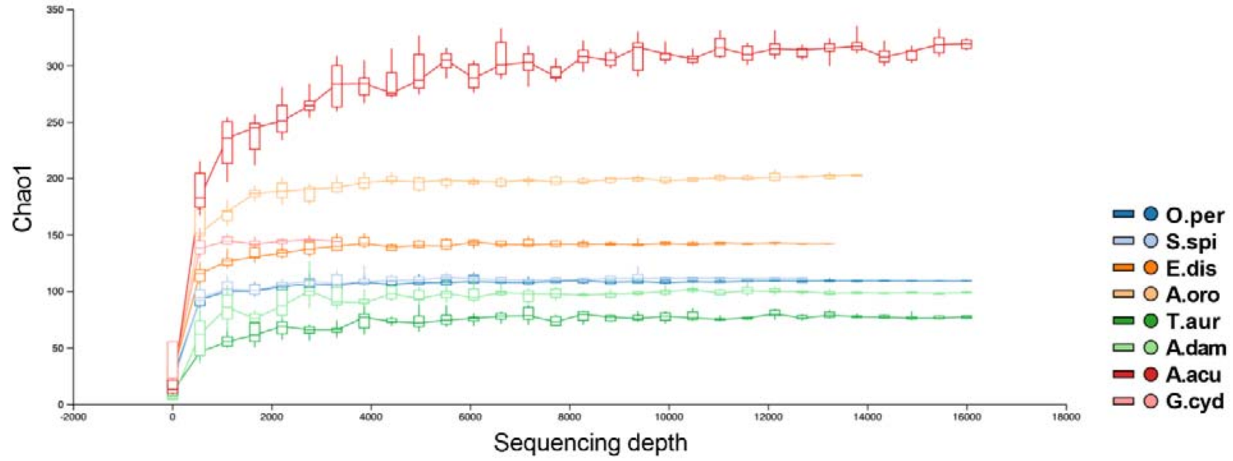

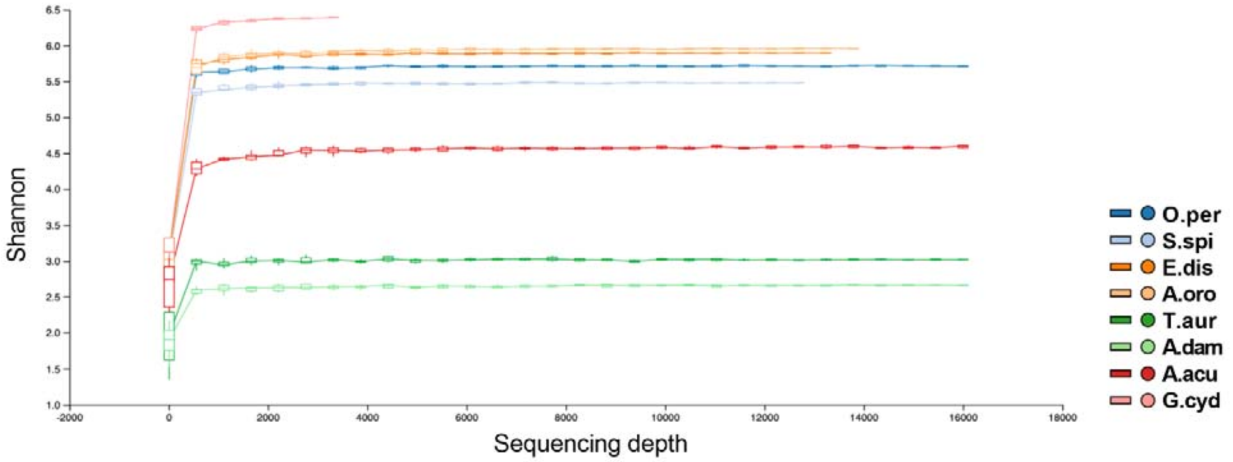

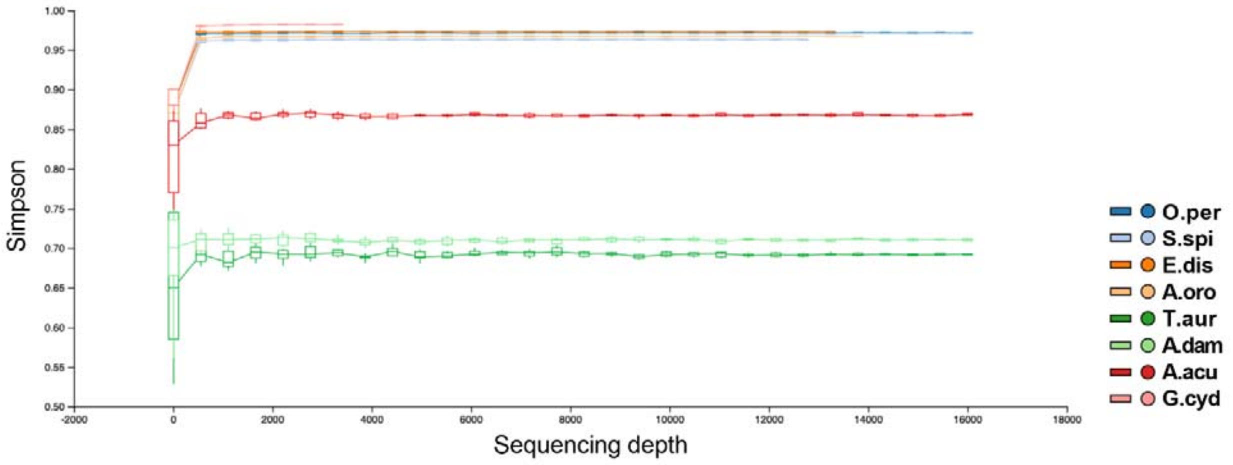

Figure S9. Taxonomy BarPlot. Sample code: O.per= *Oceanapia* cf. *perforata*, S.spi= *Sarcotragus spinosulus*, E.dis= *Erylus discophorus*, A.oro= *Agelas oroides*, T.aur= *Tethya aurantium*, A.dam= *Axinella damicornis*, A.acu= *Acanthella acuta* and G.cyd= *Geodia cydonium*. Each phylum is highlighted by a different color. Values are reported as percentage of relative frequency. Taxonomy code: R= regnum, P= phylum, C= class. Phylum belonging to Archea is indicated with ***.

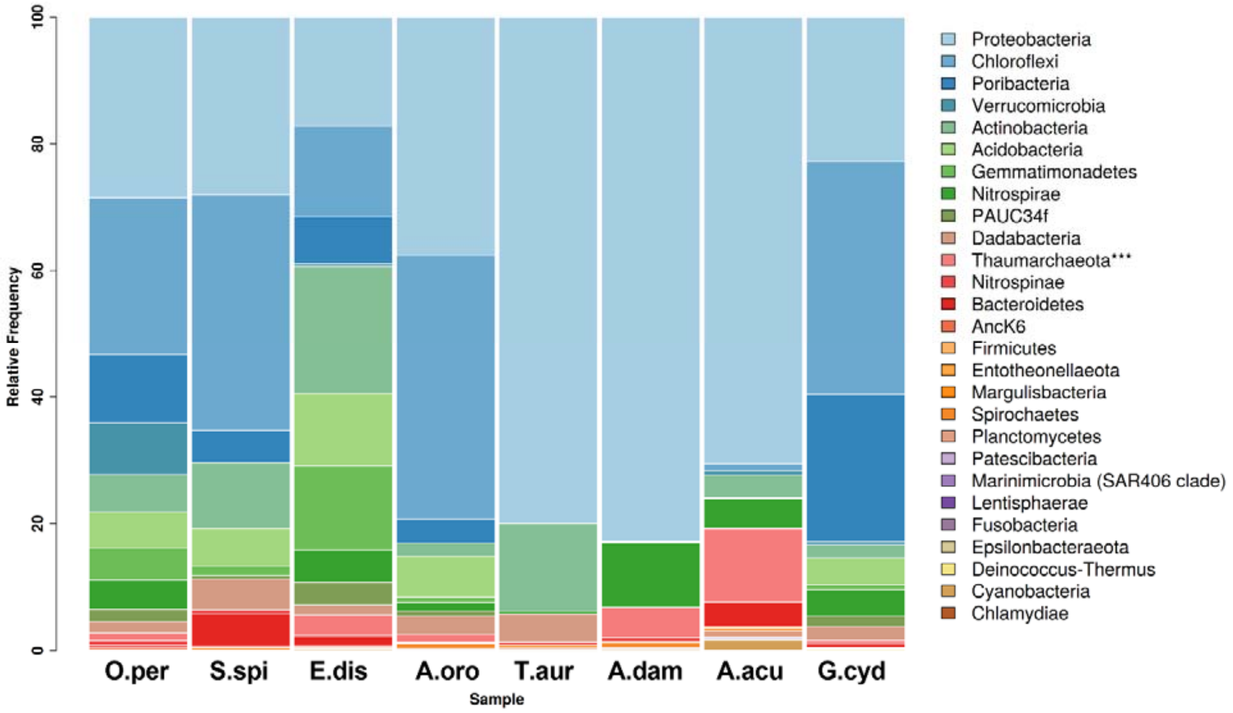

Figure S10. Distribution of ASV's frequencies.

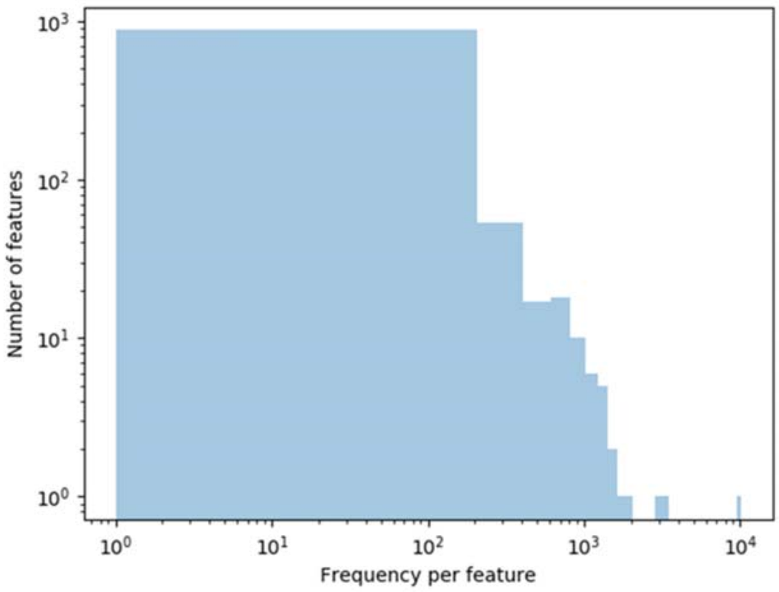

Figure S11. Distribution of ASV's frequencies for each sample (reported as a blue bar).

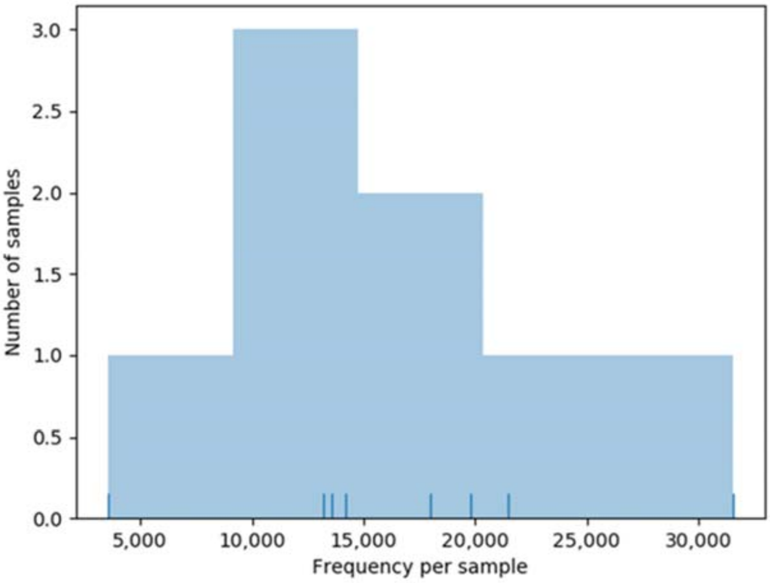

Supplement: Supplementary file 1 — Supplementary Information. [file 41598_2021_713_MOESM1_ESM.pdf]
